# The simulation experiment description markup language (SED-ML): language specification for level 1 version 5

**DOI:** 10.1515/jib-2024-0008

**Published:** 2024-04-15

**Authors:** Lucian P. Smith, Frank T. Bergmann, Alan Garny, Tomáš Helikar, Jonathan Karr, David Nickerson, Herbert Sauro, Dagmar Waltemath, Matthias König

**Affiliations:** 7284 University of Washington, Seattle, USA; BioQUANT/COS, 132140 Heidelberg University, Heidelberg, Germany; Auckland Bioengineering Institute, 428614 The University of Auckland, Auckland, New Zealand; University of Nebraska, Lincoln, USA; 5925 Icahn School of Medicine at Mount Sinai, New York, USA; University of Greifswald, Greifswald, Germany; 9373 Humboldt University, Berlin, Germany

## Abstract

Modern biological research is increasingly informed by computational simulation experiments, which necessitate the development of methods for annotating, archiving, sharing, and reproducing the conducted experiments. These simulations increasingly require extensive collaboration among modelers, experimentalists, and engineers. The Minimum Information About a Simulation Experiment (MIASE) guidelines outline the information needed to share simulation experiments. SED-ML is a computer-readable format for the information outlined by MIASE, created as a community project and supported by many investigators and software tools. Level 1 Version 5 of SED-ML expands the ability of modelers to define simulations in SED-ML using the Kinetic Simulation Algorithm Onotoloy (KiSAO). While it was possible in Version 4 to define a simulation entirely using KiSAO, Version 5 now allows users to define tasks, model changes, ranges, and outputs using the ontology as well. SED-ML is supported by a growing ecosystem of investigators, model languages, and software tools, including various languages for constraint-based, kinetic, qualitative, rule-based, and spatial models, and many simulation tools, visual editors, model repositories, and validators. Additional information about SED-ML is available at https://sed-ml.org/.

***Corresponding author: Lucian P. Smith**, University of Washington, Seattle, USA, E-mail: lpsmith@uw.edu. https://orcid.org/0000-0001-7002-6386
**Frank T. Bergmann**, BioQUANT/COS, Heidelberg University, Heidelberg, Germany. https://orcid.org/0000-0001-5553-4702
**Alan Garny and David Nickerson**, Auckland Bioengineering Institute, The University of Auckland, Auckland, New Zealand. https://orcid.org/0000-0001-7606-5888 (A. Garny), https://orcid.org/0000-0003-4667-9779 (D. Nickerson)
**Tomáš Helikar**, University of Nebraska, Lincoln, USA. https://orcid.org/0000-0003-3653-1906
**Jonathan Karr**, Icahn School of Medicine at Mount Sinai, New York, USA. https://orcid.org/0000-0002-2605-5080
**Herbert Sauro**, University of Washington, Seattle, USA. https://orcid.org/0000-0002-3659-6817
**Dagmar Waltemath**, University of Greifswald, Greifswald, Germany. https://orcid.org/0000-0002-5886-5563
**Matthias König**, Humboldt University, Berlin, Germany. https://orcid.org/0000-0003-1725-179X

## Simulation Experiment Description Markup Language (SED-ML) : Level 1 Version 5

January 3, 2024

**Editors**

| | |
|---|---|
| Lucian Smith | *University of Washington, USA* |
| Frank T Bergmann | *BioQUANT/COS, Heidelberg University, Germany* |
| Alan Garny | *Auckland Bioengineering Institute, New Zealand* |
| Tomáš Helikar | *University of Nebraska-Lincoln, USA* |
| Jonathan Karr | *Icahn School of Medicine at Mount Sinai, USA* |
| David Nickerson | *Auckland Bioengineering Institute, New Zealand* |
| Herbert Sauro | *University of Washington, USA* |
| Dagmar Waltemath | *University of Greifswald, Germany* |
| Matthias König | *Humboldt University of Berlin, Germany* |

To find the most up to date information about SED-ML, including links to published SED-ML files, examples, and supporting software, please visit
https://sed-ml.org/

The latest release of the Level 1 Version 5 specification is available at
https://identifiers.org/combine.specifications/sed-ml.level-1.version-5

To discuss SED-ML and the SED-ML specification write to the mailing list
sed-ml-discuss@googlegroups.com.

To contact the SED-ML editors write to sed-ml-editors@googlegroups.com.

1 Introduction 5
1.0.1 Color conventions in this document 5
1.1 SED-ML overview 5
1.2 Example simulation experiment 6
1.2.1 Time-course simulation 7
1.2.2 Applying pre-processing 7
1.2.3 Applying post-processing 8

2 SED-ML technical specification 9
2.1 General data types and classes 9
2.1.1 Primitive data types 9
2.1.1.1 ID 10
2.1.1.2 SId 10
2.1.1.3 SIdRef 10
2.1.1.4 TargetType 10
2.1.1.5 XPath 10
2.1.1.6 MathML 10
2.1.1.7 anyURI 11
2.1.1.8 URN 11
2.1.1.9 NuMLSId 11
2.1.1.10 NuMLSIdRef 11
2.1.1.11 CurveType 11
2.1.1.12 SurfaceType 11
2.1.1.13 LineType 11
2.1.1.14 SedColor 11
2.1.1.15 MarkerType 11
2.1.1.16 MappingType 12
2.1.1.17 ExperimentType 12
2.1.1.18 AxisType 12
2.1.1.19 ScaleType 12
2.1.2 Reference relations 12
2.1.2.1 modelReference 13
2.1.2.2 simulationReference 13
2.1.2.3 taskReference 13
2.1.3 listOf* containers 14
2.1.4 SEDBase 14
2.1.5 Notes 15
2.1.6 Annotation 16
2.1.7 Algorithm 16
2.1.7.1 AlgorithmParameter 17
2.1.8 Calculation 18
2.1.8.1 Math 19
2.1.8.2 Parameter 20
2.1.8.3 Variable 20
2.1.8.4 AppliedDimension 24
2.2 SED-ML Components 26
2.2.1 SED-ML top level element 26
2.2.1.1 xmlns 28
2.2.1.2 level 28
2.2.1.3 version 28
2.2.1.4 listOfDataDescriptions 28
2.2.1.5 listOfModels 28
2.2.1.6 listOfSimulations 29
2.2.1.7 listOfTasks 29
2.2.1.8 listOfDataGenerators 29
2.2.1.9 listOfOutputs 30
2.2.1.10 listOfStyles 30
2.2.1.11 listOfAlgorithmParameters (global) 30
2.2.2 DataDescription 30
2.2.3 DataDescription components 32
2.2.3.1 DimensionDescription 32
2.2.3.2 DataSource 32
2.2.3.3 Slice 33
2.2.4 Model 34
2.2.5 Change 36
2.2.5.1 NewXML 37
2.2.5.2 AddXML 37
2.2.5.3 ChangeXML 38
2.2.5.4 RemoveXML 38

2.2.5.5 ChangeAttribute . . . 38
2.2.5.6 ComputeChange . . . 39
2.2.6 Simulation . . . 40
2.2.6.1 UniformTimeCourse . . . 41
2.2.6.2 OneStep . . . 42
2.2.6.3 SteadyState . . . 42
2.2.6.4 Analysis . . . 42
2.2.7 AbstractTask . . . 43
2.2.7.1 Task . . . 43
2.2.7.2 Repeated Task . . . 44
2.2.8 Task components . . . 46
2.2.8.1 SubTask . . . 46
2.2.8.2 SetValue . . . 47
2.2.8.3 Range . . . 47
2.2.9 ParameterEstimationTask . . . 50
2.2.9.1 Objective . . . 51
2.2.9.2 LeastSquareObjectiveFunction . . . 51
2.2.9.3 AdjustableParameter . . . 52
2.2.9.4 Bounds . . . 52
2.2.9.5 ExperimentReference . . . 53
2.2.9.6 FitExperiment . . . 53
2.2.9.7 FitMapping . . . 54
2.2.10 DataGenerator . . . 54
2.2.11 Output . . . 55
2.2.12 Plot . . . 56
2.2.12.1 Plot2D . . . 58
2.2.12.2 Plot3D . . . 58
2.2.12.3 Axis . . . 58
2.2.12.4 AbstractCurve . . . 60
2.2.12.5 Curve . . . 61
2.2.12.6 ShadedArea . . . 61
2.2.12.7 Surface . . . 62
2.2.12.8 ParameterEstimationResultPlot . . . 63
2.2.12.9 WaterfallPlot . . . 64
2.2.13 Report . . . 64
2.2.13.1 DataSet . . . 64
2.2.14 ParameterEstimationReport . . . 65
2.2.15 Figure . . . 65
2.2.15.1 SubPlot . . . 66
2.2.16 Style . . . 67
2.2.16.1 Line . . . 68
2.2.16.2 Marker . . . 69
2.2.16.3 Fill . . . 70

**3 Concepts used in SED-ML** **71**
3.1 MathML . . . 71
3.1.1 MathML elements . . . 71
3.1.2 MathML symbols . . . 71
3.1.2.1 MathML csymbols for dimensional input . . . 71
3.1.2.2 MathML Distribution Functions . . . 72
3.1.3 NA values . . . 73
3.2 URI scheme . . . 74
3.2.1 Model references . . . 74
3.2.2 Data references . . . 74
3.2.3 Symbols . . . 74
3.2.4 Annotation Scheme . . . 75
3.3 URN scheme . . . 75
3.3.1 Language references . . . 75
3.3.2 Data format references . . . 75
3.3.2.1 NuML (Numerical Markup Language) . . . 75
3.3.2.2 CSV (Comma Separated Values) . . . 76
3.3.2.3 TSV (Tab Separated Values) . . . 77
3.3.2.4 HDF5 (Hierarchical Data Format version 5) . . . 78
3.4 XPath . . . 78
3.5 NuML . . . 79
3.6 KiSAO . . . 79
3.7 COMBINE archive . . . 79
3.8 SED-ML resources . . . 79

4 Acknowledgements 80

A Examples 82
A.1 Example simulation experiment (L1V3_repressilator.omex) . . . 82
A.2 Simulation experiments with dataDescriptions . . . 85
A.2.1 Plotting data with simulations (L1V3_plotting-data-numl.omex) . . . 85
A.3 Simulation experiments with repeatedTasks . . . 87
A.3.1 Time course parameter scan (L1V3_repeated-scan-oscli.omex) . . . 87
A.3.2 Steady state parameter scan (L1V3_repeated-steady-scan-oscli.omex) . . . 88
A.3.3 Stochastic simulation (L1V3_repeated-stochastic-runs.omex) . . . 90
A.3.4 Simulation perturbation (L1V3_oscli-nested-pulse.omex) . . . 91
A.3.5 2D steady state parameter scan (L1V3_parameter-scan-2d.omex) . . . 93
A.4 Simulation experiments with different model languages . . . 97
A.4.1 Van der Pol oscillator in SBML (L1V3_vanderpol-sbml.omex) . . . 97
A.4.2 Van der Pol oscillator in CellML (L1V3_vanderpol-cellml.omex) . . . 99
A.5 Reproducing publication results . . . 102
A.5.1 Le Loup model (L1V3_leloup-sbml.omex) . . . 102
A.5.2 IkappaB signaling (L1V3_ikkapab.omex) . . . 104

B Validation 106
B.1 Validation of SED-ML documents . . . 106
B.1.1 Validation and consistency rules . . . 106

## 1. Introduction

The Simulation Experiment Description Markup Language (SED-ML) is an XML-based format for describing simulation experiments, including model changes, calibrations, simulations, analyses, and computations and visualizations of simulation results.

The number of computational models of biological systems is growing at an ever increasing pace. At the same time, their size and complexity are also increasing. It is now generally accepted that one must be able to exchange the mathematical structure of such models, for instance to build on existing studies by reusing models or to reproduce model results. The efforts to standardize the representation of computational models in various areas of biology, such as the Systems Biology Markup Language (SBML) [16], CellML [9] or NeuroML [13], resulted in an increase of the exchange and re-use of models. However, the description of models is not sufficient to reproduce, reuse, and combine simulation experiments and their results. One also needs to describe the procedures the models are subjected to, i.e., the information required to reproduce simulation experiments among users and software tools. The increasing use of computational simulation experiments to inform modern biological research creates new challenges to reproduce, annotate, archive, and share such experiments.

SED-ML is a computer-readable exchange format for describing simulation experiments. Critically, SED-ML can be used with a wide range of model formats, modeling frameworks, simulation algorithms, and simulation tools. For many simulation experiments, SED-ML can capture the information in the Minimum Information About a Simulation Experiment (MIASE) [21] guidelines.

SED-ML is developed as a community project and defined via this technical specification and a corresponding XML Schema.

This document describes Level 1 Version 5 of SED-ML, which is the successor of Level 1 Version 4 (published in [19] and originally described in [22]).

#### 1.0.1 Color conventions in this document

Throughout this document, we use coloring to carry additional information for the benefit of those viewing the document on media that can display color:

- We use a red color in text and a dark blue color in figures to indicate changes between this version of the specification, namely SED-ML Level 1 Version 4, and the *most recent previous release* of the specification (which, for the present case, is SED-ML Level 1 Version 3). The changes may be either additions or deletions of text; in the case of deletions, entire sentences, paragraphs or sections are colored to indicate a change has occurred inside them. In UML diagrams, blue text and/or lines are used to indicate semantic changes or additions.

- We use a blue color in text to indicate a hyperlink from one point in this document to another. When viewed in electronic form, clicking on blue-colored text will cause a jump to the section, figure, table or page to which the link refers.

### 1.1 SED-ML overview

SED-ML specifies for a given simulation experiment

- which datasets to use (DataDescription);

- which models to use (Model);

- which modifications to apply to models before simulation or other analyses (Change);
- which simulation, model calibration, or other analysis procedures to run on each model (Simulation and *AbstractTask*);
- how to post-process the results of these simulations and analysis (DataGenerator); and
- how these results should be presented and exported as figures and tables (Output).

A SED-ML document contains the following main objects to describe this information: DataDescription, Model, Simulation, *AbstractTask*, DataGenerator, and Output.

##### *DataDescription*

The DataDescription class enables investigators to specify datasets for a simulation experiment. Such data can be used for instance to parameterize model simulations or to plot data together with simulation results.

##### *Model*

The Model class enables investigators to describe the externally-defined models involved in a simulation experiment.

##### *Simulation*

The Simulation class enables investigators to describe the settings required for each simulation and analysis. These includes information such as the type of each simulation/analysis, the algorithm required to execute the simulation/analysis, and the algorithm parameters that the simulation/analysis should be executed with.

##### AbstractTask

The *AbstractTask*'s three child classes (Task, RepeatedTask, and ParameterEstimationTask) enable investigators to specify an execution of an experiment through a combination of a Simulation or FitExperiment with a Model. This allows investigators to describe a variety of different experiment formats in a flexible way.

##### *DataGenerator*

The DataGenerator class enables investigators to encode post-processing of simulation/analysis results before the generation of outputs, e.g., one can normalize the result of an observable, or calculate the mean of several observables. In the definition of a DataGenerator, any addressable variable or parameter of any model or DataSource may be referenced, and used to calculate new values using MathML.

##### *Output*

The Output class enables investigators to describe how post-processing simulation results should be exported, such as with two dimensional plots (Plot2D), three dimensional plots (Plot3D), and data tables (Report).

Detailed technical information about these classes is available in Chapter 2.

### 1.2 Example simulation experiment

This section illustrates an example simulation experiment for the repressilator model [10]. The corresponding SED-ML is listed in Appendix A.1. A COMBINE archive for this simulation experiment is available as `L1V3_repressilator.omex` at `https://sed-ml.org/`.

Additional example SED-ML files are available in Appendix A. Numerous complete examples with model files are available as COMBINE archives at `https://sed-ml.org/`.

The repressilator is a synthetic oscillating network of transcription regulators in *Escherichia coli*. The network is composed of the three repressor genes Lactose Operon Repressor (`lacI`), Tetracycline Repressor (`tetR`) and Repressor CI (`cI`), which code for proteins binding to the promoter of the other, blocking their transcription. The three inhibitions form a cyclic negative-feedback loop. To describe the interactions of the molecular species involved in the network, the authors built a simple mathematical model of coupled first-order differential equations. All six molecular species included in the network (three mRNAs, three repressor proteins) participate in creation (transcription/translation) and degradation processes. The model was used to determine the influence of the various parameters on the dynamic behavior of the system. In particular, parameter values were sought which induce stable oscillations in the concentrations of the system components.

#### 1.2.1 Time-course simulation

Figure 1c of the reference publication [10] (reproduced with SED-ML in Figure 1.1 and Figure 1.2) reported the following simulation.

- The Model imports the repressilator model identified by the Unified Resource Identifier (URI) [3] `https://www.ebi.ac.uk/biomodels/model/download/BIOMD0000000012?filename=BIOMD0000000012_url.xml`.
- The Simulation defines a uniform time course simulation for 1000 minutes, recording output each minute, using CVODE (KISAO:0000019).
- Several DataGenerator elements and an Output are used to define a 2D plot of `lacI`, `tetR` and `cI` against time.

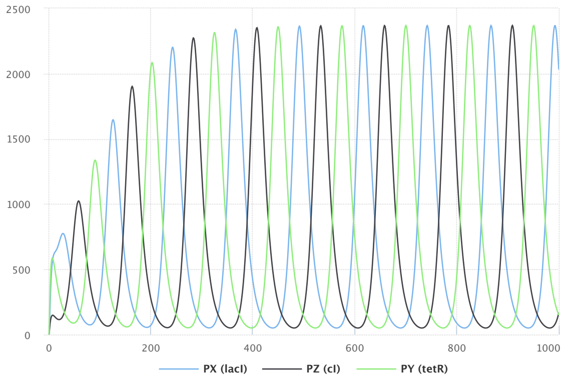

**Figure 1.1:** *Time-course simulation of the repressilator depicting repressor proteins lacI, tetR and cI. Simulation with SED-ML web tools [2].*

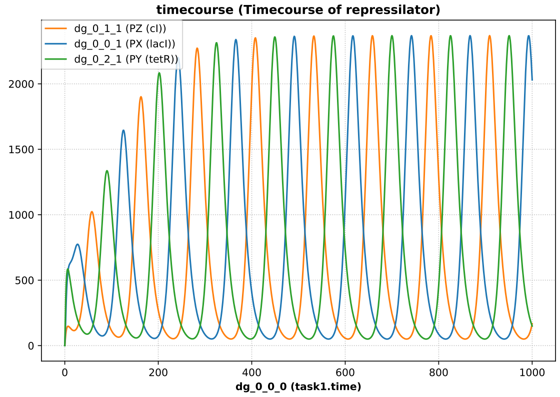

**Figure 1.2:** *Time-course simulation of the repressilator depicting repressor proteins lacI, tetR and cI. Simulation with tellurium [6].*

#### 1.2.2 Applying pre-processing

A common step in a simulation experiment is the modification of model parameters before simulation. When changing the parameter values for the protein copies per promoter, `tps_repr`, and the leakiness in protein copies per promoter, `tps_active`, as illustrated below, the system's behavior switches from sustained oscillations to damped oscillations (Figure 1.3 on the following page and Figure 1.4 on the next page).

- Everything is defined as in Section 1.2.1 above.
- Two child Change elements of the Model are defined that change the value of the parameter `tps_repr` from `0.0005` to `1.3e-05`, and `tps_active` from `0.5` to `0.013`.
- The same Simulation, DataGenerator, and Output elements are used.

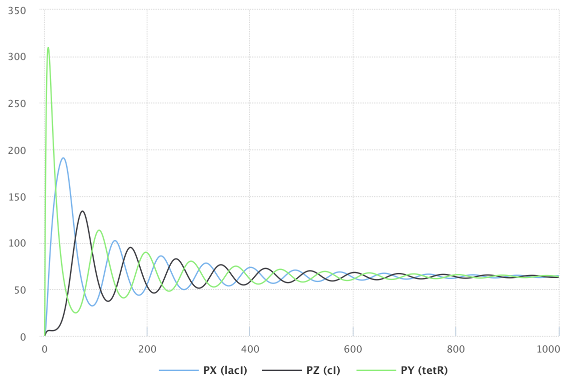

**Figure 1.3:** *Time-course simulation of the repressilator after changing parameters* `tps_repr` *and* `tps_active`*. Simulation with SED-ML web tools [2].*

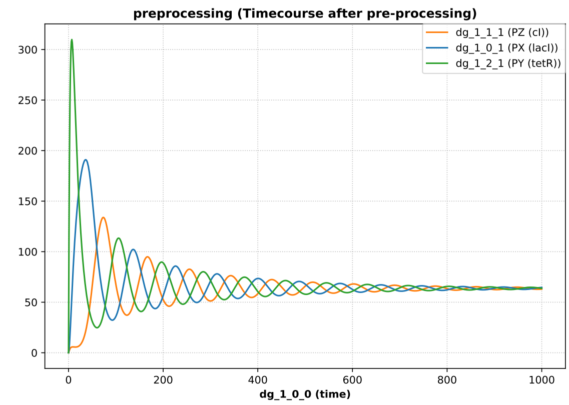

**Figure 1.4:** *Time-course simulation of the repressilator after changing parameters* `tps_repr` *and* `tps_active`*. Simulation with tellurium [6].*

#### 1.2.3 Applying post-processing

In a simulation experiment, the raw numerical output of simulations must often be post-processed before plotting or reporting. Normalized plots (Figure 1.5 and Figure 1.6) of the results of the first simulation (Section 1.2.1), which highlights the influence of the variables on each other (in phase-plane), can be constructed as follows:

- Everything is initially defined as in Section 1.2.1 above.
- Three new DataGenerator elements are defined that normalize the values of `lacI(t)`, `tetR(t)` and `cI(t)` by dividing each by their respective maximums.
- A new 2D plot Output is created where these normalized values are plotted against each other: `lacI` vs. `cI`, `cI` vs. `tetR`, and `tetR` vs. `lacI`.

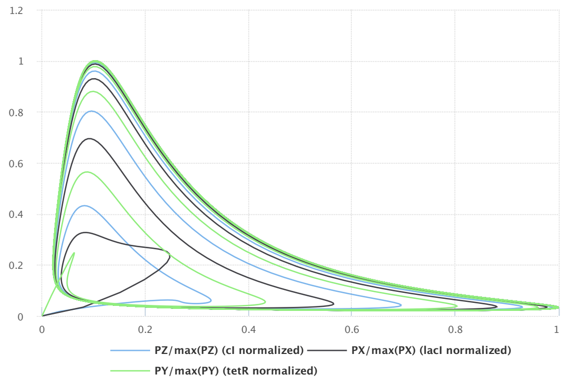

**Figure 1.5:** *Time-course simulation of the repressilator. Normalized* `lacI`*,* `tetR` *and* `cI` *in phase-plane. Simulation with SED-ML web tools [2].*

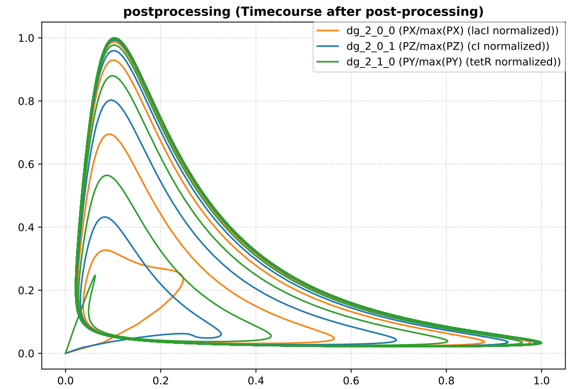

**Figure 1.6:** *Time-course simulation of the repressilator. Normalized* `lacI`*,* `tetR` *and* `cI` *in phase-plane. Simulation with tellurium [6].*

## 2. SED-ML technical specification

This document is the technical specification of SED-ML Level 1 Version 5. The corresponding UML class diagrams are included throughout this document. Please note, the UML diagrams do not capture all concepts of SED-ML. Example simulation experiments encoded with SED-ML are provided in Appendix A. Complete examples with model files are available at `https://sed-ml.org/`.

### 2.1 General data types and classes

This section introduces concepts used repeatedly throughout the specification of SED-ML. This includes primitive data types, reference relations, and base classes (*SEDBase*, Notes, Annotation, Calculation, Parameter, Variable).

The main SED-ML components based on these general data types, attributes and classes are described in Section 2.2.

#### 2.1.1 Primitive data types

Primitive data types comprise the set of data types used in SED-ML classes. Most primitive types in SED-ML are taken from the data types defined in XML Schema 1.0, including `string`, `boolean`, `int`, `positiveInteger`, `double` and `XML`.

A few additional primitive types are defined by SED-ML itself, summarized in Table 2.1, and described more fully below.

| Data type | Summary | Used |
|---|---|---|
| `ID` | External unique ID | Everywhere, as 'metaid' |
| SId | Internal unique ID | Everywhere, as 'id' |
| SIdRef | Reference to an SId | All internal references |
| `TargetType` | External reference to model element | In 'target' attributes |
| `XPath` | External references to XML elements | In 'target' attributes referencing XML |
| `MathML` | Math definitions | Anywhere calculations are performed |
| `anyURI` | Reference to external resource | Variable, DataDescription and Model |
| `URN` | Externally-defined definition | DataDescription and Model |
| `NuMLSId` | An ID in a NuML element | DimensionDescription |
| `NuMLSIdRef` | Reference to a NuMLSId | DataSource, Slice |
| SedColor | Definition of RGB/RGBA for SED-ML | Line, Marker, and Fill |
| CurveType | Defined list of possible curve types | The Curve 'type' attribute |
| SurfaceType | Defined list of possible surface types | The Surface 'type' attribute |
| LineType | Defined list of possible line types | The Line 'type' attribute |
| MarkerType | Defined list of possible marker types | The Marker 'type' attribute |
| MappingType | Defined list of possible fit mapping types | The FitMapping 'type' attribute |
| ExperimentType | Defined list of possible fit experiment types | The FitExperiment 'type' attribute |
| AxisType | Defined list of possible axis types | The Axis 'type' attribute |
| ScaleType | Defined list of possible bounds scale types | The Bounds 'scale' attribute |

**Table 2.1:** *Primitive data types defined by SED-ML.*

##### 2.1.1.1 Type ID

The XML Schema 1.0 type ID is identical to the XML 1.0 type ID. The literal representation of this type consists of strings of characters restricted as summarized in Figure 2.1. For a detailed description see the SBML specification on type ID [15].

```
NameChar   ::=   letter | digit | '.'  | '-' | ' ' | ':'  | CombiningChar | Extender
ID         ::=   ( letter | ' ' | ':'  )  NameChar*
```

**Figure 2.1:** *The definition of the type ID. The characters ( and ) are used for grouping, the character * indicates "zero or more times", and the character | indicates "or". Please consult the XML 1.0 specification for the complete definitions of letter, digit, CombiningChar, and Extender.*

##### 2.1.1.2 Type SId

The type SId is the type of the id attribute found on the majority of SED-ML components. SId is a data type derived from string, but with restrictions about the characters permitted and the sequences in which those characters may appear. The definition is shown in Figure 2.2. For a detailed description see the SBML specification on type SId [15].

```
letter   ::=   'a'..'z','A'..'Z'
digit    ::=   '0'..'9'
idChar   ::=   letter | digit | '_'
SId      ::=   ( letter | '_' ) idChar*
```

**Figure 2.2:** *The definition of the type SId*

##### 2.1.1.3 Type SIdRef

Type SIdRef is used for all attributes that refer to identifiers of type SId in a model. This type is derived from SId, but with the restriction that the value of an attribute having type SIdRef must equal the value of some SId attribute. In other words, a SIdRef value must be an existing identifier.

As with SId, the equality of SIdRef values is determined by exact character sequence match; i.e., comparisons of these identifiers must be performed in a case-sensitive manner.

##### 2.1.1.4 Type TargetType

Type TargetType is used to identify elements of a model. This type is derived from type string, and it is up to model languages to define how elements encoded in the language should be identified. For XML-based languages, XPath must be used in conjuction with ChangeXML, AddXML, or RemoveXML. Model languages are encouraged to provide clear documentation about how model elements can be identified.

##### 2.1.1.5 Type XPath

Type XPath is derived from type TargetType and is used to identify nodes and attributes within an XML representation of a model. XPath in SED-ML is an XPath version 1 expression which can be used to unambiguously identify an element or attribute in an XML file. The concept of XPath is described in Section 3.4. Note, model languages can choose to use XPath to identify abstract concepts implied by models that are not defined in XML files, such as 'the current value of the object corresponding to an XML element within the state of a simulation run'.

##### 2.1.1.6 Type MathML

Type MathML is used to describe mathematical expression in MathML. The concept of MathML and the allowed subset of MathML on a MathML attribute is described in Section 3.1.

##### 2.1.1.7 Type anyURI

Type `anyURI` is used in annotations and to reference model files, data files, and implicit model variables. For a description of the uses of `anyURI` see Section 3.2. The notion of implicit variables is explained in Section 3.2.3.

##### 2.1.1.8 Type URN

Type `URN` is used to reference the model language and data description formats. It is derived from URI, is defined by the Network Working Group RFCs 1737 and 2141, and consists of a colon-delimited string beginning with "`urn:`". For a description of the uses of `URN` see Section 3.3.

##### 2.1.1.9 Type NuMLSId

The type `NuMLSId` is the type of the `id` attribute found on NuML components. `NuMLSId` is a data type derived from SId, with the same restrictions about the characters permitted and the sequences in which those characters may appear as SId. The concept of NuML is described in Section 3.5.

##### 2.1.1.10 Type NuMLSIdRef

Type `NuMLSIdRef` is used for all attributes that refer to identifiers of type `NuMLSId` in a model. This type is derived from `NuMLSId`, but with the restriction that the value of an attribute having type `NuMLSIdRef` must equal the value of some `NuMLSId` attribute. In other words, a `NuMLSIdRef` value must be an existing NuML identifier.

As with `NuMLSId`, the equality of `NuMLSIdRef` values is determined by exact character sequence match; i.e., comparisons of these identifiers must be performed in a case-sensitive manner.

##### 2.1.1.11 Type CurveType

The `CurveType` primitive data type is used in the definition of the Curve class. `CurveType` is derived from type `string` and its values are restricted to being one of the following possibilities: "`points`", "`bar`", "`barStacked`", "`horizontalBar`", and "`horizontalBarStacked`". Attributes of type `CurveType` cannot take on any other values. The meaning of these values is discussed in the context of the Curve class's definition in 2.2.12.5.

##### 2.1.1.12 Type SurfaceType

The `SurfaceType` primitive data type is used in the definition of the Surface class. `SurfaceType` is derived from type `string` and its values are restricted to being one of the following possibilities: "`parametricCurve`", "`surfaceMesh`", "`surfaceContour`", "`contour`", "`heatMap`", "`stackedCurves`", and "`bar`". Attributes of type `SurfaceType` cannot take on any other values. The meaning of these values is discussed in the context of the Surface class's definition in 2.2.12.7.

##### 2.1.1.13 Type LineType

The `LineType` primitive data type is used in the definition of the Line class. `LineType` is derived from type `string` and its values are restricted to being one of the following possibilities: "`none`", "`solid`", "`dash`", "`dot`", "`dashDot`", and "`dashDotDot`". Attributes of type `LineType` cannot take on any other values. The meaning of these values is discussed in the context of the Line class's definition in 2.2.16.1.

##### 2.1.1.14 Type SedColor

The `SedColor` primitive data type is used in the definition of various children of the Style class. `SedColor` is derived from type `string` and its values are allowed to be a six-character RGB hex value (where the alpha is assumed to be 100%), or an eight-character RGBA hex value. For example, 808000FF would be red and green 50.2%, blue 0%, and alpha 100%, i.e. a brown. Attributes of type `SedColor` cannot take on any other values.

##### 2.1.1.15 Type MarkerType

The `MarkerType` primitive data type is used in the definition of the Marker class. `MarkerType` is derived from type `string` and its values are restricted to being one of the following possibilities: "`none`",

"square", "circle", "diamond", "xCross", "plus", "star", "triangleUp", "triangleDown", "triangleLeft", "triangleRight", "hDash", and "vDash". Attributes of type MarkerType cannot take on any other values. The meaning of these values is discussed in the context of the Marker class's definition in 2.2.16.2.

##### 2.1.1.16 Type MappingType

The MappingType primitive data type is used in the definition of the FitMapping class. MappingType is derived from type string and its values are restricted to being one of the following possibilities: "time", "experimentalCondition" and "observable". Attributes of type MappingType cannot take on any other values. The meaning of these values is discussed in the context of the FitMapping class's definition in 2.2.9.7.

##### 2.1.1.17 Type ExperimentType

The ExperimentType primitive data type is used in the definition of the FitExperiment class. ExperimentType is derived from type string and its values are restricted to being one of the following possibilities: "steadyState" and "timeCourse". Attributes of type ExperimentType cannot take on any other values. The meaning of these values is discussed in the context of the FitExperiment class's definition in 2.2.9.6.

##### 2.1.1.18 Type AxisType

The AxisType primitive data type is used in the definition of the Axis class. AxisType is derived from type string and its values are restricted to being one of the following possibilities: "linear", and "log10". Attributes of type AxisType cannot take on any other values. The meaning of these values is discussed in the context of the Axis class's definition in 2.2.12.3.

##### 2.1.1.19 Type ScaleType

The ScaleType primitive data type is used in the definition of the Bounds class. ScaleType is derived from type string and its values are restricted to being one of the following possibilities: "linear", "log", and "log10". Attributes of type ScaleType cannot take on any other values. The meaning of these values is discussed in the context of the Bounds class's definition in 2.2.9.4.

#### 2.1.2 Reference relations

The reference concept is used to refer to a particular element inside the SED-ML document. It may occur as an association between:

- two Models (modelReference)
- a Variable and a Model (modelReference)
- a Variable and an *AbstractTask* (taskReference)
- a Task and the simulated Model (modelReference)
- a Task and the Simulation (simulationReference)
- an Output and a DataGenerator (dataReference)

The definition of a Task requires a reference to a particular Model object (modelReference); furthermore, the Task object must be associated with a particular Simulation object (simulationReference).

Depending on the use of the reference relation in connection with a Variable object, it may take different roles:

a. The reference association might occur between a Variable object and a Model object, e.g., if the variable is to define a Change. In that case the variable element contains a modelReference to refer to the particular model that contains the variable used to define the change.

b. If the reference is used as an association between a Variable object and an *AbstractTask* object inside the dataGenerator class, then the variable element contains a taskReference to unambiguously refer to an observable in a given task.

##### 2.1.2.1 modelReference

The modelReference is a reference used to refer to a particular Model via a SIdRef. The modelReference either represents a relation between two Model objects, a Variable object and a Model object, or a relation between a Task object and a Model object.

The `source` attribute of a Model is allowed to reference either a URI or an SId of a second Model. Circular constructs where a model `A` refers to a model `B` and `B` to `A` (directly or indirectly) are invalid.

If pre-processing needs to be applied to a model before simulation, then the model update can be specified by creating a Change object. In the particular case that a change must be calculated with a mathematical function, variables need to be defined. To refer to an existing entity in a defined Model, the modelReference is used.

The `modelReference` attribute of the `variable` element contains the id of a model that is defined in the document.

Listing 2.1 shows the use of the `modelReference` element. In the example, a change is applied on model `m0001`. In the `computeChange` element a list of variables is defined. One of those variable is `v1` which is defined in another model (`cellML`). The XPath expression given in the target attribute identifies the variable in the model which carries the ID `cellML`.

```
<model id="m0001" [..]>
    <listOfChanges>
        <computeChange>
            <listOfVariables>
                <variable id="v1" modelReference="cellML" target="/cellml:model/cellml:component[
                    @cmeta:id='MP']/cellml:variable[@name='vsP']/@initial_value" />
                [..]
            </listOfVariables>
            <listOfParameters [..] />
                <math>
                [CALCULATION OF CHANGE]
                </math>
        </computeChange>
    </listOfChanges>
    [..]
</model>
```

**Listing 2.1:** *SED-ML `modelReference` attribute inside a variable definition of a `computeChange` element*

The modelReference is also used to indicate that a Model object is used in a particular Task. Listing 2.2 shows how this can be done for a sample SED-ML document.

```
<listOfTasks>
    <task id="t1" name="Baseline" modelReference="model1" simulationReference="simulation1" />
    <task id="t2" name="Modified" modelReference="model2" simulationReference="simulation1" />
</listOfTasks>
```

**Listing 2.2:** *SED-ML `modelReference` definition inside a `task` element*

The example defines two different tasks; the first one applies the simulation settings of `simulation1` on `model1`, the second one applies the same simulation settings on `model2`.

##### 2.1.2.2 simulationReference

The simulationReference is used to refer to a particular Simulation via a SIdRef, e.g., in a Task.

Listing 2.2 shows the reference to a defined simulation for a sample SED-ML document. In the example, both tasks `t1` and `t2` use the simulation settings defined in `simulation1` to run the experiment.

##### 2.1.2.3 taskReference

The taskReference is a reference used to refer to a particular *AbstractTask* via a SIdRef. The taskReference is used in SubTask to reference the respective subtask, or in Variable within a DataGenerator.

DataGenerator objects are created to apply post-processing to the simulation results before final output. For certain types of post-processing Variable objects need to be created. These link to a task defined within the ListOfTasks from which the model that contains the variable of interest can be inferred. A taskReference association is used to realise that link from a Variable object inside a DataGenerator to an *AbstractTask* object. Listing 2.3 gives an example.

```
<listOfDataGenerators>
```

```
    <dataGenerator id="tim3" name="tim mRNA (difference v1-v2+20)">
    <listOfVariables>
        <variable id="v1" taskReference="t1" [..] />
    </listOfVariables>
    <math [..]/>
    </dataGenerator>
</listOfDataGenerators>
```

**Listing 2.3:** *SED-ML* `taskReference` *definition inside a* `dataGenerator` *element*

The example shows the definition of a variable `v1` in a `dataGenerator` element. The variable appears in the model that is used in task `t1`. The task definition of `t1` might look as shown in Listing 2.4.

```
<listOfTasks>
    <task id="t1" name="task definition" modelReference="model1" simulationReference="simulation1" />
</listOfTasks>
```

**Listing 2.4:** *Use of the reference relations in a task definition*

Task `t1` references the model `model1`. Therefore we can conclude that the variable `v1` defined in Listing 2.3 targets an element of the model with ID `model1`. The targeting process itself will be explained in section 2.1.8.3 on page 22.

#### 2.1.3 `listOf*` containers

SED-ML `listOf*` elements serve as containers for a collection of objects of the same type. For example, the `listOfModels` contains all Model objects of a SED-ML document. Lists do not carry any further semantics nor do they add additional attributes. They might, however, be annotated with Notes and Annotation as they are derived from SEDBase. All `listOf*` elements are optional in a SED-ML document (with exception of `listOfRanges` and `listOfSubTasks` in a RepeatedTask, which are mandatory).

#### 2.1.4 `SEDBase`

SEDBase is the base class of all SED-ML classes (Figure 2.3). The SEDBase class has the optional attribute `metaid`, and the two optional subelements `notes` and `annotation`.

The optional `notes` and `annotation` subelements provide investigators the ability to attach additional information to all SED-ML objects.

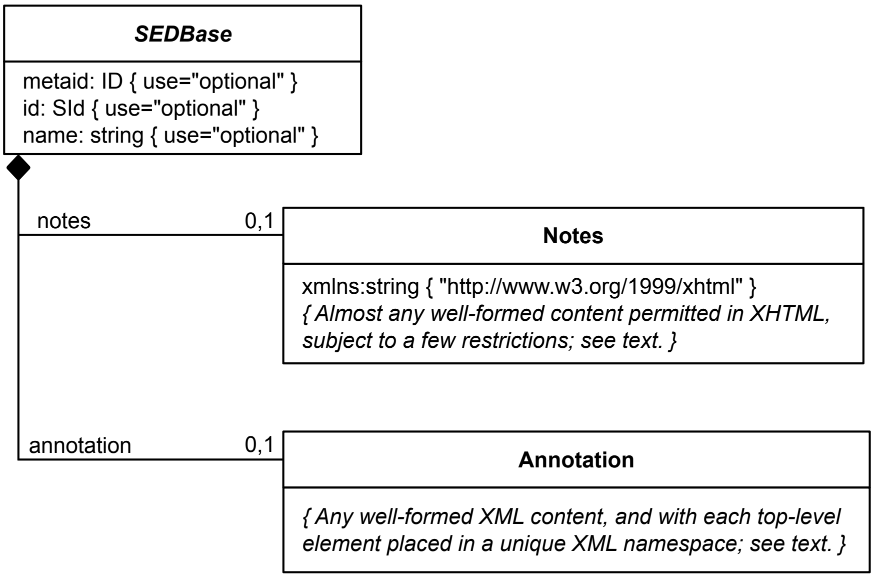

**Figure 2.3:** *The SEDBase, Notes, and Annotation classes*

*id*

The id attribute is an optional attribute on the *SEDBase* class. The id attribute value on an object serves as its *identifier*. The data type of id on *SEDBase* is SId (Section 2.1.1.2). Every SId attribute value in a SED-ML Document must be unique. Whenever a SED-ML element references another SED-ML element, it must use this identifier to do so.

Although id is optional on *SEDBase*, object classes derived from *SEDBase* may stipulate that id is a required attribute for those classes.

In earlier versions of SED-ML, the attributes id and name were defined on individual object subclasses. The movement of these attributes to *SEDBase* in this version has no practical effect on these classes.

An example for an id is given in Listing 2.5. In the example the model has the id m00001.

```
<model id="m00001" language="urn:sedml:language:sbml"
        source="https://www.ebi.ac.uk/biomodels/model/download/BIOMD0000000012?filename=
             BIOMD0000000012_url.xml">
    [MODEL DEFINITION]
</model>
```

**Listing 2.5:** *SED-ML id definition, e.g., for a model*

*name*

The attribute name is an optional attribute on *SEDBase* of type string. In contrast to the id attribute, the name attribute is not intended to be used for cross-referencing purposes within a model. Its purpose instead is to provide a human-readable label for a component. The data type of name is the type string defined in XML Schema [4, 20]. SED-ML imposes no restrictions as to the content of name attributes beyond those restrictions defined by the string type in XML Schema. In addition, name values do not need to be unique.

Listing 2.6 extends the model definition in Listing 2.5 by a model name.

```
<model id="m00001" name="Circadian oscillator" language="urn:sedml:language:sbml"
        source="https://www.ebi.ac.uk/biomodels/model/download/BIOMD0000000012?filename=
             BIOMD0000000012_url.xml">
    [MODEL DEFINITION]
</model>
```

**Listing 2.6:** *SED-ML name definition, e.g., for a model*

*metaid*

The main purpose of the metaid attribute of data type ID is to use the Annotation class to attach semantic annotations to elements of SED-ML documents. The metaid attribute must be globally unique throughout a SED-ML document, i.e., the metaid must be unambiguous throughout a whole SED-ML document.

A metaid is required to apply a Notes or Annotation to a SED-ML element.

*notes*

The optional notes element stores Notes on *SEDBase*.

*annotation*

The optional annotation element stores Annotation on *SEDBase*.

#### 2.1.5 Notes

A Notes can be used to provide a human-readable description of an element of a SED-ML document. Instances of the Notes class may contain any valid XHTML [18]. The namespace URL for XHTML content inside the Notes class is http://www.w3.org/1999/xhtml, which may be declared either in the SedML element, or directly in the top level XHTML elements contained within the notes element. For details on of how to set the namespace and examples see the SBML specification [15].

Table 2.2 on the following page shows all attributes and sub-elements for the Notes element.

Notes does not have any further sub-elements defined in SED-ML, nor attributes associated with it.

| attribute | description |
|---|---|
| xmlns:string "http://www.w3.org/1999/xhtml" | page 28 |
| **sub-elements** | |
| *well-formed content permitted in XHTML* | |

**Table 2.2:** *Attributes and nested elements for Notes. $^{o}$denotes optional elements and attributes.*

Listing 2.7 shows the use of the `notes` element.

```
<sedML [..]>
    <notes>
        <p xmlns="http://www.w3.org/1999/xhtml">The enclosed simulation description shows the oscillating
              behaviour of the Repressilator model using deterministic and stochastic simulators.</p>
    </notes>
</sedML>
```

**Listing 2.7:** *The* `notes` *element*

In this example, the namespace declaration is inside the `notes` element and the note is related to the `sedML` root element of the SED-ML file. A note may, however, occur inside *any* SED-ML XML element, except `note` itself and Annotation.

#### 2.1.6 Annotation

An Annotation can be used to capture computer-processable information about an element of a SED-ML document. Annotations may contain any valid XML content. For further guidelines on how to use annotations see the SBML specification [15]. The recommended style of annotations in SED-ML is briefly described in Section 3.2.4.

Listing 2.8 shows the use of the `annotation` element. In this example, a `model` element is annotated with a reference to the original publication. The `model` contains an `annotation` that uses the model-qualifier `isDescribedBy` to link to the external resource `https://identifiers.org/pubmed/10415827`. In natural language, the annotation content could be interpreted as "The model is described by the published article available from PubMed under the identifier 10415827".

```
<sedML>
    [..]
    <model id="model1" metaid="_001" language="urn:sedml:language:cellml" source="goldbeter1999a.cellml"
         >
        <annotation>
            <rdf:RDF xmlns:rdf="http://www.w3.org/1999/02/22-rdf-syntax-ns#" xmlns:bqmodel="http://
                 biomodels.net/model-qualifiers/">
                <rdf:Description rdf:about="#_001">
                <bqmodel:isDescribedBy>
                <rdf:Bag>
                    <rdf:li rdf:resource="https://identifiers.org/pubmed/10415827"/>
                </rdf:Bag>
                </bqmodel:isDescribedBy>
                </rdf:Description>
            </rdf:RDF>
        </annotation>
    </model>
    [..]
</sedML>
```

**Listing 2.8:** *The annotation element*

#### 2.1.7 Algorithm

The Algorithm class is used as a child of the Simulation, *AbstractTask*, Change, Output, Range, and FitExperiment classes (defined later) as a way to declare uses of those classes that are not explicitly defined in this specification. Instead, the algorithms are defined in the KiSAO ontology, which can be changed and adapted outside of the release cycle of SED-ML specifications. The KiSAO ontology was originally designed to store Simulation algorithms and algorithm parameters, and is expanding into the other types of algorithms as well.

An Algorithm has a mandatory attribute `kisaoID` which contains a KiSAO reference to the particular algorithm used by its parent Simulation, *AbstractTask*, Change, Output, Range, or FitExperiment. In addition, the Algorithm has an optional `listOfAlgorithmParameters` child containing algorithmParameter children, which are used to parameterize the algorithm. If the algorithm in question is a hybrid algorithm, the root Algorithm object's `kisaoID` will reference the hybrid algorithm itself, and may have AlgorithmParameter children indicating which specific algorithms are being combined.

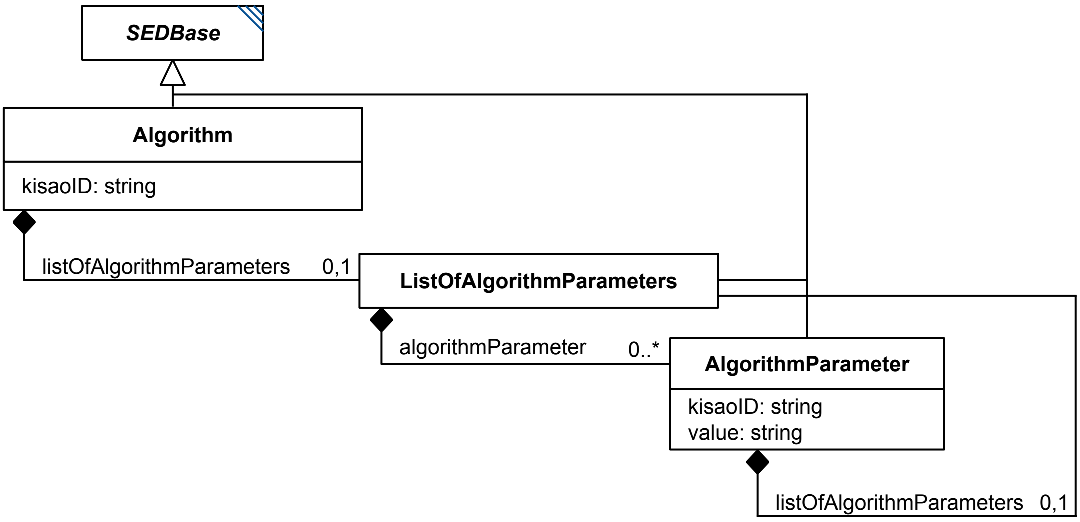

**Figure 2.4:** *The Algorithm, ListOfAlgorithmParameters, and AlgorithmParameter classes.*

The example given in Listing 2.41, completed by algorithm definitions results in the code given in Listing 2.9. In the example, for both simulations an algorithm is defined. In the first simulation `s1` a deterministic approach is used (Euler forward method), in the second simulation `s2` a stochastic approach is used (Stochsim nearest neighbor).

```
<listOfSimulations>
    <uniformTimeCourse id="s1" name="time course simulation over 100 minutes" [..]>
        <algorithm kisaoID="KISAO:0000030" />
    </uniformTimeCourse>
    <uniformTimeCourse id="s2" name="time course definition for concentration of p" [..]>
        <algorithm kisaoID="KISAO:0000021" />
    </uniformTimeCourse>
</listOfSimulations>
```

**Listing 2.9:** *The SED-ML* **`algorithm`** *element for two different time course simulations, defining two different algorithms. KISAO:0000030 refers to the* Euler forward method *; KISAO:0000021 refers to the* StochSim nearest neighbor algorithm.

*`listOfAlgorithmParameters`*

The `listOfAlgorithmParameters` contains the settings for the simulation algorithm used in a simulation (Figure 2.12 on page 40). It may list several instances of the AlgorithmParameter class. The `listOfAlgorithmParameters` is optional and may contain zero to many parameters.

Listing 2.10 shows the use of the `listOfAlgorithmParameters` element.

```
<listOfAlgorithmParameters>
    <algorithmParameter name="absolute tolerance" kisaoID="KISAO:0000211" value="23"/>
</listOfAlgorithmParameters>
```

**Listing 2.10:** *SED-ML listOfAlgorithmParameters element*

##### 2.1.7.1 `AlgorithmParameter`

The AlgorithmParameter class allows to parameterize a particular simulation algorithm. The set of possible parameters for a particular instance is determined by the algorithm that is referenced by the `kisaoID` of the enclosing algorithm element (Figure 2.12 on page 40). Parameters of simulation algorithms are unambiguously referenced by the mandatory `kisaoID` attribute. Their value is set in the mandatory `value` attribute. An AlgorithmParameter may also have child AlgorithmParameter elements through a ListOfAlgorithmParameters.

Any AlgorithmParameter defined in a Simulation overrides any global AlgorithmParameter defined in the SED-ML Document. And in the reverse, any AlgorithmParameter left undefined in a Simulation may be defined by a global AlgorithmParameter element. Any AlgorithmParameter child of a Simulation applies only to that individual Simulation, and assumes its previous value (if set globally) or becomes unset (if not) outside of the context of that Simulation (for example, within a RepeatedTask).

NOTE: the global ListOfAlgorithmParameters was added to SED-ML in Level 1 Version 5. As such, the only place to define a random seed (`KISAO:0000488`) for the simulation experiment as a whole in previous versions was in a Simulation, which might be part of a RepeatedTask. Rather than indicating that each repeat was to receive the same seed, resulting in identical traces, users would generally use the 'seed' parameter to indicate that the experiment as a whole was to be replicable from one run to the next. Current users of SED-ML should use a global AlgorithmParameter for this purpose, but older versions or older files may use the previous scheme.

##### *`value`*

The `value` sets the value of the AlgorithmParameter. For XML purposes, it is of type `string`, but should contain a value that makes sense for the `kisaoID` in question: if the KiSAO term is a value, the string should contain a number; if the KiSAO term is a Boolean, the string should contain the string "`true`" or "`false`", etc. The string must be non-empty; to explicitly state that a value is not set, this should be encoded in the string as a `KISAO:0000629`, which indicates that the value is `Null`.

```
<algorithm kisaoID="KISAO:0000032">
    <listOfAlgorithmParameters>
        <algorithmParameter name="absolute tolerance" kisaoID="KISAO:0000211" value="23"/>
    </listOfAlgorithmParameters>
</algorithm>
```

**Listing 2.11:** *The SED-ML `algorithmParameter` element setting the parameter value for the simulation algorithm. `KISAO:0000032` refers to the* explicit fourth-order Runge-Kutta method*; `KISAO:00000211` refers to the* absolute tolerance.

##### *`listOfAlgorithmParameters`*

The child `listOfAlgorithmParameters` of an AlgorithmParameter may contain parameters that modify or refine the parent parameter, depending on the KiSAO term used.

```
<algorithm name="hybrid method" kisaoID="KISAO:0000352">
    <listOfAlgorithmParameters>
        <algorithmParameter name="modeling and simulation algorithm" kisaoID="KISAO:0000000" value="KISAO:0000019">
            <listOfAlgorithmParameters>
                <algorithmParameter name="absolute tolerance"   kisaoID="KISAO:0000211" value="1e-07"/>
                <algorithmParameter name="integration method"   kisaoID="KISAO:0000475" value="BDF"/>
                <algorithmParameter name="interpolate solution" kisaoID="KISAO:0000481" value="true"/>
                <algorithmParameter name="iteration type"       kisaoID="KISAO:0000476" value="Newton"/>
                <algorithmParameter name="linear solver"        kisaoID="KISAO:0000477" value="Dense"/>
                <algorithmParameter name="lower half-bandwidth" kisaoID="KISAO:0000480" value="0"/>
                <algorithmParameter name="maximum number of steps" kisaoID="KISAO:0000415" value="500"/>
                <algorithmParameter name="maximum step size"    kisaoID="KISAO:0000467" value="0"/>
                <algorithmParameter name="preconditioner"       kisaoID="KISAO:0000478" value="Banded"/>
                <algorithmParameter name="relative tolerance"   kisaoID="KISAO:0000209" value="1e-07"/>
                <algorithmParameter name="upper half-bandwidth" kisaoID="KISAO:0000479" value="0"/>
            </listOfAlgorithmParameters>
        </algorithmParameter>
        <algorithmParameter name="modeling and simulation algorithm" kisaoID="KISAO:0000000" value="KISAO:0000282">
            <listOfAlgorithmParameters>
                <algorithmParameter name="linear solver"        kisaoID="KISAO:0000477" value="Dense"/>
                <algorithmParameter name="lower half-bandwidth" kisaoID="KISAO:0000480" value="0"/>
                <algorithmParameter name="maximum iterations"   kisaoID="KISAO:0000486" value="200"/>
                <algorithmParameter name="upper half-bandwidth" kisaoID="KISAO:0000479" value="0"/>
            </listOfAlgorithmParameters>
        </algorithmParameter>
    </listOfAlgorithmParameters>
</algorithm>
```

**Listing 2.12:** *A SED-ML `algorithmParameter` element defining a mixed simulator, each with their own set of algorithm parameters.*

#### 2.1.8 Calculation

The Calculation class is an abstract base class for the ComputeChange, DataGenerator, and FunctionalRange classes (defined later). A Calculation inherits from *SEDBase*, and adds three children: a required

Math child, and optional lists of Variable and Parameter objects. In all three of its uses, it performs a calculation that optionally may depend on locally-defined elements. This abstract class is provided for convenience, since all three other classes contain this same relatively complicated structure. However, as FunctionalRange also inherits from Range, and ComputeChange also inherits from Change, implementations may choose to simply re-instantiate the child elements of Calculation on these or other derived classes, in environments where multiple inheritance is illegal or infeasible.

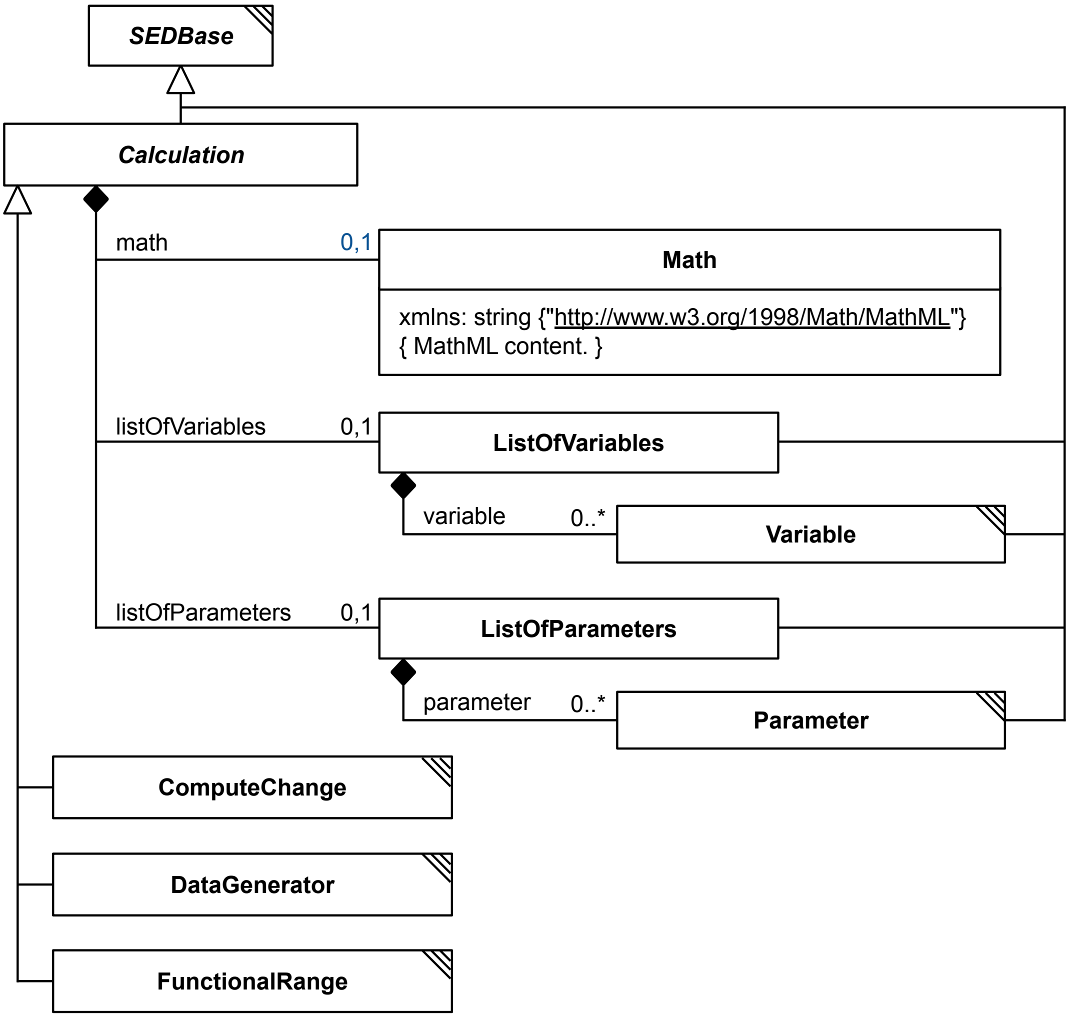

**Figure 2.5:** *The Calculation, Math, ListOfVariables, ListOfParameters, and Parameter classes.*

In the ListOfVariables, the Variable elements define identifiers referring to model variables or range values, which may then be used within the Math expression. These references always retrieve the current value of the variable in the context of the Calculation. A ListOfVariables may contain any number of Variable entries.

In the ListOfParameters, the Parameter elements define simple values that may be used in the Math of the Calculation.

The Math encompasses the mathematical expression that is used to compute the value for the Calculation.

##### 2.1.8.1 Math

A Calculation's optional child element `math` contains a MathML expression used to calculate a value in the context of the Calculation. The available subset of mathematical functions and elements which can be used in the Math element are listed in Section MathML. If there is no child algorithm of the derived class, or if the child algorithm is not defined, then this math child is required.

##### 2.1.8.2 Parameter

The Parameter class (Figure 2.6) can be used to create named parameters with a constant value for use in mathematical expressions. The Parameter class introduces the required attribute `value` of type `double`, and inherits other attributes and children from *SEDBase*, with the exception that the attribute `id` is required instead of optional. The `id` takes on the value of the `value` in the context of the Math of the parent Calculation. Its `id` may not be used in a Calculation that is not its parent, but it must nevertheless be globally unique.

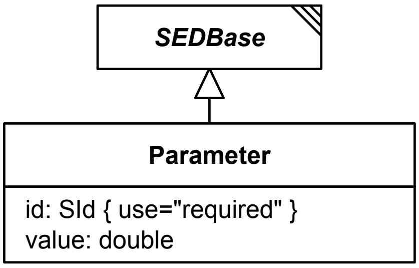

**Figure 2.6:** *The Parameter class*

A Parameter can be used wherever a mathematical expression to compute a value is defined, e.g., in ComputeChange, FunctionalRange or DataGenerator. The Parameter definitions are local to the particular class defining them. Using Parameters rather than including numbers directly within mathematical expressions enables investigators to use notes and annotations to provide additional information about the constants involved in mathematical expressions.

Every Parameter is defined inside a ListOfParameters. The element is optional and may contain zero to many parameters.

Listing 2.13 shows the use of the `parameter` element. This example defines a parameter `p1` with the value `40`.

```
<listOfParameters>
    <parameter id="p1" name="KM" value="40" />
</listOfParameters>
```

**Listing 2.13:** *The definition of a parameter in SED-ML*

*value*

The `value` attribute of data type `double` is required for each Parameter. Each Parameter has exactly one fixed `value`.

##### 2.1.8.3 Variable

A Variable (Figure 2.7 on the following page) is a reference to a mathematical value. The Variable class inherits the attributes and children of *SEDBase*, changing the attribute `id` to be required, and adds several more. A Variable references a single mathematical element (which in some cases may be multidimensional) by using its attributes `target`, `symbol`, `target2`, `symbol2`, and `term`, either together or separately. It may also need to reference a particular model in a particular context by using the `modelReference` and `taskReference` attributes. Finally, it may reduce the dimensionality of the resulting math by using the `dimensionTerm` attribute with one or more AppliedDimension children, as members of its ListOfAppliedDimensions child. All of these additional attributes and child objects are optional, but must together be used to fully define a unique mathematical reference.

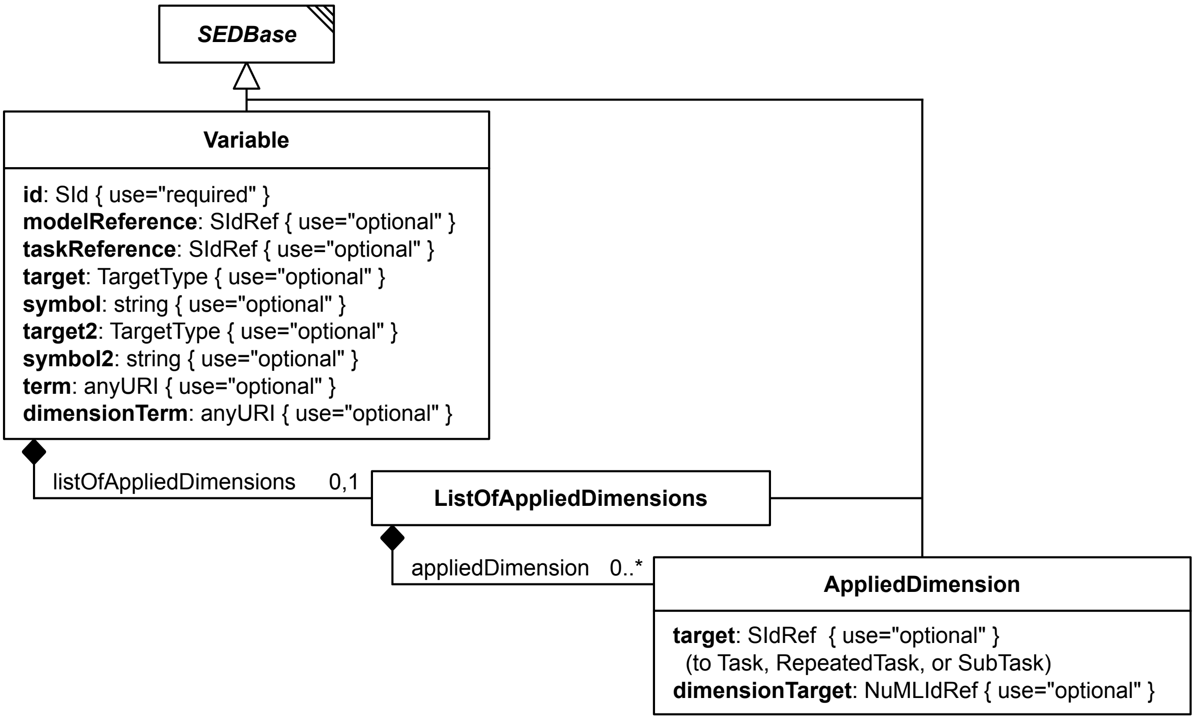

**Figure 2.7:** *The Variable, ListOfAppliedDimensions, and AppliedDimension classes*

A Variable may be used in one of the following ways:

- To reference an explicit element of a model, using the `target` attribute. An SBML Species or a CellML Variable are two examples.
- To reference a DataGenerator or DataSource in the same document, using the `target` attribute. A `target` with the value "`#dataSource1`" is one example.
- To reference an implicit element of a model, using the `symbol` attribute. 'Time' in an SBML model is one example.
- To reference an implicit aspect of an explicit model element, using both the `target` and `symbol` attributes. 'The concentration of an SBML Species' is one example.
- To reference a mathematical concept implicit in the model, using the `term` attribute. 'The Stoichiometry matrix' is one example.
- To reference a mathematical concept implicit in the model for one or two explicit or implicit model elements, using the `term` attribute in combination with one or more of the `target`, `symbol`, `target2`, and `symbol2` attributes. 'The rate of change of species `S1` with respect to time' is one example; 'the elasticity of reaction `J1` with respect to `p0`' is another.
- To reference a mathematical dimension reduction of any of the above, using the `dimensionTerm` in combination with one or more AppliedDimension children, along with other attributes as above that point to any multidimensional construct. 'The average of model variable `P0` over time' is one example; 'the smallest eigenvalue' is another.

In addition, a Variable must either reference data directly (using a `target` that points to a DataSource or DataGenerator), or clearly reference a single model or task/model combination from which the above mathematics is derived. This can be done using the `taskReference` and/or `modelReference` attributes, and depends on the context of the Variable:

- If the `target` of the Variable references a DataSource, neither the `modelReference` nor the `taskReference` may be defined, as external data has neither models nor tasks. Otherwise:

- In a ComputeChange child of a Model, there is no task, and a single `modelReference` is used to reference the initial state of any model, including the parent of the ComputeChange. The `taskReference` must not be defined.

- In a FunctionalRange, the task is taken to be the parent of the FunctionalRange, and the `modelReference` is taken to be the model being changed by the parent *AbstractTask*. Either the `modelReference` or `taskReference` may be defined so as to be explicit. The `modelReference` is required if the parent *AbstractTask* itself references multiple models.

- In a DataGenerator, the task must be defined with the `taskReference` attribute. If the referenced *AbstractTask* uses multiple models, the `modelReference` attribute must also be defined. The `modelReference` attribute may also be defined to be explicit.

- In a SetValue, the `taskReference` is taken to be the parent *AbstractTask*, and the `modelReference` is used to reference the current state of any model. If the model in question is not modified by the *AbstractTask*, that model's initial state is used. The `taskReference` may be set explicitly if desired, and must reference the parent *AbstractTask* if so.

For a Variable child of a DataGenerator, the Variable is by definition multidimensional, taking on the dimensions of the referenced *AbstractTask* in addition to its base definition. In all other cases, the Variable has only the dimensions of its base definition, and is derived from the current or initial state of the referenced Model, as above. In essense, this means that a Variable that points to "`p1`" in a Model can be:

- The scalar value of `p1` in the context of a ComputeChange,

- A vector of `p1` values in the context of a DataGenerator pointing to a timecourse Task, or

- A multidimensional vector of `p1` values in the context of a DataGenerator pointing to a RepeatedTask of timecourses.

Listing 2.14 shows the use of the `variable` element. In the example a variable `v1` is defined to compute a change on a model constituent (referenced by the `target` attribute on `computeChange`). The value of `v1` corresponds to the value of the targeted model constituent referenced by the `target` attribute. The second variable `v2` is used inside a `dataGenerator`. As the variable is time as used in `task1`, the `symbol` attribute is used to refer to the SED-ML URI for time.

```
<sedML>
    <listOfModels>
        <model [..]>
            <listOfChanges>
                <computeChange target="TARGET ELEMENT OR ATTRIBUTE">
                <listOfVariables>
                   <variable id="v1" name="maximum velocity" target="PATH TO MODEL ELEMENT/ATTRIBUTE" />
                   [FURTHER VARIABLE DEFINITIONS]
                </listOfVariables>
                [..]
                </computeChange>
            </listOfChanges>
            [..]
        </model>
        [..]
    </listOfModels>
    <listOfDataGenerators>
        <dataGenerator [..]>
            <listOfVariables>
                <variable id="v2" name="time" taskReference="task1" symbol="KISAO:0000832" />
                [FURTHER VARIABLE DEFINITIONS]
            </listOfVariables>
        </dataGenerator>
    </listOfDataGenerators>
    [..]
</sedML>
```

**Listing 2.14:** *SED-ML `variable` definitions inside the `computeChange` element and inside the `dataGenerator` element*

##### *`target`*

An instance of Variable can refer to a model constituent inside a particular model through the address stored in the `target` attribute, such as an `XPath` expression.

Note that while it is possible to write XPath expressions that select multiple nodes within a referenced model, when used within a `target` attribute, a single element or attribute *must* be selected by the expression.

The `target` attribute may also be used in several situations to reference another SED-ML element with mathematical meaning, by containing a fragment identifier consisting of a hash character (`#`) followed by the SId of the element (i.e. "`#id001`"):

- Any Variable may use a `target` to reference a DataSource. In this situation, the Variable has the mathematical meaning and dimensionality (which may be reduced) of the referenced data.
- A Variable inside a DataGenerator may use a `target` to reference a different DataGenerator. In this situation, the Variable has the mathematical meaning and dimensionality (which may be reduced) of that DataGenerator.
- A Variable inside a RepeatedTask may use a `target` to reference a Range. In this situation, the Variable has the mathematical meaning of the scalar value of the Range for that iteration of the RepeatedTask.

There are no other situations in SED-ML where the `id` of a SED-ML element may be used as the `target` of a Variable. Also note that multidimensional DataSource ids may not be used in RepeatedTask elements, nor Range ids in DataGenerator elements. (To access multidimensional data for a Range, a DataRange may be used instead.)

Listing 2.15 shows the use of the `target` attribute in a SED-ML file. In the example the `target` is used to reference a species with `id='PY'` in an SBML model.

```
<listOfVariables>
    <variable id="v1" name="TetR protein" taskReference="task1"
        target="/sbml:sbml/sbml:listOfSpecies/sbml:species[@id='PY']" />
</listOfVariables>
```

**Listing 2.15:** *SED-ML `target` definition*

It should be noted that the identifiers and names inside the SED-ML document do not have to match the identifiers and names that the model and its constituents have in the model definition. In Listing 2.15, the variable with ID `v1` is defined. It is described as `TetR protein`. The reference points to a species in the referenced SBML model. The particular species can be identified through its ID in the SBML model, namely `PY`. However, SED-ML also permits using identical identifiers and names as in the referenced models. The following Listing 2.16 is another valid example for the specification of a variable, but uses the same naming in the variable definition as in the original model (as opposed to Listing 2.15):

```
<listOfVariables>
    <variable id="PY" name="TetR protein"  taskReference="task1"
        target="/sbml:sbml/sbml:listOfSpecies/sbml:species[@id='PY']" />
</listOfVariables>
```

**Listing 2.16:** *SED-ML variable definition using the original model identifier and name in SED-ML*

```
<sbml [..]>
    <listOfSpecies>
        <species metaid="PY" id="PY" name="TetR protein" [..]>
        [..]
        </species>
    </listOfSpecies>
    [..]
</sbml>
```

**Listing 2.17:** *Species definition in the referenced model*

The XPath expression used in the `target` attribute unambiguously leads to the particular place in the SBML model, i.e., the species is to be found in the *sbml* element, and there inside the *listOfSpecies* (Listing 2.17).

*`symbol`*

The `symbol` attribute of type `string` is used to refer either to a predefined, implicit variable or to a predefined implicit function to be performed on the `target`. In both cases, the `symbol` should be a `kisaoID` (and follow the format of that attribute) that represents that variable's concept. The notion of implicit variables is explained in Section 3.2.3. For backwards compatibility, the old string "`urn:sedml:symbol:time`" is also allowed, though interpreters should interpret "`KISAO:0000832`" as meaning the same thing.

In the case where the `symbol` refers to a function, the function is applied to the `target` of the Variable. If the function reduces the dimensionality of the Variable, at least one AppliedDimension child should be used.

Listing 2.18 shows the use of the `symbol` attribute in a SED-ML file. The example encodes a variable "`t1`" defined to be the SED-ML symbol for `time`. How to use this variable to calculate a change is explained in Section 2.2.5.6.

```
<listOfVariables>
    <variable id="t1" name="time" taskReference="task1" symbol="KISAO:0000832" />
</listOfVariables>
```

**Listing 2.18:** *SED-ML `symbol` definition*

###### *`term`*

The `term` attribute is of type `string`, and should conform to the syntax of a `kisaoID`. The `term` may refer to a function (such as 'rate of change', `KISAO:0000834`) that relates two variables to each other, instead of just one, or it may refer to an analysis (such as 'the eigenvalue matrix', `KISAO:0000813`) that is dependent on the model as a whole and not on individual model elements.

###### *`target2`*

A `target2` attribute has exactly the same constraints and behavior as a `target` attribute, but refers to a second mathematical element, and is always used in conjunction with a `term`.

###### *`symbol2`*

A `symbol2` attribute has exactly the same constraints and behavior as a `symbol` attribute, but refers to a second mathematical element, and is always used in conjunction with a `term`.

```
<listOfVariables>
    <variable id="S1prime" name="S1'"  taskReference="task1"
        target="/sbml:sbml/sbml:listOfSpecies/sbml:species[@id='S1']"
        symbol2="KISAO:0000832"
        dimensionTerm="KISAO:0000834" />
</listOfVariables>
```

**Listing 2.19:** *SED-ML variable definition of 'the rate of change of S1 with respect to time'*

###### *`taskReference`*

The `taskReference` element of data type SIdRef is used to reference a Task via a taskReference. The usage depends on the context the Variable is used in.

###### *`modelReference`*

The `modelReference` element of data type SIdRef is used to reference a Model via a modelReference. The usage depends on the context the Variable is used in.

Together, the `taskReference` and `modelReference` attributes define a model and its context which the other attributes use to pull data from.

###### *`dimensionTerm`*

A `dimensionTerm` attribute has exactly the same constraints as the `term` attribute, but must refer to a KiSAO term that reduces the dimensionality of multidimensional data. Currently, all such KiSAO terms inherit from "`KISAO:0000824`" ('aggregation function') and includes functions such as mean ("`KISAO:-0000825`"), standard deviation ("`KISAO:0000826`"), and maximum ("`KISAO:0000828`").

Together with the AppliedDimension children, the `dimensionTerm` defines how to reduce the dimensionality of the data defined by the other attributes of a Variable.

##### 2.1.8.4 *`AppliedDimension`*

An AppliedDimension object is exclusively used when the `dimensionTerm` of the Variable is defined, and describes which dimension or dimensions that function is applied to. When multiple dimensions are defined, the function is applied over both at once, and not sequentially. For example, a variable derived from a Task inside a RepeatedTask will have the dimensionality of both. If the `dimensionTerm` of the parent Variable is the 'mean' function ("`KISAO:0000825`"), the following options are available:

- The Variable contains a single AppliedDimension child that refers to the RepeatedTask. The resulting data will have the same dimensions as if the Variable referred directly to the Task, but averaged over every repeat of the RepeatedTask. This situation is particularly common when the Task is a stochastic time course simulation, and the RepeatedTask is a simple loop of that Task.
- The Variable contains a single AppliedDimension child that refers to the Task. The resulting data will be a vector with the same number of entries as there were repeats of the RepeatedTask. This situation is particularly helpful when the RepeatedTask is a parameter scan, and the Variable is tracking a model variable that oscillates during the Task. The resulting vector will be the average value of that model variable under each of the different starting conditions.
- The Variable contains two AppliedDimension children, one that refers to the RepeatedTask and one to the Task. The resulting data will be a single value, that has been averaged over both the Task and RepeatedTask. In this case, the function is performed on an element-by-element basis.

The `term` of the parent Variable with one or more AppliedDimension children should always reference a function that reduces the dimensionality of the data (i.e. children of `KISAO:0000824`).

An AppliedDimension inherits the attributes and children of *SEDBase*, and adds the attributes `target` (of type SIdRef), and `dimensionTarget` (of type `NuMLIdRef`), both of which are optional, but one of which must be present.

##### *`target`*

The `target` attribute of an AppliedDimension is used when the applied dimension is a Task or RepeatedTask, which must be implicitly involved in the construction of the dimensionality of the parent Variable.

Possible values for the `target` attribute include:

- The id of a RepeatedTask
- The id of a Task referenced by a RepeatedTask
- The id of a SubTask child of a RepeatedTask

##### *`dimensionTarget`*

The `dimensionTarget` attribute of an AppliedDimension is used when the Variable references an external data set. The `NuMLIdRef` must reference a dimension of the referenced data.

### 2.2 SED-ML Components

This section describes the major components of SED-ML. Each subsection includes UML diagrams for the relevant classes. Example simulation experiments are provided in Appendix A. Complete examples with model files are available at `https://sed-ml.org/`.

#### 2.2.1 `SED-ML` top level element

Each SED-ML Level 1 Version 5 document has a main class called SED-ML which defines the document's structure and content (Figure 2.8 on the next page). It consists of several parts connected to the SED-ML class via `listOf*` constructs:

- DataDescription (for specification of external data),
- Model (for specification of models),
- Simulation (for specification of simulation setups),
- *AbstractTask* (for the linkage of models and simulation setups),
- DataGenerator (for the definition of post-processing),
- Output (for the specification of plots and reports).
- Style (for the specification of plot element styles).
- AlgorithmParameter (for the definition of global algorithm parameters).

A SED-ML document needs to have the SED-ML namespace defined through the mandatory `xmlns` attribute. In addition, the SED-ML `level` and `version` attributes are required.

The root element of each SED-ML XML file is the `sedML` element, encoding `level` and `version` of the file, and setting the necessary namespaces. Nested inside the `sedML` element are the six optional lists serving as containers for the encoded information: `listOfDataDescriptions` for all external data, `listOfModels` for all models, `listOfSimulations` for all simulations, `listOfTasks` for all tasks, `listOfDataGenerators` for all post-processing definitions, `listOfOutputs` for all output definitions, ListOfStyles for all style definitions. and ListOfAlgorithmParameters for parameters that apply to processing this SED-ML file as a whole.

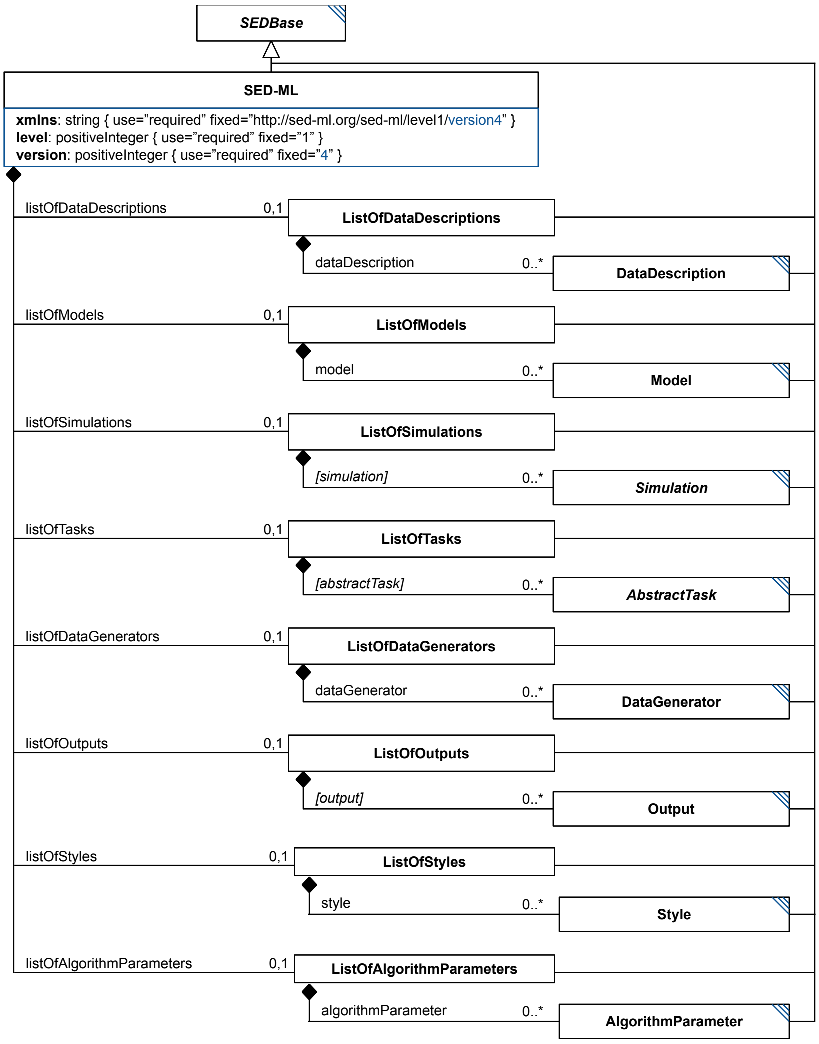

**Figure 2.8:** *The SED-ML class*

The basic XML structure of a SED-ML file is shown in listing 2.20.

```
<?xml version="1.0" encoding="utf-8"?>
<sedML xmlns:math="http://www.w3.org/1998/Math/MathML"
       xmlns="http://sed-ml.org/sed-ml/level1/version4" level="1" version="4">
    <listOfDataDescriptions>
        [DATA REFERENCES AND TRANSFORMATIONS]
    </listOfDataDescriptions>
    <listOfModels>
        [MODEL REFERENCES AND APPLIED CHANGES]
    </listOfModels>
    <listOfSimulations>
        [SIMULATION SETUPS]
    </listOfSimulations>
    <listOfTasks>
        [MODELS LINKED TO SIMULATIONS]
    </listOfTasks>
    <listOfDataGenerators>
        [DEFINITION OF POST-PROCESSING]
    </listOfDataGenerators>
    <listOfOutputs>
        [DEFINITION OF OUTPUT]
    </listOfOutputs>
    <listOfStyles>
        [DEFINITION OF STYLES]
    </listOfStyles>
    <listOfAlgorithmParameters>
        [PARAMETERS TO APPLY TO THE ENTIRE SIMULATION PROCESS]
    </listOfAlgorithmParameters>
</sedML>
```

**Listing 2.20:** *The SED-ML root element*

##### 2.2.1.1 xmlns

The xmlns attribute declares the namespace for the SED-ML document. The pre-defined namespace for SED-ML documents is http://sed-ml.org/sed-ml/level1/version4.

In addition, SED-ML makes use of the MathML namespace http://www.w3.org/1998/Math/MathML to enable the encoding of mathematical expressions. SED-ML notes use the XHTML namespace http://www.w3.org/1999/xhtml. Additional external namespaces might be used in annotations.

##### 2.2.1.2 level

The current SED-ML level is 1. Major revisions containing substantial changes will lead to the definition of forthcoming levels. The level attribute is required and its value is a fixed decimal. For SED-ML Level 1 Version 5 the value is set to 1, as shown in the example in Listing 2.20.

##### 2.2.1.3 version

The current SED-ML version is 4. Minor revisions containing corrections and refinements of SED-ML elements, or new constructs which do not affect backwards compatibility, will lead to the definition of forthcoming versions.

The version attribute is required and its value is a fixed decimal. For SED-ML Level 1 Version 5 the value is set to 4, as shown in the example in Listing 2.20.

##### 2.2.1.4 listOfDataDescriptions

In order to reference data in a simulation experiment, the data files along with a description on how to access such files and what information to extract from them have to be defined. The SED-ML document uses the listOfDataDescriptions container to define DataDescriptions for referencing external data (Figure 2.8 on the previous page). The listOfDataDescriptions is optional and may contain zero or more DataDescriptions.

Listing 2.21 shows the use of the listOfDataDescriptions element.

```
<listOfDataDescriptions>
    <dataDescription id="Data1" name="Oscli Time Course Data" source="./oscli.numl">
        <dimensionDescription>
            <compositeDescription indexType="double" id="time" name="time" xmlns="http://www.numl.org/
                 numl/level1/version1">
                <compositeDescription indexType="string" id="SpeciesIds" name="SpeciesIds">
                    <atomicDescription valueType="double" name="Concentrations" />
                </compositeDescription>
            </compositeDescription>
        </dimensionDescription>
        <listOfDataSources>
            <dataSource id="dataS1">
                <listOfSlices>
                    <slice reference="SpeciesIds" value="S1" />
                </listOfSlices>
            </dataSource>
            <dataSource id="dataTime" indexSet="time" />
        </listOfDataSources>
    </dataDescription>
</listOfDataDescriptions>
```

**Listing 2.21:** *SED-ML listOfDataDescriptions element*

##### 2.2.1.5 listOfModels

The models used in a simulation experiment are defined in the listOfModels container (Figure 2.8 on the previous page). The listOfModels is optional and may contain zero or more Models. However, if a SED-ML document contains one or more Tasks, at least one Model must be defined to which the Task elements refer (see Section 2.1.2.1).

Listing 2.22 shows the use of the listOfModels element.

```
<listOfModels>
    <model id="m0001" language="urn:sedml:language:sbml"
        source="https://www.ebi.ac.uk/biomodels/model/download/BIOMD0000000012?filename=
             BIOMD0000000012_url.xml" />
    <model id="m0002" language="urn:sedml:language:cellml"
        source="https://models.cellml.org/workspace/leloup_gonze_goldbeter_1999/rawfile/
             bfaac0e80b23726ffe05b02f98b3d1d01a2ee3b7/leloup_gonze_goldbeter_1999_a.cellml" />
```

```
</listOfModels>
```

**Listing 2.22:** *SED-ML listOfModels element*

##### 2.2.1.6 listOfSimulations

The `listOfSimulations` element is the container for Simulation descriptions (Figure 2.8 on page 27). The `listOfSimulations` is optional and may contain zero or more Simulations. However, if the SED-ML document contains one or more Tasks, at least one Simulation element must be defined to which the Task elements refer (see Section 2.1.2.2).

Listing 2.23 shows the use of the `listOfSimulation` element.

```
<listOfSimulations>
    <simulation id="s1" [..]>
        [UNIFORM TIMECOURSE DEFINITION]
    </simulation>
    <simulation id="s2" [..]>
        [UNIFORM TIMECOURSE DEFINITION]
    </simulation>
</listOfSimulations>
```

**Listing 2.23:** *The SED-ML* `listOfSimulations` *element, containing two simulation setups*

##### 2.2.1.7 listOfTasks

The `listOfTasks` element contains the defined tasks for the simulation experiment (Figure 2.8 on page 27). The `listOfTasks` is optional and may contain zero or more tasks, each of which is an instance of a subclass of *AbstractTask*.

Each top-level task is defined such that its execution is independent of the others: if one task is executed after another, the states of the models must be completely reset so there's no cross-contamination of one task to the next. This means that the top-level tasks are particularly well suited to being executed in parallel, should that be desired.

SED-ML interpreters may choose to execute all the tasks in the list, or they may choose to examine the list of outputs, and only execute the tasks that are necessary to produce the requested output. This situation comes up most often when one task is listed as a SubTask of a RepeatedTask: the outputs may well only require the RepeatedTask to be run, meaning an independent execution of the singular Task is not necessary, even though it's on this list.

Listing 2.24 shows the use of the `listOfTasks` element.

```
<listOfTasks>
    <task id="t1" name="simulating v1" modelReference="m1" simulationReference="s1">
    [FURTHER TASK DEFINITIONS]
</listOfTasks>
```

**Listing 2.24:** *The SED-ML* `listOfTasks` *element, defining one task*

##### 2.2.1.8 listOfDataGenerators

The `listOfDataGenerators` container holds the dataGenerator definitions of a simulation experiment (Figure 2.8 on page 27). The `listOfDataGenerators` is optional and in general may contain zero or more DataGenerators.

In SED-ML, all variable and parameter values used in the Output class need to be defined as a DataGenerator beforehand.

Listing 2.25 shows the use of the `listOfDataGenerators` element.

```
<listOfDataGenerators>
    <dataGenerator id="d1" name="time">
        [DATA GENERATOR DEFINITION FOLLOWING]
    </dataGenerator>
    <dataGenerator id="LaCI" name="LaCI repressor">
        [DATA GENERATOR DEFINITION FOLLOWING]
    </dataGenerator>
</listOfDataGenerators>
```

**Listing 2.25:** *The* `listOfDataGenerators` *element, defining two data generators* time *and* LaCI repressor

##### 2.2.1.9 listOfOutputs

The listOfOutputs container holds the Output definitions of a simulation experiment (Figure 2.8 on page 27). The listOfOutputs is optional and may contain zero or more outputs.

Listing 2.26 shows the use of the listOfOutputs element.

```
<listOfOutputs>
    <report id="report1">
        [REPORT DEFINITION FOLLOWING]
    </report>
    <plot2D id="plot1">
        [2D PLOT DEFINITION FOLLOWING]
    </plot2D>
</listOfOutputs>
```

**Listing 2.26:** *The listOfOutput element*

##### 2.2.1.10 listOfStyles

The listOfStyles container holds the Style definitions of a simulation experiment (Figure 2.8 on page 27). The listOfStyles is optional and may contain zero or more styles.

Listing 2.27 shows the use of the listOfStyles element.

```
<listOfStyles>
    <style id="redline">
        [STYLE DEFINITION FOLLOWING]
    </style>
    <plot2D id="redline_bluesquares" baseStyle="redline">
        [STYLE DEFINITION FOLLOWING]
    </plot2D>
</listOfStyles>
```

**Listing 2.27:** *The listOfStyles element*

##### 2.2.1.11 listOfAlgorithmParameters (global)

The listOfAlgorithmParameters container holds the AlgorithmParameter objects that apply globally. This can include parameters like a seed (KISAO:0000488) that apply to the simulation experiment as a whole, as well as algorithm parameters that might apply to all tasks of a particular type, such as the absolute tolerance (KISAO:0000211). If an AlgorithmParameter is defined for a particular Simulation, it will take precedent over any global AlgorithmParameter with the same KiSAO ID. The listOfAlgorithmParameters is optional and may contain zero or more parameters.

```
<listOfAlgorithmParameters>
    <algorithmParameter name="absolute tolerance" kisaoID="KISAO:0000211" value="23"/>
    <algorithmParameter name="seed" kisaoID="KISAO:0000488" value="1001"/>
</listOfAlgorithmParameters>
```

**Listing 2.28:** *The global listOfAlgorithParameters element*

#### 2.2.2 DataDescription

The DataDescription class (Figure 2.9 on the next page) allows to reference external data, and contains a description on how to access the data, in what format it is, and what subset of data to extract.

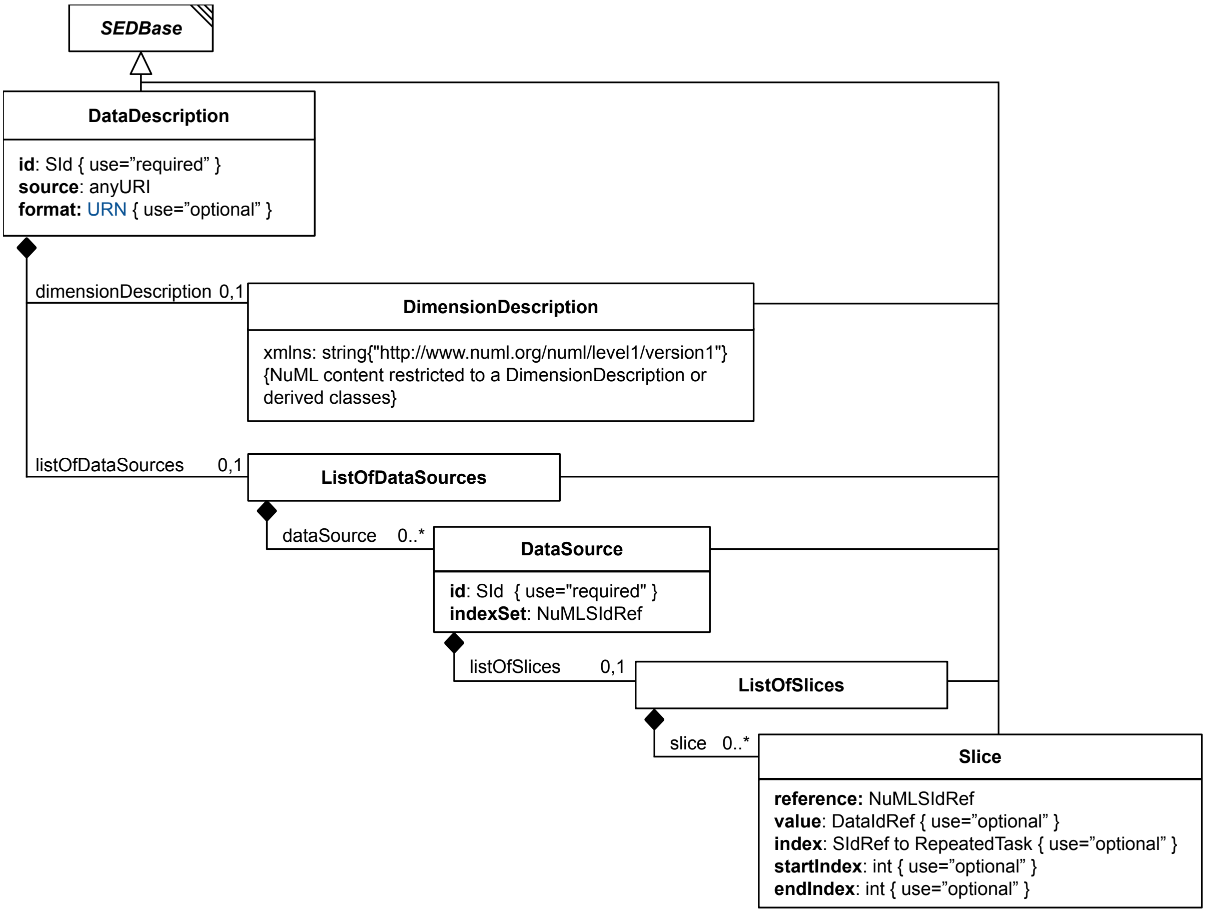

**Figure 2.9:** *The SED-ML DataDescription class*

The DataDescription class introduces four attributes: the required attributes `id` and `source` and the optional attributes `format` and `name`. In addition two optional elements are defined: `dimensionDescription` and `listOfDataSources`.

Listing 2.29 shows the use of the `dataDescription` element.

```
<dataDescription id="Data1" name="Oscli Time Course Data" format="urn:sedml:format:numl"
    source="https://svn.code.sf.net/p/libsedml/code/trunk/Samples/data/oscli.numl" >
    [...]
</dataDescription>
```

**Listing 2.29:** *SED-ML `dataDescription` element*

#### *source*

The required `source` attribute of data type `anyURI` is used to specify the data file. The `source` attribute provides a location of a data file, analog to how the `source` attribute on the Model is handled. In order to resolve the `source` attribute, the same mechanisms are allowed as for the Model `source` element, i.e., via the local file system, a relative link, or an online resource.

#### *format*

The optional `format` attribute of data type `URN` is used to specify the format of the DataDescription. The allowed formats are defined in the format references, e.g., NuML (`urn:sedml:format:numl`) or CSV (`urn:sedml:format:csv`). If it is not explicitly defined the default value for `format` is `urn:sedml:format:numl`, referring to NuML representation of the data.

*dimensionDescription*

The optional **dimensionDescription** contains a DimensionDescription providing the dimension description of the data file. If the format is NuML (urn:sedml:format:numl) and a **dimensionDescription** is set, then the **dimensionDescription** must be identical to the **dimensionDescription** of the NuML file. If the format is not NuML, the **dimensionDescription** is required.

*listOfDataSources*

The optional **listOfDataSources** contains zero or more DataSource elements. A DataSource extracts chunks out of the external data provided by the outer DataDescription element.

#### 2.2.3 DataDescription components

##### 2.2.3.1 *DimensionDescription*

The DimensionDescription class (Figure 2.9 on the previous page) defines the dimensions and data types of the external data provided by the outer DataDescription element. The DimensionDescription is a NuML container containing the dimension description of the dataset.

In the following example a nested NuML **compositeDescription** with **time** spanning one dimension and **SpeciesIds** spanning a second dimension is given. This two dimensional space is then filled with **double** values representing concentrations.

```
<dimensionDescription>
    <compositeDescription indexType="double" id="time" name="time"
        xmlns="http://www.numl.org/numl/level1/version1">
        <compositeDescription indexType="string" id="SpeciesIds" name="SpeciesIds">
            <atomicDescription valueType="double" id="Concentration" name="Concentration" />
        </compositeDescription>
    </compositeDescription>
</dimensionDescription>
```

**Listing 2.30:** *SED-ML dimensionDescription element*

##### 2.2.3.2 *DataSource*

The DataSource class (Figure 2.9 on the preceding page) extracts chunks out of the dataset provided by the outer DataDescription element. The DataSource class introduces three attributes: the required attribute id and the optional attributes name, indexSet, and listOfSlices (Figure 2.9 on the previous page).

DataSource elements can be used anywhere in the SED-ML Description. Specifically their id attribute can be referenced as the **target** of any Variable, pre-pended by a '#' inside DataGenerator, ComputeChange or SetValue objects if the referenced data is a scalar, and as the **target** of a Variable in any DataGenerator even if the referenced data is multidimensional.

The **id** may also be used as the **sourceReference** of a DataRange, where the referenced data may be multidimensional, and as the **dataSource** or **pointWeight** of a FitMapping, where the referenced data must be one dimensional.

Here an example that references the DataSource **dataS1**:

```
<listOfDataDescriptions>
  <dataDescription id="data1" name="data file" source="./example.numl" format="urn:sedml:format:numl">
    <dimensionDescription>
      <compositeDescription indexType="double" name="Time">
        <compositeDescription indexType="string" name="SpeciesIds">
          <atomicDescription valueType="double" name="Values" />
        </compositeDescription>
      </compositeDescription>
    </dimensionDescription>
    <listOfDataSources>
      <dataSource id="dataS1">
        <listOfSlices>
          <slice reference="SpeciesIds" value="S1" />
        </listOfSlices>
      </dataSource>
      <dataSource id="dataTime" indexSet="Time" />
    </listOfDataSources>
  </dataDescription>
</listOfDataDescriptions>
<listOfDataGenerators>
  <dataGenerator id="dgDataS1" name="S1 (data)">
```

```
    <listOfVariables>
      <variable id="varS1" modelReference="model1" target="#dataS1" />
    </listOfVariables>
    <math xmlns="http://www.w3.org/1998/Math/MathML">
      <ci> varS1 </ci>
    </math>
  </dataGenerator>
  ...
</listOfDataGenerators>
```

This represents a change from Level 1 Version 1 and Level 1 Version 2, in which a `taskReference` was always present for a `variable` in a DataGenerator.

To indicate that the `target` of the Variable is an entity defined within the current SED-ML description (and not an entity in an external document, such as referenced by a XPath expression) the hashtag (#) with the reference to an `id` is used.

In addition, this example uses the `modelReference`, in order to facilitate a mapping of the data with a given model.

Data may contain NA values. All calculations containing a NA value have NA as a result.

Since data elements defined via the DimensionDescription of the DataDescription or within the NuML file are either values or indices, the DataSource element provides two ways of addressing those elements, the `indexSet` and `listOfSlices`.

*indexSet*

The `indexSet` attribute allows to address all indices provided by NuML elements with `indexType`.

For example for the `indexSet` `time` below, a dataSource extracts the set of all timepoints stored in the index.

```
<dataSource id="dataTime" indexSet="time" />
```

Similarly

```
<dataSource id="allIds" indexSet="SpeciesIds" />
```

extracts all the species id strings stored in that index set. Valid values for `indexSet` are all NuML Id elements declared in the `dimensionDescription`.

If the `indexSet` attribute is specified the corresponding `dataSource` may not define any `slice` elements.

*listOfSlices*

The `listOfSlices` contains one or more Slice elements. The `listOfSlices` container holds the Slice definitions of a DataSource (Figure 2.9 on page 31). The `listOfSlices` is optional and may contain zero to many Slices.

##### 2.2.3.3 Slice

If a DataSource does not define the `indexSet` attribute, it will contain Slice elements. Each slice removes one dimension from the data hypercube.

The Slice class introduces a required `reference` attribute of type `NuMLSIdRef`, and four optional attributes: `value` of type `DataIdRef`, `index` of type SIdRef, and `startIndex` and `endIndex`, both of type `int` (Figure 2.9 on page 31).

#### 2.2.4 `Model`

The Model class defines the models used in a simulation experiment (Figure 2.10).

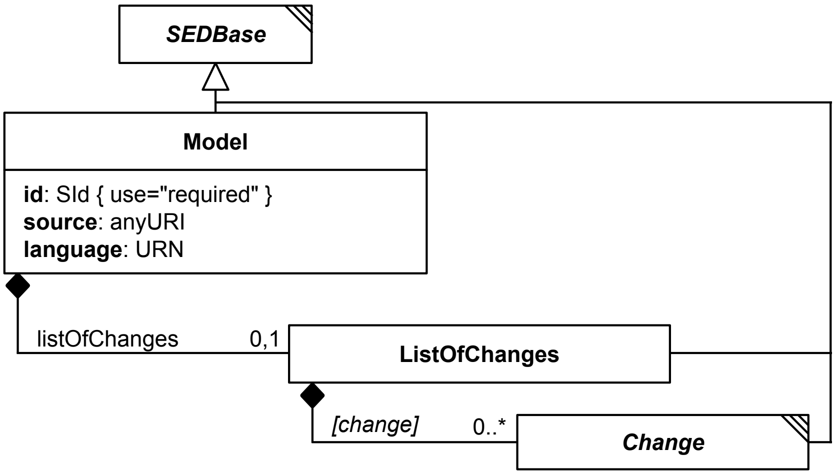

**Figure 2.10:** *The SED-ML Model class*

Each instance of the Model class has the required attributes `id`, `source`, and `language`, the optional attribute `name`, and the optional child `listOfChanges`.

The `language` attribute defines the format the model is encoded in.

The Model class refers to the particular model of interest through the `source` attribute. The restrictions on the model reference are

- The model must be encoded in a well-defined format.
- To refer to the model encoding language, a reference to a valid definition of that format must be given (`language` attribute).
- To refer to a particular model in an external resource, an unambiguous reference must be given (`source` attribute).

A model might need to undergo pre-processing before simulation. Those pre-processing steps are specified in the `listOfChanges` via the Change class.

Listing 2.31 shows the use of the `model` element. In the example the `listOfModels` contains three models: The first model `m0001` is the Repressilator model from BioModels Database available from `https://www.ebi.ac.uk/biomodels/model/download/BIOMD0000000012?filename=BIOMD0000000012_url.xml`. For the SED-ML simulation the model might undergo preprocessing steps described in the `listOfChanges`. Based on the description of the first model `m0001`, the second model `m00002` is built, which is a modified version of the Repressilator model. `m0002` refers to the model `m001` in its `source` attribute. `m0002` might then have additional changes applied to it on top of the changes defined in the pre-processing of `m0001`. The third model in the code example is a model in CellML representation. The model `m0003` is available from the given URL in the `source` attribute. Again, it might have pre-processing steps applied before used in a simulation.

```
<listOfModels>
    <model id="m0001" language="urn:sedml:language:sbml"
        source="https://www.ebi.ac.uk/biomodels/model/download/BIOMD0000000012?filename=
             BIOMD0000000012_url.xml">
        <listOfChanges>
            <change>
                [MODEL PRE-PROCESSING]
            </change>
        </listOfChanges>
    </model>
    <model id="m0002" language="urn:sedml:language:sbml" source="#m0001">
        <listOfChanges>
            [MODEL PRE-PROCESSING]
        </listOfChange>
    </model>
    <model id="m0003" language="urn:sedml:language:cellml" source="https://models.cellml.org/workspace/
         leloup_gonze_goldbeter_1999/rawfile/bfaac0e80b23726ffe05b02f98b3d1d01a2ee3b7/
         leloup_gonze_goldbeter_1999_a.cellml">
        [MODEL PRE-PROCESSING]
    </model>
</listOfModels>
```

**Listing 2.31:** *SED-ML* `model` *element*

#### `language`

The required `language` attribute of data type URN is used to specify the format of the model. Example formats are SBML (`urn:sedml:language:sbml`) or CellML (`urn:sedml:language:cellml`). The supported languages are defined in the language references.

The use of the `language` attribute is required for two reasons. Firstly, it helps to decide whether or not one is able to run the simulation, that is to parse the model referenced in the SED-ML file. Secondly, the language attribute is also needed to decide how to handle the Symbols in the Variable class, as the interpretation of Symbols depends on the language of the representation format.

#### `source`

To make a model accessible for the execution of a SED-ML file, the `source` must be specified through either an URI or a reference to an `SId` of an existing Model. The URI should follow the proposed URI Scheme for Model references.

There are three typical ways to identify a model with the `source` attribute: by relative path, by identifier, or by URL.

An example for the definition of a model via a relative path URI is given in Listing 2.32. The example defines one model `m1` with the model `source` available from "`oscillator.xml`" in the same directory or location as the SED-ML file. A `source` value of "`./oscillator.xml`" would accomplish the same thing more explicitly, with "`./`" being shorthand for 'the current directory'.

```
<model id="m1" name="repressilator" language="urn:sedml:language:sbml"
    source="oscillator.xml">
    <listOfChanges>
        [MODEL PRE-PROCESSING]
    </listOfChanges>
</model>
```

**Listing 2.32:** *The SED-ML* `source` *element, using the URI scheme*

An example for the definition of a model using an URL is given in Listing 2.33. In the example one model is defined. The `language` of the model is `CellML`. The *URL* pointing to the model is used in the

`source` attribute.

```
<model id="m1" name="repressilator" language="urn:sedml:language:cellml"
    source="https://models.cellml.org/exposure/bba4e39f2c7ba8af51fd045463e7bdd3/aguda_b_1999.cellml">
    <listOfChanges />
</model>
```

**Listing 2.33:** *The SED-ML* `source` *element, using a URL*

MIRIAM URNs are no longer recommended due to increased difficulty in resolving them, but the scheme is still valid and interpreters may find SED-ML files that use them. An example for the definition of a model via an URI is given in Listing 2.34. The example defines one model `m1` with the model `source` available from `urn:miriam:biomodels.db:BIOMD0000000012`. The MIRIAM URN can be resolved into the SBML model stored in BioModels Database under the identifier `BIOMD0000000012` by querying the Biomodels webservice and requesting the 'main' SBML file for that biomodel. The resulting URL is `https://www.ebi.ac.uk/biomodels/model/download/BIOMD0000000012?filename=BIOMD0000000012_url.xml`.

```
<model id="m1" name="repressilator" language="urn:sedml:language:sbml"
    source="urn:miriam:biomodels.db:BIOMD0000000012">
    <listOfChanges>
        [MODEL PRE-PROCESSING]
    </listOfChanges>
</model>
```

**Listing 2.34:** *The SED-ML* `source` *element, using the URI scheme*

##### *listOfChanges*

The `listOfChanges` (Figure 2.10 on page 34) contains the Changes to be applied to a particular Model. The `listOfChanges` is optional and may contain zero to many Changes.

Listing 2.35 shows the use of the `listOfChanges` element.

```
<model id="m0001" [..]>
    <listOfChanges>
        [CHANGE DEFINITION]
    </listOfChanges>
</model>
```

**Listing 2.35:** *The SED-ML* `listOfChanges` *element, defining a change on a model*

#### 2.2.5 Change

The Change class allows to describe changes applied to a model before simulation (Figure 2.11 on the following page). Changes can be of the following types:

- Changes based on mathematical calculations (ComputeChange)
- Changes on attributes of the model (ChangeAttribute)
- For XML-encoded models, changes on any XML snippet of the model's XML representation (AddXML, ChangeXML, RemoveXML)

The Change class is abstract and serves as the base class for different types of changes, the ChangeAttribute, AddXML, ChangeXML, RemoveXML, and ComputeChange.

The Change class has the mandatory attribute target which defines the target of the change. The `target` attribute holds an unambiguous description of the address of the element, elements, attribute, or attributes that are to undergo the defined changes, such as a valid XPath expression pointing to the specified XML. For NewXML, AddXML, ChangeXML, and RemoveXML, target must be an XPath expression. This XPath expression must always select an appropriate target for the particular Change used.

##### *algorithm*

The optional child `algorithm` element may define the algorithm used for the execution of the Change. The algorithm is defined via the Algorithm class. For changes that don't easily fit into the above categories, the ComputeChange class should be used in conjunction with the child Algorithm to use KiSAO to define the change in question.

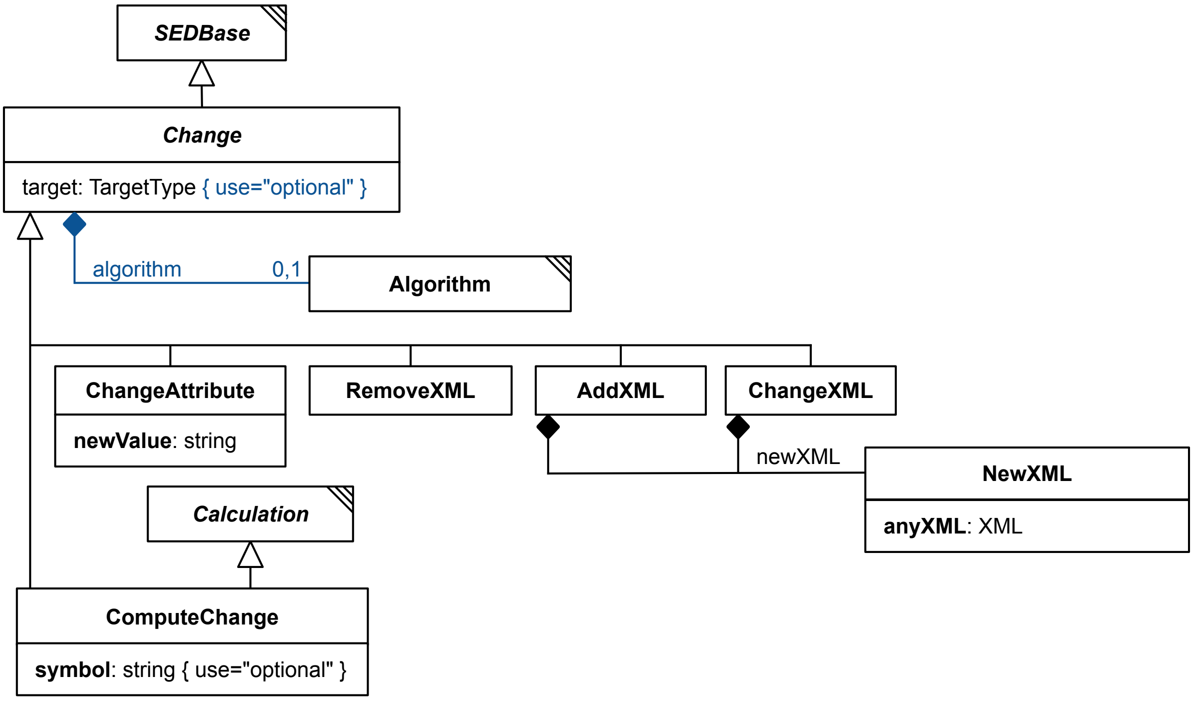

**Figure 2.11:** *The SED-ML Change class*

`target`

The optional `target` attribute holds an unambiguous description of the address of an element or attribute of a model that is to undergo the defined changes. For XML model languages such as SBML, `target` must be a valid XPath expression of data type `xpath` pointing to the XML that is to undergo the defined changes. If the Change has no algorithm child defined, the target is required.

##### 2.2.5.1 NewXML

The `newXML` element provides a piece of XML code (Figure 2.11). `NewXML` must hold a valid piece of XML in the appropriate namespace which after insertion into the original model must result in a valid model file (according to the model language specification as given by the `language` attribute of the model).

The `newXML` element is used at two different places inside SED-ML Level 1 Version 5:

1. If it is used as a sub-element of the `addXML` element, then the XML it contains *is inserted as a child* of the XML element addressed by the XPath.
2. If it is used as a sub-element of the `changeXML` element, then the XML it contains *replaces* the XML element or elements addressed by the XPath.

Examples are given in the relevant change class definitions.

##### 2.2.5.2 AddXML

The AddXML class specifies a snippet of XML that is added as a child of the element selected by the XPath expression in the `target` attribute (Figure 2.11). The new piece of XML code is provided by the NewXML class, and may contain one or more XML elements.

An example for a change that adds an additional parameter to a model is given in Listing 2.36. In the example the model is changed so that a parameter with ID `V_mT` is added to its list of parameters. The `newXML` element adds an additional XML element to the original model. The element's name is `parameter` and it is added to the existing parent element `listOfParameters` that is addressed by the XPath expression in the `target` attribute.

```
<model language="urn:sedml:language:sbml" [..]>
    <listOfChanges>
        <addXML target="/sbml:sbml/sbml:model/sbml:listOfParameters" >
```

```
            <newXML>
                <sbml:parameter xmlns:sbml="http://www.sbml.org/sbml/level3/version1/core"
                                metaid="metaid_0000010" id="V_mT" value="0.7" />
            </newXML>
        </addXML>
    </listOfChanges>
</model>
```

**Listing 2.36:** *The* `addXML` *element with its* `newXML` *sub-element*

#### 2.2.5.3 ChangeXML

The ChangeXML class allows you to replace any XML element(s) in the model that can be addressed by a valid XPath expression (Figure 2.11 on the previous page).

The XPath expression is specified in the required `target` attribute, and may target one or more XML elements. The replacement XML content is specified in the NewXML class, and may also contain one or more XML elements.

An example for a change that adds an additional parameter to a model is given in Listing 2.37. In the example the model is changed in the way that its parameter with ID `V_mT` is substituted by two other parameters `V_mT_1` and `V_mT_2`. The `target` attribute defines that the parameter with ID `V_mT` is to be changed. The `newXML` element then specifies the XML that is to be exchanged for that parameter.

```
<model [..]>
    <listOfChanges>
        <changeXML target="/sbml:sbml/sbml:model/sbml:listOfParameters/sbml:parameter[@id='V_mT']" >
            <newXML>
                <sbml:parameter xmlns:sbml="http://www.sbml.org/sbml/level3/version1/core"
                                metaid="metaid_0000010" id="V_mT_1" value="0.7" />
                <sbml:parameter xmlns:sbml="http://www.sbml.org/sbml/level3/version1/core"
                                metaid="metaid_0000050" id="V_mT_2" value="4.6"> />
            </newXML>
        </changeXML>
    </listOfChanges>
</model>
```

**Listing 2.37:** *The* `changeXML` *element*

#### 2.2.5.4 RemoveXML

The RemoveXML class can be used to delete one or more XML elements or attributes in the model that are addressed by the XPath expression (Figure 2.11 on the preceding page). The XPath is specified in the required `target` attribute.

An example for the removal of an XML element from a model is given in Listing 2.38. In the example the model is changed by deleting the reaction with ID `V_mT` from the model's list of reactions.

```
<model [..]>
    <listOfChanges>
        <removeXML target="/sbml:sbml/sbml:model/sbml:listOfReactions/sbml:reaction[@id='J1']" />
    </listOfChanges>
</model>
```

**Listing 2.38:** *The* `removeXML` *element*

#### 2.2.5.5 ChangeAttribute

The ChangeAttribute class allows to define updates on the attribute values of the corresponding model (Figure 2.11 on the previous page). ChangeAttribute requires to specify the `target` of the change, i.e., the location of the addressed attribute, and also the `newValue` of that attribute. Note that the `target` must select a single attribute within the corresponding model.

Despite its name, the 'attribute' changed by this class need not be an XML attribute, and hence, its `target` need not be an XPath. Every target model language may define what 'attributes' may be changed by this contruct, and how to indicate those attributes.

The ChangeXML class covers the possibilities provided by the ChangeAttribute class, i.e, everything that can be expressed by a ChangeAttribute construct can also be expressed by ChangeXML. However, for the common case of changing an attribute value ChangeAttribute is easier to use, and so it is recommended to use the ChangeAttribute for any changes of an attribute's value, and to use the more general ChangeXML for other cases.

##### *newValue*

The mandatory **newValue** attribute of data type `string` assignes a new value to the targeted attribute.

The example in Listing 2.39 shows the update of the value of two parameters inside an SBML model.

```
<model id="model1" name="Circadian Chaos" language="urn:sedml:language:sbml"
    source="https://www.ebi.ac.uk/biomodels/model/download/BIOMD0000000012?filename=BIOMD0000000012_url.
        xml">
    <listOfChanges>
        <changeAttribute target="/sbml:sbml/sbml:model/sbml:listOfParameters/sbml:parameter[@id='V_mT']/
            @value" newValue="0.28"/>
        <changeAttribute target="/sbml:sbml/sbml:model/sbml:listOfParameters/sbml:parameter[@id='V_dT']/
            @value" newValue="4.8"/>
    </listOfChanges>
</model>
```

**Listing 2.39:** *The* `changeAttribute` *element and its* `newValue` *attribute*

#### 2.2.5.6 *ComputeChange*

The ComputeChange class permits to change, prior to the experiment, the numerical value of any single element or attribute of a Model addressable by a `target`, based on a calculation (Figure 2.11 on page 37). It inherits the `target` attribute from the Change abstract base class, as well as the standard *SEDBase* attributes and children, and adds the optional attribute `symbol` (of type string). Its ability to perform a calculation is described in the Calculation class. (For implementations, if multiple inheritance is not possible, the children of Calculation should just be added directly to the ComputeChange class itself.)

The change is calculated from the Math of the Calculation, and applied to the `target` of the Change. In this context, a `target` that points to an XML element (either the `target` of the ComputeChange or a `target` of a child Variable) is referencing that element's mathematical meaning. For some model languages (such as SBML), this means that the model state must be initialized, so the element value can be read (in the case of a Variable) or changed (in the case of a ComputeChange).

In contrast, a `target` that points to an XML attribute simply is referencing that attribute's value, which may be read or set directly in the XML document without initializing the whole model.

Note also that when a ComputeChange refers to another model, that model is not allowed to be modified by ComputeChanges which directly or indirectly refer to this model, nor to the target of this ComputeChange. In other words, cycles in the definitions of computed changes are prohibited. This does mean that other models may also need to be initialized (and changes applied) in order to apply the changes to this model.

##### *symbol*

The optional `symbol` attribute of data type `string` may be used in addition to the `target` when the particular value associated with the `target` may be described in multiple ways. In particular, a species whose value could be expressed either as a concentration or an amount may be set by using the `target` to point to the species, and setting the `symbol` to “`KISAO:0000838`” to set the concentration, or setting the `symbol` to “`KISAO:0000836`” to set the amount.

Listing 2.40 shows the use of the `computeChange` element.

```
<model [..]>
    <listOfChanges>
    <computeChange target="/sbml:sbml/sbml:model/sbml:listOfParameters/sbml:parameter[@id='sensor']">
        <listOfVariables>
            <variable modelReference="model1" id="R" name="regulator"
                target="/sbml:sbml/sbml:model/sbml:listOfSpecies/sbml:species[@id='regulator']" />
            <variable modelReference="model2" id="S" name="sensor"
                target="/sbml:sbml/sbml:model/sbml:listOfParameters/sbml:parameter[@id='sensor']" />
        <listOfVariables/>
        <listOfParameters>
            <parameter id="n" name="cooperativity" value="2">
            <parameter id="K" name="sensitivity" value="1e-6">
        <listOfParameters/>
        <math  xmlns="http://www.w3.org/1998/Math/MathML">
        <apply>
          <times />
          <ci>S</ci>
          <apply>
            <divide />
            <apply>
              <power />
              <ci>R</ci>
              <ci>n</ci>
```

```
            </apply>
            <apply>
              <plus />
              <apply>
                <power />
                <ci>K</ci>
                <ci>n</ci>
              </apply>
              <apply>
                <power />
                <ci>R</ci>
                <ci>n</ci>
              </apply>
            </apply>
        </apply>
        </math>
    </computeChange>
    </listOfChanges>
</model>
```

**Listing 2.40:** *The computeChange element*

The example in Listing 2.40 computes a change of the variable `sensor` of the model `model2`. To do so, it uses the value of the variable `regulator` coming from model `model1`. In addition, the calculation uses two additional parameters, the cooperativity `n`, and the sensitivity `K`. The mathematical expression in the mathML then computes the new initial value of `sensor` using the equation: $S = S \times \frac{R^n}{K^n + R^n}$

### 2.2.6 Simulation

A simulation is the execution of some defined algorithm(s). Simulations are described differently depending on the type of simulation experiment to be performed (Figure 2.12).

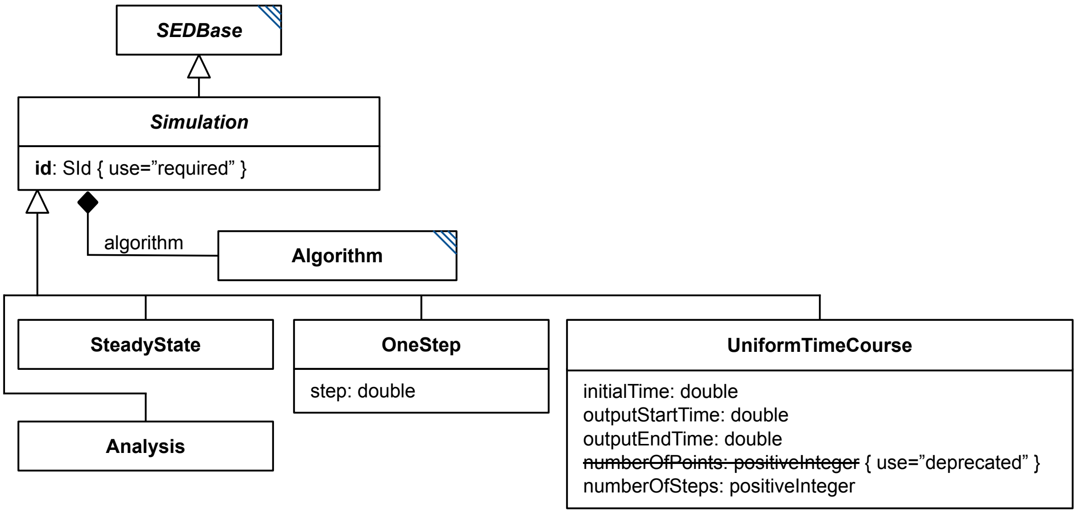

**Figure 2.12:** *The definitions of the Simulation, SteadyState, OneStep, UniformTimeCourse, and Analysis classes*

Simulation is an abstract class and serves as the container for the different types of simulation experiments. SED-ML Level 1 Version 5 provides the predefined simulation classes UniformTimeCourse, OneStep, SteadyState, and Analysis.

Each instance of the Simulation class has an unambiguous and mandatory `id`. An additional, optional `name` may be given to the simulation. Every simulation has a required element `algorithm` describing the simulation Algorithm.

```
<listOfSimulations>
    <uniformTimeCourse [..]>
        [SIMULATION SPECIFICATION]
    </uniformTimeCourse>
    <uniformTimeCourse [..]>
        [SIMULATION SPECIFICATION]
    </uniformTimeCourse>
```

```
</listOfSimulations>
```

**Listing 2.41:** *The SED-ML* `listOfSimulations` *element, defining two different UniformTimecourse simulations*

##### *`algorithm`*

The mandatory child `algorithm` element defines the simulation algorithm or algorithms used for the execution of the simulation. The algorithm is defined via the Algorithm class.

#### 2.2.6.1 *`UniformTimeCourse`*

The UniformTimeCourse class calculates a time course output with equidistant time points. Each instance of the UniformTimeCourse class has, in addition to the elements from Simulation, the mandatory elements `initialTime`, `outputStartTime`, `outputEndTime`, and `numberOfSteps` (Figure 2.12 on the previous page).

Listing 2.42 shows the use of the `uniformTimeCourse` element.

```
<listOfSimulations>
    <uniformTimeCourse id="s1"  name="time course simulation of variable v1 over 100 minutes"
        initialTime="0" outputStartTime="0" outputEndTime="2500" numberOfSteps="1000">
         <algorithm [..] />
    </uniformTimeCourse>
</listOfSimulations>
```

**Listing 2.42:** *The SED-ML* `uniformTimeCourse` *element, defining a uniform time course simulation over 2500 time units with 1000 simulation points.*

##### *`initialTime`*

The attribute `initialTime` of type `double` represents what the time is at the start of the simulation, for purposes of output variables, and for calculating the `outputStartTime` and `outputEndTime`. In most cases, this will be `0.0`. The model must be set up such that `intialTime` is correct internally with respect to any output variables that may be produced. Listing 2.42 shows an example.

##### *`outputStartTime`*

Sometimes a researcher is not interested in simulation results at the start of the simulation, i.e., the initial time. The `UniformTimeCourse` class uses the attribute `outputStartTime` of type `double`, and describes the time (relative to the `intialTime`) that output is to be collected. To be valid the `outputStartTime` cannot be before `initialTime`. For an example, see Listing 2.42.

##### *`outputEndTime`*

The attribute `outputEndTime` of type `double` marks the end time of the simulation, relative to the `initialTime`. See Listing 2.42 for an example.

##### *`numberOfSteps`*

When executed, the `UniformTimeCourse` simulation produces an output on a regular grid starting with `outputStartTime` and ending with `outputEndTime`. The attribute `numberOfSteps` of type `integer` describes the number of steps expected to produce the result. Software interpreting the `UniformTimeCourse` is expected to produce a first outputPoint at time `outputStartTime` and then `numberOfSteps` output points with the results of the simulation. Thus a total of `numberOfSteps + 1` output points will be produced.

Just because the output points lie on the regular grid described above, does not mean that the simulation algorithm has to work with the same step size. Usually the step size the simulator chooses will be adaptive and much smaller than the required output step size. On the other hand a stochastic simulator might not have any new events occurring between two grid points. Nevertheless the simulator has to produce data on this regular grid. For an example, see Listing 2.42.

This attribute used to be named `numberOfPoints`, but was defined to be 'the number of output points minus one', which was confusing. The old name is thus deprecated, and the new name is more in line with its definition.

##### 2.2.6.2 OneStep

The OneStep class calculates one further output step for the model from its current state. Each instance of the OneStep class has, in addition to the elements from Simulation, the mandatory element `step` (Figure 2.12 on page 40).

Listing 2.43 shows the use of the `oneStep` element.

```
<listOfSimulations>
    <oneStep id="s1" step="0.1">
        <algorithm kisaoID="KISAO:0000019" />
    </oneStep>
</listOfSimulations>
```

**Listing 2.43:** *The SED-ML* `oneStep` *element, specifying to apply the simulation algorithm for another output step of size 0.1.*

*step*

The OneStep class has one required attribute `step` of type `double`. It defines the next output point that should be reached by the algorithm, by specifying the increment from the current output point. Listing 2.43 shows an example.

Note that the `step` does not necessarily equate to one integration step. The simulator is allowed to take as many steps as needed. However, after running oneStep, the desired output time is reached.

##### 2.2.6.3 SteadyState

The SteadyState represents a steady state computation (as for example implemented by NLEQ or Kinsolve). The SteadyState class has no additional elements than the elements from Simulation (Figure 2.12 on page 40).

Listing 2.44 shows the use of the `steadyState` element.

```
<listOfSimulations>
    <steadyState id="steady">
        <algorithm kisaoID="KISAO:0000282" />
    </steadyState >
</listOfSimulations>
```

**Listing 2.44:** *The SED-ML* `steadyState` *element, defining a steady state simulation with id* `steady`*.*

##### 2.2.6.4 Analysis

The Analysis represents any sort of analysis or simulation of a Model, entirely defined by its child Algorithm. If a simulation can be defined by a different Simulation, that should be used instead, so that tools are more likely to recognize the request. But for any simultion or any analysis not covered by SteadyState, OneStep, or UniformTimeCourse, the only thing necessary is a KiSAO term for the Algorithm defining what to do. The following examples illustrate analyses that could not be created with other SED-ML Simulation classes:

Listing 2.45 shows the use of the `analysis` element.

```
<listOfSimulations>
    <analysis id="time_course_to_stop_condition">
        <algorithm kisaoID="KISAO:0000263"name="NFSim">
            <algorithmParameter kisaoID="KISAO:0000525" value="ObsA&gt;9"/>
            <algorithmParameter kisaoID="KISAO:0000840" value="0" name="start time"/>
            <algorithmParameter kisaoID="KISAO:0000841" value="10000" name="max end time"/>
            <algorithmParameter kisaoID="KISAO:0000842" value="0.5" name="observed step size"/>
        </algorithm>
    </analysis >
</listOfSimulations>
```

**Listing 2.45:** *The SED-ML* `analysis` *element, defining a time course with a stop condition (ObsA* $<$ *9).*

Listing 2.46 shows the use of the `analysis` element.

```
<listOfSimulations>
    <analysis id="non_uniform_time_course">
        <algorithm kisaoID="KISAO:0000057" name="Brownian diffusion Smoluchowski method">
            <algorithmParameter kisaoID="KISAO:0000525" value="ObsA&gt;9" name="stop condition"/>
            <algorithmParameter kisaoID="KISAO:0000840" value="0" name="start time"/>
            <algorithmParameter kisaoID="KISAO:0000841" value="100" name="max end time"/>
```

```
        </algorithm>
    </analysis >
</listOfSimulations>
```

**Listing 2.46:** *The SED-ML* `analysis` *element, defining a non-uniform time course.*

Listing 2.47 shows the use of the `analysis` element.

```
<listOfSimulations>
    <analysis id="non_uniform_time_course">
        <algorithm kisaoID="KISAO:0000662" name="Klarner ASP logical model trap space identification
             method">
            <algorithmParameter kisaoID="KISAO:0000216" value="true" name="use reduced model"/>
        </algorithm>
    </analysis >
</listOfSimulations>
```

**Listing 2.47:** *The SED-ML* `analysis` *element, defining the Klarner ASP logical model trap space identification method, using the reduced model.*

#### 2.2.7 `AbstractTask`

In SED-ML the subclasses of AbstractTask define which Simulations should be executed with which Models in the simulation experiment. AbstractTask is the base class of all SED-ML tasks, i.e. Task and RepeatedTask. It inherits the attributes and children of *SEDBase*, but changes the `id` attribute to be required instead of optional.

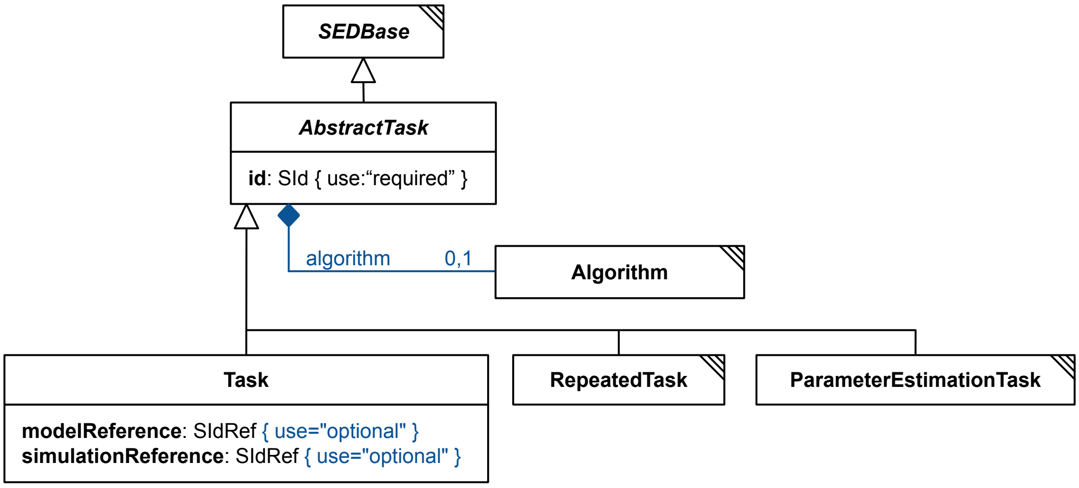

**Figure 2.13:** *The SED-ML AbstractTask and Task classes. The RepeatedTask and ParameterEstimationTask classes are defined below.*

##### *`algorithm`*

The optional child `algorithm` element defines the algorithm used for the execution of the *AbstractTask*. The algorithm is defined via the Algorithm class.

##### *2.2.7.1 `Task`*

A Task links a Model to a certain Simulation description via their respective identifiers (Figure 2.13), using the `modelReference` and the `simulationReference`. The task class inherits the attributes and children of the *AbstractTask*.

##### *`modelReference`*

The optional `modelReference` attribute of type SIdRef must, if defined, refer to a Model. Inside a RepeatedTask, the state of the model may have been changed, otherwise, the Model is assumed to be in its initial state. If the child Algorithm is not defined, this attribute is required.

*simulationReference*

The optional `simulationReference` attribute of type SIdRef must, if defined, refer to a Simulation. If the child Algorithm is not defined, this attribute is required.

In SED-ML it is only possible to link one Simulation description to one Model at a time. However, one can define as many tasks as needed within one experiment description. Please note that the tasks may be executed in any order, as determined by the implementation.

In the example, a simulation setting `simulation1` is applied first to `model1` and then to `model2`.

```
<listOfTasks>
    <task id="t1" name="task definition" modelReference="model1"
        simulationReference="simulation1" />
    <task id="t2" name="another task definition" modelReference="model2"
        simulationReference="simulation1" />
</listOfTasks>
```

**Listing 2.48:** *The* `task` *element*

#### 2.2.7.2 Repeated Task

The RepeatedTask (Figure 2.14) provides a looping construct, allowing complex tasks to be composed from individual tasks. The RepeatedTask performs a specified task (or sequence of tasks as defined in the `listOfSubTasks`) multiple times (where the exact number is specified through a Range construct as defined in `range`), while allowing specific quantities in the model or models to be altered at each iteration (as defined in the `listOfChanges`).

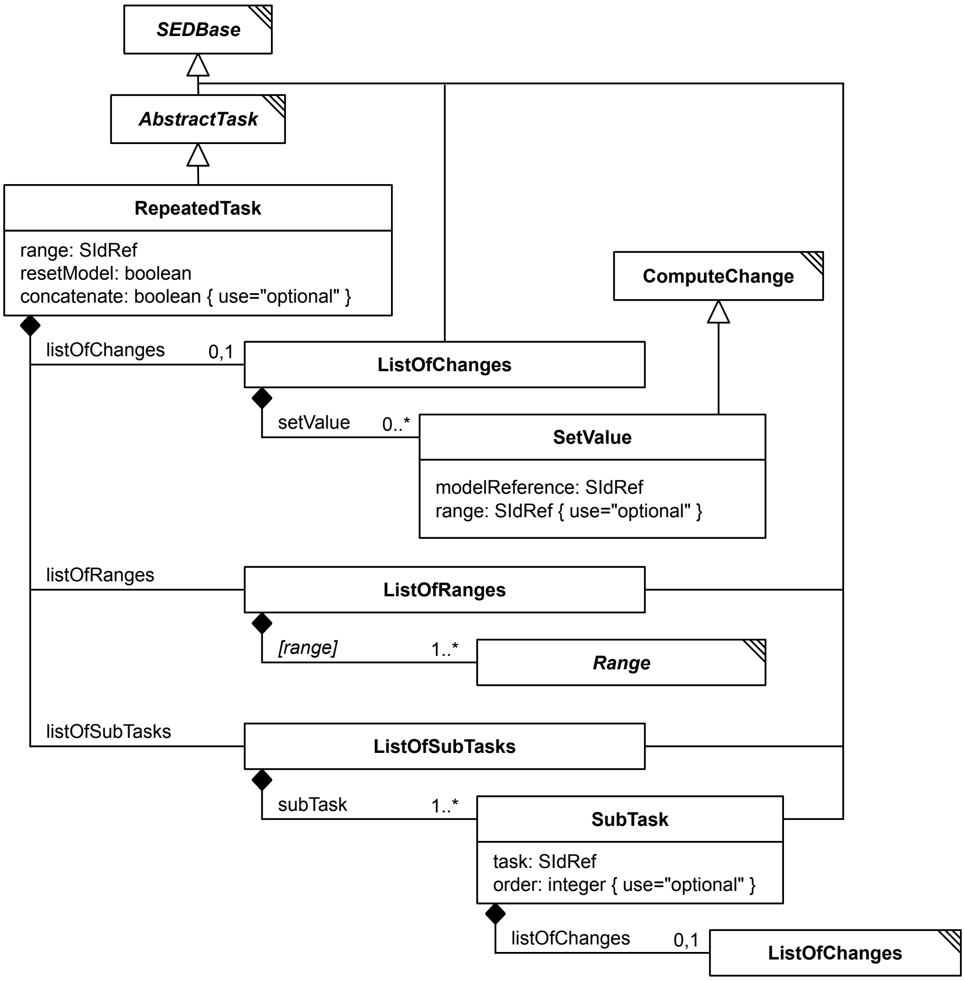

**Figure 2.14:** *The SED-ML RepeatedTask class*

The RepeatedTask inherits the required attribute `id` and optional attribute `name` from *AbstractTask*. Additionally it has the two required attributes `range` and `resetModel`, an optional attribute `concatenate`, and the child elements `listOfRanges` (required), `listOfChanges` (optional) and `listOfSubTasks` (required).

The order of activities within each iteration of a RepeatedTask is as follows:

- The entire model state for any involved Model is reset if specified by the `resetModel` attribute.
- Any changes to the model or models specified by SetValue objects in the `listOfChanges` are applied to each Model.

Then, for each SubTask child of the RepeatedTask, in the order specified by its `order` attribute:

- Any AlgorithmParameter children of the associated Simulation are applied (with the possible exception of the `seed`; see Section 2.1.7.1).
- Any SetValue children of the SubTask are applied to the relevant Model.
- The referenced Task or RepeatedTask of the SubTask is executed.

Listing 2.49 shows the use of the `repeatedTask` element. In the example, `task1` is repeated three times, each time with a different value for a model parameter `w`.

```
<task id="task1" modelReference="model1" simulationReference="simulation1" />
<repeatedTask id="task3" resetModel="false" range="current"
    xmlns:s='http://www.sbml.org/sbml/level3/version1/core'>
  <listOfRanges>
    <vectorRange id="current">
        <value> 1 </value>
        <value> 4 </value>
        <value> 10 </value>
    </vectorRange>
  </listOfRanges>
  <listOfChanges>
     <setValue target="/s:sbml/s:model/s:listOfParameters/s:parameter[@id='w']" modelReference="model1">
       <listOfVariables>
         <variable id="val" name="current range value" target="#current" />
       </listOfVariables>
       <math xmlns="http://www.w3.org/1998/Math/MathML">
         <ci> val </ci>
       </math>
     </setValue>
  </listOfChanges>
  <listOfSubTasks>
    <subTask task="task1" />
  </listOfSubTasks>
</repeatedTask>
```

**Listing 2.49:** *The `repeatedTask` element*

#### *range*

The RepeatedTask has a required attribute `range` of type SIdRef. It specifies which `range` defined in the `listOfRanges` this repeated task iterates over. Listing 2.49 shows an example of a repeatedTask iterating over a single range comprising the values: `1`, `4` and `10`. If there are multiple ranges in the `listOfRanges`, then only the master `range` identified by this attribute determines how many iterations there will be in the repeatedTask. All other ranges must allow for at least as many iterations as the master range, and will be moved through in lock-step; their values can be used in setValue constructs.

#### *resetModel*

The repeatedTask has a required attribute `resetModel` of type `boolean`. It specifies whether the model or models should be reset to the initial state before processing an iteration of the defined subTasks. Here initial state refers to the state of the model or models as given in the `listOfModels`.

In the example in Listing 2.49 the repeated task is not to be reset, so a change is made, `task1` is carried out, another change is made, then `task1` continues from there, another change is applied, and `task1` is carried out a last time.

When the `resetModel` attribute is set to "`true`", the individual repeats may be executed in parallel.

##### concatenate

The RepeatedTask has an optional attribute **concatenate** of type `boolean`. It specifies whether the output of the subtasks should be appended to the results of the previous outputs ("`true`"), or whether it should be added in parallel, as a new dimension of the output ("`false`").

If this attribute is not defined, the interpreter may either concatenate or parallelize the results. As this makes the results less comparable between interpreters, it is strongly suggested that this attribute be defined.

##### listOfChanges

The optional `listOfChanges` element contains one or many SetValue elements. These elements allow the modification of values in the model or models prior to the next iteration of the RepeatedTask.

##### listOfSubTasks

The required `listOfSubTasks` contains one or more subTasks that specify which Tasks are performed in every iteration of the RepeatedTask. All subTasks have to be carried out sequentially, each continuing from the current model state or states (i.e. as at the end of the previous subTask). If the `concatentate` attribute is set "`true`", the results are concatenated (thus appearing identical to a single complex simulation), and if set "`false`", the results are added to a matrix with the additional dimension of the repeated task. The order in which to run multiple subTasks must be specified using the `order` attribute on the subTask. Subtasks can also be executed in parallel when they do not share any state. Interpreters can determine this from the descriptions of the subtasks.

```
<listOfSubTasks>
    <subTask task="task1" order="2"/>
    <subTask task="task2" order="1"/>
</listOfSubTasks>
```

**Listing 2.50:** *The `subTask` element. In this example the task `task2` must be executed before `task1`.*

##### listOfRanges

The `listOfRanges` defines one or more ranges used in the repeatedTask.

### 2.2.8 Task components

#### 2.2.8.1 *SubTask*

A SubTask (Figure 2.14 on page 44) defines the subtask which is executed in every iteration of the enclosing RepeatedTask. The SubTask has a required attribute `task` that references the `id` of another *AbstractTask*. The order in which to run multiple subTasks must be specified via the required attribute `order`. It may contain a child ListOfChanges to specify any changes that apply at the beginning of the particular subtask, in contrast to the ListOfChanges child of the RepeatedTask itself, which specifies changes that apply before any of the subtasks.

##### task

The required element `task` of data type SIdRef specifies the *AbstractTask* executed by this SubTask.

##### order

The required attribute `order` of data type `integer` specifies the order in which to run multiple subTasks in the `listOfSubTasks`. To specify that one subTask should be executed before another its `order` attribute must have a lower number (e.g. in Listing 2.50).

Leaving the `order` undefined for a SubTask implies that the SubTask may be executed before or after any other SubTask. Giving the same `order` to multiple SubTask elements is an explicit statement that each SubTask in the group may be executed before or after any other SubTask in the group. It is recommended that users always explicitly set the `order` attribute for this reason.

Any `order` value does not imply whether the SubTask may be executed in parallel with other SubTask elements. Interpreters who wish to parallelize subtasks should operate from the assumption that in the default case, each SubTask would be executed in some order, and adjust accordingly.

##### *listOfChanges*

The SetValue children of the ListOfChanges of this SubTask define changes to apply to the model state or states before this SubTask is carried out. This allows model changes between individual SubTask elements, perhaps based on the changed state of the model itself. The set of all SetValue children of the first SubTask are applied after the set of SetValue children of the RepeatedTask itself.

#### 2.2.8.2 `SetValue`

The SetValue class (Figure 2.14 on page 44) allows the modification of a Model. Each SetValue in the ListOfChanges child of the RepeatedTask fires before each repeat, and each SetValue in the ListOfChanges child of a SubTask fires before the execution of that SubTask.

SetValue inherits from the ComputeChange class, which allows it to compute arbitrary expressions involving a number of variables and parameters. SetValue inherits the standard attributes and children from *SEDBase*, a required **`target`** and optional **`symbol`** from ComputeChange, and adds a mandatory **`modelReference`** attribute and the optional attribute **`range`**.

The value to be changed is identified via the combination of the attributes **`modelReference`**, **`symbol`**, and **`target`**, in order to select an implicit or explicit variable within the referenced model.

The Math contains the expression computing the value by referring to optional parameters, variables or a range. In contrast to functionalRange, variable references in setValue retrieve always the current value of the model variable or range at the current iteration of the enclosing repeatedTask.

```
<listOfChanges>
    <setValue target="/s:sbml/s:model/s:listOfParameters/s:parameter[@id='w']"
        range="current" modelReference="model1">
        <math xmlns="http://www.w3.org/1998/Math/MathML">
            <ci> current </ci>
        </math>
    </setValue>
</listOfChanges>
```

**Listing 2.51:** *A `setValue` element setting `w` to the values of the range with id `current`.*

##### *modelReference*

The required element **`modelReference`** of data type SIdRef specifies the Model this SetValue is to modify. Each SetValue elements in the same RepeatedTask may potentially reference a different Model.

##### *range*

The optional attribute **`range`** of data type SIdRef, if defined, must reference a Range child of the parent RepeatedTask.

As in functionalRange, the attribute **`range`** may be used as a shorthand to specify the **`id`** of another Range. The current value of the referenced range may then be used within the Math defining this FunctionalRange, just as if that range had been referenced using a variable element, except that the **`id`** of the range is used directly. In other words, whenever the expression contains a **`ci`** element that contains the value specified in the **`range`** attribute, the value of the referenced range is to be inserted.

#### 2.2.8.3 Range

The Range class is the abstract base class for the different types of ranges, i.e. UniformRange, VectorRange, FunctionalRange, and DataRange (Figure 2.15 on the next page).

The Range is the iterative element of the repeated simulation experiment. Each Range defines a collection of values to iterate over. Its **`id`** may be used as the **`target`** of a Variable within the RepeatedTask by pre-pending a '#' (i.e. "**`#rangeId`**"). It is used in that context to mean the value of the range for the current iteration of the RepeatedTask.

##### *algorithm*

The optional child **`algorithm`** element may define the algorithm used for the execution of the Range. The algorithm is defined via the Algorithm class.

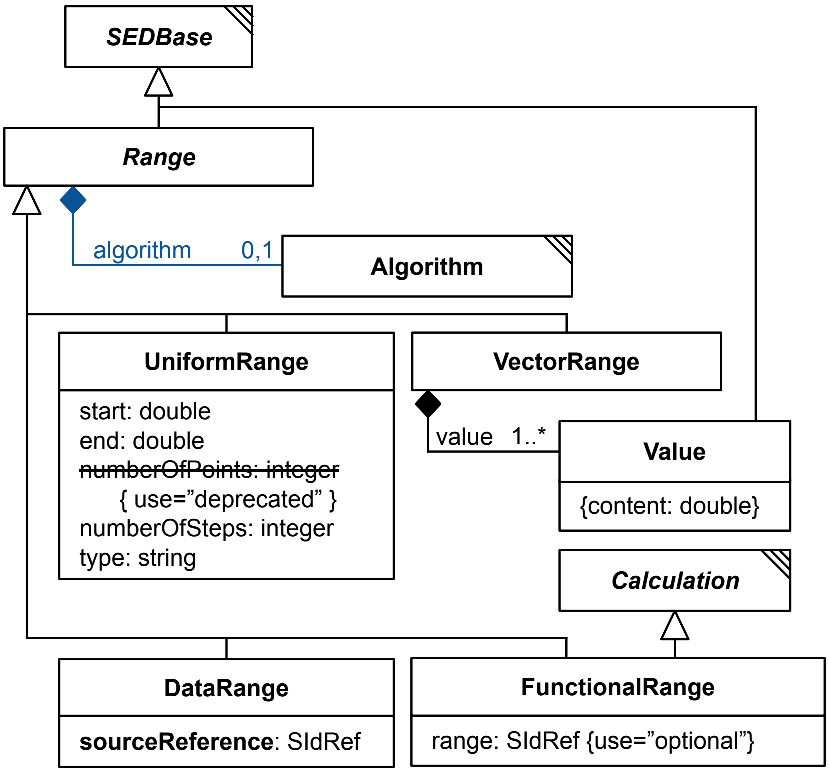

**Figure 2.15:** *The SED-ML Range class*

##### 2.2.8.3.1 `UniformRange`

The UniformRange (Figure 2.15) allows the definition of a Range with uniformly spaced values. In this it is quite similar to what is used in the UniformTimeCourse. The UniformRange is defined via three mandatory attributes: `start`, the start value; `end`, the end value and `numberOfSteps` which defines defines the number of points in addition to the start value (the actual number of points in the range will be `numberOfSteps+1`). A fourth attribute `type` that can take the values `linear` or `log` determines whether to draw the values logarithmically (with a base of 10) or linearly.

The `numberOfSteps` attribute used to be called `numberOfPoints`, but was changed to better reflect the meaning of the attribute. The old attribute name is allowed, but is deprecated. The SED-ML meaning of both attributes is the same, and has not changed.

For example, the following UniformRange will produce `101` values uniformly spaced on the interval $[0, 10]$ in ascending order.

```
<uniformRange id="current" start="0.0" end="10.0" numberOfSteps="100" type="linear" />
```

**Listing 2.52:** *The `UniformRange` element*

The following logarithmic example generates the three values `1`, `10` and `100`.

```
<uniformRange id="current" start="1.0" end="100.0" numberOfSteps="2" type="log" />
```

**Listing 2.53:** *The `UniformRange` element with a logarithmic range.*

##### 2.2.8.3.2 `VectorRange`

The VectorRange (Figure 2.15) describes an ordered collection of real values, listing them explicitly within child `value` elements.

For example, the range below iterates over the values 1, 4 and 10 in that order.

```
<vectorRange id="current">
    <value> 1 </value>
    <value> 4 </value>
    <value> 10 </value>
```

```
</vectorRange>
```

**Listing 2.54:** *The* `VectorRange` *element*

##### 2.2.8.3.3 `Value`

The Value (Figure 2.15 on the preceding page) describes a single value, e.g., the Values in a VectorRange.

##### 2.2.8.3.4 `FunctionalRange`

The FunctionalRange (Figure 2.15 on the previous page) constructs a range through calculations that determine the next value based on the value(s) of other range(s) or model variables. It inherits an optional child Algorithm from Range, adds the optional attribute **`range`**, and also inherits from Calculation, from which it gets the three optional children Math, ListOfVariables, and ListOfParameters.

The optional attribute **`range`** of type SIdRef may be used as a shorthand to specify the **`id`** of another Range. The current value of the referenced range may then be used within the function defining this FunctionalRange, just as if that range had been referenced using a variable element, except that the **`id`** of the range is used directly. In other words, whenever the expression contains a **`ci`** element that contains the value specified in the **`range`** attribute, the value of the referenced range is to be inserted.

The value of any Variable child of a FunctionalRange should be calculated before the `listOfChanges` have been applied to the models in the RepeatedTask and before the first simulation begins, and will not be affected by any SubTask in the RepeatedTask.

For example:

```
<functionalRange id="current" range="index"
    xmlns:s='http://www.sbml.org/sbml/level3/version1/core'>
    <listOfVariables>
        <variable id="w" name="current parameter value" modelReference="model2"
            target="/s:sbml/s:model/s:listOfParameters/s:parameter[@id='w']" />
    </listOfVariables>
    <math xmlns="http://www.w3.org/1998/Math/MathML">
      <apply>
        <times/>
          <ci> w </ci>
          <ci> index </ci>
      </apply>
    </math>
</functionalRange>
```

**Listing 2.55:** *An example of a* `functionalRange` *where a parameter* `w` *of model* `model2` *is multiplied by* `index` *each time it is called.*

Here is another example, this time using the values in a piecewise expression:

```
<uniformRange id="index" start="0" end="10" numberOfSteps="100" />
<functionalRange id="current" range="index">
    <math xmlns="http://www.w3.org/1998/Math/MathML">
        <piecewise>
            <piece>
                <cn> 8 </cn>
                <apply>
                    <lt />
                    <ci> index </ci>
                    <cn> 1 </cn>
                </apply>
            </piece>
            <piece>
                <cn> 0.1 </cn>
                <apply>
                    <and />
                    <apply>
                        <geq />
                        <ci> index </ci>
                        <cn> 4 </cn>
                    </apply>
                    <apply>
                        <lt />
                        <ci> index </ci>
                        <cn> 6 </cn>
                    </apply>
                </apply>
            </piece>
            <otherwise>
                <cn> 8 </cn>
            </otherwise>
        </piecewise>
    </math>
```

```
</functionalRange>
```

**Listing 2.56:** *A* `functionalRange` *element that returns 8 if* `index` *is smaller than 1,* `0.1` *if* `index` *is between 4 and 6, and 8 otherwise.*

##### 2.2.8.3.5 `DataRange`

The DataRange (Figure 2.15 on page 48) constructs a range by reference to external data. It inherits from Range, and adds the required atribute `sourceReference` of type SIdRef. The `sourceReference` must point to a DataDescription with a single dimension, whose values are used as the values of the range.

For example:

```
    <dataRange id="current" sourceReference="dosage_times" />
```

**Listing 2.57:** *An example of a* `dataRange` *which tracks a variable from an external file.*

#### 2.2.9 `ParameterEstimationTask`

The ParameterEstimationTask inherits from *AbstractTask*, and defines a parameter estimation experiment: given a set of constraints, what are the optimal parameter values for a particular model? A ParameterEstimationTask calculates optimal AdjustableParameter values for every FitExperiment child of the task. It provides access to the optimal values for the estimated parameters, and will also change the model state such that the estimated parameters will have those values. If used in a ParameterEstimationResultPlot, WaterfallPlot, or ParameterEstimationReport, various other pieces of information will be output, as defined in those classes.

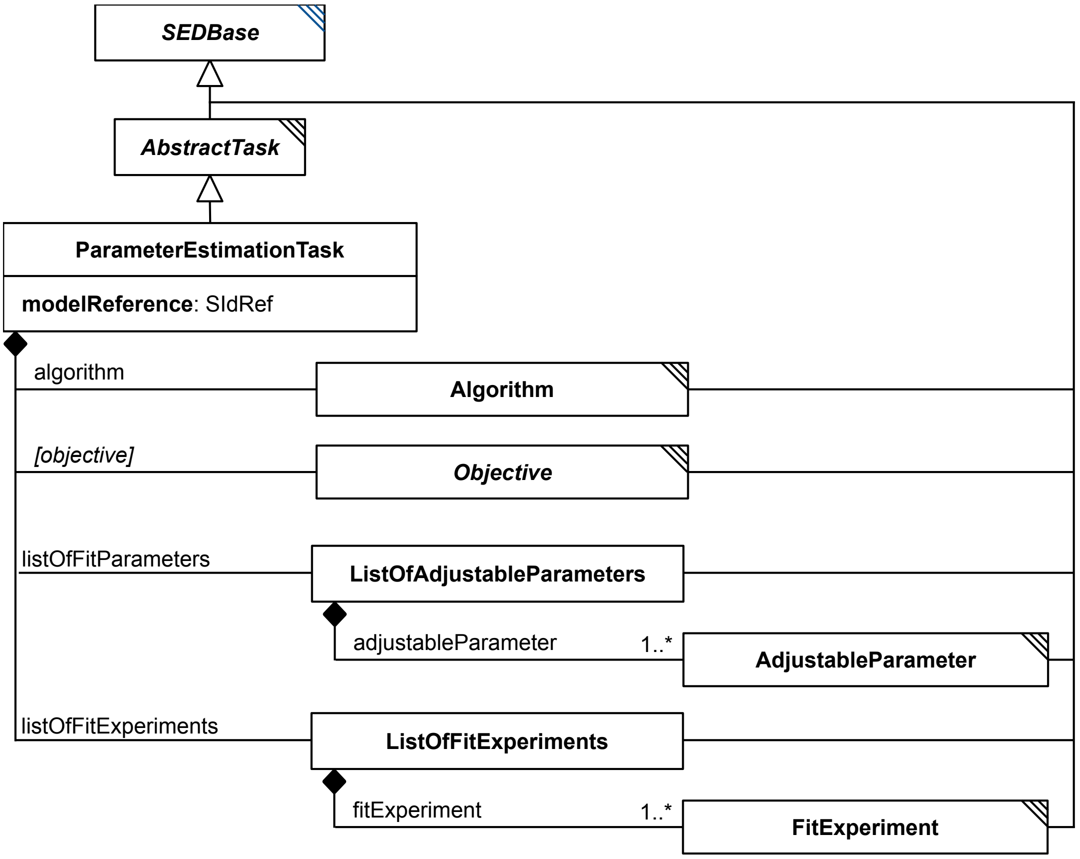

**Figure 2.16:** *The SED-ML ParameterEstimationTask class*

A ParameterEstimationTask has four required children: an Algorithm, an Objective, at least one AdjustableParameter in a ListOfAdjustableParameters, and at least one FitExperiment in a ListOfFitExperiments. It also has a required `modelReference` attribute of type SIdRef.

*`modelReference`*

The `modelReference` attribute of data type SIdRef is used to reference a Model in the same SED-ML Document. This model is the one to be used for parameter fitting.

*`algorithm`*

The inherited `algorithm` child of a ParameterEstimationTask is changed to be required: it defines the Algorithm to be used for parameter fitting. Any algorithm parameters must be included as child AlgorithmParameter elements. The Algorithm class is defined in section 2.1.7. One particular algorithm parameter is KiSAO:0000498 (“`number of runs`”), which can be used to set up a repeated ParameterEstimationTask.

*`objective`*

The `objective` child of the ParameterEstimationTask defines the objective function to be minimized during the parameter estimation. In Level 1 Version 5, there is only a single Objective option: the LeastSquareObjectiveFunction (called “`leastSquareObjectiveFunction`” instead of “`objective`”). In future versions of SED-ML, other objectives may be introduced that cover additional use cases.

*`adjustableParameters`*

The required ListOfAdjustableParameters child of a ParameterEstimationTask must contain at least one AdjustableParameter. Each AdjustableParameter defines a single parameter to be estimated.

*`fitExperiments`*

The required ListOfFitExperiments child of a ParameterEstimationTask must contain at least one FitExperiment. Each FitExperiment defines a mapping between experimental data and observables from the model as well as any initial conditions that need to be applied to the model.

##### 2.2.9.1 `Objective`

The Objective inherits from *SEDBase*, and does not introduce any new attributes or children. It is an abstract base class intended to (eventually) organize the different objective function possibilities for parameter estimation tasks.

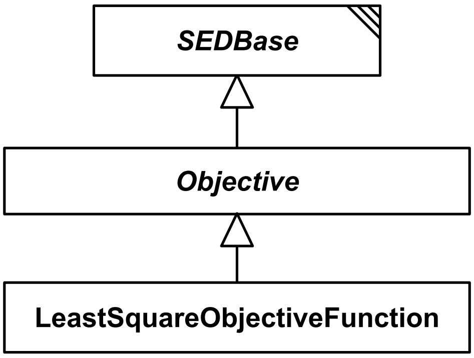

**Figure 2.17:** *The SED-ML Objective and LeastSquareObjectiveFunction classes*

##### 2.2.9.2 `LeastSquareObjectiveFunction`

The LeastSquareObjectiveFunction inherits from Objective, and does not introduce any new attributes or children. Its use indicates that the ParameterEstimationTask is to minimize the least squares of the residuals of the fit experiments to estimate the parameters.

The particular method used to determine the least squares can be defined through the use of Algorithm-

Parameters on the Algorithm of the ParameterEstimationTask.

##### 2.2.9.3 AdjustableParameter

The AdjustableParameter inherits from *SEDBase*, and adds a required attribute `target` of type Target, a required child Bounds, and an optional child ListOfExperimentReferences with zero or more ExperimentReference elements, and an optional attribute `initalValue` of type `double`.

The `target` of an AdjustableParameter must point to an adjustable element of the Model referenced by the parent ParameterEstimationTask. This element is one of the elements whose value can be changed by the task in order to optimize the fit experiments.

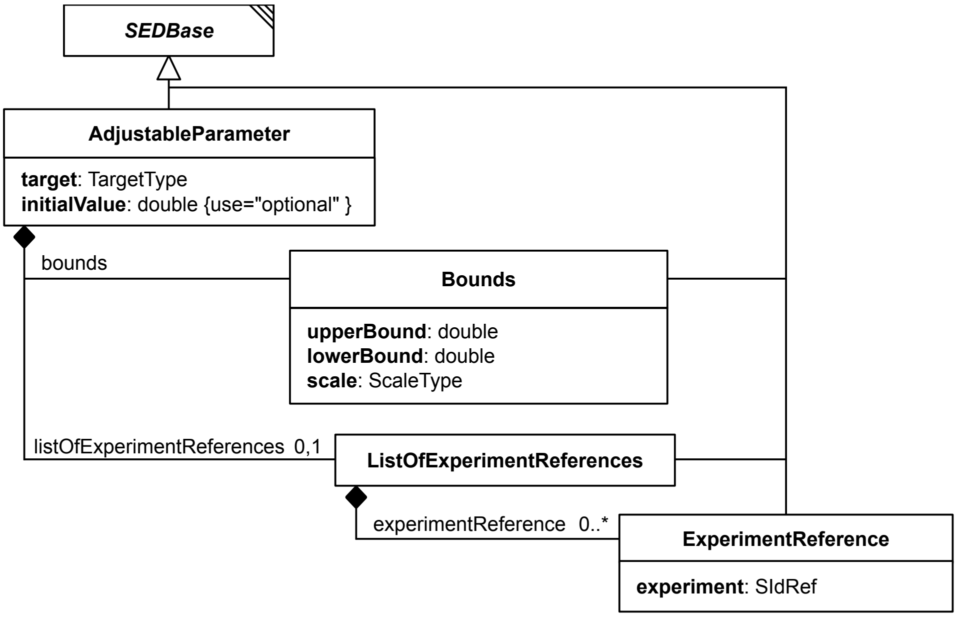

**Figure 2.18:** *The SED-ML AdjustableParameter, Bounds, ListOfExperimentReferences, and ExperimentReference classes*

The `initialValue`, if defined, is the value that the AdjustableParameter is to be set at the beginning of the ParameterEstimationTask. Otherwise, the value of the AdjustableParameter at the model's current state is used, unless that value is outside the `upperBound` and `lowerBound`, in which case any value between or including those values is allowed.

The required Bounds child of the AdjustableParameter defines the allowed range of values for the targeted element.

If an AdjustableParameter has no ExperimentReference children, it is adjusted for every FitExperiment. If an AdjustableParameter has one or more ExperimentReference children, it is only adjusted in those experiments; in all other experiments it retains its initial value (the value of the optional `initialValue` of the AdjustableParameter, if defined, or the value it obtained from the model, if not).

##### 2.2.9.4 Bounds

A Bounds object defines the allowable range of values for an AdjustableParameter. A Bounds inherits from *SEDBase*, and adds three required attributes (`upperBound` and `lowerBound`, both of type `double`, and `scale`, of type ScaleType), and one optional attribute (`initialValue`, of type `double`).

The `lowerBound` defines the lowest value the parent AdjustableParameter may take during the ParameterEstimationTask, and `upperBound` the highest, with both values being legal outputs of the system. The `lowerBound` must be less than or equal to the `upperBound`, though if it is equal, there is nothing to optimize, since only that single value is allowed.

The `scale`, of type ScaleType, defines the structure of the search space between the upper and lower bounds. The allowed values are:

- linear: The bounds enclose a linear search space
- log: The bounds enclose a search space scaled by its natural log.
- log10: The bounds enclose a search space scaled by its log base-10 values.

##### 2.2.9.5 *ExperimentReference*

An ExperimentReference inherits from *SEDBase* and adds the single required attribute `experiment`, of type SIdRef, which must point to a FitExperiment in the same ParameterEstimationTask.

##### 2.2.9.6 *FitExperiment*

The FitExperiment inherits from *SEDBase*, and adds the required attribute `type` of type ExperimentType, a required Algorithm child, and a required ListOfFitMappings child which must in turn contain one or more FitMapping children.

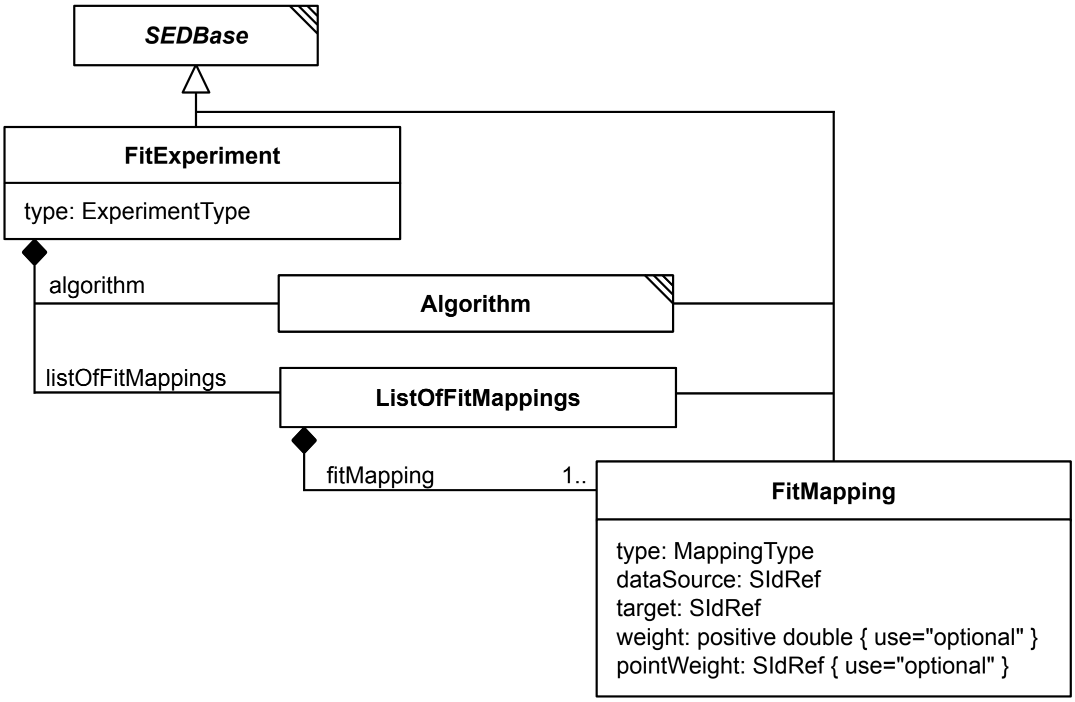

**Figure 2.19:** *The SED-ML FitExperiment, ListOfFitMappings, and FitMappings classes*

A FitExperiment describes an experiment for which there are known experimental conditions, and expected experimental output. The differences between the expected experimental output and the simulated output is used by the Objective to determine the optimal values to use for the AdjustableParameters.

The `type` attribute indicates whether the experiment is a time-course experiment ("`timeCourse`"), or a steady-state experiment ("`steadyState`").

The Algorithm of a FitExperiment describes the algorithm (time course or steady state), and can also be used to define any algorithm parameters of the experiment.

The FitMapping children are used to map externallly-set experimental conditions, observables, and time (in time course experiments) to the model.

##### 2.2.9.7 `FitMapping`

A FitMapping inherits from *SEDBase*, and adds three required attributes `dataSouce` and `target`, both of type SIdRef, `type` of type MappingType, and two optional attributes `weight` of type `positive double`, and `pointWeight` of type SIdRef. A FitMapping is used to correlate elements of a model simulation with data for that simulation, whether time, inputs (experimental conditions) or outputs (observables).

The `type` is of type MappingType, and may take one of the following three values:

- time: Used only in time course simulations, a "`time`" FitMapping maps the time points of the observables to the time points of the simulated output. This also serves to declare what time points must be output by the simulation: unlike a UniformTimeCourse, a FitExperiment time course must at least output the time points mapped here, so that the observables may be directly compared to each other. (Note that here as in elsewhere in SED-ML, 'time' is used as a common label of what is more formally an 'independent variable' for some simulators.)
- experimentalCondition: Any FitMapping of type "`experimentalCondition`" maps a single value to a model element. The model element must be set to the value as part of the model's initial condition.
- observable: An "`observable`" FitMapping maps the output of the simulation to a set of data. These data are used by the Objective to calculate the goodness of fit.

The `dataSource` is an SIdRef to a DataSource in the SED-ML Document. This is a pointer to the expected values of the "`observable`" FitMappings, to the time values of "`time`" FitMappings, or the target initial conditions of "`experimentalConditions`" FitMappings.

The `target` is an SIdRef to a DataGenerator in the SED-ML Document. Any Variable in the referenced DataGenerator must contain a `modelRef` to a Model referenced in an AdjustableParameter that applies to this FitExperiment.

The `weight` or `pointWeight` attributes are used for "`observable`" FitMappings to weight the contribution of that particular observable to the Objective function. For every FitMapping of type "`observable`", either `weight` or `pointWeight` must be defined. For FitMappings with `type` of "`experimentalCondition`" or "`time`", neither attribute may be defined.

If `weight` is defined, that value is used as the weight for all values in the series. If `pointWeight` is defined instead, it must be an SIdRef to a DataGenerator or DataSource with the same dimensionality as the `dataSource`. Each value in the referenced `pointWeight` is then used as the weight of the comparison of the corresponding `dataSource` and `target`.

No weight may be negative or infinite. A `NaN` may be used in a `pointWeight` vector for missing data. Commonly, all weights will have a value between zero and one.

#### 2.2.10 `DataGenerator`

The DataGenerator class prepares the raw simulation results for later output (Figure 2.20). It encodes the post-processing to be applied to the simulation data. The post-processing steps could be anything, from simple normalisations of data to mathematical calculations. It inherits from Calculation, changing the `id` attribute to be required instead of optional.

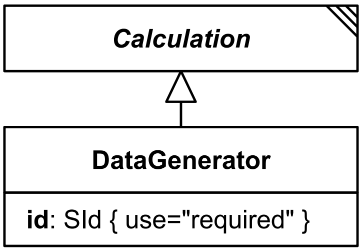

**Figure 2.20:** *The SED-ML DataGenerator class.*

Variable objects in DataGenerator elements may be scalar or multidimensional. If the Math of a DataGenerator attempts to apply functions to multi-dimensional elements, those functions always apply to the individual scalar values of that data. If multiple multidimensional Variable ids are used in the same Math, those ids must each have the same dimensions as each other. No vector or matrix algebra functions such as dot products or cross products are allowed.

A Variable in a DataGenerator may use the id of a DataSource as its `target`, pre-pended by a '`#`', i.e. "`#dataSourceId`". This Variable may be multidimensional, and if so, must follow the above strictures.

When multidimensional data is output to a Report, information about the dimensions should be stored in the output format chosen for the report, such as CSV or HDF5.

It is left up to interpreters how to store or output 'ragged' matrices, where the data in some dimensions might not have the same lengths as each other. One practice is to leave the data in this uneven state; another option is to fill out the 'missing' data with NaNs. The only requirement is that mathematical operations should not be affected by this choice. For example, the 'mean' of a vector should be the same whether or not it was extended with NaNs.

*Output from multiple models*

It is possible to create a RepeatedTask that affects multiple models through different SubTask children. In this situation, individual Variable children of a DataGenerator must define both a `taskReference` to the RepeatedTask and `modelReference` so it's clear which specific element is being tracked. However, the question then becomes: what value does that Variable take while the RepeatedTask is performing a Simulation in a SubTask that does not involve that Model? In this situation, the Variable is assumed to retain its last known value (should it have one) for the duration of the Simulation (which will be its initialized value if no Simulation has been performed yet that affects that Variable). If the model has no initialized value for the element, its value is assumed to be $NaN$.

This is an unusual situation, so much so that different simulators may create different outputs, or fail to implement support for it at all. For this reason, it is recommended that all SubTask elements in a RepeatedTask reference the same Model.

Listing 2.58 shows the use of the `dataGenerator` element. In the example the `listOfDataGenerator` contains two `dataGenerator` elements. The first one, `d1`, refers to the task definition `task1` (which itself refers to a particular model), and from the corresponding model it reuses the symbol `time`. The second one, `d2`, references a particular species defined in the same model (and referred to via the `taskReference="task1"`). The model species with `id PX` is reused for the data generator `d2` without further post-processing.

```
<listOfDataGenerators>
    <dataGenerator id="d1" name="time">
        <listOfVariables>
            <variable id="time" taskReference="task1" symbol="KISAO:0000832" />
        </listOfVariables >
        <listOfParameters />
        <math xmlns="http://www.w3.org/1998/Math/MathML">
            <ci> time </ci>
        </math>
    </dataGenerator>
    <dataGenerator id="d2" name="LaCI repressor">
        <listOfVariables>
            <variable id="v1" taskReference="task1"
                target="/sbml:sbml/sbml:model/sbml:listOfSpecies/sbml:species[@id='PX']" />
        </listOfVariables>
        <math xmlns="http://www.w3.org/1998/Math/MathML">
            <math:ci>v1</math:ci>
        </math>
    </dataGenerator>
</listOfDataGenerators>
```

**Listing 2.58:** *Definition of two* `dataGenerator` *elements,* time *and* LaCI repressor

#### 2.2.11 Output

The Output class describes how the results of a simulation are presented (Figure 2.21 on the next page). The available output classes are *Plot*, Report, ParameterEstimationReport, and Figure, or the Output class itself can be used directly if none of its subclasses apply. The data used in an Output is provided via the DataGenerator class.

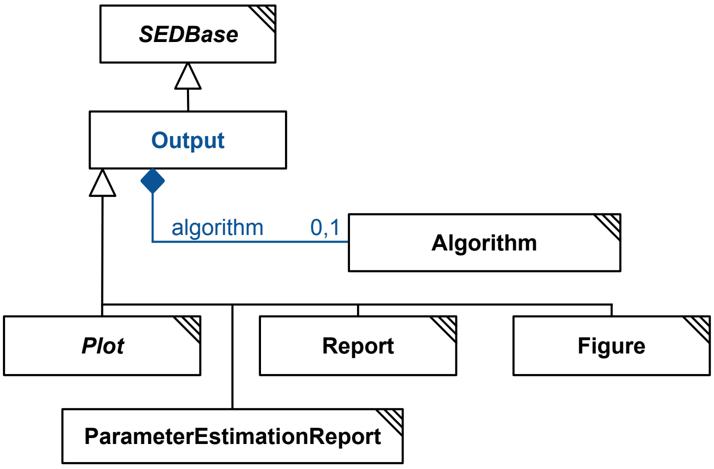

**Figure 2.21:** *The definition of the SED-ML Output class. The subclasses are defined below.*

The Output class inherits the `id` and `name` attributes from *SEDBase*, as well as the optional `annotation` and `notes` chidren. When producing a printed table or figure, users may want to use the `name` as the title, and the `notes` as the legend.

The output of a SED-ML file may be used to compare simulation executions from the same tool or from different tools. As such, interpreters may choose to focus on the output of a SED-ML file, and execute only the tasks necessary to produce this output. Repeated executions of the same SED-ML should always produce comparable output. When a stochastic run is given a seed, interpreters should be aware that users may expect to get identical results from repeated runs on the same architecture, including when tasks are run in parallel.

*`algorithm`*

The optional child `algorithm` element defines the algorithm used for the execution of the Output. The algorithm is defined via the Algorithm class.

Like the Analysis class, the Output class when used on its own represents any sort of output from a simulation experiment, entirely defined by its child Algorithm. If the output can be defined by a different Output object, that should be used instead, so that tools are more likely to recognize the request. But for any output not covered by a *Plot*, Report, Figure, or ParameterEstimationReport subclass, the only thing necessary is a KiSAO term for the Algorithm defining what to do, along with any AlgorithmParameter children to provide more detail. One might use the Output class to define spatial output, for example, or an animation. As long as a KiSAO term can be created to describe the output, it can be captured in an Output.

#### 2.2.12 Plot

The *Plot* is an abstract base class for two- and three-dimensional plot outputs. It defines the size and axes of a plot, as well as whether or not a legend should be displayed.

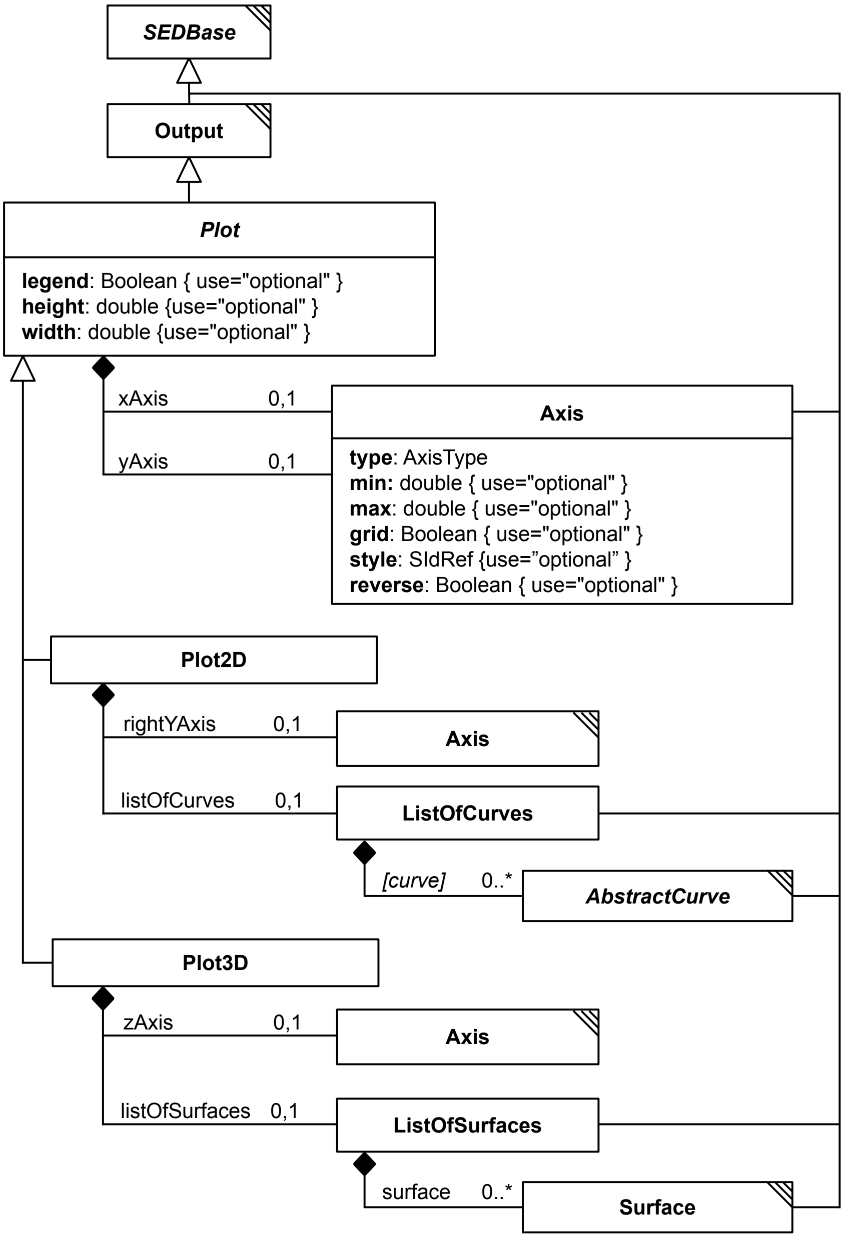

**Figure 2.22:** *The definition of the SED-ML* Plot, *Plot2D*, *Plot3D*, *Axis*, *ListOfCurves*, *and ListOf-Surfaces classes. The* AbstractCurve *and Surface classes are defined below.*

The *Plot* class inherits the attributes and children from *SEDBase*, and adds three optional attributes: `legend`, of type Boolean, `height` of type `double`, and `width` of type `double`. It also defines two optional Axis children, an `xAxis` and a `yAxis`.

*`legend`*

The `legend` attribute defines whether a legend should be displayed ("`true`") or not ("`false`"). The position and styling of the legend is unspecified. If the attribute is not defined, it is up to the tool whether to display the legend or not, and does not mean that the attribute has a default value of "`false`".

*`height` and `width`*

The `height` and `width` elements, both of type `double`, may be used to define the size of the plot, in pixels (or the equivalent in the application's display environment). If either is not defined, the application may choose what size to display the plot.

*xAxis and yAxis*

The optional `xAxis` and `yAxis` children, each of type Axis, define the x and y axes (respectively) by which the Curve or Surface children are to be interpreted. If either child is omitted, that axis is undefined, and it is up to the tool whether and how to display any necessary axes, and to decide whether that axis should be linear or logarithmic.

##### 2.2.12.1 Plot2D

The Plot2D class is used for two dimensional plot outputs. In addition to the features it inherits from *Plot*, it may contain any number of Curve definitions in the `listOfCurves`, as well as an optional child `rightYAxis`.

*rightYAxis*

If a Plot2D contains a child `rightYAxis`, this defines a new Y axis, displayed on the right, which any of the Curve children may be scaled to. Each Curve contains the information about which axis it is to be scaled to. The `rightYAxis` is to be displayed on the right of the plot, and may differ significantly in scale and range from the `yAxis`. A Plot2D with no `yAxis` may not have a `rightYAxis`.

*listOfCurves*

Each child *AbstractCurve* of a Plot2D represents a line to be displayed on the plot. The *AbstractCurve* itself will define what data it contains, and how it should be displayed.

##### 2.2.12.2 Plot3D

The Plot3D class is used for three dimensional plot outputs (Figure 2.21 on page 56). In addition to the elements it inherits from *Plot*, the Plot3D may contain a number of child Surface definitions in a `listOfSurfaces`, and may additionally define a `zAxis` child, of type Axis.

*listOfSurfaces*

Each child Surface of a Plot3D represents a surface to be displayed on the plot. The Surface itself will define what data it contains, and how it should be displayed.

*zAxis*

When a Plot3D contains a child `zAxis`, that Axis defines the characteristics of the z axis. If no `zAxis` is provided, those characteristics are undefined, and the tool may choose how and whether to display that axis, as well as what type it is (linear or logarithmic).

##### 2.2.12.3 Axis

The Axis class is used to define whether an axis for a given *Plot* is linear or logarithmic, and how to display it. It inherits the attributes and children from *SEDBase*, and adds the required attribute `type` of type AxisType (either 'linear' or 'log10'), as well as the optional attributes `min` and `max`, both of type `double`, `grid` of type `boolean`, and `style` of type SIdRef.

*name and id*

The Axis class inherits the `name` and `id` attributes from *SEDBase*. The `name`, if present, should be used as the label for the axis. If it is not present, the `id` may be used.

*type*

The `type` value of "`linear`" means the axis should be scaled linearly, while a value of "`log10`" indicates it should have a log10 scale. Other scalings are not possible in this version of SED-ML. This attribute replaces the "`log`" attributes that used to be present on Curve objects in previous versions of SED-ML.

*min and max*

The `min` and `max` values indicate the minimum and maximum values for the axis. Data points outside of this range should not be shown on the parent *Plot*. Either value may be set or not, and if not set, a value must be chosen for display that is less than (for `min`) or greater than (for `max`) the most extreme value along that axis for any Curve or Surface in that *Plot*. Do note that in some cases, a given Curve may not have any data points associated with one Y Axis, as its data may be associated with the alternative Y Axis.

Note that `min` and `max` will have the same units as the data plotted along it, regardless of the value of the `type`. An axis with a `min` value of "`1`" and a `max` value of "`100`" will either be plotted with '50' halfway between those two extremes if the `type` is "`linear`", or with '10' halfway between those two extremes if the `type` is "`log10`".

*`grid`*

The `grid` attribute indicates whether grid lines should ("`true`") or should not ("`false`") be displayed in the *Plot* for tick marks along that axis. If the `grid` attribute is not defined, this means it is up to the tool whether or not to display the grid lines; it does not have a default value of "`false`".

*`style`*

The `style` attribute, if present, must be an SIdRef to a Style in the same SED-ML Document. If defined, it indicates how to display the axis itself, for features such as color and/or line thickness for the axis and its labels. If not present, any style may be used. Note that it is possible to suppress an axis from being displayed entirely if the corresponding Style of an Axis has a `line` with a `style` of "`none`".

*`reverse`*

The `reverse` attribute indicates whether the axis should be plotted from the minimum value to the maximum value ("`false`") or from the maximum value to the minimum value ("`true`") (i.e. left to right or bottom to top, depending on the axis). If not defined, either is technically possible, but should be assumed to go from minimum to maximum.

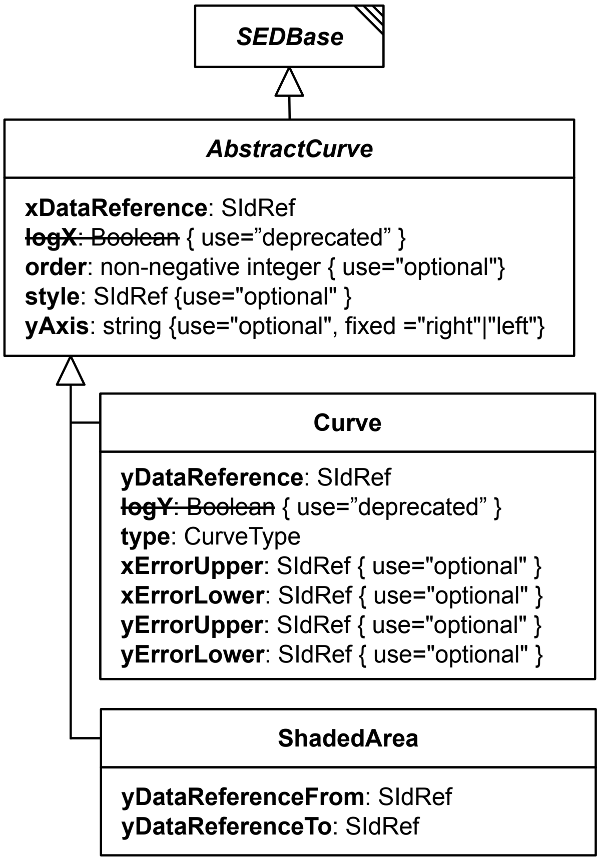

**Figure 2.23:** *The definition of the SED-ML* AbstractCurve*, Curve, and ShadedArea classes.*

##### 2.2.12.4 `AbstractCurve`

An *AbstractCurve* is a two-dimensional Output component representing a (processed) simulation result (Figure 2.23 on the previous page). Zero or more *AbstractCurve* instances define a Plot2D (Figure 2.21 on page 56). The *AbstractCurve* class defines the attributes common to the Curve and ShadedArea child classes. In addition to the optional `id` and `name` attributes it inherits from *SEDBase*, it also defines the required attribute `xDataReference`, and the optional attributes `order`, `style`, and `yAxis`. It is also legal but discouraged to include an attribute `logX`.

The `name` of the *AbstractCurve* should be used to label the curve in the given Plot2D, or, if `name` is not defined, the `id` may be used. If neither are present, the `name` or `id` of the referenced `yDataReference` may be used in the case of a Curve or the `yDataReferenceFrom` and/or `yDataReferenceTo` in the case of a ShadedArea. Because of the complications this can engender, it is highly recommended to define the `name` of all *AbstractCurve* elements.

*`xDataReference`*

The `xDataReference` attribute must be an SIdRef to a DataGenerator in the same SED-ML Document. The referenced DataGenerator will contain the information for the x coordinates for the data to be plotted. If the y-coordinate data is ordinal or categorical, this attribute should point to a simple ordinal DataGenerator.

The dimensionality of the `xDataReference` must match the y data, but need not be one-dimensional. When a curve is being displayed, each one-dimensional vector within the x and y data should be displayed on the same plot. This will effectively flatten the data to the two dimensions of the plot. When being displayed as lines, each vector should be plotted separately, so that the plot is not overlaid with spurious lines from the end of one vector to the beginning of the next.

*`order`*

The `order` attribute is of type `non-negative integer` and, if present, defines the order in which this Curve must be displayed relative to other Curve elements in the same *Plot*. A Curve with a lower `order` will be added earlier to the displayed curves. This means that for lines, the curve with the highest `order` will be fully visible, while a Curve with a lower `order` may be hidden by a Curve with a higher `order`. A Curve with no `order` may be displayed in front or behind any other Curve. For adjacent bars, the bar with the lower `order` is presented to the left of any bar with a higher `order`. For stacked bars, the bar with the lower `order` is presented underneath any bar with a higher `order`. As with lines, any bar with no `order` defined may be placed in any position relative to the other bars in the Curve.

*`style`*

The `style` attribute is of type SIdRef and, if present, must reference a Style in the same SED-ML Document. It can be used to indicate styling information for the line, marker, and/or fill for this Curve or ShadedArea. If not present, any style may be used.

*`yAxis`*

The `yAxis` attribute is of type `string` and must be defined if the parent *Plot* defines both a `yAxis` and a `rightYAxis`. If it has the value of “`left`”, it means that the data is to be displayed corresponding to the `yAxis` of the parent *Plot*, and if it has the value of “`right`”, it means that the data is to be displayed corresponding to the `rightYAxis` of the parent *Plot*. If the parent *Plot* has no defined `rightYAxis`, this attribute must not be defined.

*`logX` (deprecated)*

The `logX` attribute, of type `Boolean`, was used in previous versions of SED-ML to indicate whether the x axis of the *Plot* should be linear or log10. This allowed mutliple Curve objects in the same *Plot* to contradict each other, and has therefore been moved to Axis. The `logX` attribute on Curve has therefore been deprecated, and will always be ignored.

##### 2.2.12.5 Curve

A Curve is a two-dimensional Output component representing a (processed) simulation result (Figure 2.21 on page 56). Zero or more Curve instances define a Plot2D (Figure 2.21 on page 56). In addition to the attributes it inherits from *AbstractCurve* (and *SEDBase*), it also defines the required attribute `yDataReference` of type SIdRef. It also defines the optional attribute `type` of type CurveType, and the optional attributes `xErrorUpper`, `xErrorLower`, `yErrorUpper`, and `yErrorLower`, all of type SIdRef.

*yDataReference*

Like the `xDataReference`, the `yDataReference` must be the `SId` of a DataGenerator in the same SED-ML Document. The referenced DataGenerator will contain the information for the y coordinates for the data to be plotted. The dimensions of the y data should match the x data. If the y data is multi-dimensional (such as time course data over several stochastic replicates), one dimension should match the x data (time, in our example), and the other dimension should simply be replicated as separate curves on the same plot (with the same style and label).

*type*

The optional `type` attribute is of type CurveType, and determines the kind of curve being displayed. The possible values are:

- **points**: The curve is plotted as points, with the y values defined via the `yDataGenerator`. The x values of the points are plotted at the `xDataGenerator` position. Depending on the `style`, markers and/or a line are plotted. To display only a set of markers the Line from its Style is set to have a `type` of "`none`". Similarly, to display a line only with no markers the Marker from its Style is set to have a `type` of "`none`". (If both are set to "`none`", the curve will not be displayed at all!) The Fill of a Style has no meaning and, if present, will be ignored.

- **bar**: The curve is plotted as bars with the height of the bars defined via the `yDataGenerator` values. The middle of the bars are plotted at the `xDataGenerator` position. The style of the bars is defined via the `style`, with the fill color defined in the Fill and the bar edge style in the Line. The Marker of a Style has no meaning and, if present, will be ignored.

- **barStacked**: The curve is plotted as with `bar`, but stacked instead of adjacent.

- **horizontalBar**: The curve is plotted as a bar plot, but the y axis is vertical and the x axis is horizontal.

- **horizontalBarStacked**: The curve is plotted as a stacked bar plot, but the y axis is vertical and the x axis is horizontal.

*xErrorUpper, xErrorLower, yErrorUpper, and yErrorLower*

The optional attributes `xErrorUpper`, `xErrorLower`, `yErrorUpper`, and `yErrorLower` may be declared to define the error in the data present in the Curve. Each attribute must, if defined, point to a DataGenerator in the same SED-ML Document. The `xErrorUpper` and `xErrorLower` must have the same dimensionality as the `xDataReference`, and the `yErrorUpper` and `yErrorLower` must have the same dimensionality as the `yDataReference`. Each set of data represents the error in that dimension, in distance from the given data point. The `xErrorUpper` refers to the error in the positive direction, and `xErrorLower` refers to the error in the negative direction. To set symmetrical errors `xErrorUpper` and `xErrorLower` should point to the same DataGenerator. The same is true for `yErrorUpper` and `yErrorLower`.

##### 2.2.12.6 ShadedArea

A ShadedArea is an *AbstractCurve* that defines an area instead of a series of points. In addition to what is inherited from *AbstractCurve*, a ShadedArea defines the required attributes `yDataReferenceFrom` and `yDataReferenceTo`, both of which must be an SIdRef for a DataGenerator in the same SED-ML Document. The area between these two sets of points is then filled for display. If the `style` is defined, the Fill of that Style is used to color the fill. The Marker and Line of a Style has no meaning for a ShadedArea and, if present, will be ignored.

*yDataReferenceFrom and yDataReferenceTo*

The attributes `yDataReferenceFrom` and `yDataReferenceTo` are both of type SIdRef, and must reference data of the same dimensionality. The values of the two attributes may be swapped, with the only effect being the direction of the shading between them, if two fill colors are used.

##### 2.2.12.7 Surface

A Surface is a parallel class to *AbstractCurve* that defines a three-dimensional surface instead of a two-dimensional curve (Figure 2.24). In addition to the optional `id` and `name` attributes it inherits from *SEDBase*, it also defines the required attributes `xDataReference`, `yDataReference`, and `zDataReference`, all of type SIdRef. It also defines the optional attributes `style` of type SIdRef, and `type`, of type SurfaceType.

The `name` of the Surface should be used to label the surface in the given Plot3D, or, if `name` is not defined, the `id` may be used. If neither are present, the `name` or `id` of the referenced `zDataReference` may be used. In general, it is highly recommended to define the `name` of all Surface elements.

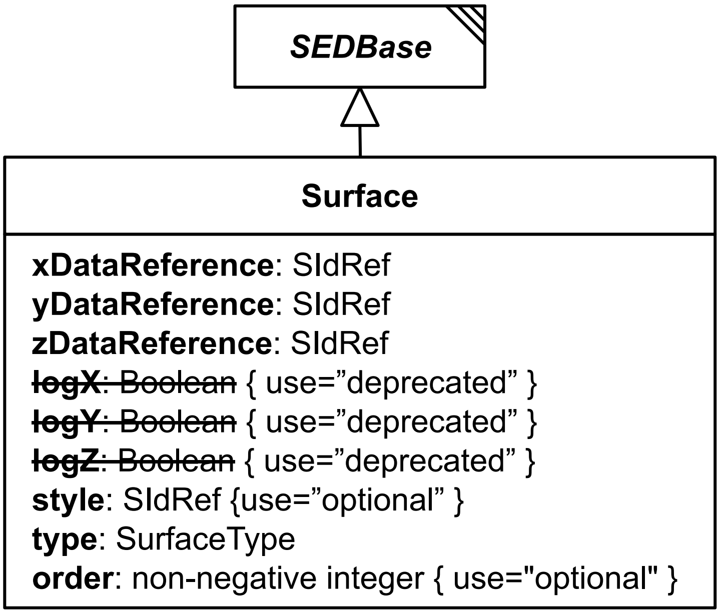

**Figure 2.24:** *The definition of the SED-ML Surface class.*

*xDataReference, yDataReference, and zDataReference*

The three data reference attributes must point to DataGenerator elements in the same SED-ML Document, which define the surface to be plotted. All three attributes are required. If the `zDataReference` is intended to be plotted by index, the `xDataReference` and `yDataReference` attributes should point to DataGenerator elements that generate those indices.

As with an *AbstractCurve*, the dimensionality of the attributes `xDataReference`, `yDataReference` and `zDataReference` must match each other, but need not be one-dimensional. When a surface is being displayed, each one-dimensional vector within the x, y, and z data should be displayed on the same plot. This will effectively flatten the data to the three dimensions of the plot. When the data is being plotted as lines, Each vector should be plotted with its own line, so that the plot is not overlaid with spurious lines from the end of one vector to the beginning of the next.

*style*

The `style` attribute, if defined, must contain the `SId` of a Style object in the same SED-ML Document. This Style determines how any lines, markers, or fills on that surface should be displayed, if present for that type of Surface.

*type*

The `type` attribute, if present, determines the type of surface and how it should be displayed. The options are:

- **parametricCurve**: Each successive data point is plotted in order, potentially joined by a line. If the z data is 2-dimensional instead of a vector, the last point of the first vector should not be connected to the first point of the next. The line and marker styles can be set from the `style` (including removing them if the `type` of either is set to "`none`").
- **surfaceMesh**: The data are plotted as a wireframe, with adjacent-in-space data points connected with lines. The line style can be set from the `style`.
- **surfaceContour**: The data is plotted as a continuous surface. The fill color can be set from the `style`, as can the lines and/or markers, if displaying those elements are desired.
- **contour**: The 3D data are plotted as a 2D surface, with contour lines (similar to elevation plots). The line style can be set from the `style`.
- **heatMap**: The 3D data are plotted as a 2D surface, with color representing the values. The colors can be set from the `fill` of the `style`.
- **bar**: The data is plotted as a 3D bar plot.

*logX, logY, logZ (deprecated)*

The `logX`, `logY` and `logZ` attributes, of type `Boolean`, were used in previous versions of SED-ML to indicate whether the respective axis of the *Plot* should be linear or log10. This allowed multiple objects in the same *Plot* to contradict each other, and has therefore been moved to Axis. The `logX`, `logY` and `logZ` attributes on Surface have therefore been deprecated, and will always be ignored.

##### 2.2.12.8 ParameterEstimationResultPlot

A ParameterEstimationResultPlot class is used to create a default plot from a ParameterEstimationTask. It inherits from *Plot*, and adds a single required attribute `taskReference` of type SIdRef that points to that task.

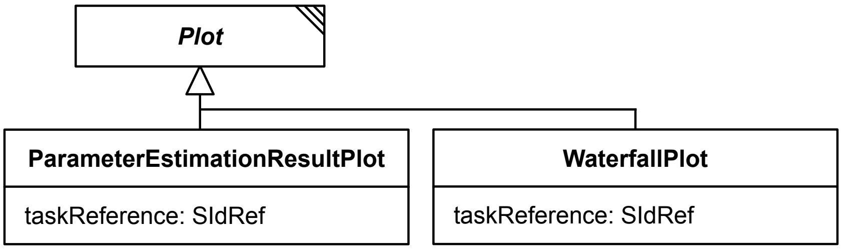

**Figure 2.25:** *The definition of the SED-ML ParameterEstimationResultPlot and WaterfallPlot classes.*

The plot should display the relevant information collected during the parameter estimation, but the specifics may vary from tool to tool depending on the particular method used. At the very least, the optimal AdjustableParameter values should be reported, along with any information that would let the user determine the confidence in those estimates, such as the residuals.

It is possible to reproduce and/or have more control over the contents of a *Plot* that covers the contents of a ParameterEstimationTask by creating DataGenerator elements that use Variable objects using a `dimensionTerm` and referencing particular elements of a ParameterEstimationTask such as the residuals of the Objective, or the overall $\chi^2$ value of the task. This is the only way to get direct control over the Style of anything displayed in a ParameterEstimationResultPlot. But the data itself should be displayed in some form by default in a ParameterEstimationReport.

##### 2.2.12.9 `WaterfallPlot`

The WaterfallPlot class is used to create a default plot of a particular style from a ParameterEstimationTask. It inherits from *Plot*, and adds a single required attribute `taskReference` of type SIdRef that points to that task.

Like a ParameterEstimationResultPlot, a WaterfallPlot displays a range of results and data from a ParameterEstimationTask that might not otherwise be easily accessible. Different tools and different experiments may result in different types and styles of waterfall plots. For an overview of the sort of data present in one, see Gillespie, 2012 [12].

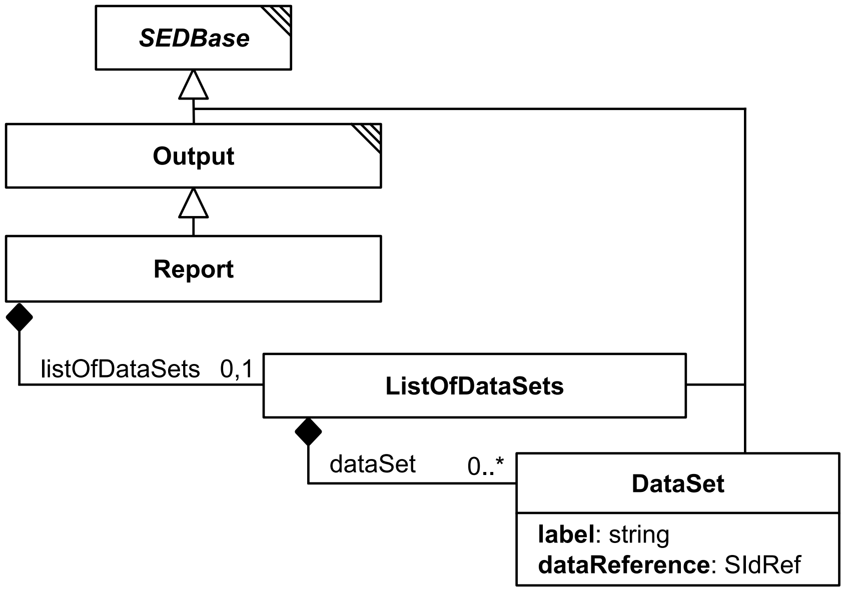

**Figure 2.26:** *The definition of the SED-ML Report, ListOfDataSets, and DataSet classes.*

#### 2.2.13 `Report`

The Report class defines a data map consisting of several single instances of the DataSet in the child `listOfDataSets` (Figure 2.26). Its output returns the simulation result processed via DataGenerators in actual numbers. The elements of the report are defined by creating an instance of the DataSet for each element of the report and are identified by the `label` of the DataSet.

The simulation result itself, i.e. concrete result numbers, are not stored in SED-ML, but the directive how to calculate them from the output of the simulator is provided through the dataGenerator. The encoding of simulation results is not part of SED-ML Level 1 Version 5, but it is recommended that 2D output be exported as CSV files, using the `label` as column headers, and that output with more dimensions be exported as HDF5, again using the `label` to uniquely identify the data sets.

##### 2.2.13.1 `DataSet`

The DataSet class holds definitions of data to be used in the Report class (Figure 2.26). DataSets are labeled references to instances of the DataGenerator class. It defines the required attributes `label` of type `string` and `dataReference` of type SIdRef.

Each data set in a Report must have an unambiguous `label`. A `label` is a human readable descriptor of a data set for use in a Report. In general the Report is a map between labels and data from DataGenerator instances, but can be interpreted as a data table for certain tasks. For example, in the special case of time series results, the report could be a tabular data set with the `label` being the column heading and the time series results being the columns.

*label*

The `label` attribute is of type `string` defines a unique label for every DataSet in a given Report.

*dataReference*

The `dataReference` attribute is of type SIdRef, and must be the ID of a DataGenerator element in the same SED-ML Document. The data produced by that particular DataGenerator fills the according dataSet in the report.

Listing 2.59 shows the use of the `dataSet` element. The example shows the definition of a dataSet. The referenced dataGenerator `dg1` must be defined in the `listOfDataGenerators`.

```
<listOfDataSets>
    <dataSet id="d1" name="v1 over time" dataReference="dg1" label="_1">
</listOfDataSets>
```

**Listing 2.59:** *The SED-ML* `dataSet` *element, defining a data set containing the result of the referenced task*

#### 2.2.14 ParameterEstimationReport

A ParameterEstimationReport class is used to create a default report from a ParameterEstimationTask. It has a single required attribute `taskReference` of type SIdRef that points to that task.

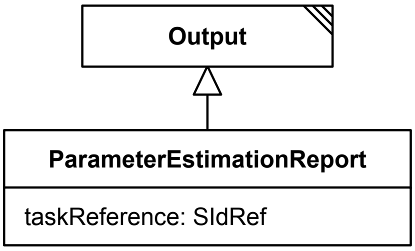

**Figure 2.27:** *The definition of the SED-ML ParameterEstimationReport class.*

The report should include the relevant information collected during the parameter estimation, but the specifics may vary from tool to tool depending on the particular method used. At the very least, the optimal AdjustableParameter values should be reported, along with any information that would let the user determine the confidence in those estimates.

It is possible to reproduce and/or have more control over the contents of a Report that covers the contents of a ParameterEstimationTask by creating DataGenerator elements that use Variable objects using a `dimensionTerm` and referencing particular elements of a ParameterEstimationTask such as the residuals of the Objective, or the overall $\chi^2$ value of the task. But most of these values should be produced by default in a ParameterEstimationReport.

#### 2.2.15 Figure

The Figure class provides a mechanism to arrange and display several *Plot* elements together. It inherits the attributes and children of Output, and additionally defines two required attributes `numRows` and `numCols`, both of type `positive integer`, and can additionally contain any number of SubPlot children through a ListOfSubPlots.

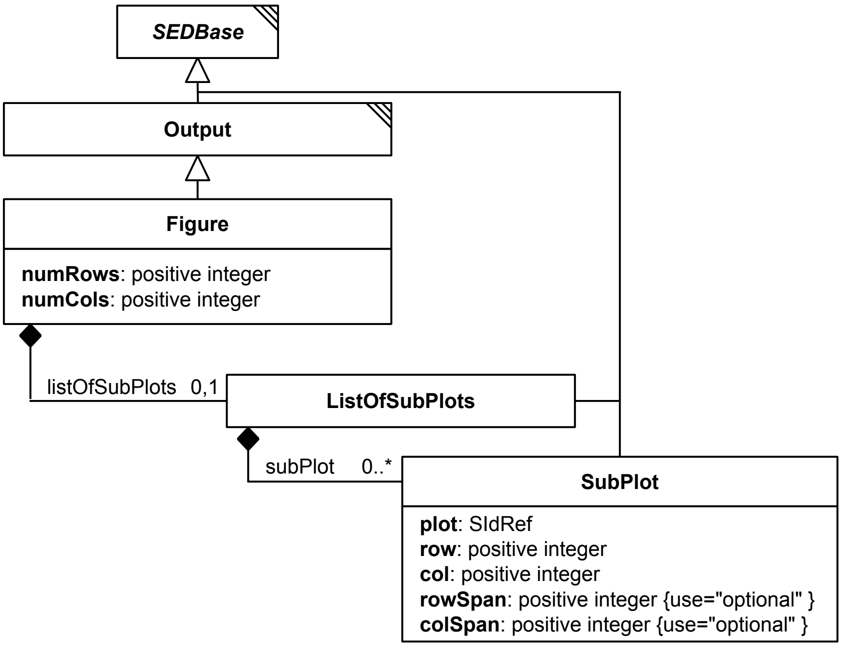

**Figure 2.28:** *The definition of the SED-ML Figure, ListOfSubPlots, and SubPlot classes.*

*`numRows` and `numCols`*

The `numRows` and `numCols` attributes define the number of rows and columns, respectively, to be contained in the figure. The relative size of each row and columns is not defined, but should be large enough to contain the *Plot* elements to be displayed in them.

*`listOfSubPlots`*

The `listOfSubPlots` child of a Figure contains all the *Plot* elements to display. Each SubPlot declares itself where it is to be displayed in the Figure.

##### 2.2.15.1 `SubPlot`

The SubPlot class inherits from *SEDBase* and additionally defines three required attributes (`plot`, of type SIdRef, and `row` and `col`, both of type `positive integer`), and two optional attributes (`rowSpan` and `colSpan`, both of type `positive integer`). Each SubPlot defines where in the Figure the referenced *Plot* should be displayed.

*`plot`*

The `plot` attribute must be an SIdRef to a *Plot*. The referenced *Plot* will be displayed in the Figure. It is not necessary for each `plot` to be unique, if the same *Plot* should be displayed multiple times.

*`row` and `col`*

The `row` and `col` attributes define the row and column, respectively, within the Figure where the *Plot* is to be displayed. This must not conflict with any other SubPlot in the same Figure, and may not be greater than the Figure's `numRows` or `numCols` attributes, respectively. Rows and columns are both numbered starting with "1", rows are ordered top to bottom, and columns are ordered left to right, so `row=''1'' col=''1''` places a *Plot* in the upper left corner of the Figure.

*`rowSpan` and `colSpan`*

The optional `rowSpan` and `colSpan` attributes are used when a *Plot* is to be displayed in multiple rows and/or columns in a Figure. Each attribute indicates the number of rows and/or columns the figure is to span. The value must be a positive integer, and it must not be greater than the number of available rows and/or columns in the Figure.

In the following example, a 3x3 Figure is defined with four subplots. The first is in the upper left corner, the second in the top row occupying columns 2 and 3, the next a 2x2 subplot in the lower left, and the final subplot in the right-most column, occupying rows 2 and 3.

```
<figure id="fig1" name="Figure 1" numRows="3" numCols="3">
    <listOfSubPlots>
        <subPlot id="ax1" plot="plot_y1" row="1" col="1" />
        <subPlot id="ax2" plot="plot_y2" row="1" col="2" colSpan="2"/>
        <subPlot id="ax3" plot="plot_y3" row="2" col="1" colSpan="2" rowSpan="2"/>
        <subPlot id="ax4" plot="plot_y4" row="2" col="3" rowSpan="2"/>
    </listOfSubPlots>
    <notes><p xmlns="xhtml">Figure 1 - Example for figure with text legend and sub-plots.</p></notes>
</figure>
```

**Listing 2.60:** *The SED-ML* **`figure`** *element, defining a figure with four subplots of different sizes*

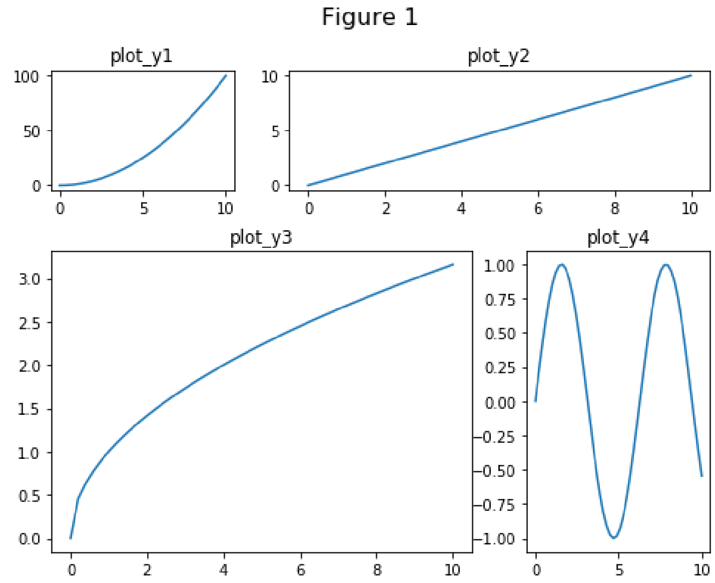

**Figure 2.29:** *The output of Listing 2.60*

#### 2.2.16 Style

The Style class (Figure 2.30 on the next page) defines a graphical style for use in Figure or *Plot* elements.

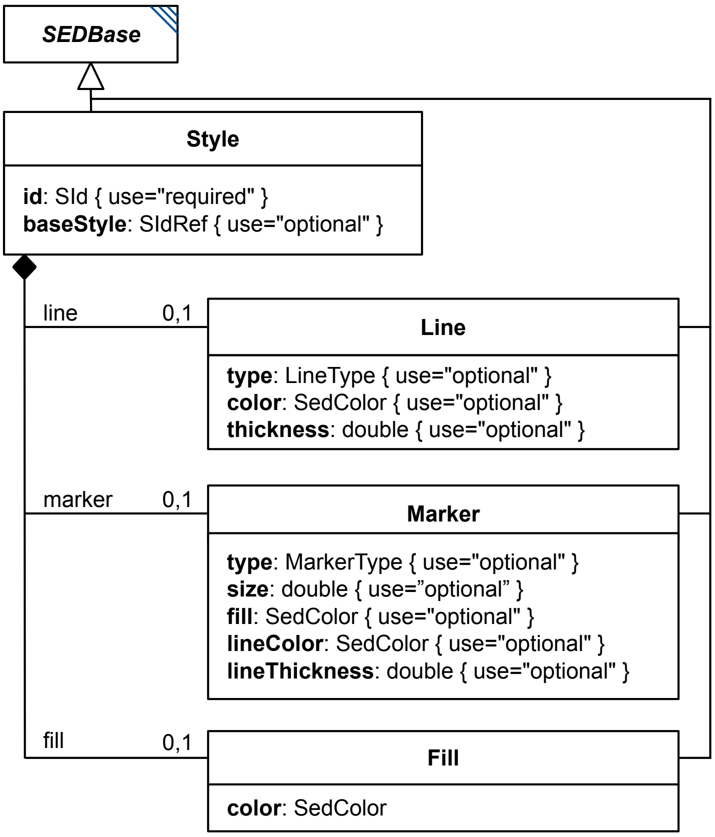

**Figure 2.30:** *The SED-ML Style class*

The Style class inherits the attributes and children from *SEDBase*, extending the **id** attribute to be required, adding an optional **baseStyle** of type SIdRef, and allowing up to three optional chidren of type Line, Marker, and Fill. Collectively, these elements describe a visual style that can be applied to an *AbstractCurve* or Surface.

*baseStyle*

The optional **baseStyle** attribute of data type SIdRef is used to reference a different Style in the same SED-ML Document. If present, any defined aspect of the referenced Style is assumed to apply to the current Style, unless superseded by an element of the current Style. For example, if one Style "**style1**" defines a black line with a blue marker, and a second Style "**style2**" has a **baseStyle** of "**style1**" and defines a red line, applying a "**style2**" would result in a red line with a blue marker.

##### 2.2.16.1 Line

The Line class inherits the attributes and children of *SEDBase*, and adds three optional attributes: **type** of type LineType, **color** of type SedColor, and **thickness** of type **double**. If any of these attributes are defined, lines presented in the parent Style should have that type, color, and/or thickness. If any of the attributes is not defined, it can be defined by the Style referenced in the **baseStyle**, or is undefined and can be anything.

*type*

The **type** attribute defines how lines are to be drawn. The options are:

- **none**: The line is not to be displayed at all.
- **solid**: The line is to be displayed as a continuous line.
- **dash**: The line is to be displayed as a series of short lines.
- **dot**: The line is to be displayed as a series of dots.

- **dashDot**: The line is to be displayed as a series of single lines and single dot combinations.
- **dashDotDot**: The line is to be displayed as a series of single lines and two dot combinations.

*color*

The `color` attribute defines what color the line should be. See the SedColor for a description of how colors are defined in SED-ML.

*thickness*

The `thickness` attribute defines the thickness of the line, in pixels (or the equivalent in the application's display environment).

##### 2.2.16.2 Marker

The Marker class inherits the attributes and children of *SEDBase*, and adds five optional attributes: `type` of type MarkerType, `size` of type `double`, `fill` of type SedColor, `lineColor` of type SedColor, and `lineThickness` of type `double`. If any of these attributes are defined, markers presented in the parent Style should have that attribute. If any of the attributes is not defined, it can be defined by the Style referenced in the `baseStyle`, or is undefined and can be anything.

*type*

The `type` attribute defines how markers are to be drawn. The options are:

- **none**: The marker is not to be displayed at all.
- **square**: The marker is to be displayed as a square.
- **circle**: The marker is to be displayed as a circle.
- **diamond**: The marker is to be displayed as a diamond.
- **xCross**: The marker is to be displayed as an 'x'.
- **plus**: The marker is to be displayed as a plus.
- **star**: The marker is to be displayed as a star.
- **triangleUp**: The marker is to be displayed as an upwards-pointing triangle.
- **triangleDown**: The marker is to be displayed as a downwards-pointing triangle.
- **triangleLeft**: The marker is to be displayed as a left-pointing triangle.
- **triangleRight**: The marker is to be displayed as a right-pointing triangle.
- **hDash**: The marker is to be displayed as a horizontal dash.
- **vDash**: The marker is to be displayed as a vertical dash.

*size*

The `size` attribute defines what size, in pixels, the marker should be (or the equivalent in the application's display environment.

*fill*

The `fill` attribute defines what color the interior of the marker should be. See the SedColor for a description of how colors are defined in SED-ML.

*lineColor*

The `lineColor` attribute defines what color the border of the marker should be. See the SedColor for a description of how colors are defined in SED-ML.

*lineThickness*

The `thickness` attribute defines the thickness of the marker's border, in pixels (or the equivalent in the application's display environment).

##### 2.2.16.3 Fill

The Fill class inherits the attributes and children of *SEDBase*, and adds the optional attributes `color` of type SedColor. When defined, fills presented in the parent Style should have that color. If any of the attributes is not defined, it can be defined by the Style referenced in the `baseStyle`, or is undefined and can be anything.

*color*

The `color` attribute defines what color the fill should be. See the SedColor for a description of how colors are defined in SED-ML.

## 3. Concepts used in SED-ML

### 3.1 MathML

SED-ML encodes mathematical expressions using a subset of MathML 2.0 [5]. MathML is an international standard for encoding mathematical expressions using XML. It is also used as a representation of mathematical expressions in other formats, such as SBML and CellML, two of the model languages supported by SED-ML.

SED-ML files can use mathematical expressions to encode for example pre-processing steps applied to the computational model (ComputeChange), or post processing steps applied to the raw simulation data before output (DataGenerator).

SED-ML classes reference MathML expressions via the element Math of data type `MathML`.

#### 3.1.1 MathML elements

The allowed MathML in SED-ML is restricted to the following subset:

- *token*: `cn`, `ci`, `csymbol`, `sep`
- *general*: `apply`, `piecewise`, `piece`, `otherwise`
- *relational operators*: `eq`, `neq`, `gt`, `lt`, `geq`, `leq`
- *arithmetic operators*: `plus`, `minus`, `times`, `divide`, `power`, `root`, `abs`, `exp`, `ln`, `log`, `floor`, `ceiling`, `factorial`, `quotient`, `max`, `min`, `rem`
- *logical operators*: `and`, `or`, `xor`, `not`, `implies`
- *qualifiers*: `degree`, `logbase`
- *trigonometric operators*: `sin`, `cos`, `tan`, `sec`, `csc`, `cot`, `sinh`, `cosh`, `tanh`, `sech`, `csch`, `coth`, `arcsin`, `arccos`, `arctan`, `arcsec`, `arccsc`, `arccot`, `arcsinh`, `arccosh`, `arctanh`, `arcsech`, `arccsch`, `arccoth`
- *constants*: `true`, `false`, `notanumber`, `pi`, `infinity`, `exponentiale`
- *MathML annotations*: `semantics`, `annotation`, `annotation-xml`

#### 3.1.2 MathML symbols

All the operations listed above describe functions of scalar-valued SED variables, or element-wise computations of matrix-valued SED variables. Matrix-valued SED variables can arise in multiple ways. For example, a variable for a basic task of a non-spatial UniformTimeCourse would be a vector with length equal to the number of steps of the time course plus one. A Variable for a RepeatedTask of a non-spatial time course could be represented a matrix with dimensions for the iterations of the repeated tasks, its subtasks, and the steps of the nested basic task. MathML functions for matrices should be evaluated on an element-wise basis. For example, if $M$ and $N$ were two 2D matrix-valued SED variables, $M + 3$ would add three to every element of $M$, $R = M + N$ would only be valid if $M$ and $N$ have the same dimensions, and $R_{i,j}$ would be equal to $M_{i,j} + N_{i,j}$. If the lengths of the dimensions are not equal (i.e. if $M_{i,j}$ exists but $N_{i,j}$ does not), the missing value should be assumed to be $NaN$ (not a number). At this point, SED-ML does not define an algebra for matrix computations.

##### 3.1.2.1 MathML csymbols for dimensional input

While the new `dimensionTerm` attribute of the Variable class provides functionality to reduce the dimensionality of matrices, previous version of SED-ML defined the MathML functions min, max, sum, and product, each of which would reduce any n-dimensional vector to a single scalar value. It is recommended that users switch to using Variable elements with a `dimensionTerm` for their increased functionality, but the old functions are still defined here for backwards compatibility. The only allowed symbols to be used in aggregate functions are the identifiers of Variables defined in the `listOfVariables` of a DataGenerator. These Variables represent the data collected from the simulation experiment in the associated Task. They always return scalar values, regardless of the dimensionality of the Variable, and ignore any NaN values the vector or matrix might have.

*min*

The `min` of a variable represents the smallest value the simulation experiment for that variable (Listing 3.1).

```
<apply>
    <csymbol encoding="text" definitionURL="http://sed-ml.org/#min">
        min
    </csymbol>
    <ci> variableId </ci>
</apply>
```

**Listing 3.1:** *Example for the use of the MathML* `min` *function.*

*max*

The `max` of a variable represents the largest value the simulation experiment for that variable (Listing 3.2).

```
<apply>
    <csymbol encoding="text" definitionURL="http://sed-ml.org/#max">
        max
    </csymbol>
    <ci> variableId </ci>
</apply>
```

**Listing 3.2:** *Example for the use of the MathML* `max` *function.*

*sum*

The `sum` of a variable represents the sum of all values of the variable returned by the simulation experiment (Listing 3.3).

```
<apply>
    <csymbol encoding="text" definitionURL="http://sed-ml.org/#sum">
        sum
    </csymbol>
    <ci> variableId </ci>
</apply>
```

**Listing 3.3:** *Example for the use of the MathML* `sum` *function.*

*product*

The `product` of a variable represents the multiplication of all values of the variable returned by the simulation experiment (Listing 3.4).

```
<apply>
    <csymbol encoding="text" definitionURL="http://sed-ml.org/#product">
        product
    </csymbol>
    <ci> variableId </ci>
</apply>
```

**Listing 3.4:** *Example for the use of the MathML* `product` *function.*

##### 3.1.2.2 MathML Distribution Functions

The following functions are added to MathML as csymbols to represent draws from distributions: uniform, normal, lognormal, poisson, and gamma:

*uniform*

The uniform of a variable represents a draw from a uniform distribution. It has two arguments: the first is 'min' and the second is 'max', with 'max' requried to be greater than 'min'. The draw from the distribution must be between 'min' and 'max', and may include 'min', but may not include 'max'.

```
<apply>
    <csymbol encoding="text" definitionURL="http://sed-ml.org/functions/#uniform">
        uniform
    </csymbol>
    <ci> minId </ci>
    <ci> maxId </ci>
</apply>
```

**Listing 3.5:** *Example for the use of the MathML* `uniform` *function.*

##### `normal`

The normal of a variable represents a draw from a normal distribution. It has two arguments: the first is 'mean', and the second is 'stdev', that define the mean and the standard deviation, respectively, of the distribution.

```
<apply>
    <csymbol encoding="text" definitionURL="http://sed-ml.org/functions/#normal">
        normal
    </csymbol>
    <ci> meanId </ci>
    <ci> stdevId </ci>
</apply>
```

**Listing 3.6:** *Example for the use of the MathML* `normal` *function.*

##### `lognormal`

The lognormal of a variable represents a draw from a log-normal distribution. It has two arguments: the first is 'mean', and the second is 'stdev', that define the mean and the standard deviation, respectively, of the distribution.

```
<apply>
    <csymbol encoding="text" definitionURL="http://sed-ml.org/functions/#lognormal">
        lognormal
    </csymbol>
    <ci> meanId </ci>
    <ci> stdevId </ci>
</apply>
```

**Listing 3.7:** *Example for the use of the MathML* `lognormal` *function.*

##### `gamma`

The gamma of a variable represents a draw from a gamma distribution. It has two arguments: the first is 'shape', and the second is 'scale', that define the shape and scale, respectively, of the distribution.

```
<apply>
    <csymbol encoding="text" definitionURL="http://sed-ml.org/functions/#gamma">
        gamma
    </csymbol>
    <ci> shapeId </ci>
    <ci> scaleId </ci>
</apply>
```

**Listing 3.8:** *Example for the use of the MathML* `gamma` *function.*

##### `poisson`

The poisson of a variable represents a discrete value drawn from a poisson distribution. It has a single argument: 'rate', the expected rate of occurrences for the distribution.

```
<apply>
    <csymbol encoding="text" definitionURL="http://sed-ml.org/functions/#poisson">
        poisson
    </csymbol>
    <ci> rateId </ci>
</apply>
```

**Listing 3.9:** *Example for the use of the MathML* `poisson` *function.*

#### 3.1.3 NA values

NA (not available) values can occur within a simulation experiment. Examples are missing values in a DataSource or simulation results with NA values. All math operations encoded in MathML in SED-ML are well defined on NA values.

NA values in a Curve or Surface should be ignored during plotting.

### 3.2 URI scheme

URIs are used in SED-ML as a mechanism

- to reference models (3.2.1 Model references)
- to reference data files (3.2.2 Data references)
- to enable addressing implicit model variables (3.2.3 Symbols)
- to annotate SED-ML elements (3.2.4 Annotation Scheme)

#### 3.2.1 Model references

The two principle recommended methods for referencing data is by URL or by relative pathname. Any URL should preferably point to a public, consistent location that provides the model description file. References to curated, open model bases are recommended, such as the BioModels Database. Relative pathnames are useful both when working with a collection or folder of related files, or when the files are collected into a COMBINE archive.

For additional information see the `source` attribute on Model.

An alternative means to obtain a model may be to provide a single resource containing necessary models and a SED-ML file. Although a specification of such a resource is beyond the scope of this document, the recommended means is the COMBINE archive.

#### 3.2.2 Data references

The two principle recommended methods for referencing data is by URL or by relative pathname. Both of these methods will work if the file or files are transferred to a new location, or to a COMBINE archive. Absolute pathnames will work when used in their original locations, but not when moved to a new location or bundled into an archive, and are therefore not recommended.

For additional information see the `source` attribute on DataDescription.

#### 3.2.3 Symbols

Some variables used in a simulation experiment are not explicitly defined in the model, but may be implicitly contained in it. For example, to plot a variable's behaviour over time, that variable is defined in an SBML model, whereas time is not explicitly defined.

SED-ML can refer to such implicit variables via the Symbol concept. Such implicit variables are defined using KiSAO through the `kisaoID` format to reference the implied variable.

For example, to refer in a SED-ML file to the definition of time, the string `KISAO:0000832` is used. For backwards compatibility, the string "`urn:sedml:symbol:time`" may be used.

With very few exceptions, symbols refer to mathematics of a model that can be read out of the model, but cannot be set directly. You cannot use a `symbol` attribute to set the time of a model, for example, nor may you set the Stoichiometry matrix nor the elasticities. The only partial exception to this is that the amount, concentation, or particle number of a species may be set by an element using both a `target` attribute to indicate the species and a `symbol` to indicate which form to use.

Table 3.1 lists the predefined symbols in SED-ML.

| Language | URN | KiSAO ID | Definition |
|---|---|---|---|
| SBML | `urn:sedml:symbol:time` | KISAO:0000832 | Time in SBML is an intrinsic model variable that is addressable in model equations via a csymbol `time`. |

**Table 3.1:** *The single predefined symbol in SED-ML. For Level 1 Version 5, KiSAO IDs are used instead, though 'time' is still allowed for backwards compatibility. The latest list of KiSAO terms is available from* `https://github.com/SED-ML/KiSAO`.

#### 3.2.4 Annotation Scheme

When annotating SED-ML elements with semantic annotations, the MIRIAM URI Scheme should be used. In addition to providing the data type (e.g., PubMed) and the particular data entry inside that data type (e.g., `10415827`), the relation of the annotation to the annotated element should be described using the standardized biomodels.net qualifier. The list of qualifiers, as well as further information about their usage, is available from `http://www.biomodels.net/qualifiers/`.

### 3.3 URN scheme

URNs are a subset of URIs, and are used in SED-ML as a mechanism

- to specify the language of the referenced model (3.3.1 Language references)
- to specify the format of the referenced dataset (3.3.2 Data format references)

#### 3.3.1 Language references

The evaluation of a SED-ML document is required in order for software to decide whether or not it can be used in a particular simulation environment. One crucial criterion is the particular model representation language used to encode the model. A simulation software usually only supports a small subset of the representation formats available to model biological systems computationally.

To help software decide whether or not it supports a SED-ML description file, the information on the model encoding for each referenced model can be provided through the `language` attribute, as the description of a language name and version through an unrestricted `String` is error-prone. A prerequisite for a language to be fully supported by SED-ML is that a formalised language definition, e.g., an XML Schema, is provided online. SED-ML also defines a set of standard URIs to refer to particular language definitions.

To specify the language a model is encoded in, a set of pre-defined SED-ML URNs can be used (Table 3.2 on the next page). The structure of SED-ML language URNs is `urn:sedml:language:`*`name.version`*. One can be as specific as defining a model being in a particular version of a language, e.g., SBML Level 3 Version 1 as `urn:sedml:language:sbml.level-3.version-1`.

For additional information see the `language` attribute on Model.

#### 3.3.2 Data format references

To help software decide whether or not it supports a SED-ML file, the information on the dataDescription encoding for each referenced dataDescription can be provided through the `format` attribute.

To specify the format of a dataDescription, a set of pre-defined SED-ML URNs can be used (Table 3.3 on the following page). The structure of SED-ML format URNs is `urn:sedml:format:`*`name.version`*.

If it is not explicitly defined the default value for `format` is `urn:sedml:format:numl`, referring to NuML representation of the data. However, the use of the `format` attribute is strongly encouraged.

For additional information see the `format` attribute on DataDescription and the description of individual formats and their use in SED-ML below.

##### *3.3.2.1 NuML (Numerical Markup Language)*

NuML is an exchange format for numerical data. Data in the NuML format (`urn:sedml:format:numl`) is defined via `resultComponents` with a single dataset corresponding to a single `resultComponent`. In the case that a NuML file consists of multiple `resultComponents` the first `resultComponent` contains the data used in the DataDescription. There is currently no mechanism in SED-ML to reference the additional `resultComponents`.

If a `dimensionDescription` is set on the DataDescription, than this `dimensionDescription` must be identical to the `dimensionDescription` of the NuML file.

| Language | URN |
|---|---|
| BNGL (generic) | `urn:sedml:language:bngl` |
| CellML (generic) | `urn:sedml:language:cellml` |
| CellML 1.0 | `urn:sedml:language:cellml.1_0` |
| CellML 1.1 | `urn:sedml:language:cellml.1_1` |
| CellML 2.0 | `urn:sedml:language:cellml.2_0` |
| GINML (generic) | `urn:sedml:language:ginml` |
| HOC (generic) | `urn:sedml:language:hoc` |
| Kappa (generic) | `urn:sedml:language:kappa` |
| LEMS (generic) | `urn:sedml:language:lems` |
| MorpheusML (generic) | `urn:sedml:language:morpheusml` |
| NeuroML (generic) | `urn:sedml:language:neuroml` |
| NeuroML Version 1.8.1 Level 1 | `urn:sedml:language:neuroml.version-1_8_1.level-1` |
| NeuroML Version 1.8.1 Level 2 | `urn:sedml:language:neuroml.version-1_8_1.level-2` |
| NeuroML Version 1.8.1 Level 3 | `urn:sedml:language:neuroml.version-1_8_1.level-3` |
| NeuroML Version 2.1 | `urn:sedml:language:neuroml.version-2_1` |
| PharmML (generic) | `urn:sedml:language:pharmml` |
| SBML (generic) | `urn:sedml:language:sbml` |
| SBML Level 1 Version 1 | `urn:sedml:language:sbml.level-1.version-1` |
| SBML Level 1 Version 2 | `urn:sedml:language:sbml.level-1.version-2` |
| SBML Level 2 Version 1 | `urn:sedml:language:sbml.level-2.version-1` |
| SBML Level 2 Version 2 | `urn:sedml:language:sbml.level-2.version-2` |
| SBML Level 2 Version 3 | `urn:sedml:language:sbml.level-2.version-3` |
| SBML Level 2 Version 4 | `urn:sedml:language:sbml.level-2.version-4` |
| SBML Level 2 Version 5 | `urn:sedml:language:sbml.level-2.version-5` |
| SBML Level 3 Version 1 | `urn:sedml:language:sbml.level-3.version-1` |
| SBML Level 3 Version 2 | `urn:sedml:language:sbml.level-3.version-2` |
| Smoldyn (generic) | `urn:sedml:language:smoldyn` |
| VCML (generic) | `urn:sedml:language:vcml` |
| ZGINML (generic) | `urn:sedml:language:zginml` |

**Table 3.2:** *Predefined model language URNs. The latest list of language URNs is available from* *https://sed-ml.org/urns.html*.

| Data Format | URN |
|---|---|
| NuML (generic) | `urn:sedml:format:numl` |
| NuML Level 1 Version 1 | `urn:sedml:format:numl.level-1.version-1` |
| CSV | `urn:sedml:format:csv` |
| TSV | `urn:sedml:format:tsv` |
| HDF5 | `urn:sedml:format:hdf5` |

**Table 3.3:** *Predefined dataDescription format URNs. The latest list of format URNs is available from* *https://sed-ml.org/urns.html*.

##### 3.3.2.2 CSV (Comma Separated Values)

Data in the CSV format (`urn:sedml:format:csv`) must follow the following rules when used in combination with SED-ML:

- Each record is one line - Line separator may be LF (0x0A) or CRLF (0x0D0A), a line separator may also be embedded in the data (making a record more than one line but still acceptable).
- Fields are separated with commas.
- Embedded commas - Field must be delimited with double-quotes.
- Leading and trailing whitespace is ignored - Unless the field is delimited with double-quotes in that case the whitespace is preserved.

- Embedded double-quotes - Embedded double-quote characters must be doubled, and the field must be delimited with double-quotes.
- Embedded line-breaks - Fields must be surounded by double-quotes.
- Always Delimiting - Fields may always be delimited with double quotes, the delimiters will be parsed and discarded by the reading applications.
- The first record is the header record defining the unique column ids
- Lines starting with "#" are treated as comment lines and ignored
- Empty lines are allowed and ignored
- For numerical data the "." decimal separator is used
- The following strings are interpreted as NaN: "", "#N/A", "#N/A N/A", "#NA", "-1.#IND", "-1.#QNAN", "-NaN", "-nan", "1.#IND", "1.#QNAN", "N/A", "NA", "NULL", "NaN", "nan".

A dataset in CSV is always encoding two dimensional data.

When using data in the CSV format SED-ML, the `dimensionDescription` is required on the DataDescription.

The `dimensionDescription` must consist of an outer `compositeDescription` with `indexType="integer"` which allows to reference the rows of the CSV by index and a inner `compositeDescription` which allows to reference the columns of the CSV by their column header id. Within the inner `compositeDescription` exactly one `atomicDescription` must exist. All data in the CSV must have the same type which is defined via the `valueType` on the `atomicDescription`.

Below an example of the required `dimensionDescription` for a CSV is provided. In the example the `time` and `S1` columns are read from the CSV file

```
# ./example.csv
time, S1, S2
0.0, 10.0, 0.0
0.1, 9.9, 0.1
0.2, 9.8, 0.2
```

**Listing 3.10:** *Example CSV*

```

<dataDescription id="datacsv" name="Example CSV dataset" source="./example.csv" format="urn:sedml:format:csv">
  <dimensionDescription>
    <compositeDescription indexType="integer" name="Index">
      <compositeDescription indexType="string" name="ColumnIds">
        <atomicDescription valueType="double" name="Values" />
      </compositeDescription>
    </compositeDescription>
  </dimensionDescription>
  <listOfDataSources>
    <dataSource id="dataTime">
      <listOfSlices>
        <slice reference="ColumnIds" value="time" />
      </listOfSlices>
    </dataSource>
    <dataSource id="dataS1">
      <listOfSlices>
        <slice reference="ColumnIds" value="S1" />
      </listOfSlices>
    </dataSource>
  </listOfDataSources>
  ...
</dataDescription>
```

**Listing 3.11:** *SED-ML `dimensionDescription` element for the example.csv*

##### 3.3.2.3 TSV (Tab Separated Values)

The format TSV (`urn:sedml:format:tsv`) is defined identical to CSV with the exceptions listed below

- Fields are separated with tabs instead of commas.
- Embedded tab - Field must be delimited with double-quotes (embedded comma field must not be delimited with double quotes)

##### 3.3.2.4 HDF5 (Hierarchical Data Format version 5)

The format HDF5 is defined at https://portal.hdfgroup.org/display/HDF5/HDF5. It supports the storage of multidimensional data, and is therefore ideal for storing the SED-ML output of repeated tasks; particularly nested repeated tasks. It also supports external storage of data, making it appropriate for extremely large data sets, such as that output by spatial simulations.

Each dimension of SED-ML RepeatedTask output should be labeled according to the relevant `id` of the SED-ML object that describes that dimension, namely:

- The `id` of the top-level RepeatedTask
- The `id` of the SubTask
- The `id` of any nested SubTask (for arbitrarily-deeply nested subtasks).
- The dimension of the data itself (i.e. `time` for a UniformTimeCourse).
- The `id` of the requested variable, or the infix representation of the Math from the DataGenerator.

When a Variable's `dimensionTerm` is used to reduce the dimensionality of a set of data (using an appropriate KiSAO value and AppliedDimension children), information about the dimension reduction may be included as annotation, i.e. one could annotate a SubTask dimension as 'averaged over the RepeatedTask `[id]`'. When a DataGenerator contains a Variable that outputs a matrix, that matrix can also be labeled appropriately (such as with species or reaction ids).

When output from multiple tasks are combined mathematically, their dimensions must match exactly, so the ids from either (or a combination of both) may be used. Again, annotations are recommended to describe how the data was combined.

Each dimension may also be annotated with an ontology term such as one from the 'Semanticscience Integrated Ontology' (SIO, https://bioportal.bioontology.org/ontologies/SIO).

### 3.4 XPath

XPath is a language for finding and referencing information in an XML document [7]. Within SED-ML Level 1 Version 5, XPath version 1 expressions can be used to identify nodes and attributes within an XML representation of an XML-encoded model in the following ways:

- Within a Variable definition, where XPath identifies the model variable required for manipulation in SED-ML. In this context, the XPath must always reference a single XML element, and not an attribute nor multiple XML elements.
- Within a Change definition, where XPath is used to identify the target XML to which a change should be applied. In this context, the XPath may point to anything in the XML as appropriate for the Change (i.e. an attribute in a ChangeAttribute; one or more elements or attributes to remove in a RemoveXML, etc.).

For proper application, XPath expressions should contain prefixes that allow their resolution to the correct XML namespace within an XML document. For example, the XPath expression referring to a species $X$ in an SBML model:

`/sbml:sbml/sbml:model/sbml:listOfSpecies/sbml:species[@id='X']` ✔ *-CORRECT*

is preferable to

`/sbml/model/listOfSpecies/species[@id='X']` ✘ *-INCORRECT*

which will only be interpretable by standard XML software tools if the SBML file declares no namespaces (and hence is invalid SBML).

Following the convention of other XPath host languages such as XPointer and XSLT, the prefixes used within XPath expressions must be declared using namespace declarations within the SED-ML document, and be in-scope for the relevant expression. Thus for the correct example above, there must also be an ancestor element of the node containing the ssion that has an attribute like:

`xmlns:sbml='http://www.sbml.org/sbml/level3/version1/core'`

(a different namespace URI may be used; the key point is that the prefix 'sbml' must match that used in the XPath expression).

### 3.5 NuML

The Numerical Markup Language (NuML) aims to standardize the exchange and archiving of numerical results. Additional information including the NuML specification is available from `https://github.com/NuML/NuML`.

NuML constructs are used in SED-ML for referencing external data sets in the DataDescription class. NuML is used to define the DimensionDescription of external datasets in the DataDescription. In addition, `NuMLSIds` are used for retrieving subsets of data via either the `indexSet` element in the DataSource or within the Slice class.

### 3.6 KiSAO

The Kinetic Simulation Algorithm Ontology (KiSAO [8]) is used in SED-ML to specify simulation algorithms and algorithmParameters. KiSAO is a community-driven approach of classifying and structuring simulation approaches by model characteristics and numerical characteristics. The ontology is available in OWL format from BioPortal at `https://purl.bioontology.org/ontology/KiSAO`.

Defining simulation algorithms through KISAO terms not only identifies the simulation algorithm used for the SED-ML simulation, it also enables software to find related algorithms, if the specific implementation is not available. For example, software could decide to use the CVODE integration library for an analysis instead of a specific Runge Kutta 4,5 implementation.

Should a particular simulation algorithm or algorithm parameter not exist in KiSAO, please request one via `https://github.com/SED-ML/KiSAO/issues/new/choose`.

### 3.7 COMBINE archive

A COMBINE archive [1] is a single file that supports the exchange of all the information necessary for a modeling and simulation experiment in biology. A COMBINE archive file is a ZIP container that includes a manifest file, listing the content of the archive, an optional metadata file adding information about the archive and its content, and the files describing the model. The content of a COMBINE archive consists of files encoded in COMBINE standards whenever possible, but may include additional files defined by an Internet Media Type. Several tools that support the COMBINE archive are available, either as independent libraries or embedded in modeling software.

The COMBINE archive is described at `https://co.mbine.org/documents/archive` and in [1].

COMBINE archives are the recommended means for distributing simulation experiment descriptions in SED-ML, the respective data and model files, and the Outputs of the simulation experiment (figures and reports). All SED-ML specification examples in Appendix A are available as COMBINE archive from `https://sed-ml.org`.

### 3.8 SED-ML resources

Information on SED-ML can be found on `https://sed-ml.org`. The SED-ML XML Schema, the UML schema, SED-ML examples, and additional information is available from `https://github.com/sed-ml`.

## 4. Acknowledgements

The SED-ML specification is developed with the input of many people. The following individuals have contributed to the SED-ML specifications.

- Richard Adams (Editor, 2011-2012)
- Eran Agmon (Editor, 2023-2025)
- Frank Bergmann (Editor, 2011-2014, 2020-2022)
- Jonathan Cooper (Editor, 2012-2015)
- Alan Garny (Editor, 2018-2020)
- Tomáš Helikar (Editor, 2021-2023)
- Jonathan Karr (Editor, 2021-2023)
- Matthias König (Editor, 2017-2019, 2020-2022)
- David Nickerson (Editor, 2011-2013, 2015-2017, 2019-2021, 2023-2025)
- Nicolas Le Novère (editorial advisor, 2011-2012, 2013)
- Brett Olivier (Editor, 2015-2017)
- Andrew Miller (Editor, 2011-2012)
- Ion Moraru (Editor, 2014-2016)
- Sven Sahle (Editor, 2014-2016)
- Herbert Sauro (Editor, 2018-2020)
- Lucian Smith (Editor, 2016-2018, 2022-2024)
- Dagmar Waltemath (Editor, 2011-2014, 2017-2019)

The most recent funding for this effort includes:

- National Institute for Biomedical Imaging and Bioengineering award P41GM109824. (LS, HS, DN, JK)
- National Institute of General Medical Sciences award R01GM123032. (LS, HS)
- National Science Foundation award 1933453 (LS, HS)
- National Institute of General Medical Sciences, grant R35GM119771 (JK)
- Federal Ministry of Education and Research (BMBF, Germany) within the research network de.NBI (grant number 031L0104A) (FB)
- National Institute of Health grant number R35GM119770 (TH)

- The Federal Ministry of Education and Research (BMBF, Germany) within the research network Systems Medicine of the Liver (grant number 031L0054) and within ATLAS by grant number 031L0304B; by the German Research Foundation (DFG) within the Research Unit Program FOR 5151 QuaLiPerF by grant number 436883643 and by grant number 465194077 (Priority Programme SPP 2311, Subproject SimLivA). (MK)

In addition, we thank the many members of the SED-ML coummunity who have contributed to the development of SED-ML.

## A. Examples

This appendix presents several SED-ML examples. Complete versions of these and additional examples are available as Combine archives at https://sed-ml.org/. These examples illustrate the main features of SED-ML. Please note, these examples do not demonstrate the full capabilities of SED-ML. The specifications of SED-ML (Chapter 2) provide a more comprehensive view of the simulation experiments that can be captured with SED-ML.

The examples presented here involved models encoded in CellML and SBML. Please note, SED-ML can be used with additional model languages. See Section 3.3.1 for more information about using SED-ML with additional model languages. Example SED-ML files for additional model languages are available at https://run.biosimulations.org/.

### A.1 Example simulation experiment (L1V3_repressilator.omex)

This example lists the SED-ML for the example in the introduction (Section 1.2). It illustrates the use of a `dimensionTerm` to calculate the maximum value of a vector with the KiSAO term for 'maximum' ("`KISAO:0000828`") as the `term`. This document can be found at https://sed-ml.org/examples/L1V4/L1V4_repressilator/repressilator.xml, and an OMEX version at https://sed-ml.org/examples/L1V4/L1V4_repressilator.omex.

```
<?xml version="1.0" encoding="UTF-8"?>
<!-- Created by phraSED-ML version v1.0.7 with libSBML version 5.15.0. -->
<sedML xmlns="http://sed-ml.org/sed-ml/level1/version5" level="1" version="5">
  <listOfSimulations>
    <uniformTimeCourse id="sim1" initialTime="0" outputStartTime="0" outputEndTime="1000" numberOfSteps="
        1000">
      <algorithm kisaoID="KISAO:0000019"/>
    </uniformTimeCourse>
  </listOfSimulations>
  <listOfModels>
    <model id="model1" language="urn:sedml:language:sbml.level-3.version-1" source="https://www.ebi.ac.uk
        /biomodels/model/download/BIOMD0000000012?filename=BIOMD0000000012_url.xml"/>
    <model id="model2" language="urn:sedml:language:sbml.level-3.version-1" source="#model1">
      <listOfChanges>
        <changeAttribute target="/sbml:sbml/sbml:model/sbml:listOfParameters/sbml:parameter[@id='ps_0']/
            @value" newValue="1.3e-05"/>
        <changeAttribute target="/sbml:sbml/sbml:model/sbml:listOfParameters/sbml:parameter[@id='ps_a']/
            @value" newValue="0.013"/>
      </listOfChanges>
    </model>
  </listOfModels>
  <listOfTasks>
    <task id="task1" modelReference="model1" simulationReference="sim1"/>
    <task id="task2" modelReference="model2" simulationReference="sim1"/>
  </listOfTasks>
  <listOfDataGenerators>
    <!-- timecourse -->
    <dataGenerator id="dg_0_0_0" name="task1.time">
      <listOfVariables>
        <variable id="task1_____time" symbol="urn:sedml:symbol:time" taskReference="task1"/>
      </listOfVariables>
      <math xmlns="http://www.w3.org/1998/Math/MathML">
        <ci> task1_____time </ci>
      </math>
    </dataGenerator>
    <dataGenerator id="dg_0_0_1" name="PX (lacI)">
      <listOfVariables>
        <variable id="task1_____PX" target="/sbml:sbml/sbml:model/sbml:listOfSpecies/sbml:species[@id='PX
            ']" taskReference="task1" modelReference="model1"/>
      </listOfVariables>
      <math xmlns="http://www.w3.org/1998/Math/MathML">
        <ci> task1_____PX </ci>
      </math>
    </dataGenerator>
```

```
    <dataGenerator id="dg_0_1_1" name="PZ (cI)">
      <listOfVariables>
        <variable id="task1_____PZ" target="/sbml:sbml/sbml:model/sbml:listOfSpecies/sbml:species[@id='PZ']" taskReference="task1" modelReference="model1"/>
      </listOfVariables>
      <math xmlns="http://www.w3.org/1998/Math/MathML">
        <ci> task1_____PZ </ci>
      </math>
    </dataGenerator>
    <dataGenerator id="dg_0_2_1" name="PY (tetR)">
      <listOfVariables>
        <variable id="task1_____PY" target="/sbml:sbml/sbml:model/sbml:listOfSpecies/sbml:species[@id='PY']" taskReference="task1" modelReference="model1"/>
      </listOfVariables>
      <math xmlns="http://www.w3.org/1998/Math/MathML">
        <ci> task1_____PY </ci>
      </math>
    </dataGenerator>
    <!-- pre-processing -->
    <dataGenerator id="dg_1_0_0" name="time">
      <listOfVariables>
        <variable id="task2_____time" symbol="urn:sedml:symbol:time" taskReference="task2"/>
      </listOfVariables>
      <math xmlns="http://www.w3.org/1998/Math/MathML">
        <ci> task2_____time </ci>
      </math>
    </dataGenerator>
    <dataGenerator id="dg_1_0_1" name="PX (lacI)">
      <listOfVariables>
        <variable id="task2_____PX" target="/sbml:sbml/sbml:model/sbml:listOfSpecies/sbml:species[@id='PX']" taskReference="task2" modelReference="model2"/>
      </listOfVariables>
      <math xmlns="http://www.w3.org/1998/Math/MathML">
        <ci> task2_____PX </ci>
      </math>
    </dataGenerator>
    <dataGenerator id="dg_1_1_1" name="PZ (cI)">
      <listOfVariables>
        <variable id="task2_____PZ" target="/sbml:sbml/sbml:model/sbml:listOfSpecies/sbml:species[@id='PZ']" taskReference="task2" modelReference="model2"/>
      </listOfVariables>
      <math xmlns="http://www.w3.org/1998/Math/MathML">
        <ci> task2_____PZ </ci>
      </math>
    </dataGenerator>
    <dataGenerator id="dg_1_2_1" name="PY (tetR)">
      <listOfVariables>
        <variable id="task2_____PY" target="/sbml:sbml/sbml:model/sbml:listOfSpecies/sbml:species[@id='PY']" taskReference="task2" modelReference="model2"/>
      </listOfVariables>
      <math xmlns="http://www.w3.org/1998/Math/MathML">
        <ci> task2_____PY </ci>
      </math>
    </dataGenerator>
    <!-- post-processing -->
    <dataGenerator id="dg_2_0_0" name="PX/max(PX) (lacI normalized)">
      <listOfVariables>
        <variable id="task1_____PX" target="/sbml:sbml/sbml:model/sbml:listOfSpecies/sbml:species[@id='PX']" taskReference="task1" modelReference="model1"/>
        <variable id="task1_____PX_max" target="/sbml:sbml/sbml:model/sbml:listOfSpecies/sbml:species[@id='PX']" taskReference="task1" modelReference="model1" dimensionTerm="KISAO:0000828"/>
      </listOfVariables>
      <math xmlns="http://www.w3.org/1998/Math/MathML">
        <apply>
          <divide/>
          <ci> task1_____PX </ci>
          <ci> task1_____PX_max </ci>
        </apply>
      </math>
    </dataGenerator>
    <dataGenerator id="dg_2_0_1" name="PZ/max(PZ) (cI normalized)">
      <listOfVariables>
        <variable id="task1_____PZ" target="/sbml:sbml/sbml:model/sbml:listOfSpecies/sbml:species[@id='PZ']" taskReference="task1" modelReference="model1"/>
        <variable id="task1_____PZ_max" target="/sbml:sbml/sbml:model/sbml:listOfSpecies/sbml:species[@id='PZ']" taskReference="task1" modelReference="model1" dimensionTerm="KISAO:0000828"/>
      </listOfVariables>
      <math xmlns="http://www.w3.org/1998/Math/MathML">
        <apply>
          <divide/>
          <ci> task1_____PZ </ci>
          <ci> task1_____PZ_max </ci>
        </apply>
      </math>
    </dataGenerator>
    <dataGenerator id="dg_2_1_0" name="PY/max(PY) (tetR normalized)">
      <listOfVariables>
        <variable id="task1_____PY" target="/sbml:sbml/sbml:model/sbml:listOfSpecies/sbml:species[@id='PY']" taskReference="task1" modelReference="model1"/>
```

```
        <variable id="task1_____PY_max" target="/sbml:sbml/sbml:model/sbml:listOfSpecies/sbml:species[@id
            ='PY']" taskReference="task1" modelReference="model1" dimensionTerm="KISAO:0000828"/>
      </listOfVariables>
      <math xmlns="http://www.w3.org/1998/Math/MathML">
        <apply>
          <divide/>
          <ci> task1_____PY </ci>
          <ci> task1_____PY_max </ci>
        </apply>
      </math>
    </dataGenerator>
  </listOfDataGenerators>
  <listOfOutputs>
    <plot2D id="timecourse" name="Timecourse of repressilator">
      <listOfCurves>
        <curve id="plot_0__plot_0_0_0__plot_0_0_1" xDataReference="dg_0_0_0" yDataReference="dg_0_0_1"/>
        <curve id="plot_0__plot_0_0_0__plot_0_1_1" xDataReference="dg_0_0_0" yDataReference="dg_0_1_1"/>
        <curve id="plot_0__plot_0_0_0__plot_0_2_1" xDataReference="dg_0_0_0" yDataReference="dg_0_2_1"/>
      </listOfCurves>
    </plot2D>
    <plot2D id="preprocessing" name="Timecourse after pre-processing">
      <listOfCurves>
        <curve id="plot_1__plot_1_0_0__plot_1_0_1" xDataReference="dg_1_0_0" yDataReference="dg_1_0_1"/>
        <curve id="plot_1__plot_1_0_0__plot_1_1_1" xDataReference="dg_1_0_0" yDataReference="dg_1_1_1"/>
        <curve id="plot_1__plot_1_0_0__plot_1_2_1" xDataReference="dg_1_0_0" yDataReference="dg_1_2_1"/>
      </listOfCurves>
    </plot2D>
    <plot2D id="postprocessing" name="Timecourse after post-processing">
      <listOfCurves>
        <curve id="plot_2__plot_2_0_0__plot_2_0_1" xDataReference="dg_2_0_0" yDataReference="dg_2_0_1"/>
        <curve id="plot_2__plot_2_1_0__plot_2_0_0" xDataReference="dg_2_1_0" yDataReference="dg_2_0_0"/>
        <curve id="plot_2__plot_2_0_1__plot_2_1_0" xDataReference="dg_2_0_1" yDataReference="dg_2_1_0"/>
      </listOfCurves>
    </plot2D>
  </listOfOutputs>
</sedML>
```

**Listing A.1:** *SED-ML document for example simulation experiment.*

### A.2 Simulation experiments with dataDescriptions

The DataDescription provides means to use external datasets in simulation experiments. In this section simulation experiments using the dataDescription are presented.

#### A.2.1 Plotting data with simulations (`L1V3_plotting-data-numl.omex`)

This example demonstrates the use of the DataDescription and DataSource to load external data in SED-ML. In the example a model is simulated (using a uniformTimeCourse simulation) and the simulation results are plotted. In addition data is plotted using the dataDescription and DataSource), extracting the `S1` and `time` column from it and renders it. The listed example uses data encoded in NuML as format (`urn:sedml:format:numl`). This document can be found at `https://sed-ml.org/examples/L1V3/L1V3_plotting-data-numl/plotting-data-numl.xml`, and an OMEX version at `https://sed-ml.org/examples/L1V3/L1V3_plotting-data-numl.omex`.

The corresponding example using CSV (`urn:sedml:format:csv`) as format to encode the data is available as `L1V3_plotting-data-csv.omex`.

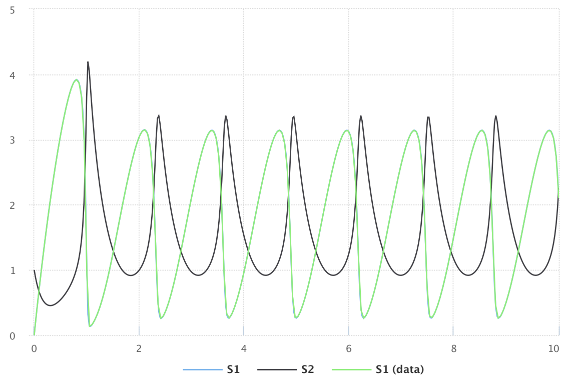

**Figure A.1:** *The simulation result from the simulation description given in Listing A.2. Simulation with SED-ML web tools [2].*

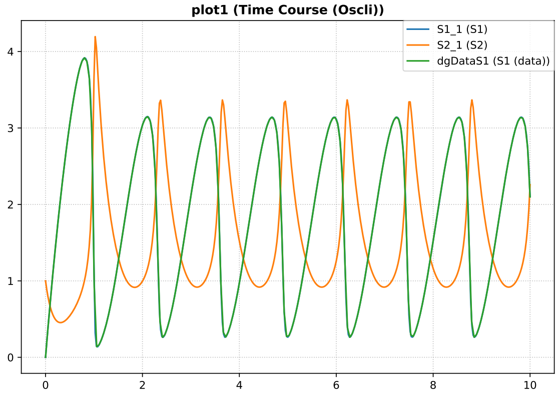

**Figure A.2:** *Simulation with tellurium [6].*

```xml
<?xml version="1.0" encoding="utf-8"?>
<sedML level="1" version="5" xmlns="http://sed-ml.org/sed-ml/level1/version5">
    <listOfDataDescriptions>
        <dataDescription id="Data1" name="oscillator data" source="./oscli.numl" format="urn:sedml:format:numl">
            <dimensionDescription>
                <compositeDescription indexType="double" id="time" name="time" xmlns="http://www.numl.org/numl/level1/version1">
                    <compositeDescription indexType="string" id="SpeciesIds" name="SpeciesIds">
                        <atomicDescription valueType="double" name="Concentrations"/>
                    </compositeDescription>
                </compositeDescription>
            </dimensionDescription>
            <listOfDataSources>
                <dataSource id="dataS1">
                    <listOfSlices>
                        <slice reference="SpeciesIds" value="S1"/>
                    </listOfSlices>
                </dataSource>
                <dataSource id="dataTime" indexSet="time"/>
            </listOfDataSources>
        </dataDescription>
    </listOfDataDescriptions>
    <listOfSimulations>
        <uniformTimeCourse id="sim1" initialTime="0" outputStartTime="0" outputEndTime="10" numberOfSteps="400">
            <algorithm kisaoID="KISAO:0000019">
                <listOfAlgorithmParameters>
                    <algorithmParameter kisaoID="KISAO:0000209" value="1E-06"/>
                    <algorithmParameter kisaoID="KISAO:0000211" value="1E-12"/>
                    <algorithmParameter kisaoID="KISAO:0000415" value="10000"/>
                </listOfAlgorithmParameters>
            </algorithm>
```

```xml
        </uniformTimeCourse>
    </listOfSimulations>
    <listOfModels>
        <model id="model1" language="urn:sedml:language:sbml" source="./oscli.xml"/>
    </listOfModels>
    <listOfTasks>
        <task id="task1" modelReference="model1" simulationReference="sim1"/>
    </listOfTasks>
    <listOfDataGenerators>
        <dataGenerator id="time_1" name="time">
            <listOfVariables>
                <variable id="time" name="time" taskReference="task1" symbol="urn:sedml:symbol:time"/>
            </listOfVariables>
            <math xmlns="http://www.w3.org/1998/Math/MathML">
                <ci>time</ci>
            </math>
        </dataGenerator>
        <dataGenerator id="S1_1" name="S1">
            <listOfVariables>
                <variable id="S1" name="S1" taskReference="task1"
                          target="/sbml:sbml/sbml:model/sbml:listOfSpecies/sbml:species[@id='S1']"/>
            </listOfVariables>
            <math xmlns="http://www.w3.org/1998/Math/MathML">
                <ci>S1</ci>
            </math>
        </dataGenerator>
        <dataGenerator id="S2_1" name="S2">
            <listOfVariables>
                <variable id="S2" name="S2" taskReference="task1"
                          target="/sbml:sbml/sbml:model/sbml:listOfSpecies/sbml:species[@id='S2']"/>
            </listOfVariables>
            <math xmlns="http://www.w3.org/1998/Math/MathML">
                <ci>S2</ci>
            </math>
        </dataGenerator>
        <dataGenerator id="dgDataS1" name="S1 (data)">
            <listOfVariables>
                <variable id="varS1" modelReference="model1" target="#dataS1"/>
            </listOfVariables>
            <math xmlns="http://www.w3.org/1998/Math/MathML">
                <ci>varS1</ci>
            </math>
        </dataGenerator>
        <dataGenerator id="dgDataTime" name="Time">
            <listOfVariables>
                <variable id="varTime" modelReference="model1" target="#dataTime"/>
            </listOfVariables>
            <math xmlns="http://www.w3.org/1998/Math/MathML">
                <ci>varTime</ci>
            </math>
        </dataGenerator>
    </listOfDataGenerators>
    <listOfOutputs>
        <plot2D id="plot1" name="Time Course (Oscli)">
            <listOfCurves>
                <curve id="curve1" xDataReference="time_1" yDataReference="S1_1"/>
                <curve id="curve2" xDataReference="time_1" yDataReference="S2_1"/>
                <curve id="curve3" xDataReference="dgDataTime" yDataReference="dgDataS1"/>
            </listOfCurves>
        </plot2D>
    </listOfOutputs>
</sedML>
```

**Listing A.2:** *SED-ML document using DataSource and DataDescription*

### A.3 Simulation experiments with repeatedTasks

The RepeatedTask makes it possible to encode a large number of different simulation experiments. In this section several such simulation experiments are presented.

#### A.3.1 Time course parameter scan (`L1V3_repeated-scan-oscli.omex`)

In this example a repeatedTask is used to run repeated uniformTimeCourse simulations with a deterministic simulation algorithm. Within the repeatedTask after each run the parameter value is changed, resulting in a time course parameter scan.

NOTE: This example produces three dimensional results (time, species concentration, multiple repeats). SED-ML Level 1 Version 5 provides ways to post-process these values with the `dimensionTerm` of the Variable class, but here they are not used, meaning that every individual element in the `xDataReference` is plotted vs. every individual element in the `yDataReference`, effectively flattening the values by overlaying them onto the desired plot. The breaks between dimensions should be used as breaks between any connected lines, so that spurious lines from the end of one plot to the beginning of the next are not present. This document can be found at https://sed-ml.org/examples/L1V3/L1V3_repeated-scan-oscli/repeated-scan-oscli.xml, and an OMEX version at https://sed-ml.org/examples/L1V3/L1V3_repeated-scan-oscli.omex.

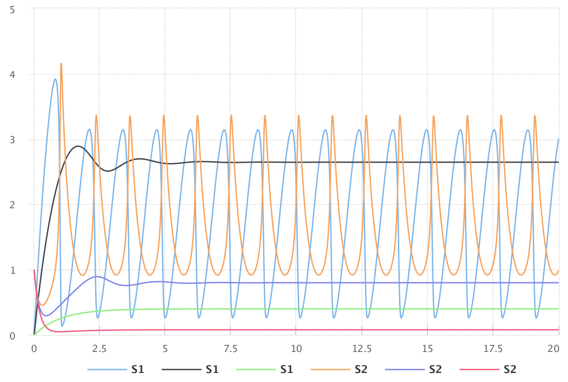

**Figure A.3:** *The simulation result gained from the simulation description given in Listing A.3. Simulation with SED-ML web tools [2].*

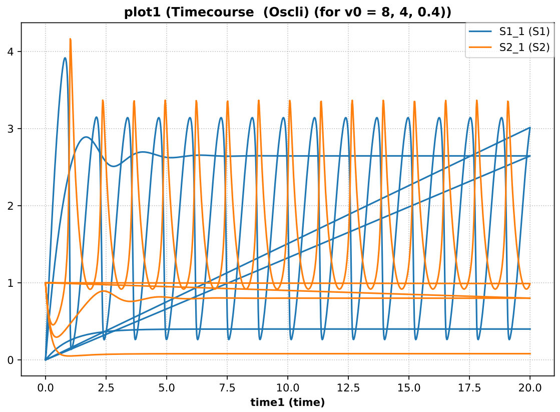

**Figure A.4:** *Simulation with tellurium [6].*

```
<?xml version="1.0" encoding="utf-8"?>
<sedML xmlns="http://sed-ml.org/sed-ml/level1/version5" level="1" version="5">
  <listOfSimulations>
    <uniformTimeCourse id="timecourse1" initialTime="0" outputStartTime="0" outputEndTime="20"
         numberOfSteps="1000">
      <algorithm kisaoID="KISAO:0000019" />
    </uniformTimeCourse>
  </listOfSimulations>
  <listOfModels>
    <model id="model1" language="urn:sedml:language:sbml" source="./oscli.xml" />
  </listOfModels>
  <listOfTasks>
    <task id="task0" modelReference="model1" simulationReference="timecourse1" />
    <repeatedTask id="task1" resetModel="true" range="current">
      <listOfRanges>
        <vectorRange id="current">
          <value>8</value>
          <value>4</value>
          <value>0.4</value>
        </vectorRange>
      </listOfRanges>
      <listOfChanges>
        <setValue target="/sbml:sbml/sbml:model/sbml:listOfParameters/sbml:parameter[@id='J0_v0']"
          range="current" modelReference="model1">
          <math xmlns="http://www.w3.org/1998/Math/MathML">
            <ci> current </ci>
          </math>
        </setValue>
```

```
      </listOfChanges>
      <listOfSubTasks>
        <subTask order="1" task="task0" />
      </listOfSubTasks>
    </repeatedTask>
  </listOfTasks>
  <listOfDataGenerators>
    <dataGenerator id="time1" name="time">
      <listOfVariables>
        <variable id="time" symbol="urn:sedml:symbol:time" taskReference="task1" />
      </listOfVariables>
      <math xmlns="http://www.w3.org/1998/Math/MathML">
        <ci> time </ci>
      </math>
    </dataGenerator>
    <dataGenerator id="J0_v0_1" name="J0_v0">
      <listOfVariables>
        <variable id="J0_v0" name="J0_v0" taskReference="task1" target="/sbml:sbml/sbml:model/
             sbml:listOfParameters/sbml:parameter[@id='J0_v0']" />
      </listOfVariables>
      <math xmlns="http://www.w3.org/1998/Math/MathML">
        <ci> J0_v0 </ci>
      </math>
    </dataGenerator>
    <dataGenerator id="S1_1" name="S1">
      <listOfVariables>
        <variable id="S1" name="S1" taskReference="task1" target="/sbml:sbml/sbml:model/
             sbml:listOfSpecies/sbml:species[@id='S1']" />
      </listOfVariables>
      <math xmlns="http://www.w3.org/1998/Math/MathML">
        <ci> S1 </ci>
      </math>
    </dataGenerator>
    <dataGenerator id="S2_1" name="S2">
      <listOfVariables>
        <variable id="S2" name="S2" taskReference="task1" target="/sbml:sbml/sbml:model/
             sbml:listOfSpecies/sbml:species[@id='S2']" />
      </listOfVariables>
      <math xmlns="http://www.w3.org/1998/Math/MathML">
        <ci> S2 </ci>
      </math>
    </dataGenerator>
  </listOfDataGenerators>
  <listOfOutputs>
    <plot2D id="plot1" name="Timecourse  (Oscli) (for v0 = 8, 4, 0.4)">
      <listOfCurves>
        <curve id="curve1" xDataReference="time1" yDataReference="S1_1" />
        <curve id="curve2" xDataReference="time1" yDataReference="S2_1" />
      </listOfCurves>
    </plot2D>
  </listOfOutputs>
</sedML>
```

**Listing A.3:** *SED-ML document implementing the one dimensional time course parameter scan*

#### A.3.2 Steady state parameter scan (`L1V3_repeated-steady-scan-oscli.omex`)

In this example a repeatedTask is used in combination with a steadyState simulation task (performing a steady state computation). On each repeat a parameter is varied resulting in a steady state parameter scan. This document can be found at `https://sed-ml.org/examples/L1V3/L1V3_repeated-steady-scan-oscli/repeated-steady-scan-oscli.xml`, and an OMEX version at `https://sed-ml.org/examples/L1V3/L1V3_repeated-steady-scan-oscli.omex`.

```
<?xml version="1.0" encoding="utf-8"?>
<!-- Written by libSedML v1.1.4992.38982 see http://libsedml.sf.net -->
<sedML xmlns="http://sed-ml.org/sed-ml/level1/version5" level="1" version="5">
  <listOfSimulations>
    <steadyState id="steady1">
      <algorithm kisaoID="KISAO:0000282" />
    </steadyState>
  </listOfSimulations>
  <listOfModels>
    <model id="model1" language="urn:sedml:language:sbml" source="./oscli.xml" />
  </listOfModels>
  <listOfTasks>
    <task id="task0" modelReference="model1" simulationReference="steady1" />
    <repeatedTask id="task1" resetModel="true" range="current">
      <listOfRanges>
        <uniformRange id="current" start="0" end="10" numberOfSteps="100" type="linear" />
      </listOfRanges>
      <listOfChanges>
        <setValue target="/sbml:sbml/sbml:model/sbml:listOfParameters/sbml:parameter[@id='J0_v0']"
          range="current" modelReference="model1">
          <math xmlns="http://www.w3.org/1998/Math/MathML">
            <ci> current </ci>
```

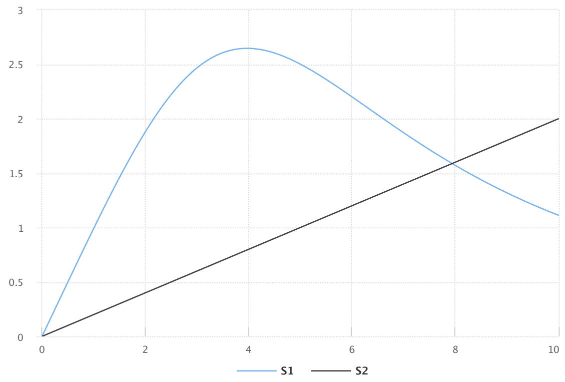

**Figure A.5:** *The simulation result from the simulation description given in Listing A.4. Simulation with SED-ML web tools [2].*

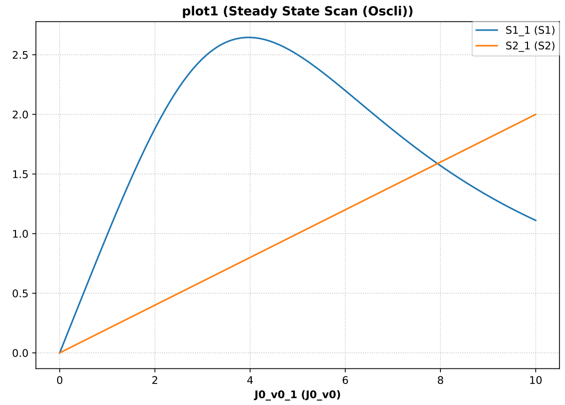

**Figure A.6:** *Simulation with tellurium [6].*

```
          </math>
        </setValue>
      </listOfChanges>
      <listOfSubTasks>
        <subTask order="1" task="task0" />
      </listOfSubTasks>
    </repeatedTask>
  </listOfTasks>
  <listOfDataGenerators>
    <dataGenerator id="J0_v0_1" name="J0_v0">
      <listOfVariables>
        <variable id="J0_v0" name="J0_v0" taskReference="task1" target="/sbml:sbml/sbml:model/
             sbml:listOfParameters/sbml:parameter[@id='J0_v0']" />
      </listOfVariables>
      <math xmlns="http://www.w3.org/1998/Math/MathML">
        <ci> J0_v0 </ci>
      </math>
    </dataGenerator>
    <dataGenerator id="S1_1" name="S1">
      <listOfVariables>
        <variable id="S1" name="S1" taskReference="task1" target="/sbml:sbml/sbml:model/
             sbml:listOfSpecies/sbml:species[@id='S1']" />
      </listOfVariables>
      <math xmlns="http://www.w3.org/1998/Math/MathML">
        <ci> S1 </ci>
      </math>
    </dataGenerator>
    <dataGenerator id="S2_1" name="S2">
      <listOfVariables>
        <variable id="S2" name="S2" taskReference="task1" target="/sbml:sbml/sbml:model/
             sbml:listOfSpecies/sbml:species[@id='S2']" />
      </listOfVariables>
      <math xmlns="http://www.w3.org/1998/Math/MathML">
        <ci> S2 </ci>
      </math>
    </dataGenerator>
  </listOfDataGenerators>
  <listOfOutputs>
    <plot2D id="plot1" name="Steady State Scan (Oscli)">
      <listOfCurves>
        <curve id="curve1" xDataReference="J0_v0_1" yDataReference="S1_1" />
        <curve id="curve2" xDataReference="J0_v0_1" yDataReference="S2_1" />
      </listOfCurves>
    </plot2D>
    <report id="report1" name="Steady State Values">
      <listOfDataSets>
        <dataSet id="col1" dataReference="J0_v0_1" label="J0_v0" />
        <dataSet id="col2" dataReference="S1_1" label="S1" />
        <dataSet id="col3" dataReference="S2_1" label="S2" />
      </listOfDataSets>
    </report>
  </listOfOutputs>
</sedML>
```

**Listing A.4:** *SED-ML document implementing the one dimensional steady state parameter scan*

#### A.3.3 Stochastic simulation (L1V3_repeated-stochastic-runs.omex)

In this example a repeatedTask is used to run a stochastic simulation multiple times. Running just one stochastic trace does not provide a complete picture of the behavior of a system. A large number of such traces is needed. This example demonstrates the basic use case of running ten traces of a simulation by using a repeatedTask which runs ten uniform time course simulations (each performing a stochastic simulation run).

NOTE: This example produces three dimensional results (time, species concentration, multiple repeats). SED-ML Level 1 Version 5 provides ways to post-process these values with the `dimensionTerm` of the Variable class, but here they are not used, meaning that every individual element in the `xDataReference` is plotted vs. every individual element in the `yDataReference`, effectively flattening the values by overlaying them onto the desired plot. The breaks between dimensions should be used as breaks between any connected lines, so that spurious lines from the end of one plot to the beginning of the next are not present.

This document can be found at https://sed-ml.org/examples/L1V3/L1V3_repeated-stochastic-runs/repeated-stochastic-runs.xml, and an OMEX version at https://sed-ml.org/examples/L1V3/L1V3_repeated-stochastic-runs.omex.

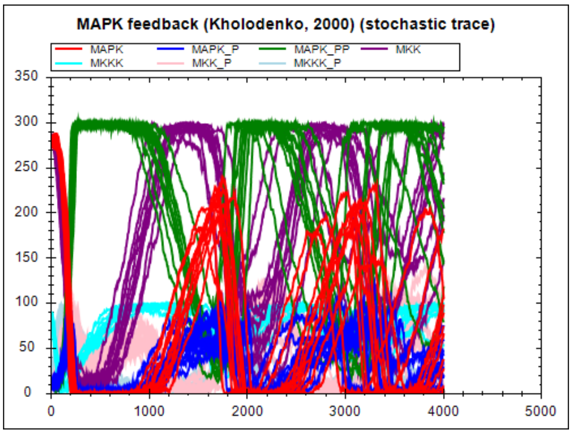

**Figure A.7:** *The simulation result from the simulation description given in Listing A.5. Simulation with SED-ML web tools [2].*

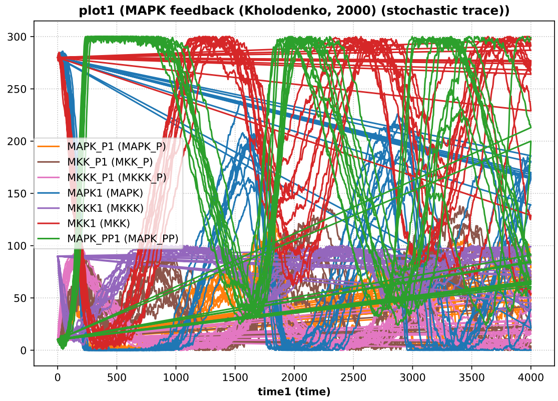

**Figure A.8:** *Simulation with tellurium [6].*

```xml
<?xml version="1.0" encoding="utf-8"?>
<sedML xmlns="http://sed-ml.org/sed-ml/level1/version5" level="1" version="5">
  <listOfSimulations>
    <uniformTimeCourse id="timecourse1" initialTime="0" outputStartTime="0" outputEndTime="4000"
        numberOfSteps="1000">
      <algorithm kisaoID="KISAO:0000241" />
    </uniformTimeCourse>
  </listOfSimulations>
  <listOfModels>
    <model id="model1" language="urn:sedml:language:sbml" source="./BorisEJB.xml" />
  </listOfModels>
  <listOfTasks>
    <task id="task0" modelReference="model1" simulationReference="timecourse1" />
    <repeatedTask id="task1" resetModel="true" range="current">
      <listOfRanges>
        <uniformRange id="current" start="0" end="10" numberOfSteps="10" type="linear" />
      </listOfRanges>
      <listOfSubTasks>
        <subTask order="1" task="task0" />
      </listOfSubTasks>
    </repeatedTask>
  </listOfTasks>
  <listOfDataGenerators>
    <dataGenerator id="time1" name="time">
      <listOfVariables>
        <variable id="time" taskReference="task1" symbol="urn:sedml:symbol:time" />
      </listOfVariables>
      <math xmlns="http://www.w3.org/1998/Math/MathML">
        <ci> time </ci>
      </math>
    </dataGenerator>
```

```
    <dataGenerator id="MAPK1" name="MAPK">
      <listOfVariables>
        <variable id="MAPK" name="MAPK" taskReference="task1" target="/sbml:sbml/sbml:model/
             sbml:listOfSpecies/sbml:species[@id='MAPK']" />
      </listOfVariables>
      <math xmlns="http://www.w3.org/1998/Math/MathML">
        <ci> MAPK </ci>
      </math>
    </dataGenerator>
    <dataGenerator id="MAPK_P1" name="MAPK_P">
      <listOfVariables>
        <variable id="MAPK_P" name="MAPK_P" taskReference="task1" target="/sbml:sbml/sbml:model/
             sbml:listOfSpecies/sbml:species[@id='MAPK_P']" />
      </listOfVariables>
      <math xmlns="http://www.w3.org/1998/Math/MathML">
        <ci> MAPK_P </ci>
      </math>
    </dataGenerator>
    <dataGenerator id="MAPK_PP1" name="MAPK_PP">
      <listOfVariables>
        <variable id="MAPK_PP" name="MAPK_PP" taskReference="task1" target="/sbml:sbml/sbml:model/
             sbml:listOfSpecies/sbml:species[@id='MAPK_PP']" />
      </listOfVariables>
      <math xmlns="http://www.w3.org/1998/Math/MathML">
        <ci> MAPK_PP </ci>
      </math>
    </dataGenerator>
    <dataGenerator id="MKK1" name="MKK">
      <listOfVariables>
        <variable id="MKK" name="MKK" taskReference="task1" target="/sbml:sbml/sbml:model/
             sbml:listOfSpecies/sbml:species[@id='MKK']" />
      </listOfVariables>
      <math xmlns="http://www.w3.org/1998/Math/MathML">
        <ci> MKK </ci>
      </math>
    </dataGenerator>
    <dataGenerator id="MKK_P1" name="MKK_P">
      <listOfVariables>
        <variable id="MKK_P" name="MKK_P" taskReference="task1" target="/sbml:sbml/sbml:model/
             sbml:listOfSpecies/sbml:species[@id='MKK_P']" />
      </listOfVariables>
      <math xmlns="http://www.w3.org/1998/Math/MathML">
        <ci> MKK_P </ci>
      </math>
    </dataGenerator>
    <dataGenerator id="MKKK1" name="MKKK">
      <listOfVariables>
        <variable id="MKKK" name="MKKK" taskReference="task1" target="/sbml:sbml/sbml:model/
             sbml:listOfSpecies/sbml:species[@id='MKKK']" />
      </listOfVariables>
      <math xmlns="http://www.w3.org/1998/Math/MathML">
        <ci> MKKK </ci>
      </math>
    </dataGenerator>
    <dataGenerator id="MKKK_P1" name="MKKK_P">
      <listOfVariables>
        <variable id="MKKK_P" name="MKKK_P" taskReference="task1" target="/sbml:sbml/sbml:model/
             sbml:listOfSpecies/sbml:species[@id='MKKK_P']" />
      </listOfVariables>
      <math xmlns="http://www.w3.org/1998/Math/MathML">
        <ci> MKKK_P </ci>
      </math>
    </dataGenerator>
  </listOfDataGenerators>
  <listOfOutputs>
    <plot2D id="plot1" name="MAPK feedback (Kholodenko, 2000) (stochastic trace)">
      <listOfCurves>
        <curve id="curve1" xDataReference="time1" yDataReference="MAPK1" />
        <curve id="curve2" xDataReference="time1" yDataReference="MAPK_P1" />
        <curve id="curve3" xDataReference="time1" yDataReference="MAPK_PP1" />
        <curve id="curve4" xDataReference="time1" yDataReference="MKK1" />
        <curve id="curve5" xDataReference="time1" yDataReference="MKKK1" />
        <curve id="curve6" xDataReference="time1" yDataReference="MKK_P1" />
        <curve id="curve7" xDataReference="time1" yDataReference="MKKK_P1" />
      </listOfCurves>
    </plot2D>
  </listOfOutputs>
</sedML>
```

**Listing A.5:** *SED-ML document implementing repeated stochastic runs*

#### A.3.4 Simulation perturbation (L1V3_oscli-nested-pulse.omex)

Often it is interesting to see how the dynamic behavior of a model changes when some perturbations are applied to the model. In this example a repeatedTask is used iterating a oneStep task (that advances an

ODE integration to the next output step). During the steps a single parameter is modified effectively causing the oscillations of a model to stop. Once the value is reset the oscillations recover.

Note: In the example a functionalRange is used, although the same result could also be achieved using the setValue element directly.

This document can be found at the URL `https://sed-ml.org/examples/L1V3/L1V3_oscli-nested-pulse/oscli-nested-pulse.xml`, and an OMEX version at the URL `https://sed-ml.org/examples/L1V3/L1V3_oscli-nested-pulse.omex`.

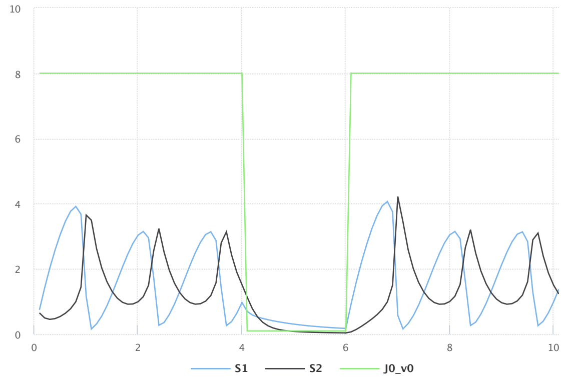

**Figure A.9:** *The simulation result from the simulation description given in Listing A.6. Simulation with SED-ML web tools [2].*

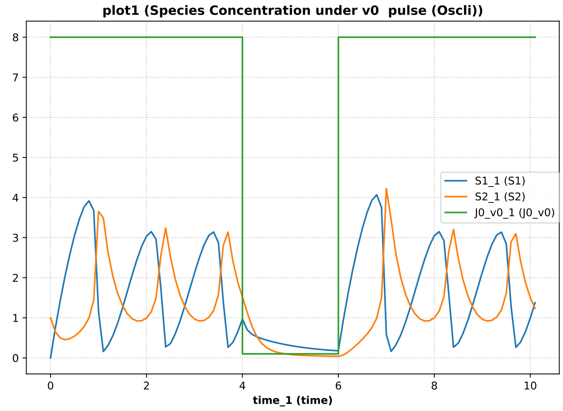

**Figure A.10:** *Simulation with tellurium [6].*

```
<?xml version="1.0" encoding="utf-8"?>
<sedML xmlns="http://sed-ml.org/sed-ml/level1/version5" level="1" version="5">
  <listOfSimulations>
    <oneStep id="stepper" step="0.1">
      <algorithm kisaoID="KISAO:0000019" />
    </oneStep>
  </listOfSimulations>
  <listOfModels>
    <model id="model1" language="urn:sedml:language:sbml" source="./oscli.xml" />
  </listOfModels>
  <listOfTasks>
    <task id="task0" modelReference="model1" simulationReference="stepper" />
    <repeatedTask id="task1" resetModel="false" range="index">
      <listOfRanges>
        <uniformRange id="index" start="0" end="10" numberOfSteps="100" type="linear" />
        <functionalRange id="current" range="index">
          <math xmlns="http://www.w3.org/1998/Math/MathML">
            <piecewise>
              <piece>
                <cn> 8 </cn>
                <apply>
                  <lt />
                  <ci> index </ci>
                  <cn> 1 </cn>
                </apply>
              </piece>
              <piece>
                <cn> 0.1 </cn>
                <apply>
                  <and />
                  <apply>
                    <geq />
                    <ci> index </ci>
                    <cn> 4 </cn>
                  </apply>
                  <apply>
                    <lt />
                    <ci> index </ci>
                    <cn> 6 </cn>
                  </apply>
                </apply>
              </piece>
              <otherwise>
                <cn> 8 </cn>
              </otherwise>
```

```
            </piecewise>
          </math>
        </functionalRange>
      </listOfRanges>
      <listOfChanges>
        <setValue target="/sbml:sbml/sbml:model/sbml:listOfParameters/sbml:parameter[@id='J0_v0']"
          range="current" modelReference="model1">
          <math xmlns="http://www.w3.org/1998/Math/MathML">
            <ci> current </ci>
          </math>
        </setValue>
      </listOfChanges>
      <listOfSubTasks>
        <subTask order="1" task="task0" />
      </listOfSubTasks>
    </repeatedTask>
  </listOfTasks>
  <listOfDataGenerators>
    <dataGenerator id="time_1" name="time">
      <listOfVariables>
        <variable id="time" symbol="urn:sedml:symbol:time" taskReference="task1" />
      </listOfVariables>
      <math xmlns="http://www.w3.org/1998/Math/MathML">
        <ci> time </ci>
      </math>
    </dataGenerator>
    <dataGenerator id="J0_v0_1" name="J0_v0">
      <listOfVariables>
        <variable id="J0_v0" name="J0_v0" taskReference="task1" target="/sbml:sbml/sbml:model/
             sbml:listOfParameters/sbml:parameter[@id='J0_v0']" />
      </listOfVariables>
      <math xmlns="http://www.w3.org/1998/Math/MathML">
        <ci> J0_v0 </ci>
      </math>
    </dataGenerator>
    <dataGenerator id="S1_1" name="S1">
      <listOfVariables>
        <variable id="S1" name="S1" taskReference="task1" target="/sbml:sbml/sbml:model/
             sbml:listOfSpecies/sbml:species[@id='S1']" />
      </listOfVariables>
      <math xmlns="http://www.w3.org/1998/Math/MathML">
        <ci> S1 </ci>
      </math>
    </dataGenerator>
    <dataGenerator id="S2_1" name="S2">
      <listOfVariables>
        <variable id="S2" name="S2" taskReference="task1" target="/sbml:sbml/sbml:model/
             sbml:listOfSpecies/sbml:species[@id='S2']" />
      </listOfVariables>
      <math xmlns="http://www.w3.org/1998/Math/MathML">
        <ci> S2 </ci>
      </math>
    </dataGenerator>
  </listOfDataGenerators>
  <listOfOutputs>
    <plot2D id="plot1" name="Species Concentration under v0  pulse (Oscli)">
      <listOfCurves>
        <curve id="curve1" xDataReference="time_1" yDataReference="S1_1" />
        <curve id="curve2" xDataReference="time_1" yDataReference="S2_1" />
        <curve id="curve3" xDataReference="time_1" yDataReference="J0_v0_1" />
      </listOfCurves>
    </plot2D>
    <report id="report1" name="Species Concentration under v0  pulse (Oscli)">
      <listOfDataSets>
        <dataSet id="col0" dataReference="time_1" label="time" />
        <dataSet id="col1" dataReference="J0_v0_1" label="J0_v0" />
        <dataSet id="col2" dataReference="S1_1" label="S1" />
        <dataSet id="col3" dataReference="S2_1" label="S2" />
      </listOfDataSets>
    </report>
  </listOfOutputs>
</sedML>
```

**Listing A.6:** *SED-ML document implementing the perturbation experiment*

#### A.3.5 2D steady state parameter scan (`L1V3_parameter-scan-2d.omex`)

This example uses a repeatedTask which runs over another repeatedTask which performs a steady state computation. Each repeated simulation task modifies a different parameter.

NOTE: This example produces three dimensional results (time, species concentration, multiple repeats). SED-ML Level 1 Version 5 provides ways to post-process these values with the `dimensionTerm` attribute of the Variable class, but here they are not used, meaning that every individual element in the `xDataReference` is plotted vs. every individual element in the `yDataReference`, effectively flattening the values by overlaying them onto the desired plot. The breaks between dimensions should be used as breaks between any connected lines, so that spurious lines from the end of one plot to the beginning of the next are not present.

This document can be found at the URL https://sed-ml.org/examples/L1V3/L1V3_parameter-scan-2d/parameter-scan-2d.xml, and an OMEX version at the URL https://sed-ml.org/examples/L1V3/L1V3_parameter-scan-2d.omex.

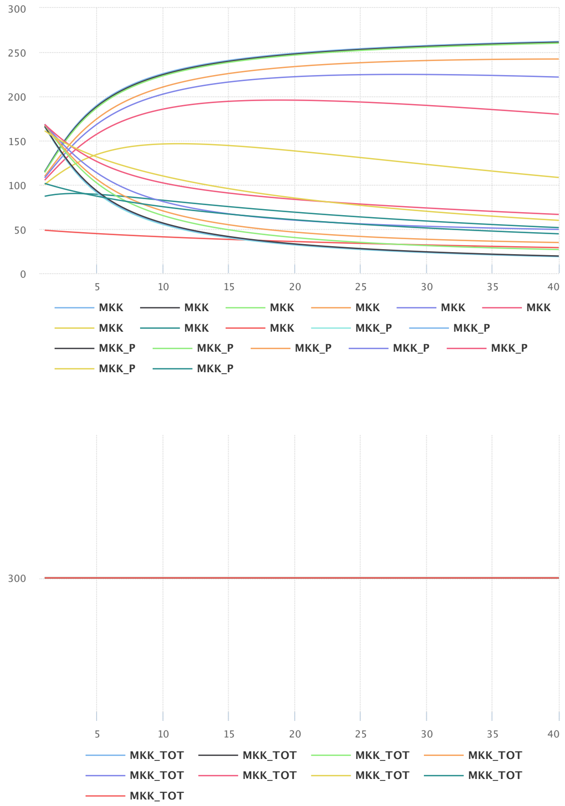

**Figure A.11:** *The simulation result gained from the simulation description given in Listing A.7. Simulation with SED-ML web tools [2].*

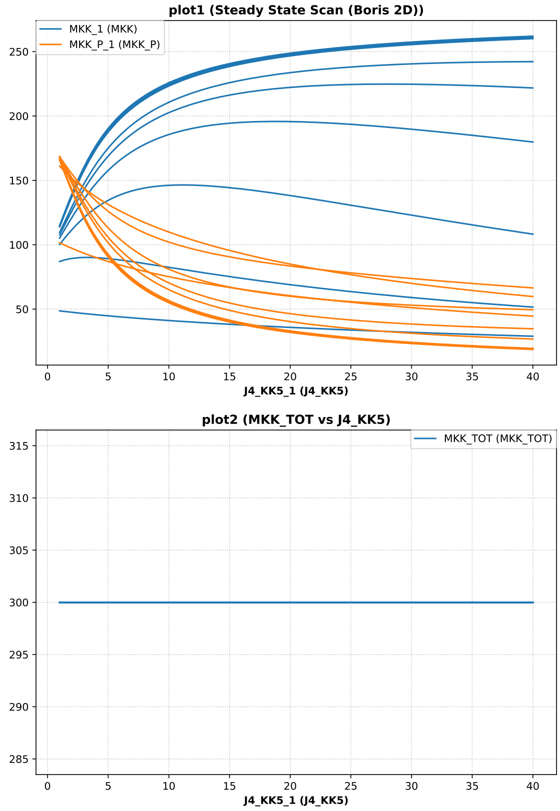

**Figure A.12:** *Simulation with tellurium [6].*

```xml
<?xml version="1.0" encoding="utf-8"?>
<sedML xmlns="http://sed-ml.org/sed-ml/level1/version5" level="1" version="5">
  <listOfSimulations>
    <steadyState id="steady1">
      <algorithm kisaoID="KISAO:0000282" />
    </steadyState>
  </listOfSimulations>
  <listOfModels>
    <model id="model1" language="urn:sedml:language:sbml" source="BorisEJB.xml" />
  </listOfModels>
  <listOfTasks>
    <task id="task0" modelReference="model1" simulationReference="steady1" />
    <repeatedTask id="task1" resetModel="false" range="current">
      <listOfRanges>
        <vectorRange id="current">
          <value>1</value>
          <value>5</value>
          <value>10</value>
          <value>50</value>
          <value>60</value>
          <value>70</value>
          <value>80</value>
          <value>90</value>
          <value>100</value>
        </vectorRange>
      </listOfRanges>
      <listOfChanges>
```

```
        <setValue target="/sbml:sbml/sbml:model/sbml:listOfParameters/sbml:parameter[@id='J1_KK2']"
          range="current" modelReference="model1">
          <math xmlns="http://www.w3.org/1998/Math/MathML">
            <ci> current </ci>
          </math>
        </setValue>
      </listOfChanges>
      <listOfSubTasks>
        <subTask order="1" task="task2" />
      </listOfSubTasks>
    </repeatedTask>
    <repeatedTask id="task2" resetModel="false" range="current1">
      <listOfRanges>
        <uniformRange id="current1" start="1" end="40" numberOfSteps="100" type="linear" />
      </listOfRanges>
      <listOfChanges>
        <setValue target="/sbml:sbml/sbml:model/sbml:listOfParameters/sbml:parameter[@id='J4_KK5']"
          range="current1" modelReference="model1">
          <math xmlns="http://www.w3.org/1998/Math/MathML">
            <ci> current1 </ci>
          </math>
        </setValue>
      </listOfChanges>
      <listOfSubTasks>
        <subTask order="1" task="task0" />
      </listOfSubTasks>
    </repeatedTask>
  </listOfTasks>
  <listOfDataGenerators>
    <dataGenerator id="J4_KK5_1" name="J4_KK5">
      <listOfVariables>
        <variable id="J4_KK5" name="J4_KK5" taskReference="task1" target="/sbml:sbml/sbml:model/
             sbml:listOfParameters/sbml:parameter[@id='J4_KK5']" />
      </listOfVariables>
      <math xmlns="http://www.w3.org/1998/Math/MathML">
        <ci> J4_KK5 </ci>
      </math>
    </dataGenerator>
    <dataGenerator id="J1_KK2_1" name="J1_KK2">
      <listOfVariables>
        <variable id="J1_KK2" name="J1_KK2" taskReference="task1" target="/sbml:sbml/sbml:model/
             sbml:listOfParameters/sbml:parameter[@id='J1_KK2']" />
      </listOfVariables>
      <math xmlns="http://www.w3.org/1998/Math/MathML">
        <ci> J1_KK2 </ci>
      </math>
    </dataGenerator>
    <dataGenerator id="MKK_1" name="MKK">
      <listOfVariables>
        <variable id="MKK" name="MKK" taskReference="task1" target="/sbml:sbml/sbml:model/
             sbml:listOfSpecies/sbml:species[@id='MKK']" />
      </listOfVariables>
      <math xmlns="http://www.w3.org/1998/Math/MathML">
        <ci> MKK </ci>
      </math>
    </dataGenerator>
    <dataGenerator id="MKK_P_1" name="MKK_P">
      <listOfVariables>
        <variable id="MKK_P" name="MKK_P" taskReference="task1" target="/sbml:sbml/sbml:model/
             sbml:listOfSpecies/sbml:species[@id='MKK_P']" />
      </listOfVariables>
      <math xmlns="http://www.w3.org/1998/Math/MathML">
        <ci> MKK_P </ci>
      </math>
    </dataGenerator>
    <dataGenerator id="MKK_PP_1" name="MKK_PP_1">
      <listOfVariables>
        <variable id="MKK_PP_1" name="MKK_PP" taskReference="task1" target="/sbml:sbml/sbml:model/
             sbml:listOfSpecies/sbml:species[@id='MKK_PP']" />
      </listOfVariables>
      <math xmlns="http://www.w3.org/1998/Math/MathML">
        <ci> MKK_PP_1 </ci>
      </math>
    </dataGenerator>
    <dataGenerator id="MKK_TOT" name="MKK_TOT">
      <listOfVariables>
        <variable id="MKK" name="MKK" taskReference="task1" target="/sbml:sbml/sbml:model/
             sbml:listOfSpecies/sbml:species[@id='MKK']" />
        <variable id="MKK_P" name="MKK_P" taskReference="task1" target="/sbml:sbml/sbml:model/
             sbml:listOfSpecies/sbml:species[@id='MKK_P']" />
        <variable id="MKK_PP" name="MKK_PP" taskReference="task1" target="/sbml:sbml/sbml:model/
             sbml:listOfSpecies/sbml:species[@id='MKK_PP']" />
      </listOfVariables>
      <math xmlns="http://www.w3.org/1998/Math/MathML">
        <apply>
          <plus/>
          <ci> MKK </ci>
          <ci> MKK_P </ci>
          <ci> MKK_PP </ci>
```

```
        </apply>
      </math>
    </dataGenerator>
  </listOfDataGenerators>
  <listOfOutputs>
    <plot2D id="plot1" name="Steady State Scan (Boris 2D)">
      <listOfCurves>
        <curve id="curve1" xDataReference="J4_KK5_1" yDataReference="MKK_1" />
        <curve id="curve2" xDataReference="J4_KK5_1" yDataReference="MKK_P_1" />
      </listOfCurves>
    </plot2D>
    <plot2D id="plot2" name="MKK_TOT vs J4_KK5">
      <listOfCurves>
        <curve id="curve3" xDataReference="J4_KK5_1" yDataReference="MKK_TOT" />
      </listOfCurves>
    </plot2D>
    <report id="report1" name="Steady State Values (Boris2D)">
      <listOfDataSets>
        <dataSet id="col0" dataReference="J4_KK5_1" label="J4_KK5" />
        <dataSet id="col1" dataReference="J1_KK2_1" label="J1_KK2" />
        <dataSet id="col2" dataReference="MKK_1" label="MKK" />
        <dataSet id="col3" dataReference="MKK_P_1" label="MKK_P" />
        <dataSet id="col_4" dataReference="MKK_PP_1" label="MKK_PP_1" />
        <dataSet id="col4" dataReference="MKK_TOT" label="MKK_TOT" />
      </listOfDataSets>
    </report>
  </listOfOutputs>
</sedML>
```

**Listing A.7:** *SED-ML document implementing the two dimensional steady state parameter scan*

### A.4 Simulation experiments with different model languages

SED-ML allows to specify models in various languages, e.g., SBML [16] and CellML [9] (see Section 3.3.1 for more information). This section demonstrates the same simulation experiment with the model either in SBML (Appendix A.4.1) or in CellML (Appendix A.4.2).

#### A.4.1 Van der Pol oscillator in SBML (`L1V3_vanderpol-sbml.omex`)

The following example provides a SED-ML description for the simulation of the Van der Pol oscillator in SBML [16]. The time-course and the behavior in the phase plane are plotted. The mathematical model and the performed simulation experiment are identical to Appendix A.4.2. This document can be found at `https://sed-ml.org/examples/L1V3/L1V3_vanderpol-sbml/vanderpol.xml`, and an OMEX version at `https://sed-ml.org/examples/L1V3/L1V3_vanderpol-sbml.omex`.

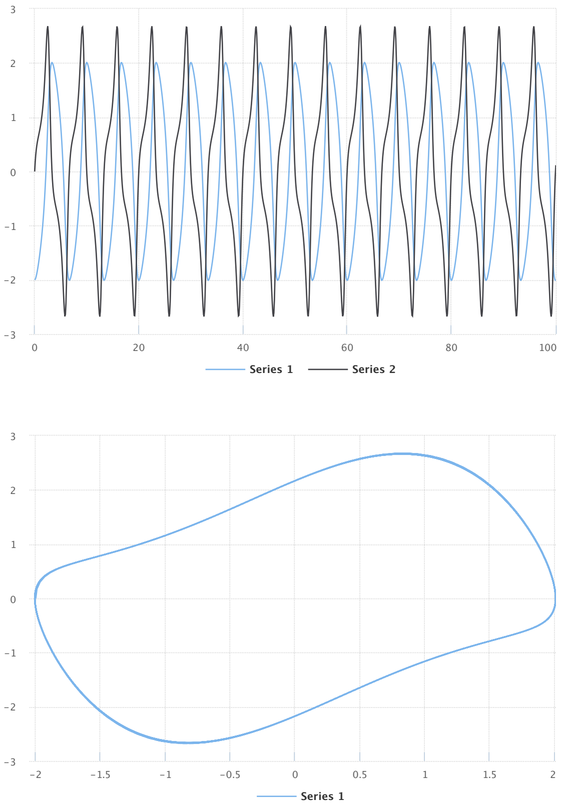

**Figure A.13:** *The simulation result gained from the simulation description given in Listing A.8. Simulation with SED-ML web tools [2].*

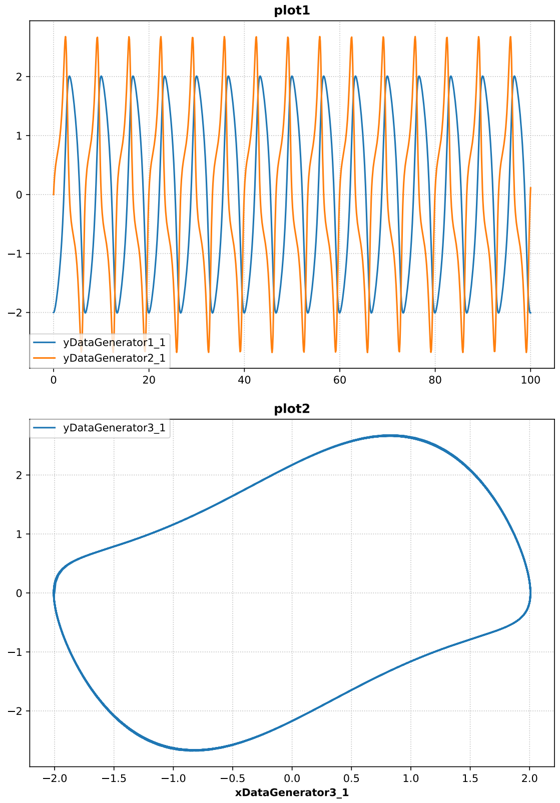

**Figure A.14:** *Simulation with tellurium [6].*

```
<?xml version='1.0' encoding='UTF-8'?>
<sedML level="1" version="5" xmlns="http://sed-ml.org/sed-ml/level1/version5">
    <listOfSimulations>
        <uniformTimeCourse id="simulation1" initialTime="0" numberOfSteps="1000" outputEndTime="100"
             outputStartTime="0">
            <algorithm kisaoID="KISAO:0000019">
                <listOfAlgorithmParameters>
                    <algorithmParameter kisaoID="KISAO:0000211" value="1e-07"/>
                    <algorithmParameter kisaoID="KISAO:0000475" value="BDF"/>
                    <algorithmParameter kisaoID="KISAO:0000481" value="true"/>
                    <algorithmParameter kisaoID="KISAO:0000476" value="Newton"/>
                    <algorithmParameter kisaoID="KISAO:0000477" value="Dense"/>
                    <algorithmParameter kisaoID="KISAO:0000480" value="0"/>
                    <algorithmParameter kisaoID="KISAO:0000415" value="500"/>
                    <algorithmParameter kisaoID="KISAO:0000467" value="0"/>
                    <algorithmParameter kisaoID="KISAO:0000478" value="Banded"/>
                    <algorithmParameter kisaoID="KISAO:0000209" value="1e-07"/>
```

```
                <algorithmParameter kisaoID="KISAO:0000479" value="0"/>
            </listOfAlgorithmParameters>
        </algorithm>
    </uniformTimeCourse>
</listOfSimulations>
<listOfModels>
    <model id="model" language="urn:sedml:language:sbml" source="vanderpol-sbml.xml"/>
</listOfModels>
<listOfTasks>
    <repeatedTask id="repeatedTask" range="once" resetModel="true">
        <listOfRanges>
            <vectorRange id="once">
                <value> 1 </value>
            </vectorRange>
        </listOfRanges>
        <listOfSubTasks>
            <subTask order="1" task="task1"/>
        </listOfSubTasks>
    </repeatedTask>
    <task id="task1" modelReference="model" simulationReference="simulation1"/>
</listOfTasks>
<listOfDataGenerators>
    <dataGenerator id="xDataGenerator1_1">
        <listOfVariables>
            <variable id="xVariable1_1" taskReference="task1" symbol="urn:sedml:symbol:time" />
        </listOfVariables>
        <math xmlns="http://www.w3.org/1998/Math/MathML">
            <ci> xVariable1_1 </ci>
        </math>
    </dataGenerator>
    <dataGenerator id="yDataGenerator1_1">
        <listOfVariables>
            <variable id="yVariable1_1" target="/sbml:sbml/sbml:model/sbml:listOfSpecies/sbml:species[@id='x']" taskReference="repeatedTask" modelReference="model"/>
        </listOfVariables>
        <math xmlns="http://www.w3.org/1998/Math/MathML">
            <ci> yVariable1_1 </ci>
        </math>
    </dataGenerator>
    <dataGenerator id="xDataGenerator2_1">
        <listOfVariables>
            <variable id="xVariable2_1" taskReference="task1" symbol="urn:sedml:symbol:time" />
        </listOfVariables>
        <math xmlns="http://www.w3.org/1998/Math/MathML">
            <ci> xVariable2_1 </ci>
        </math>
    </dataGenerator>
    <dataGenerator id="yDataGenerator2_1">
        <listOfVariables>
            <variable id="yVariable2_1" target="/sbml:sbml/sbml:model/sbml:listOfSpecies/sbml:species[@id='y']" taskReference="repeatedTask" modelReference="model"/>
        </listOfVariables>
        <math xmlns="http://www.w3.org/1998/Math/MathML">
            <ci> yVariable2_1 </ci>
        </math>
    </dataGenerator>
    <dataGenerator id="xDataGenerator3_1">
        <listOfVariables>
            <variable id="xVariable3_1" target="/sbml:sbml/sbml:model/sbml:listOfSpecies/sbml:species[@id='x']" taskReference="repeatedTask" modelReference="model"/>
        </listOfVariables>
        <math xmlns="http://www.w3.org/1998/Math/MathML">
            <ci> xVariable3_1 </ci>
        </math>
    </dataGenerator>
    <dataGenerator id="yDataGenerator3_1">
        <listOfVariables>
            <variable id="yVariable3_1" target="/sbml:sbml/sbml:model/sbml:listOfSpecies/sbml:species[@id='y']" taskReference="repeatedTask" modelReference="model"/>
        </listOfVariables>
        <math xmlns="http://www.w3.org/1998/Math/MathML">
            <ci> yVariable3_1 </ci>
        </math>
    </dataGenerator>
</listOfDataGenerators>
<listOfOutputs>
    <plot2D id="plot1">
        <listOfCurves>
            <curve id="curve1_1" xDataReference="xDataGenerator1_1" yDataReference="yDataGenerator1_1"/>
            <curve id="curve2_1" xDataReference="xDataGenerator2_1" yDataReference="yDataGenerator2_1"/>
        </listOfCurves>
    </plot2D>
    <plot2D id="plot2">
        <listOfCurves>
            <curve id="curve3_1" xDataReference="xDataGenerator3_1" yDataReference="yDataGenerator3_1"/>
        </listOfCurves>
```

```
        </plot2D>
    </listOfOutputs>
</sedML>
```

**Listing A.8:** *Van der Pol Model (SBML) Simulation Description in SED-ML*

#### A.4.2 Van der Pol oscillator in CellML (`L1V3_vanderpol-cellml.omex`)

The following example provides a SED-ML description for the simulation of the Van der Pol model in CellML [9]. The time-course and the behavior in the phase plane are plotted. The mathematical model and the performed simulation experiment are identical to Appendix A.4.1. This document can be found at `https://sed-ml.org/examples/L1V3/L1V3_vanderpol-cellml/vanderpol.xml`, and an OMEX version at `https://sed-ml.org/examples/L1V3/L1V3_vanderpol-cellml.omex`.

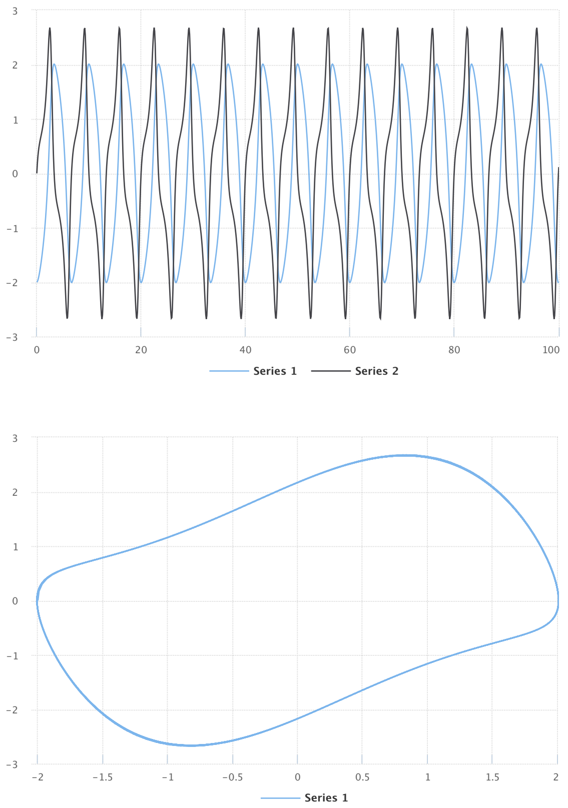

**Figure A.15:** *The simulation result gained from the simulation description given in Listing A.9. Simulation with SED-ML web tools [2].*

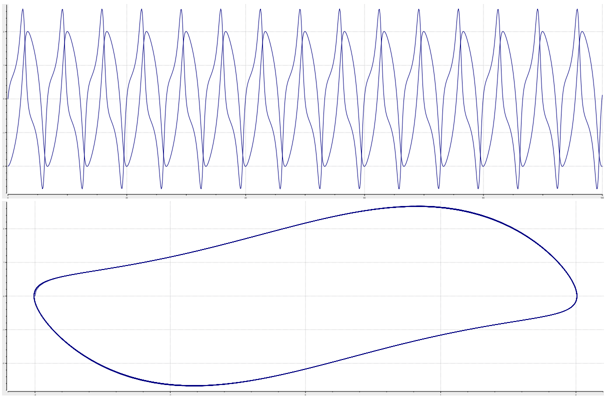

**Figure A.16:** *Simulation with OpenCOR [11].*

```
<?xml version='1.0' encoding='UTF-8'?>
<sedML level="1" version="5" xmlns="http://sed-ml.org/sed-ml/level1/version5" xmlns:cellml="http://www.
      cellml.org/cellml/1.0#">
    <listOfSimulations>
        <uniformTimeCourse id="simulation1" initialTime="0" numberOfSteps="1000" outputEndTime="100"
             outputStartTime="0">
            <algorithm kisaoID="KISAO:0000019">
                <listOfAlgorithmParameters>
                    <algorithmParameter kisaoID="KISAO:0000211" value="1e-07"/>
                    <algorithmParameter kisaoID="KISAO:0000475" value="BDF"/>
                    <algorithmParameter kisaoID="KISAO:0000481" value="true"/>
                    <algorithmParameter kisaoID="KISAO:0000476" value="Newton"/>
                    <algorithmParameter kisaoID="KISAO:0000477" value="Dense"/>
                    <algorithmParameter kisaoID="KISAO:0000480" value="0"/>
                    <algorithmParameter kisaoID="KISAO:0000415" value="500"/>
                    <algorithmParameter kisaoID="KISAO:0000467" value="0"/>
                    <algorithmParameter kisaoID="KISAO:0000478" value="Banded"/>
                    <algorithmParameter kisaoID="KISAO:0000209" value="1e-07"/>
                    <algorithmParameter kisaoID="KISAO:0000479" value="0"/>
```

```
                </listOfAlgorithmParameters>
            </algorithm>
        </uniformTimeCourse>
    </listOfSimulations>
    <listOfModels>
        <model id="model" language="urn:sedml:language:cellml.1_0" source="vanderpol-model.cellml"/>
    </listOfModels>
    <listOfTasks>
        <repeatedTask id="repeatedTask" range="once" resetModel="true">
            <listOfRanges>
                <vectorRange id="once">
                    <value> 1 </value>
                </vectorRange>
            </listOfRanges>
            <listOfSubTasks>
                <subTask order="1" task="task1"/>
            </listOfSubTasks>
        </repeatedTask>
        <task id="task1" modelReference="model" simulationReference="simulation1"/>
    </listOfTasks>
    <listOfDataGenerators>
        <dataGenerator id="xDataGenerator1_1">
            <listOfVariables>
                <variable id="xVariable1_1" target="/cellml:model/cellml:component[@name='main']/cellml:variable[@name='t']" taskReference="repeatedTask"/>
            </listOfVariables>
            <math xmlns="http://www.w3.org/1998/Math/MathML">
                <ci> xVariable1_1 </ci>
            </math>
        </dataGenerator>
        <dataGenerator id="yDataGenerator1_1">
            <listOfVariables>
                <variable id="yVariable1_1" target="/cellml:model/cellml:component[@name='main']/cellml:variable[@name='x']" taskReference="repeatedTask"/>
            </listOfVariables>
            <math xmlns="http://www.w3.org/1998/Math/MathML">
                <ci> yVariable1_1 </ci>
            </math>
        </dataGenerator>
        <dataGenerator id="xDataGenerator2_1">
            <listOfVariables>
                <variable id="xVariable2_1" target="/cellml:model/cellml:component[@name='main']/cellml:variable[@name='t']" taskReference="repeatedTask"/>
            </listOfVariables>
            <math xmlns="http://www.w3.org/1998/Math/MathML">
                <ci> xVariable2_1 </ci>
            </math>
        </dataGenerator>
        <dataGenerator id="yDataGenerator2_1">
            <listOfVariables>
                <variable id="yVariable2_1" target="/cellml:model/cellml:component[@name='main']/cellml:variable[@name='y']" taskReference="repeatedTask"/>
            </listOfVariables>
            <math xmlns="http://www.w3.org/1998/Math/MathML">
                <ci> yVariable2_1 </ci>
            </math>
        </dataGenerator>
        <dataGenerator id="xDataGenerator3_1">
            <listOfVariables>
                <variable id="xVariable3_1" target="/cellml:model/cellml:component[@name='main']/cellml:variable[@name='x']" taskReference="repeatedTask"/>
            </listOfVariables>
            <math xmlns="http://www.w3.org/1998/Math/MathML">
                <ci> xVariable3_1 </ci>
            </math>
        </dataGenerator>
        <dataGenerator id="yDataGenerator3_1">
            <listOfVariables>
                <variable id="yVariable3_1" target="/cellml:model/cellml:component[@name='main']/cellml:variable[@name='y']" taskReference="repeatedTask"/>
            </listOfVariables>
            <math xmlns="http://www.w3.org/1998/Math/MathML">
                <ci> yVariable3_1 </ci>
            </math>
        </dataGenerator>
    </listOfDataGenerators>
    <listOfOutputs>
        <plot2D id="plot1">
            <listOfCurves>
                <curve id="curve1_1" xDataReference="xDataGenerator1_1" yDataReference="yDataGenerator1_1"/>
                <curve id="curve2_1" xDataReference="xDataGenerator2_1" yDataReference="yDataGenerator2_1"/>
            </listOfCurves>
        </plot2D>
        <plot2D id="plot2">
            <listOfCurves>
                <curve id="curve3_1" xDataReference="xDataGenerator3_1" yDataReference="yDataGenerator3_1"/>
```

```
            </listOfCurves>
        </plot2D>
    </listOfOutputs>
</sedML>
```

**Listing A.9:** *Van der Pol Model (CellML) Simulation Description in SED-ML*

### A.5 Reproducing publication results

SED-ML allows to describe simulation experiments from publications in a reproducible manner. This section provides such examples.

#### A.5.1 Le Loup model (L1V3_leloup-sbml.omex)

The following example provides a SED-ML description for the simulation of the model based on the publication [17].

The model is referenced by its SED-ML id model1 and refers to the model with the URL https://www.ebi.ac.uk/biomodels/model/download/BIOMD0000000012.2?filename=BIOMD0000000012_url.xml. A second model is defined in the example, using model1 as a source and applying additional changes to it, in this case updating two model parameters.

One simulation setup is defined in the listOfSimulations. It is a uniformTimeCourse over 380 time units, providing 1000 output points. The algorithm used is the CVODE solver, as denoted by the KiSAO ID KiSAO:0000019.

A number of dataGenerators are defined, which are the prerequisite for defining the simulation output. The first dataGenerator with id time collects the simulation time. tim1 maps on the Mt entity in the model that is used in task1 which in the model model1. The dataGenerator named per_tim1 maps on the Cn entity in model1. Finally the fourth and fifth dataGenerators map on the Mt and per_tim entity respectively in the updated model with ID model2.

The output defined in the experiment consists of three 2D plots. The first plot has two curves and provides the time course of the simulation using the tim mRNA concentrations from both tasks. The second plot shows the per_tim concentration against the tim concentration for the oscillating model. The third plot shows the same plot for the chaotic model. The resulting three plots are depicted in Figure A.17 and A.18. This document can be found at https://sed-ml.org/examples/L1V3/L1V3_leloup-sbml/leloup-sbml.xml, and an OMEX version at https://sed-ml.org/examples/L1V3/L1V3_leloup-sbml.omex.

```
<?xml version="1.0" encoding="utf-8"?>
<sedML  xmlns="http://sed-ml.org/sed-ml/level1/version5" level="1" version="5">
  <listOfSimulations>
    <uniformTimeCourse id="simulation1" initialTime="0" outputStartTime="0" outputEndTime="380"
        numberOfSteps="1000">
      <algorithm kisaoID="KISAO:0000019" />
    </uniformTimeCourse>
  </listOfSimulations>
  <listOfModels>
    <model id="model1" name="Circadian Oscillations" language="urn:sedml:language:sbml" source="https://
        www.ebi.ac.uk/biomodels/model/download/BIOMD0000000012?filename=BIOMD0000000012_url.xml" />
    <model id="model2" name="Circadian Chaos" language="urn:sedml:language:sbml" source="#model1">
      <listOfChanges>
        <changeAttribute target="/sbml:sbml/sbml:model/sbml:listOfParameters/sbml:parameter[@id=&quot;
            V_mT&quot;]/@value" newValue="0.28" />
        <changeAttribute target="/sbml:sbml/sbml:model/sbml:listOfParameters/sbml:parameter[@id=&quot;
            V_dT&quot;]/@value" newValue="4.8" />
      </listOfChanges>
    </model>
  </listOfModels>
  <listOfTasks>
    <task id="task1" modelReference="model1" simulationReference="simulation1" />
    <task id="task2" modelReference="model2" simulationReference="simulation1" />
  </listOfTasks>
  <listOfDataGenerators>
    <dataGenerator id="time" name="time">
      <listOfVariables>
        <variable id="t" taskReference="task1" symbol="urn:sedml:symbol:time" />
      </listOfVariables>
      <math xmlns="http://www.w3.org/1998/Math/MathML">
        <ci> t </ci>
      </math>
    </dataGenerator>
    <dataGenerator id="tim1" name="tim mRNA">
      <listOfVariables>
        <variable id="v1" taskReference="task1" target="/sbml:sbml/sbml:model/sbml:listOfSpecies/
            sbml:species[@id='Mt']" />
      </listOfVariables>
      <math xmlns="http://www.w3.org/1998/Math/MathML">
        <ci> v1 </ci>
      </math>
    </dataGenerator>
    <dataGenerator id="per_tim1" name="nuclear PER-TIM complex">
      <listOfVariables>
```

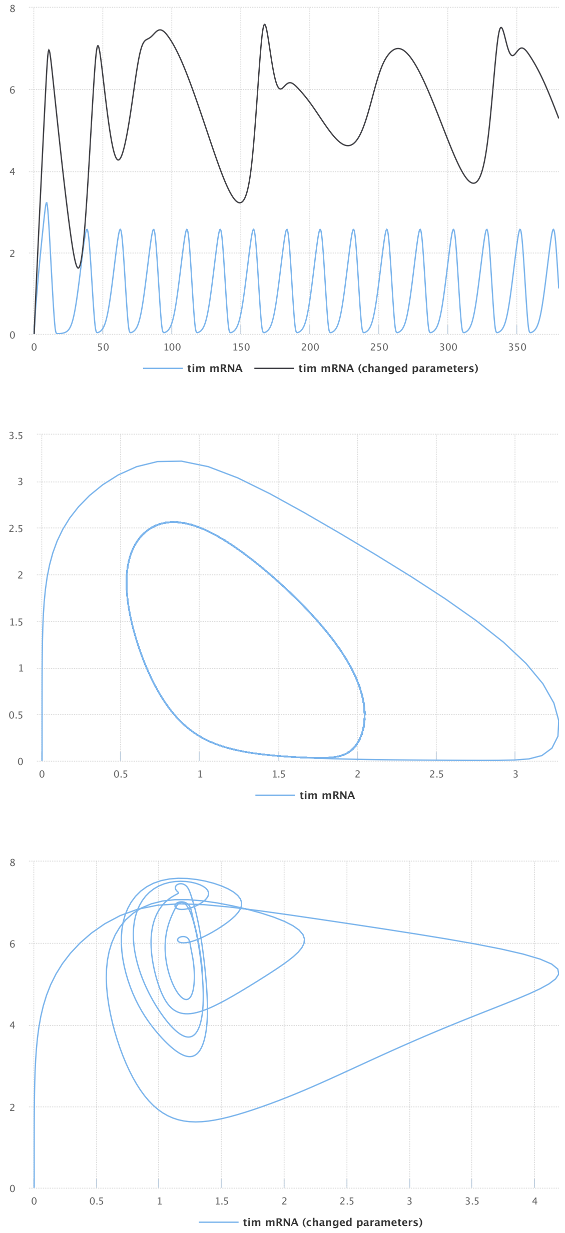

**Figure A.17:** *The simulation result gained from the simulation description given in Listing [A.10](#). Simulation with SED-ML web tools [2].*

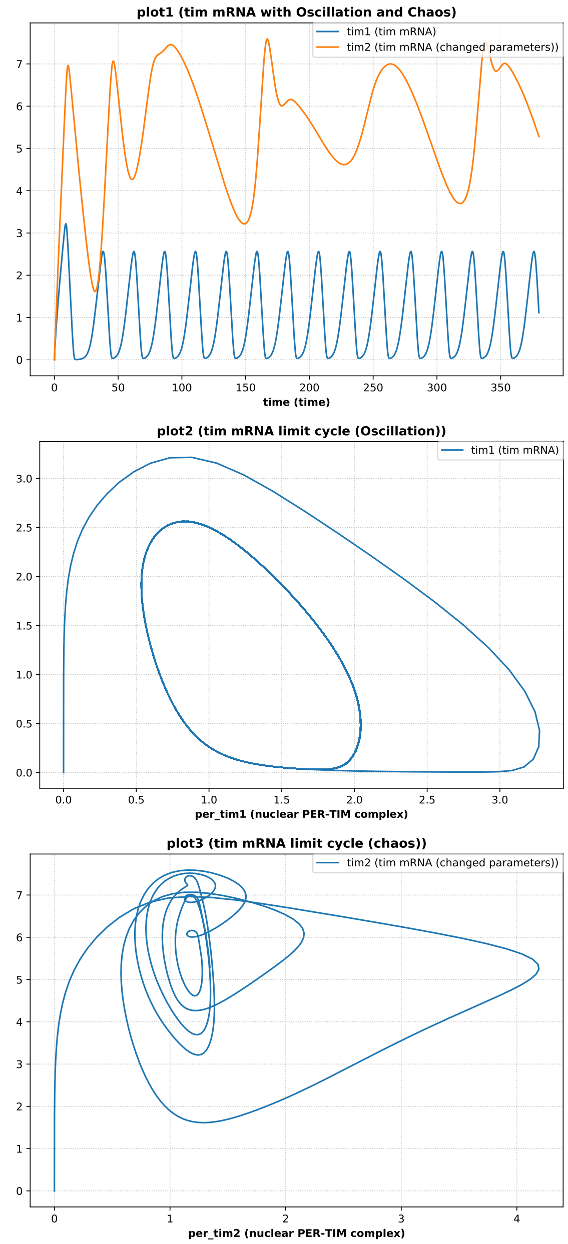

**Figure A.18:** *Simulation with tellurium [6].*

```
      <variable id="v1a" taskReference="task1" target="/sbml:sbml/sbml:model/sbml:listOfSpecies/
           sbml:species[@id='Cn']" />
    </listOfVariables>
    <math xmlns="http://www.w3.org/1998/Math/MathML">
      <ci> v1a </ci>
    </math>
  </dataGenerator>
  <dataGenerator id="tim2" name="tim mRNA (changed parameters)">
    <listOfVariables>
      <variable id="v2" taskReference="task2" target="/sbml:sbml/sbml:model/sbml:listOfSpecies/
           sbml:species[@id='Mt']" />
    </listOfVariables>
    <math xmlns="http://www.w3.org/1998/Math/MathML">
      <ci> v2 </ci>
    </math>
  </dataGenerator>
  <dataGenerator id="per_tim2" name="nuclear PER-TIM complex">
    <listOfVariables>
```

```
        <variable id="v2a" taskReference="task2" target="/sbml:sbml/sbml:model/sbml:listOfSpecies/
             sbml:species[@id='Cn']" />
      </listOfVariables>
      <math xmlns="http://www.w3.org/1998/Math/MathML">
        <ci> v2a </ci>
      </math>
    </dataGenerator>
  </listOfDataGenerators>
  <listOfOutputs>
    <plot2D id="plot1" name="tim mRNA with Oscillation and Chaos">
      <listOfCurves>
        <curve id="c1" xDataReference="time" yDataReference="tim1" />
        <curve id="c2" xDataReference="time" yDataReference="tim2" />
      </listOfCurves>
    </plot2D>
   <plot2D id="plot2" name="tim mRNA limit cycle (Oscillation)">
      <listOfCurves>
        <curve id="c3" xDataReference="per_tim1" yDataReference="tim1" />
      </listOfCurves>
    </plot2D>
   <plot2D id="plot3" name="tim mRNA limit cycle (chaos)">
      <listOfCurves>
        <curve id="c4" xDataReference="per_tim2" yDataReference="tim2" />
      </listOfCurves>
    </plot2D>
  </listOfOutputs>
</sedML>
```

**Listing A.10:** *LeLoup Model Simulation Description in SED-ML*

#### A.5.2 IkappaB signaling (L1V3_ikkapab.omex)

The following example provides a SED-ML description for the simulation of the IkappaB-NF-kappaB signaling module described in [14].

This model is referenced by its SED-ML ID `model1` and refers to the model with the URL https://www.ebi.ac.uk/biomodels/model/download/BIOMD0000000140.2?filename=BIOMD0000000140_url.xml.

The simulation description specifies one simulation `simulation1`, which is a uniform timecourse simulation that simulates the model for 41 hours. `task1` then applies this simulation to the model.

As output this simulation description collects four parameters: `Total_NFkBn`, `Total_IkBbeta`, `Total_IkBeps` and `Total_IkBalpha`. These variables are plotted against the simulation time as shown in Figure A.19 and A.20. This document can be found at https://sed-ml.org/examples/L1V3/L1V3_ikappab/ikappab.xml, and an OMEX version at https://sed-ml.org/examples/L1V3/L1V3_ikappab.omex.

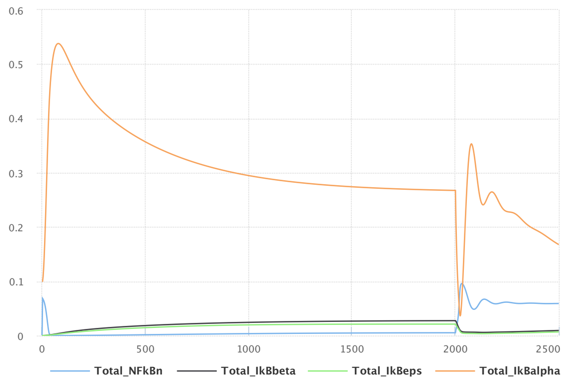

**Figure A.19:** *The simulation result gained from the simulation description given in Listing A.11. Simulation with SED-ML web tools [2].*

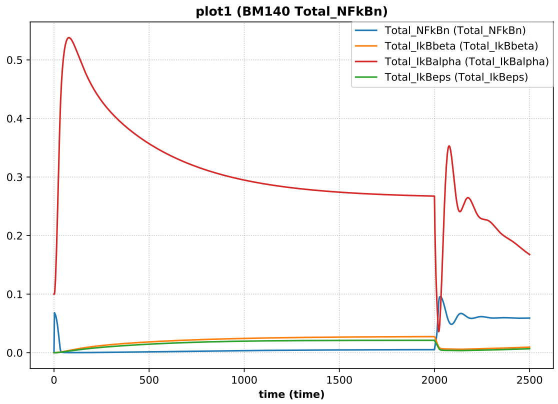

**Figure A.20:** *Simulation with tellurium [6].*

```
<?xml version="1.0" encoding="utf-8"?>
<sedML xmlns="http://sed-ml.org/sed-ml/level1/version5" level="1" version="5">
  <listOfSimulations>
    <uniformTimeCourse id="simulation1"
    initialTime="0" outputStartTime="0" outputEndTime="2500"
    numberOfSteps="1000" >
```

```
      <algorithm kisaoID="KISAO:0000019"/>
    </uniformTimeCourse>
  </listOfSimulations>
  <listOfModels>
    <model id="model1" language="urn:sedml:language:sbml" source="https://www.ebi.ac.uk/biomodels/model/download/BIOMD0000000140?filename=BIOMD0000000140_url.xml"/>
  </listOfModels>
  <listOfTasks>
    <task id="task1" modelReference="model1"
    simulationReference="simulation1"/>
  </listOfTasks>
  <listOfDataGenerators>
    <dataGenerator id="time" name="time">
      <listOfVariables>
        <variable id="time1" taskReference="task1" symbol="urn:sedml:symbol:time"/>
      </listOfVariables>
      <math xmlns="http://www.w3.org/1998/Math/MathML">
        <ci>time1</ci>
      </math>
    </dataGenerator>
    <dataGenerator id="Total_NFkBn" name="Total_NFkBn">
      <listOfVariables>
        <variable id="Total_NFkBn1" taskReference="task1"
        target="/sbml:sbml/sbml:model/sbml:listOfParameters/sbml:parameter[@id='Total_NFkBn']"/>
      </listOfVariables>
      <math xmlns="http://www.w3.org/1998/Math/MathML">
        <ci>Total_NFkBn1</ci>
      </math>
    </dataGenerator>
    <dataGenerator id="Total_IkBbeta" name="Total_IkBbeta">
      <listOfVariables>
        <variable id="Total_IkBbeta1" taskReference="task1"
        target="/sbml:sbml/sbml:model/sbml:listOfParameters/sbml:parameter[@id='Total_IkBbeta']" />
      </listOfVariables>
      <math xmlns="http://www.w3.org/1998/Math/MathML">
        <ci>Total_IkBbeta1</ci>
      </math>
    </dataGenerator>
    <dataGenerator id="Total_IkBeps" name="Total_IkBeps">
      <listOfVariables>
        <variable id="Total_IkBeps1" taskReference="task1"
        target="/sbml:sbml/sbml:model/sbml:listOfParameters/sbml:parameter[@id='Total_IkBeps']" />
      </listOfVariables>
      <math xmlns="http://www.w3.org/1998/Math/MathML">
        <ci>Total_IkBeps1</ci>
      </math>
    </dataGenerator>
    <dataGenerator id="Total_IkBalpha" name="Total_IkBalpha">
      <listOfVariables>
        <variable id="Total_IkBalpha1" taskReference="task1"
        target="/sbml:sbml/sbml:model/sbml:listOfParameters/sbml:parameter[@id='Total_IkBalpha']" />
      </listOfVariables>
      <math xmlns="http://www.w3.org/1998/Math/MathML">
        <ci>Total_IkBalpha1</ci>
      </math>
    </dataGenerator>
  </listOfDataGenerators>
  <listOfOutputs>
    <plot2D id="plot1" name="BM140 Total_NFkBn">
      <listOfCurves>
        <curve  id="c1" xDataReference="time"
        yDataReference="Total_NFkBn" />
            <curve  id="c2" xDataReference="time"
        yDataReference="Total_IkBbeta" />
        <curve  id="c3" xDataReference="time"
        yDataReference="Total_IkBeps" />
        <curve id="c4" xDataReference="time"
        yDataReference="Total_IkBalpha" />
      </listOfCurves>
    </plot2D>
  </listOfOutputs>
</sedML>
```

**Listing A.11:** *IkappaB-NF-kappaB signaling Model Simulation Description in SED-ML*

## B. Validation

### B.1 Validation of SED-ML documents

#### B.1.1 Validation and consistency rules

This section summarizes all the conditions that must (or in some cases, at least *should*) be true of a SED-ML Level 1 Version 5 model that uses the SED-ML. We use the same conventions as are used in the SED-ML Level 1 Version 5 Core specification document. In particular, there are different degrees of rule strictness. Formally, the differences are expressed in the statement of a rule: either a rule states that a condition *must* be true, or a rule states that it *should* be true. Rules of the former kind are strict SED-ML validation rules—a model encoded in SED-ML must conform to all of them in order to be considered valid. Rules of the latter kind are consistency rules. To help highlight these differences, we use the following three symbols next to the rule numbers:

- ☑ A checked box indicates a *requirement* for SED-ML conformance. If a model does not follow this rule, it does not conform to the SED-ML specification. (Mnemonic intention behind the choice of symbol: "This must be checked.")
- ▲ A triangle indicates a *recommendation* for model consistency. If a model does not follow this rule, it is not considered strictly invalid as far as the SED-ML specification is concerned; however, it indicates that the model contains a physical or conceptual inconsistency. (Mnemonic intention behind the choice of symbol: "This is a cause for warning.")
- ★ A star indicates a strong recommendation for good modeling practice. This rule is not strictly a matter of SED-ML encoding, but the recommendation comes from logical reasoning. As in the previous case, if a model does not follow this rule, it is not strictly considered an invalid SED-ML encoding. (Mnemonic intention behind the choice of symbol: "You're a star if you heed this.")

The validation rules listed in the following subsections are all stated or implied in the rest of this specification document. They are enumerated here for convenience. Unless explicitly stated, all validation rules concern objects and attributes specifically defined in SED-ML.

For convenience and brevity, we use the shorthand "`x`" to stand for an attribute or element name `x` in the namespace for SED-ML, using the namespace prefix `sedml`. In reality, the prefix string may be different from the literal "`sedml`" used here (and indeed, it can be any valid XML namespace prefix that the modeler or software chooses). We use "`x`" because it is shorter than to write a full explanation everywhere we refer to an attribute or element in the SED-ML namespace.

##### *General rules about this package*

**10101** ☑ To conform to the SED-ML specification for SED-ML Level 1 Version 5, a SED-ML document must declare "`http://sed-ml.org/sed-ml/level1/version5`" as the XMLNamespace to use for elements of this package. (Reference: SED-ML Level 1 Version 5 Section 2.2.1.1.)

**10102** ☑ Wherever they appear in a SED-ML document, elements and attributes from the SED-ML must use the "`http://sed-ml.org/sed-ml/level1/version5`" namespace, declaring so either explicitly or implicitly. (Reference: SED-ML Level 1 Version 5 Section 2.2.1.1.)

#### *Rules for Math objects*

**10201** ☑ Wherever MathML content appears in an SBML document, the MathML content must be placed within a `math` element, and that `math` element must be either explicitly or implicitly declared to be in the XML namespace "`http://www.w3.org/1998/Math/MathML`". (Reference: SED-ML Level 1 Version 4, Section 3.1.)

**10202** ☑ The following is a list of the only MathML 2.0 elements permitted in SED-ML Level 1 Version 4: `abs`, `and`, `annotation`, `annotation-xml`, `apply`, `arccosh`, `arccos`, `arccoth`, `arccot`, `arccsch`, `arccsc`, `arcsech`, `arcsec`, `arcsinh`, `arcsin`, `arctanh`, `arctan`, `ceiling`, `ci`, `cn`, `cosh`, `cos`, `coth`, `cot`, `csch`, `csc`, `csymbol`, `degree`, `divide`, `eq`, `exponentiale`, `exp`, `factorial`, `false`, `floor`, `geq`, `gt`, `implies`, `infinity`, `leq`, `ln`, `logbase`, `log`, `lt`, `max`, `min`, `minus`, `neq`, `notanumber`, `not`, `or`, `otherwise`, `piecewise`, `piece`, `pi`, `plus`, `power`, `quotient`, `rem`, `root`, `sech`, `sec`, `semantics`, `sep`, `sinh`, `sin`, `tanh`, `tan`, `times`, `true`, and `xor`. (Reference: SED-ML Level 1 Version 4, Section 3.1.)

**10203** ☑ In the SED-ML subset of MathML 2.0, the MathML attribute `encoding` is only permitted on `csymbol`, `annotation` and `annotation-xml`. No other MathML elements may have an `encoding` attribute. (Reference: SED-ML Level 1 Version 4, Section 3.1.)

**10204** ☑ In the SED-ML subset of MathML 2.0, the MathML attribute `definitionURL` is only permitted on `ci`, `csymbol` and `semantics`. No other MathML elements may have a `definitionURL` attribute. (Reference: SED-ML Level 1 Version 4, Section 3.1.)

**10205** ☑ In SED-ML, the only values permitted for the attribute `definitionURL` on a `csymbol` are "`http://sed-ml.org/#min`", "`http://sed-ml.org/#max`", "`http://sed-ml.org/#sum`", "`http://sed-ml.org/#product`", "`http://sed-ml.org/functions/#uniform`", "`http://sed-ml.org/functions/#normal`", "`http://sed-ml.org/functions/#lognormal`", "`http://sed-ml.org/functions/#gamma`", and "`http://sed-ml.org/functions/#poisson`". (Reference: SED-ML Level 1 Version 4, Section 3.1.)

**10206** ☑ In the SBML subset of MathML 2.0, the MathML attribute `type` is only permitted on the `cn` construct. No other MathML elements may have a `type` attribute. (Reference: SED-ML Level 1 Version 4, Section 3.1.)

**10207** ☑ The only permitted values for the attribute `type` on MathML `cn` elements are "`e-notation`", "`real`", "`integer`", and "`rational`". (Reference: SED-ML Level 1 Version 4, Section 3.1.)

**10214** ☑ A MathML `ci` element may not be the first element within a MathML `apply` element. (Reference: SED-ML Level 1 Version 4, Section 3.1.)

**10215** ☑ If a MathML `ci` element is not the first element within a MathML `apply`, then the `ci` element's value may only be chosen from the following set of identifiers: the identifiers of Variable and Parameter objects defined in the enclosing ComputeChange, DataGenerator, or FunctionalRange object, and, if the parent of the Math is a SetValue, the value of that SetValue's `range` attribute. (Reference: SED-ML Level 1 Version 4, Section 3.1.)

**10218** ☑ A MathML operator must be supplied the number of arguments appropriate for that operator. (Reference: SED-ML Level 1 Version 4, Section 3.1.)

**10219** ▲ Avoid using the following values for the attribute `definitionURL` on a `csymbol`, as they have been superceded by the `term` attribute of a Variable: "`http://sed-ml.org/#min`", "`http://sed-ml.org/#max`", "`http://sed-ml.org/#sum`", and "`http://sed-ml.org/#product`". (Reference: SED-ML Level 1 Version 4, Section 3.1.)

#### *General rules about identifiers*

**10301** ☑ The value of the attribute id on every SED-ML object must be unique across the set of all id attribute values of all objects in a SED-ML document. (Reference: SED-ML Level 1 Version 5, Section 2.1.4.)

**10302** ☑ The value of a `id` must conform to the syntax of the SedML data type `SId` (Reference: SED-ML Level 1 Version 5, Section 2.1.4.)

**10303** ☑ The value of a `metaid` must conform to the syntax of the XML Type ID (Reference: SED-ML Level 1 Version 5, Section 2.1.4.)

***Rules for the SED-ML Document object***

**20201** ☑ A SED-ML Document object may have the optional SED-ML Level 1 attributes `id`, `name`, and `metaid`. (Reference: SED-ML Level 1 Version 5, Section 2.1.4.)

**20202** ☑ A SED-ML Document object may have the optional SED-ML Level 1 subobjects for notes and annotations. (Reference: SED-ML Level 1 Version 5, Section 2.1.4.)

**20203** ☑ A SED-ML Document object must have the required attributes `level` and `version`. (Reference: SED-ML Level 1 Version 5, Section 2.2.1.)

**20204** ☑ A SED-ML Document object may contain one and only one instance of each of the ListOfDataDescriptions, ListOfModels, ListOfSimulations, ListOfTasks, ListOfDataGenerators, ListOfOutputs, ListOfStyles and ListOfAlgorithmParameters elements. (Reference: SED-ML Level 1 Version 5, Section 2.2.1.)

**20205** ☑ The attribute `level` on a SED-ML Document must have a positive value of data type `integer`. (Reference: SED-ML Level 1 Version 5, Section 2.2.1.)

**20206** ☑ The attribute `version` on a SED-ML Document must have a positive value of data type `integer`. (Reference: SED-ML Level 1 Version 5, Section 2.2.1.)

**20207** ☑ Apart from the general notes and annotations subobjects permitted on all SED-ML objects, a ListOfDataDescriptions container object may only contain DataDescription objects. (Reference: SED-ML Level 1 Version 5, Section 2.2.1.4.)

**20208** ☑ Apart from the general notes and annotations subobjects permitted on all SED-ML objects, a ListOfModels container object may only contain Model objects. (Reference: SED-ML Level 1 Version 5, Section 2.2.1.5.)

**20209** ☑ Apart from the general notes and annotations subobjects permitted on all SED-ML objects, a ListOfSimulations container object may only contain Simulation objects. (Reference: SED-ML Level 1 Version 5, Section 2.2.1.6.)

**20210** ☑ Apart from the general notes and annotations subobjects permitted on all SED-ML objects, a ListOfTasks container object may only contain *AbstractTask* objects. (Reference: SED-ML Level 1 Version 5, Section 2.2.1.7.)

**20211** ☑ Apart from the general notes and annotations subobjects permitted on all SED-ML objects, a ListOfDataGenerators container object may only contain DataGenerator objects. (Reference: SED-ML Level 1 Version 5, Section 2.2.1.8.)

**20212** ☑ Apart from the general notes and annotations subobjects permitted on all SED-ML objects, a ListOfOutputs container object may only contain Output objects. (Reference: SED-ML Level 1 Version 5, Section 2.2.1.9.)

**20213** ☑ Apart from the general notes and annotations subobjects permitted on all SED-ML objects, a ListOfStyles container object may only contain Style objects. (Reference: SED-ML Level 1 Version 5, Section 2.2.1.10.)

**20214** ☑ Apart from the general notes and annotations subobjects permitted on all SED-ML objects, a ListOfAlgorithmParameters container object may only contain AlgorithmParameter objects. (Reference: SED-ML Level 1 Version 5, Section 2.1.7.)

**20215** ☑ A ListOfDataDescriptions object may have the optional SED-ML Level 1 attributes `id`, `name`, and `metaid`. (Reference: SED-ML Level 1 Version 5, Section 2.2.1.4.)

**20216** ☑ A ListOfModels object may have the optional SED-ML Level 1 attributes `id`, `name`, and `metaid`. (Reference: SED-ML Level 1 Version 5, Section 2.2.1.5.)

**20217** ☑ A ListOfSimulations object may have the optional SED-ML Level 1 attributes `id`, `name`, and `metaid`. (Reference: SED-ML Level 1 Version 5, Section 2.2.1.6.)

**20218** ☑ A ListOfTasks object may have the optional SED-ML Level 1 attributes `id`, `name`, and `metaid`. (Reference: SED-ML Level 1 Version 5, Section 2.2.1.7.)

**20219** ☑ A ListOfDataGenerators object may have the optional SED-ML Level 1 attributes `id`, `name`, and `metaid`. (Reference: SED-ML Level 1 Version 5, Section 2.2.1.8.)

**20220** ☑ A ListOfOutputs object may have the optional SED-ML Level 1 attributes `id`, `name`, and `metaid`. (Reference: SED-ML Level 1 Version 5, Section 2.2.1.9.)

**20221** ☑ A ListOfStyles object may have the optional SED-ML Level 1 attributes `id`, `name`, and `metaid`. (Reference: SED-ML Level 1 Version 5, Section 2.2.1.10.)

**20222** ☑ A ListOfAlgorithmParameters object may have the optional SED-ML Level 1 attributes `id`, `name`, and `metaid`. (Reference: SED-ML Level 1 Version 5, Section 2.1.7.)

**20250** ★ Every SED-ML Document should contain at least one Output. (Reference: SED-ML Level 1 Version 5, Section 2.2.1)

#### *Rules for Model objects*

**20301** ☑ A Model object may have the required SED-ML Level 1 attribute `id` and the optional attributes `name` and `metaid`. (Reference: SED-ML Level 1 Version 5, Section 2.1.4.)

**20302** ☑ A Model object may have the optional SED-ML Level 1 subobjects for notes and annotations. (Reference: SED-ML Level 1 Version 5, Section 2.1.4.)

**20303** ☑ A Model object must have the required attributes `language`, `source` and `id`. (Reference: SED-ML Level 1 Version 5, Section 2.2.4.)

**20304** ☑ A Model object may contain one and only one instance of the ListOfChanges element. (Reference: SED-ML Level 1 Version 5, Section 2.2.4.)

**20305** ☑ The attribute `language` on a Model must have a value of data type `URN` (Reference: SED-ML Level 1 Version 5, Section 2.2.4)

**20306** ☑ The attribute `source` on a Model must have a value of data type `anyURI` (Reference: SED-ML Level 1 Version 5, Section 2.2.4)

**20307** ☑ Apart from the general notes and annotations subobjects permitted on all SED-ML objects, a ListOfChanges container object may only contain Change objects. (Reference: SED-ML Level 1 Version 5, Section 2.2.4.)

**20308** ☑ A ListOfChanges object may have the optional SED-ML Level 1 attributes `id`, `name`, and `metaid`. (Reference: SED-ML Level 1 Version 5, Section 2.2.4.)

**20350** ☑ There must not be circular dependencies in model resolution. The `source` attribute of a Model may not directly or indirectly reference itself. (Reference: SED-ML Level 1 Version 5, Section 2.2.4)

**20351** ☑ There must not be circular cross-dependencies in model change resolution. The `target` and `source` attributes of a ComputeChange may not correspond to the `target` and `source` of a different ComputeChange in a different Model. (Reference: SED-ML Level 1 Version 5, Section 2.2.4)

**20352** ☑ The model pointed to by the `source` attribute must exist. (Reference: SED-ML Level 1 Version 5, Section 2.2.4)

**20353** ☑ The model pointed to by the `source` attribute must be encoded in the language defined by the `language` attribute. (Reference: SED-ML Level 1 Version 5, Section 2.2.4)

**20354** ★ Avoid using URNs for the `source` attribute of a Model, as these have become increasingly harder to resolve. (Reference: SED-ML Level 1 Version 5, Section 2.2.4)

**20355** ☑ A Model may only contain an AddXML child if its `language` attribute describes an XML-based language. (Reference: SED-ML Level 1 Version 5, Section 2.2.4)

**20356** ☑ A Model may only contain a RemoveXML child if its `language` attribute describes an XML-based language. (Reference: SED-ML Level 1 Version 5, Section 2.2.4)

**20357** ☑ A Model may only contain a ChangeXML child if its `language` attribute describes an XML-based language. (Reference: SED-ML Level 1 Version 5, Section 2.2.4)

##### *Rules for Change objects*

**20401** ☑ A Change object may have the optional SED-ML Level 1 attributes `id`, `name`, `target` and `metaid`. (Reference: SED-ML Level 1 Version 5, Section 2.1.4.)

**20402** ☑ A Change object may have the optional SED-ML Level 1 subobjects for notes and annotations. (Reference: SED-ML Level 1 Version 5, Section 2.1.4.)

**20403** ☑ If a Change object has no child Algorithm element, it must define the  attribute `target`. (Reference: SED-ML Level 1 Version 5, Section 2.2.5.)

**20404** ☑ The attribute `target` on a Change must have a value of data type `string`. (Reference: SED-ML Level 1 Version 5, Section 2.2.5.)

**20450** ☑ A Change object or one of its derived classes may contain exactly one child Algorithm element. (Reference: SED-ML Level 1 Version 5, Section 2.2.5.)

##### *Rules for AddXML objects*

**20501** ☑ An AddXML object may have the optional SED-ML Level 1 attributes `id`, `name`, and `metaid`. (Reference: SED-ML Level 1 Version 5, Section 2.1.4.)

**20502** ☑ An AddXML object may have the optional SED-ML Level 1 subobjects for notes and annotations. (Reference: SED-ML Level 1 Version 5, Section 2.1.4.)

**20503** ☑ An AddXML object must contain one and only one instance of the XMLNode element. (Reference: SED-ML Level 1 Version 5, Section 2.2.5.2.)

**20550** ☑ The `target` attribute of an AddXML object must point to a valid target in the Model `source`. (Reference: SED-ML Level 1 Version 5, Section 2.2.5)

**20551** ☑ The `target` attribute of an AddXML object be a valid XPath when the Model `language` attribute describes an XML-based language. (Reference: SED-ML Level 1 Version 5, Section 2.2.5)

**20552** ☑ The XML child of an AddXML object must be a valid XML element or list of XML elements. (Reference: SED-ML Level 1 Version 5, Section 2.2.5.2)

**20553** ☑ The XML child of an AddXML object must be in a namespace defined by the `source` attribute of the parent Model object, or explicitly define its own namespace understood by the language of the target model. (Reference: SED-ML Level 1 Version 5, Section 2.2.5.2)

##### *Rules for ChangeAttribute objects*

**20601** ☑ A ChangeAttribute object may have the optional SED-ML Level 1 attributes `id`, `name`, and `metaid`. (Reference: SED-ML Level 1 Version 5, Section 2.1.4.)

**20602** ☑ A ChangeAttribute object may have the optional SED-ML Level 1 subobjects for notes and annotations. (Reference: SED-ML Level 1 Version 5, Section 2.1.4.)

**20603** ☑ A ChangeAttribute object must have the required attribute `newValue`. (Reference: SED-ML Level 1 Version 5, Section 2.2.5.5.)

**20604** ☑ The attribute `newValue` on a ChangeAttribute must have a value of data type `string`. (Reference: SED-ML Level 1 Version 5, Section 2.2.5.5.)

**20650** ☑ The `target` attribute of a ChangeAttribute object must point to a valid target in the Model `source`. (Reference: SED-ML Level 1 Version 5, Section 2.2.5)

**20651** ☑ The `target` attribute of a ChangeAttribute object be a valid XPath when the Model `language` attribute describes an XML-based language. (Reference: SED-ML Level 1 Version 5, Section 2.2.5)

***Rules for Variable objects***

**20701** ☑ A Variable object may have the required SED-ML Level 1 attribute `id` and the optional attributes `name` and `metaid`. (Reference: SED-ML Level 1 Version 5, Section 2.1.4.)

**20702** ☑ A Variable object may have the optional SED-ML Level 1 subobjects for notes and annotations. (Reference: SED-ML Level 1 Version 5, Section 2.1.4.)

**20703** ☑ A Variable object may have the optional attributes `symbol`, `target`, `taskReference`, `modelReference`, `term`, `symbol2`, `target2` and `dimensionTerm`. (Reference: SED-ML Level 1 Version 5, Section 2.1.8.3.)

**20704** ☑ A Variable object may contain one and only one instance of the ListOfAppliedDimensions element. (Reference: SED-ML Level 1 Version 5, Section 2.1.8.3.)

**20705** ☑ The attribute `symbol` on a Variable must have a value of data type `string`. (Reference: SED-ML Level 1 Version 5, Section 2.1.8.3.)

**20706** ☑ The attribute `target` on a Variable must have a value of data type `TargetType` (Reference: SED-ML Level 1 Version 5, Section 2.1.8.3)

**20707** ☑ The value of the attribute `taskReference` of a Variable object must be the identifier of an existing *AbstractTask* object defined in the document. (Reference: SED-ML Level 1 Version 5, Section 2.1.8.3.)

**20708** ☑ The value of the attribute `modelReference` of a Variable object must be the identifier of an existing Model object defined in the document. (Reference: SED-ML Level 1 Version 5, Section 2.1.8.3.)

**20709** ☑ The attribute `term` on a Variable must have a value of data type `anyURI` (Reference: SED-ML Level 1 Version 5, Section 2.1.8.3)

**20710** ☑ The attribute `symbol2` on a Variable must have a value of data type `string`. (Reference: SED-ML Level 1 Version 5, Section 2.1.8.3.)

**20711** ☑ The attribute `target2` on a Variable must have a value of data type `TargetType` (Reference: SED-ML Level 1 Version 5, Section 2.1.8.3)

**20712** ☑ The attribute `dimensionTerm` on a Variable must have a value of data type `string`. (Reference: SED-ML Level 1 Version 5, Section 2.1.8.3.)

**20713** ☑ Apart from the general notes and annotations subobjects permitted on all SED-ML objects, a ListOfAppliedDimensions container object may only contain AppliedDimension objects. (Reference: SED-ML Level 1 Version 5, Section 2.1.8.4.)

**20714** ☑ A ListOfAppliedDimensions object may have the optional SED-ML Level 1 attributes `id`, `name`, and `metaid`. (Reference: SED-ML Level 1 Version 5, Section 2.1.8.4.)

**20750** ☑ Every Variable with a defined `dimensionTerm` attribute must have exactly one ListOfAppliedDimensions child containing at least one child AppliedDimension object. (Reference: SED-ML Level 1 Version 5, Section 2.1.8.3)

**20751** ☑ Every Variable with a defined `target2` or `symbol2` attribute must also define the `term` attribute. (Reference: SED-ML Level 1 Version 5, Section 2.1.8.3)

**20752** ☑ The `target`, `symbol`, `term`, `target2` and `symbol2` attributes of a Variable must collectively define a single mathematical concept. (Reference: SED-ML Level 1 Version 5, Section 2.1.8.3)

**20753** ☑ When the `target` attribute of a Variable is an XPath, it must point to a single model element or attribute. (Reference: SED-ML Level 1 Version 5, Section 2.1.8.3)

**20754** ☑ If the `target` of a Variable child of a DataGenerator points to a DataSource, neither the Variable's `taskReference` nor `modelRefernce` may be set. (Reference: SED-ML Level 1 Version 5, Section 2.1.8.3)

##### *Rules for Parameter objects*

**20801** ☑ A Parameter object may have the required SED-ML Level 1 attribute **id** and the optional attributes **name** and **metaid**. (Reference: SED-ML Level 1 Version 5, Section 2.1.4.)

**20802** ☑ A Parameter object may have the optional SED-ML Level 1 subobjects for notes and annotations. (Reference: SED-ML Level 1 Version 5, Section 2.1.4.)

**20803** ☑ A Parameter object must have the required attributes **value** and **id**. (Reference: SED-ML Level 1 Version 5, Section 2.1.8.2.)

**20804** ☑ The attribute **value** on a Parameter must have a value of data type **double**. (Reference: SED-ML Level 1 Version 5, Section 2.1.8.2.)

##### *Rules for Simulation objects*

**20901** ☑ A Simulation object may have the required SED-ML Level 1 attribute **id** and the optional attributes **name** and **metaid**. (Reference: SED-ML Level 1 Version 5, Section 2.1.4.)

**20902** ☑ A Simulation object may have the optional SED-ML Level 1 subobjects for notes and annotations. (Reference: SED-ML Level 1 Version 5, Section 2.1.4.)

**20903** ☑ A Simulation object must contain one and only one instance of the Algorithm element. (Reference: SED-ML Level 1 Version 5, Section 2.2.6.)

##### *Rules for UniformTimeCourse objects*

**21001** ☑ An UniformTimeCourse object may have the optional SED-ML Level 1 attributes **id**, **name**, and **metaid**. (Reference: SED-ML Level 1 Version 5, Section 2.1.4.)

**21002** ☑ An UniformTimeCourse object may have the optional SED-ML Level 1 subobjects for notes and annotations. (Reference: SED-ML Level 1 Version 5, Section 2.1.4.)

**21003** ☑ An UniformTimeCourse object must have the required attributes **initialTime**, **outputStartTime** and **outputEndTime**, and may have the optional attributes **numberOfPoints** and **numberOfSteps**. (Reference: SED-ML Level 1 Version 5, Section 2.2.6.1.)

**21004** ☑ The attribute **initialTime** on an UniformTimeCourse must have a value of data type **double**. (Reference: SED-ML Level 1 Version 5, Section 2.2.6.1.)

**21005** ☑ The attribute **outputStartTime** on an UniformTimeCourse must have a value of data type **double**. (Reference: SED-ML Level 1 Version 5, Section 2.2.6.1.)

**21006** ☑ The attribute **outputEndTime** on an UniformTimeCourse must have a value of data type **double**. (Reference: SED-ML Level 1 Version 5, Section 2.2.6.1.)

**21007** ☑ The attribute **numberOfPoints** on an UniformTimeCourse must have a positive value of data type **integer**. (Reference: SED-ML Level 1 Version 5, Section 2.2.6.1.)

**21008** ☑ The attribute **numberOfSteps** on an UniformTimeCourse must have a positive value of data type **integer**. (Reference: SED-ML Level 1 Version 5, Section 2.2.6.1.)

**21050** ▲ Avoid use of the **numberOfPoints** attribute of a UniformTimeCourse in favor of the **numberOfSteps** attribute. "Number of Steps" accurately reflects the meaning of the attribute. (Reference: SED-ML Level 1 Version 5, Section 2.2.6.1)

**21051** ☑ The value of the **outputStartTime** attribute of a UniformTimeCourse must be equal to or greater than the value of the **initialTime** attribute. (Reference: SED-ML Level 1 Version 5, Section 2.2.6.1)

**21052** ☑ The value of the **endTime** attribute of a UniformTimeCourse must be equal to or greater than the value of the **outputStartTime** attribute. (Reference: SED-ML Level 1 Version 5, Section 2.2.6.1)

21053 ★ The value of the **numberOfPoints** attribute of a UniformTimeCourse should typically be evenly divisible by five. When this is not the case, it often indicates that the modeler is unaware that the definition of the attribute is actually 'the number of points not including the initial state'. (Reference: SED-ML Level 1 Version 5, Section 2.2.6.1)

21054 ☑ Only one of the attributes **numberOfPoints** or **numberOfSteps** may be defined on a UniformTimeCourse. (Reference: SED-ML Level 1 Version 5, Section 2.2.6.1)

#### *Rules for Algorithm objects*

21101 ☑ An Algorithm object may have the optional SED-ML Level 1 attributes **id**, **name**, and **metaid**. (Reference: SED-ML Level 1 Version 5, Section 2.1.4.)

21102 ☑ An Algorithm object may have the optional SED-ML Level 1 subobjects for notes and annotations. (Reference: SED-ML Level 1 Version 5, Section 2.1.4.)

21103 ☑ An Algorithm object must have the required attribute **kisaoID**. (Reference: SED-ML Level 1 Version 5, Section 2.1.7.)

21104 ☑ An Algorithm object may contain one and only one instance of the ListOfAlgorithmParameters element. (Reference: SED-ML Level 1 Version 5, Section 2.1.7.)

21105 ☑ The attribute **kisaoID** on an Algorithm must have a value of data type **string**. (Reference: SED-ML Level 1 Version 5, Section 2.1.7.)

21106 ☑ Apart from the general notes and annotations subobjects permitted on all SED-ML objects, a ListOfAlgorithmParameters container object may only contain AlgorithmParameter objects. (Reference: SED-ML Level 1 Version 5, Section 2.1.7.)

21107 ☑ A ListOfAlgorithmParameters object may have the optional SED-ML Level 1 attributes **id**, **name**, and **metaid**. (Reference: SED-ML Level 1 Version 5, Section 2.1.7.)

21150 ☑ The value of the **kisaoID** attribute of an Algorithm must be the ID of an algorithm in the KiSAO ontology. (Reference: SED-ML Level 1 Version 5, Section 2.1.7)

#### *Rules for AbstractTask objects*

21201 ☑ An *AbstractTask* object may have the required SED-ML Level 1 attribute **id** and the optional attributes **name** and **metaid**. (Reference: SED-ML Level 1 Version 5, Section 2.1.4.)

21202 ☑ An *AbstractTask* object may have the optional SED-ML Level 1 subobjects for notes and annotations. (Reference: SED-ML Level 1 Version 5, Section 2.1.4.)

21203 ☑ An *AbstractTask* object must have the required attribute **id**. (Reference: SED-ML Level 1 Version 5, Section 2.2.7.1.)

21250 ☑ An *AbstractTask* object may contain exactly one child Algorithm element. (Reference: SED-ML Level 1 Version 5, Section 2.2.7.1.)

21250 ★ Every *AbstractTask* should contribute to at least one Output. (Reference: SED-ML Level 1 Version 5, Section 2.2.7)

#### *Rules for Task objects*

21301 ☑ A Task object may have the required SED-ML Level 1 attribute **id** and the optional attributes **name**, **metaid**, **modelReference**, and **simulationReference**. (Reference: SED-ML Level 1 Version 5, Section 2.1.4.)

21302 ☑ A Task object may have the optional SED-ML Level 1 subobjects for notes and annotations. (Reference: SED-ML Level 1 Version 5, Section 2.1.4.)

21303 ☑ If a Task object has no child Algorithm, it must define the attributes **modelReference** and **simulationReference**. (Reference: SED-ML Level 1 Version 5, Section 2.2.7.1.)

**21304** ☑ The value of the attribute `modelReference` of a Task object must be the identifier of an existing Model object defined in the document. (Reference: SED-ML Level 1 Version 5, Section 2.2.7.1.)

**21305** ☑ The value of the attribute `simulationReference` of a Task object must be the identifier of an existing Simulation object defined in the document. (Reference: SED-ML Level 1 Version 5, Section 2.2.7.1.)

**21350** ☑ An *AbstractTask* object may contain exactly one child Algorithm element. (Reference: SED-ML Level 1 Version 5, Section 2.2.7.1.)

#### *Rules for DataGenerator objects*

**21401** ☑ A DataGenerator object may have the required SED-ML Level 1 attribute `id` and the optional attributes `name` and `metaid`. (Reference: SED-ML Level 1 Version 5, Section 2.1.4.)

**21402** ☑ A DataGenerator object may have the optional SED-ML Level 1 subobjects for notes and annotations. (Reference: SED-ML Level 1 Version 5, Section 2.1.4.)

**21403** ☑ A DataGenerator object must have the required attribute `id`. (Reference: SED-ML Level 1 Version 5, Section 2.2.10.)

**21404** ☑ A DataGenerator object may contain one and only one instance of each of the ListOfVariables, ListOfParameters and ASTNode elements. (Reference: SED-ML Level 1 Version 5, Section 2.2.10.)

**21405** ☑ Apart from the general notes and annotations subobjects permitted on all SED-ML objects, a ListOfVariables container object may only contain Variable objects. (Reference: SED-ML Level 1 Version 5, Section 2.1.8.)

**21406** ☑ Apart from the general notes and annotations subobjects permitted on all SED-ML objects, a ListOfParameters container object may only contain Parameter objects. (Reference: SED-ML Level 1 Version 5, Section 2.1.8.)

**21407** ☑ A ListOfVariables object may have the optional SED-ML Level 1 attributes `id`, `name`, and `metaid`. (Reference: SED-ML Level 1 Version 5, Section 2.1.8.)

**21408** ☑ A ListOfParameters object may have the optional SED-ML Level 1 attributes `id`, `name`, and `metaid`. (Reference: SED-ML Level 1 Version 5, Section 2.1.8.)

#### *Rules for Output objects*

**21501** ☑ An Output object may have the optional SED-ML Level 1 attributes `id`, `name`, and `metaid`. (Reference: SED-ML Level 1 Version 5, Section 2.1.4.)

**21502** ☑ An Output object may have the optional SED-ML Level 1 subobjects for notes and annotations. (Reference: SED-ML Level 1 Version 5, Section 2.1.4.)

**21550** ★ Every DataGenerator should contribute to at least one Output. (Reference: SED-ML Level 1 Version 5, Section 2.2.10)

**21551** ★ The shape of the output of every Variable child of the same DataGenerator should either be scalar or be consistent with its Variable siblings. (Reference: SED-ML Level 1 Version 5, Section 2.2.10)

**21552** ☑ If the `target` of a Variable child of a DataGenerator does not point to a DataSource, the Variable's `taskReference` must be set. (Reference: SED-ML Level 1 Version 5, Section 2.1.8.3)

**21553** ☑ If the `taskReference` of a Variable child of a DataGenerator is set and references an *AbstractTask* that references multiple models, the `modelReference` of the Variable must also be set. (Reference: SED-ML Level 1 Version 5, Section 2.1.8.3)

**21554** ☑ If the `taskReference` of a Variable child of a DataGenerator is set, the `modelReference` of the Variable, if also set, must reference a Model modified by the referenced *AbstractTask*. (Reference: SED-ML Level 1 Version 5, Section 2.1.8.3)

**21555** ☑ An Output object or one of its derived classes may contain exactly one child Algorithm element. (Reference: SED-ML Level 1 Version 5, Section 2.2.11.)

#### *Rules for Plot objects*

**21601** ☑ A *Plot* object may have the optional SED-ML Level 1 attributes **id**, **name**, and **metaid**. (Reference: SED-ML Level 1 Version 5, Section 2.1.4.)

**21602** ☑ A *Plot* object may have the optional SED-ML Level 1 subobjects for notes and annotations. (Reference: SED-ML Level 1 Version 5, Section 2.1.4.)

**21603** ☑ A *Plot* object may have the optional attributes **legend**, **height** and **width**. (Reference: SED-ML Level 1 Version 5, Section 2.2.12.)

**21604** ☑ A *Plot* object may contain one and only one instance of each of the Axis and Axis elements. (Reference: SED-ML Level 1 Version 5, Section 2.2.12.)

**21605** ☑ The attribute **legend** on a *Plot* must have a value of data type **boolean**. (Reference: SED-ML Level 1 Version 5, Section 2.2.12.)

**21606** ☑ The attribute **height** on a *Plot* must have a non-negative value of data type **double**. (Reference: SED-ML Level 1 Version 5, Section 2.2.12.)

**21607** ☑ The attribute **width** on a *Plot* must have a non-negative value of data type **double**. (Reference: SED-ML Level 1 Version 5, Section 2.2.12.)

#### *Rules for Plot2D objects*

**21701** ☑ A Plot2D object may have the optional SED-ML Level 1 attributes **id**, **name**, and **metaid**. (Reference: SED-ML Level 1 Version 5, Section 2.1.4.)

**21702** ☑ A Plot2D object may have the optional SED-ML Level 1 subobjects for notes and annotations. (Reference: SED-ML Level 1 Version 5, Section 2.1.4.)

**21703** ☑ A Plot2D object may contain one and only one instance of each of the ListOfCurves and Axis elements. (Reference: SED-ML Level 1 Version 5, Section 2.2.12.1.)

**21704** ☑ Apart from the general notes and annotations subobjects permitted on all SED-ML objects, a ListOfCurves container object may only contain *AbstractCurve* objects. (Reference: SED-ML Level 1 Version 5, Section 2.2.12.1.)

**21705** ☑ A ListOfCurves object may have the optional SED-ML Level 1 attributes **id**, **name**, and **metaid**. (Reference: SED-ML Level 1 Version 5, Section 2.2.12.1.)

**21750** ★ The shape of the data referenced by every *AbstractCurve* child of a single Plot2D object should be consistent. (Reference: SED-ML Level 1 Version 5, Section 2.2.12.1)

#### *Rules for Plot3D objects*

**21801** ☑ A Plot3D object may have the optional SED-ML Level 1 attributes **id**, **name**, and **metaid**. (Reference: SED-ML Level 1 Version 5, Section 2.1.4.)

**21802** ☑ A Plot3D object may have the optional SED-ML Level 1 subobjects for notes and annotations. (Reference: SED-ML Level 1 Version 5, Section 2.1.4.)

**21803** ☑ A Plot3D object may contain one and only one instance of each of the ListOfSurfaces and Axis elements. (Reference: SED-ML Level 1 Version 5, Section 2.2.12.2.)

**21804** ☑ Apart from the general notes and annotations subobjects permitted on all SED-ML objects, a ListOfSurfaces container object may only contain Surface objects. (Reference: SED-ML Level 1 Version 5, Section 2.2.12.2.)

**21805** ☑ A ListOfSurfaces object may have the optional SED-ML Level 1 attributes **id**, **name**, and **metaid**. (Reference: SED-ML Level 1 Version 5, Section 2.2.12.2.)

**21850** ★ The shape of the data referenced by every Surface child of a single Plot3D object should be consistent. (Reference: SED-ML Level 1 Version 5, Section 2.2.12.2)

***Rules for* AbstractCurve *objects***

**21901** ☑ An *AbstractCurve* object may have the optional SED-ML Level 1 attributes `id`, `name`, and `metaid`. (Reference: SED-ML Level 1 Version 5, Section 2.1.4.)

**21902** ☑ An *AbstractCurve* object may have the optional SED-ML Level 1 subobjects for notes and annotations. (Reference: SED-ML Level 1 Version 5, Section 2.1.4.)

**21903** ☑ An *AbstractCurve* object must have the required attribute `xDataReference`, and may have the optional attributes `logX`, `order`, `style` and `yAxis`. (Reference: SED-ML Level 1 Version 5, Section 2.2.12.5.)

**21904** ☑ The value of the attribute `xDataReference` of an *AbstractCurve* object must be the identifier of an existing DataReference object defined in the document. (Reference: SED-ML Level 1 Version 5, Section 2.2.12.5.)

**21905** ☑ The attribute `logX` on an *AbstractCurve* must have a value of data type `boolean`. (Reference: SED-ML Level 1 Version 5, Section 2.2.12.5.)

**21906** ☑ The attribute `order` on an *AbstractCurve* must have a value of data type `integer`. (Reference: SED-ML Level 1 Version 5, Section 2.2.12.5.)

**21907** ☑ The value of the attribute `style` of an *AbstractCurve* object must be the identifier of an existing Style object defined in the document. (Reference: SED-ML Level 1 Version 5, Section 2.2.12.5.)

**21908** ☑ The attribute `yAxis` on an *AbstractCurve* must have a value of data type `string`. (Reference: SED-ML Level 1 Version 5, Section 2.2.12.5.)

**21950** ▲ No `logX` attribute of any *AbstractCurve* should be set. Instead, the `type` attribute of the corresponding Axis should be used. (Reference: SED-ML Level 1 Version 5, Section 2.2.12.4)

***Rules for Curve objects***

**22001** ☑ A Curve object may have the optional SED-ML Level 1 attributes `id`, `name`, and `metaid`. (Reference: SED-ML Level 1 Version 5, Section 2.1.4.)

**22002** ☑ A Curve object may have the optional SED-ML Level 1 subobjects for notes and annotations. (Reference: SED-ML Level 1 Version 5, Section 2.1.4.)

**22003** ☑ A Curve object must have the required attributes `yDataReference` and `type`, and may have the optional attributes `logY`, `xErrorUpper`, `xErrorLower`, `yErrorUpper` and `yErrorLower`. (Reference: SED-ML Level 1 Version 5, Section 2.2.12.5.)

**22004** ☑ The value of the attribute `yDataReference` of a Curve object must be the identifier of an existing DataGenerator object defined in the document. (Reference: SED-ML Level 1 Version 5, Section 2.2.12.5.)

**22005** ☑ The value of the attribute `type` of a Curve object must conform to the syntax of SED-ML data type `CurveType` and may only take on the allowed values of `CurveType` defined in SED-ML; that is, the value must be one of the following: "`points`", "`bar`", "`barStacked`", "`horizontalBar`" or "`horizontalBarStacked`". (Reference: SED-ML Level 1 Version 5, Section 2.2.12.5.)

**22006** ☑ The attribute `logY` on a Curve must have a value of data type `boolean`. (Reference: SED-ML Level 1 Version 5, Section 2.2.12.5.)

**22007** ☑ The value of the attribute `xErrorUpper` of a Curve object must be the identifier of an existing DataGenerator object defined in the document. (Reference: SED-ML Level 1 Version 5, Section 2.2.12.5.)

**22008** ☑ The value of the attribute `xErrorLower` of a Curve object must be the identifier of an existing DataGenerator object defined in the document. (Reference: SED-ML Level 1 Version 5, Section 2.2.12.5.)

**22009** ☑ The value of the attribute `yErrorUpper` of a Curve object must be the identifier of an existing DataGenerator object defined in the document. (Reference: SED-ML Level 1 Version 5, Section 2.2.12.5.)

**22010** ☑ The value of the attribute `yErrorLower` of a Curve object must be the identifier of an existing DataGenerator object defined in the document. (Reference: SED-ML Level 1 Version 5, Section 2.2.12.5.)

**22050** ▲ No `logY` attribute of any Curve should be set. Instead, the `type` attribute of the corresponding Axis should be used. (Reference: SED-ML Level 1 Version 5, Section 2.2.12.5)

**22051** ☑ If defined, the `xErrorUpper` attribute must reference data with the same dimensionality as that referenced by the `xDataReference` attribute. (Reference: SED-ML Level 1 Version 5, Section 2.2.12.5)

**22052** ☑ If defined, the `xErrorLower` attribute must reference data with the same dimensionality as that referenced by the `xDataReference` attribute. (Reference: SED-ML Level 1 Version 5, Section 2.2.12.5)

**22053** ☑ If defined, the `yErrorUpper` attribute must reference data with the same dimensionality as that referenced by the `yDataReference` attribute. (Reference: SED-ML Level 1 Version 5, Section 2.2.12.5)

**22054** ☑ If defined, the `yErrorLower` attribute must reference data with the same dimensionality as that referenced by the `yDataReference` attribute. (Reference: SED-ML Level 1 Version 5, Section 2.2.12.5)

#### *Rules for Surface objects*

**22101** ☑ A Surface object may have the optional SED-ML Level 1 attributes `id`, `name`, and `metaid`. (Reference: SED-ML Level 1 Version 5, Section 2.1.4.)

**22102** ☑ A Surface object may have the optional SED-ML Level 1 subobjects for notes and annotations. (Reference: SED-ML Level 1 Version 5, Section 2.1.4.)

**22103** ☑ A Surface object must have the required attributes `xDataReference`, `yDataReference`, `zDataReference` and `type`, and may have the optional attributes `style`, `logX`, `logY`, `logZ` and `order`. (Reference: SED-ML Level 1 Version 5, Section 2.2.12.7.)

**22104** ☑ The value of the attribute `xDataReference` of a Surface object must be the identifier of an existing DataGenerator object defined in the document. (Reference: SED-ML Level 1 Version 5, Section 2.2.12.7.)

**22105** ☑ The value of the attribute `yDataReference` of a Surface object must be the identifier of an existing DataGenerator object defined in the document. (Reference: SED-ML Level 1 Version 5, Section 2.2.12.7.)

**22106** ☑ The value of the attribute `zDataReference` of a Surface object must be the identifier of an existing DataGenerator object defined in the document. (Reference: SED-ML Level 1 Version 5, Section 2.2.12.7.)

**22107** ☑ The value of the attribute `type` of a Surface object must conform to the syntax of SED-ML data type `SurfaceType` and may only take on the allowed values of `SurfaceType` defined in SED-ML; that is, the value must be one of the following: "`parametricCurve`", "`surfaceMesh`", "`surfaceContour`", "`contour`", "`heatMap`", "`stackedCurves`" or "`bar`". (Reference: SED-ML Level 1 Version 5, Section 2.2.12.7.)

**22108** ☑ The value of the attribute `style` of a Surface object must be the identifier of an existing Style object defined in the document. (Reference: SED-ML Level 1 Version 5, Section 2.2.12.7.)

**22109** ☑ The attribute `logX` on a Surface must have a value of data type `boolean`. (Reference: SED-ML Level 1 Version 5, Section 2.2.12.7.)

22110 ☑ The attribute **logY** on a Surface must have a value of data type **boolean**. (Reference: SED-ML Level 1 Version 5, Section 2.2.12.7.)

22111 ☑ The attribute **logZ** on a Surface must have a value of data type **boolean**. (Reference: SED-ML Level 1 Version 5, Section 2.2.12.7.)

22112 ☑ The attribute **order** on a Surface must have a value of data type **integer**. (Reference: SED-ML Level 1 Version 5, Section 2.2.12.7.)

22150 ▲ No **logX**, **logY**, or **logZ** attribute of any Surface should be set. Instead, the **type** attribute of the corresponding Axis should be used. (Reference: SED-ML Level 1 Version 5, Section 2.2.12.4)

#### *Rules for DataSet objects*

22201 ☑ A DataSet object may have the optional SED-ML Level 1 attributes **id**, **name**, and **metaid**. (Reference: SED-ML Level 1 Version 5, Section 2.1.4.)

22202 ☑ A DataSet object may have the optional SED-ML Level 1 subobjects for notes and annotations. (Reference: SED-ML Level 1 Version 5, Section 2.1.4.)

22203 ☑ A DataSet object must have the required attributes **label** and **dataReference**. (Reference: SED-ML Level 1 Version 5, Section 2.2.13.1.)

22204 ☑ The attribute **label** on a DataSet must have a value of data type **string**. (Reference: SED-ML Level 1 Version 5, Section 2.2.13.1.)

22205 ☑ The value of the attribute **dataReference** of a DataSet object must be the identifier of an existing DataGenerator object defined in the document. (Reference: SED-ML Level 1 Version 5, Section 2.2.13.1.)

22250 ☑ The **label** attributes of the DataSet children of a single Report must be unique. (Reference: SED-ML Level 1 Version 5, Section 2.2.13.1)

#### *Rules for Report objects*

22301 ☑ A Report object may have the optional SED-ML Level 1 attributes **id**, **name**, and **metaid**. (Reference: SED-ML Level 1 Version 5, Section 2.1.4.)

22302 ☑ A Report object may have the optional SED-ML Level 1 subobjects for notes and annotations. (Reference: SED-ML Level 1 Version 5, Section 2.1.4.)

22303 ☑ A Report object may contain one and only one instance of the ListOfDataSets element. (Reference: SED-ML Level 1 Version 5, Section 2.2.13.)

22304 ☑ Apart from the general notes and annotations subobjects permitted on all SED-ML objects, a ListOfDataSets container object may only contain DataSet objects. (Reference: SED-ML Level 1 Version 5, Section 2.2.13.)

22305 ☑ A ListOfDataSets object may have the optional SED-ML Level 1 attributes **id**, **name**, and **metaid**. (Reference: SED-ML Level 1 Version 5, Section 2.2.13.)

22350 ★ The shape of the output of every DataSet child of the same Report should be consistent. (Reference: SED-ML Level 1 Version 5, Section 2.2.13)

#### *Rules for AlgorithmParameter objects*

22401 ☑ An AlgorithmParameter object may have the optional SED-ML Level 1 attributes **id**, **name**, and **metaid**. (Reference: SED-ML Level 1 Version 5, Section 2.1.4.)

22402 ☑ An AlgorithmParameter object may have the optional SED-ML Level 1 subobjects for notes and annotations. (Reference: SED-ML Level 1 Version 5, Section 2.1.4.)

22403 ☑ An AlgorithmParameter object must have the required attributes **kisaoID** and **value**. (Reference: SED-ML Level 1 Version 5, Section 2.1.7.1.)

**22404** ☑ An AlgorithmParameter object may contain one and only one instance of the ListOfAlgorithmParameters element. (Reference: SED-ML Level 1 Version 5, Section 2.1.7.1.)

**22405** ☑ The attribute `kisaoID` on an AlgorithmParameter must have a value of data type `string`. (Reference: SED-ML Level 1 Version 5, Section 2.1.7.1.)

**22406** ☑ The attribute `value` on an AlgorithmParameter must have a value of data type `string`. (Reference: SED-ML Level 1 Version 5, Section 2.1.7.1.)

**22407** ☑ Apart from the general notes and annotations subobjects permitted on all SED-ML objects, a ListOfAlgorithmParameters container object may only contain AlgorithmParameter objects. (Reference: SED-ML Level 1 Version 5, Section 2.1.7.)

**22408** ☑ A ListOfAlgorithmParameters object may have the optional SED-ML Level 1 attributes `id`, `name`, and `metaid`. (Reference: SED-ML Level 1 Version 5, Section 2.1.7.)

**22450** ☑ The value of the `kisaoID` attribute of an AlgorithmParameter must be the ID of an algorithm parameter in the KiSAO ontology that is associated with the `kisaoID` of its parent Algorithm. (Reference: SED-ML Level 1 Version 5, Section 2.1.7.1)

**22451** ☑ The value of the every `kisaoID` attribute of the AlgorithmParameter children of a single Algorithm must be unique. (Reference: SED-ML Level 1 Version 5, Section 2.1.7.1)

##### *Rules for Range objects*

**22501** ☑ A Range object may have the optional SED-ML Level 1 attributes `id`, `name`, and `metaid`. (Reference: SED-ML Level 1 Version 5, Section 2.1.4.)

**22502** ☑ A Range object may have the optional SED-ML Level 1 subobjects for notes and annotations. (Reference: SED-ML Level 1 Version 5, Section 2.1.4.)

##### *Rules for ChangeXML objects*

**22601** ☑ A ChangeXML object may have the optional SED-ML Level 1 attributes `id`, `name`, and `metaid`. (Reference: SED-ML Level 1 Version 5, Section 2.1.4.)

**22602** ☑ A ChangeXML object may have the optional SED-ML Level 1 subobjects for notes and annotations. (Reference: SED-ML Level 1 Version 5, Section 2.1.4.)

**22603** ☑ A ChangeXML object may contain one and only one instance of the XMLNode element. (Reference: SED-ML Level 1 Version 5, Section 2.2.5.3.)

**22650** ☑ The `target` attribute of a ChangeXML object must point to a valid target in the Model `source`. (Reference: SED-ML Level 1 Version 5, Section 2.2.5)

**22651** ☑ The `target` attribute of a ChangeXML object be a valid XPath when the Model `language` attribute describes an XML-based language. (Reference: SED-ML Level 1 Version 5, Section 2.2.5)

**22652** ☑ The XML child of an ChangeXML object must be a valid XML element or list of XML elements. (Reference: SED-ML Level 1 Version 5, Section 2.2.5.3)

**22653** ☑ The XML child of an ChangeXML object must be in a namespace defined by the `source` attribute of the parent Model object, or explicitly define its own namespace understood by the language of the target model. (Reference: SED-ML Level 1 Version 5, Section 2.2.5.3)

##### *Rules for RemoveXML objects*

**22701** ☑ A RemoveXML object may have the optional SED-ML Level 1 attributes `id`, `name`, and `metaid`. (Reference: SED-ML Level 1 Version 5, Section 2.1.4.)

**22702** ☑ A RemoveXML object may have the optional SED-ML Level 1 subobjects for notes and annotations. (Reference: SED-ML Level 1 Version 5, Section 2.1.4.)

**22750** ☑ The `target` attribute of a RemoveXML object must point to a valid target in the Model `source`. (Reference: SED-ML Level 1 Version 5, Section 2.2.5)

22751 ☑ The `target` attribute of a RemoveXML object be a valid XPath when the Model `language` attribute describes an XML-based language. (Reference: SED-ML Level 1 Version 5, Section 2.2.5)

22752 ☑ The XML child of an RemoveXML object must be a valid XML element or list of XML elements. (Reference: SED-ML Level 1 Version 5, Section 2.2.5.4)

22753 ☑ The XML child of an RemoveXML object must be in a namespace defined by the `source` attribute of the parent Model object, or explicitly define its own namespace understood by the language of the target model. (Reference: SED-ML Level 1 Version 5, Section 2.2.5.4)

#### *Rules for SetValue objects*

22801 ☑ A SetValue object may have the optional SED-ML Level 1 attributes `id`, `name`, and `metaid`. (Reference: SED-ML Level 1 Version 5, Section 2.1.4.)

22802 ☑ A SetValue object may have the optional SED-ML Level 1 subobjects for notes and annotations. (Reference: SED-ML Level 1 Version 5, Section 2.1.4.)

22803 ☑ A SetValue object must have the required attribute `modelReference`, and may have the optional attributes `range`, `symbol` and `target`. (Reference: SED-ML Level 1 Version 5, Section 2.2.8.2.)

22804 ☑ A SetValue object may contain one and only one instance of each of the ASTNode, ListOfVariables and ListOfParameters elements. (Reference: SED-ML Level 1 Version 5, Section 2.2.8.2.)

22805 ☑ The value of the attribute `modelReference` of a SetValue object must be the identifier of an existing Model object defined in the document. (Reference: SED-ML Level 1 Version 5, Section 2.2.8.2.)

22806 ☑ The value of the `range` attribute of a SetValue must be the identifier of an existing Range child of the parent RepeatedTask. (Reference: SED-ML Level 1 Version 5, Section 2.2.8.2)

22807 ☑ The attribute `symbol` on a SetValue must have a value of data type `string`. (Reference: SED-ML Level 1 Version 5, Section 2.2.8.2.)

22808 ☑ The attribute `target` on a SetValue must have a value of data type `TargetType` (Reference: SED-ML Level 1 Version 5, Section 2.2.8.2)

22809 ☑ Apart from the general notes and annotations subobjects permitted on all SED-ML objects, a ListOfVariables container object may only contain Variable objects. (Reference: SED-ML Level 1 Version 5, Section 2.1.8.)

22810 ☑ Apart from the general notes and annotations subobjects permitted on all SED-ML objects, a ListOfParameters container object may only contain Parameter objects. (Reference: SED-ML Level 1 Version 5, Section 2.1.8.)

22811 ☑ A ListOfVariables object may have the optional SED-ML Level 1 attributes `id`, `name`, and `metaid`. (Reference: SED-ML Level 1 Version 5, Section 2.1.8.)

22812 ☑ A ListOfParameters object may have the optional SED-ML Level 1 attributes `id`, `name`, and `metaid`. (Reference: SED-ML Level 1 Version 5, Section 2.1.8.)

22850 ☑ If the `target` of a Variable child of a SetValue does not point to a DataSource, the Variable's `modelReference` must be set. (Reference: SED-ML Level 1 Version 5, Section 2.1.8.3)

22851 ☑ If the `target` of a Variable child of a SetValue does not point to a DataSource, the Variable's `taskReference`, if set, must reference the parent *AbstractTask*. (Reference: SED-ML Level 1 Version 5, Section 2.1.8.3)

***Rules for UniformRange objects***

**22901** ☑ An UniformRange object may have the optional SED-ML Level 1 attributes **id**, **name**, and **metaid**. (Reference: SED-ML Level 1 Version 5, Section 2.1.4.)

**22902** ☑ An UniformRange object may have the optional SED-ML Level 1 subobjects for notes and annotations. (Reference: SED-ML Level 1 Version 5, Section 2.1.4.)

**22903** ☑ An UniformRange object must have the required attributes **start**, **end** and **type**, and may have the optional attributes **numberOfPoints** and **numberOfSteps**. (Reference: SED-ML Level 1 Version 5, Section 2.2.8.3.1.)

**22904** ☑ The attribute **start** on an UniformRange must have a value of data type **double**. (Reference: SED-ML Level 1 Version 5, Section 2.2.8.3.1.)

**22905** ☑ The attribute **end** on an UniformRange must have a value of data type **double**. (Reference: SED-ML Level 1 Version 5, Section 2.2.8.3.1.)

**22906** ☑ The attribute **type** on an UniformRange must have a value of data type **string**. (Reference: SED-ML Level 1 Version 5, Section 2.2.8.3.1.)

**22907** ☑ The attribute **numberOfPoints** on an UniformRange must have a positive value of data type **integer**. (Reference: SED-ML Level 1 Version 5, Section 2.2.8.3.1.)

**22908** ☑ The attribute **numberOfSteps** on an UniformRange must have a positive value of data type **integer**. (Reference: SED-ML Level 1 Version 5, Section 2.2.8.3.1.)

**22950** ▲ Avoid use of the **numberOfPoints** attribute of a UniformRange in favor of the **numberOfSteps** attribute. "Number of Steps" accurately reflects the meaning of the attribute. (Reference: SED-ML Level 1 Version 5, Section 2.2.8.3.1)

**22951** ★ The value of the **numberOfPoints** attribute of a UniformRange should typically be evenly divisible by five. When this is not the case, it often indicates that the modeler is unaware that the definition of the attribute is actually 'the number of points not including the initial state'. (Reference: SED-ML Level 1 Version 5, Section 2.2.8.3.1)

**22952** ☑ Only one of the attributes **numberOfPoints** or **numberOfSteps** may be defined on a UniformRange. (Reference: SED-ML Level 1 Version 5, Section 2.2.8.3.1)

***Rules for VectorRange objects***

**23001** ☑ A VectorRange object may have the optional SED-ML Level 1 attributes **id**, **name**, and **metaid**. (Reference: SED-ML Level 1 Version 5, Section 2.1.4.)

**23002** ☑ A VectorRange object may have the optional SED-ML Level 1 subobjects for notes and annotations. (Reference: SED-ML Level 1 Version 5, Section 2.1.4.)

**23003** ☑ A VectorRange object may have the optional attribute **value**. (Reference: SED-ML Level 1 Version 5, Section 2.2.8.3.2.)

**23004** ☑ The value of the attribute **value** of a VectorRange object must be an vector of values of type **double**. (Reference: SED-ML Level 1 Version 5, Section 2.2.8.3.2.)

***Rules for FunctionalRange objects***

**23101** ☑ A FunctionalRange object may have the optional SED-ML Level 1 attributes **id**, **name**, and **metaid**. (Reference: SED-ML Level 1 Version 5, Section 2.1.4.)

**23102** ☑ A FunctionalRange object may have the optional SED-ML Level 1 subobjects for notes and annotations. (Reference: SED-ML Level 1 Version 5, Section 2.1.4.)

**23103** ☑ A FunctionalRange object must have the required attribute **range**. (Reference: SED-ML Level 1 Version 5, Section 2.2.8.3.4.)

23104 ☑ A FunctionalRange object may contain one and only one instance of each of the ListOfVariables, ListOfParameters and ASTNode elements. (Reference: SED-ML Level 1 Version 5, Section 2.2.8.3.4.)

23105 ☑ The value of the `range` attribute of a FunctionalRange must be the identifier of an existing Range sibling of the FunctionalRange. (Reference: SED-ML Level 1 Version 5, Section 2.2.8.3.4)

23106 ☑ Apart from the general notes and annotations subobjects permitted on all SED-ML objects, a ListOfVariables container object may only contain Variable objects. (Reference: SED-ML Level 1 Version 5, Section 2.1.8.)

23107 ☑ Apart from the general notes and annotations subobjects permitted on all SED-ML objects, a ListOfParameters container object may only contain Parameter objects. (Reference: SED-ML Level 1 Version 5, Section 2.1.8.)

23108 ☑ A ListOfVariables object may have the optional SED-ML Level 1 attributes `id`, `name`, and `metaid`. (Reference: SED-ML Level 1 Version 5, Section 2.1.8.)

23109 ☑ A ListOfParameters object may have the optional SED-ML Level 1 attributes `id`, `name`, and `metaid`. (Reference: SED-ML Level 1 Version 5, Section 2.1.8.)

23150 ☑ There must not be circular dependencies in the calculation of functional ranges. No Variable child of a FunctionalRange may directly or indirectly reference the parent FunctionalRange. (Reference: SED-ML Level 1 Version 5, Section 2.2.8.3.4)

23151 ☑ The `modelReference` attribute of a Variable child of a FunctionalRange must be defined if the `target` does not point to external data and the parent FunctionalRange is a child of an *AbstractTask* that involves more than one Model. (Reference: SED-ML Level 1 Version 5, Section 2.2.8.3.4)

#### *Rules for SubTask objects*

23201 ☑ A SubTask object may have the optional SED-ML Level 1 attributes `id`, `name`, and `metaid`. (Reference: SED-ML Level 1 Version 5, Section 2.1.4.)

23202 ☑ A SubTask object may have the optional SED-ML Level 1 subobjects for notes and annotations. (Reference: SED-ML Level 1 Version 5, Section 2.1.4.)

23203 ☑ A SubTask object must have the required attributes `order` and `task`. (Reference: SED-ML Level 1 Version 5, Section 2.2.8.1.)

23204 ☑ A SubTask object may contain one and only one instance of the ListOfChanges element. (Reference: SED-ML Level 1 Version 5, Section 2.2.8.1.)

23205 ☑ The attribute `order` on a SubTask must have a value of data type `integer`. (Reference: SED-ML Level 1 Version 5, Section 2.2.8.1.)

23206 ☑ The value of the attribute `task` of a SubTask object must be the identifier of an existing *AbstractTask* object defined in the document. (Reference: SED-ML Level 1 Version 5, Section 2.2.8.1.)

23207 ☑ Apart from the general notes and annotations subobjects permitted on all SED-ML objects, a ListOfChanges container object may only contain SetValue objects. (Reference: SED-ML Level 1 Version 5, Section 2.2.4.)

23208 ☑ A ListOfChanges object may have the optional SED-ML Level 1 attributes `id`, `name`, and `metaid`. (Reference: SED-ML Level 1 Version 5, Section 2.2.4.)

#### *Rules for OneStep objects*

23301 ☑ A OneStep object may have the optional SED-ML Level 1 attributes `id`, `name`, and `metaid`. (Reference: SED-ML Level 1 Version 5, Section 2.1.4.)

**23302** ☑ A OneStep object may have the optional SED-ML Level 1 subobjects for notes and annotations. (Reference: SED-ML Level 1 Version 5, Section 2.1.4.)

**23303** ☑ A OneStep object must have the required attribute `step`. (Reference: SED-ML Level 1 Version 5, Section 2.2.6.2.)

**23304** ☑ The attribute `step` on a OneStep must have a non-negative value of data type `double`. (Reference: SED-ML Level 1 Version 5, Section 2.2.6.2.)

#### *Rules for SteadyState objects*

**23401** ☑ A SteadyState object may have the optional SED-ML Level 1 attributes `id`, `name`, and `metaid`. (Reference: SED-ML Level 1 Version 5, Section 2.1.4.)

**23402** ☑ A SteadyState object may have the optional SED-ML Level 1 subobjects for notes and annotations. (Reference: SED-ML Level 1 Version 5, Section 2.1.4.)

#### *Rules for RepeatedTask objects*

**23501** ☑ A RepeatedTask object may have the required SED-ML Level 1 attribute `id` and the optional attributes `name` and `metaid`. (Reference: SED-ML Level 1 Version 5, Section 2.1.4.)

**23502** ☑ A RepeatedTask object may have the optional SED-ML Level 1 subobjects for notes and annotations. (Reference: SED-ML Level 1 Version 5, Section 2.1.4.)

**23503** ☑ A RepeatedTask object must have the required attributes `range` and `resetModel`, and may have the optional attribute `concatenate`. (Reference: SED-ML Level 1 Version 5, Section 2.2.7.2.)

**23504** ☑ A RepeatedTask object may contain one and only one instance of each of the ListOfRanges, ListOfChanges and ListOfSubTasks elements. (Reference: SED-ML Level 1 Version 5, Section 2.2.7.2.)

**23505** ☑ The value of the `range` of a RepeatedTask must be the identifier of an existing Range child of that RepeatedTask. (Reference: SED-ML Level 1 Version 5, Section 2.2.8.3)

**23506** ☑ The attribute `resetModel` on a RepeatedTask must have a value of data type `boolean`. (Reference: SED-ML Level 1 Version 5, Section 2.2.7.2.)

**23507** ☑ The attribute `concatenate` on a RepeatedTask must have a value of data type `boolean`. (Reference: SED-ML Level 1 Version 5, Section 2.2.7.2.)

**23508** ☑ Apart from the general notes and annotations subobjects permitted on all SED-ML objects, a ListOfRanges container object may only contain Range objects. (Reference: SED-ML Level 1 Version 5, Section 2.2.7.2.)

**23509** ☑ Apart from the general notes and annotations subobjects permitted on all SED-ML objects, a ListOfChanges container object may only contain SetValue objects. (Reference: SED-ML Level 1 Version 5, Section 2.2.4.)

**23510** ☑ Apart from the general notes and annotations subobjects permitted on all SED-ML objects, a ListOfSubTasks container object may only contain SubTask objects. (Reference: SED-ML Level 1 Version 5, Section 2.2.7.2.)

**23511** ☑ A ListOfRanges object may have the optional SED-ML Level 1 attributes `id`, `name`, and `metaid`. (Reference: SED-ML Level 1 Version 5, Section 2.2.7.2.)

**23512** ☑ A ListOfChanges object may have the optional SED-ML Level 1 attributes `id`, `name`, and `metaid`. (Reference: SED-ML Level 1 Version 5, Section 2.2.4.)

**23513** ☑ A ListOfSubTasks object may have the optional SED-ML Level 1 attributes `id`, `name`, and `metaid`. (Reference: SED-ML Level 1 Version 5, Section 2.2.7.2.)

**23550** ☑ There must not be circular dependencies in repeated tasks. The `task` attribute of a SubTask may not directly or indirectly reference its parent RepeatedTask. (Reference: SED-ML Level 1 Version 5, Section 2.2.7.2)

**23551** ☑ Every RepeatedTask must have exactly one ListOfRanges child containing at least one child Range object. (Reference: SED-ML Level 1 Version 5, Section 2.2.7.2)

**23552** ☑ Every RepeatedTask must have exactly one ListOfSubTasks child containing at least one child SubTask object. (Reference: SED-ML Level 1 Version 5, Section 2.2.7.2)

**23553** ☑ When a RepeatedTask has multiple Range children, they all must have at least as many entries as the one referenced by the `range` attribute. (Reference: SED-ML Level 1 Version 5, Section 2.2.7.2)

**23554** ★ The shape of the output of every SubTask child of the same RepeatedTask should be consistent. (Reference: SED-ML Level 1 Version 5, Section 2.2.7.2)

#### *Rules for ComputeChange objects*

**23601** ☑ A ComputeChange object may have the optional SED-ML Level 1 attributes `id`, `name`, and `metaid`. (Reference: SED-ML Level 1 Version 5, Section 2.1.4.)

**23602** ☑ A ComputeChange object may have the optional SED-ML Level 1 subobjects for notes and annotations. (Reference: SED-ML Level 1 Version 5, Section 2.1.4.)

**23603** ☑ A ComputeChange object may have the optional attribute `symbol`. (Reference: SED-ML Level 1 Version 5, Section 2.2.5.6.)

**23604** ☑ A ComputeChange object may contain one and only one instance of each of the ASTNode, Algorithm, ListOfVariables, and ListOfParameters elements. (Reference: SED-ML Level 1 Version 5, Section 2.2.5.6.)

**23605** ☑ The attribute `symbol` on a ComputeChange must have a value of data type `string`. (Reference: SED-ML Level 1 Version 5, Section 2.2.5.6.)

**23606** ☑ Apart from the general notes and annotations subobjects permitted on all SED-ML objects, a ListOfVariables container object may only contain Variable objects. (Reference: SED-ML Level 1 Version 5, Section 2.1.8.)

**23607** ☑ Apart from the general notes and annotations subobjects permitted on all SED-ML objects, a ListOfParameters container object may only contain Parameter objects. (Reference: SED-ML Level 1 Version 5, Section 2.1.8.)

**23608** ☑ A ListOfVariables object may have the optional SED-ML Level 1 attributes `id`, `name`, and `metaid`. (Reference: SED-ML Level 1 Version 5, Section 2.1.8.)

**23609** ☑ A ListOfParameters object may have the optional SED-ML Level 1 attributes `id`, `name`, and `metaid`. (Reference: SED-ML Level 1 Version 5, Section 2.1.8.)

**23650** ☑ The `target` attribute of a ComputeChange object must be a valid XPath when the Model `language` attribute describes an XML-based language. (Reference: SED-ML Level 1 Version 5, Section 2.2.5)

**23651** ☑ The `target` attribute of a ComputeChange object be a valid XPath when the Model `language` attribute describes an XML-based language. (Reference: SED-ML Level 1 Version 5, Section 2.2.5)

**23652** ☑ The `taskReference` attribute of a Variable child of a ComputeChange object must not be defined. (Reference: SED-ML Level 1 Version 5, Section 2.2.5)

#### *Rules for DataDescription objects*

**23701** ☑ A DataDescription object may have the required SED-ML Level 1 attribute `id` and the optional attributes `name` and `metaid`. (Reference: SED-ML Level 1 Version 5, Section 2.1.4.)

**23702** ☑ A DataDescription object may have the optional SED-ML Level 1 subobjects for notes and annotations. (Reference: SED-ML Level 1 Version 5, Section 2.1.4.)

23703 ☑ A DataDescription object must have the required attributes `source` and `id`, and may have the optional attribute `format`. (Reference: SED-ML Level 1 Version 5, Section 2.2.2.)

23704 ☑ A DataDescription object may contain one and only one instance of each of the ASTNode, DimensionDescription, and ListOfDataSources elements. (Reference: SED-ML Level 1 Version 5, Section 2.2.2.)

23705 ☑ The attribute `source` on a DataDescription must have a value of data type `anyURI` (Reference: SED-ML Level 1 Version 5, Section 2.2.2)

23706 ☑ The attribute `format` on a DataDescription must have a value of data type `URN` (Reference: SED-ML Level 1 Version 5, Section 2.2.2)

23707 ☑ Apart from the general notes and annotations subobjects permitted on all SED-ML objects, a ListOfDataSources container object may only contain DataSource objects. (Reference: SED-ML Level 1 Version 5, Section 2.2.2.)

23708 ☑ A ListOfDataSources object may have the optional SED-ML Level 1 attributes `id`, `name`, and `metaid`. (Reference: SED-ML Level 1 Version 5, Section 2.2.2.)

***Rules for DataSource objects***

23801 ☑ A DataSource object may have the required SED-ML Level 1 attribute `id` and the optional attributes `name` and `metaid`. (Reference: SED-ML Level 1 Version 5, Section 2.1.4.)

23802 ☑ A DataSource object may have the optional SED-ML Level 1 subobjects for notes and annotations. (Reference: SED-ML Level 1 Version 5, Section 2.1.4.)

23803 ☑ A DataSource object must have the required attributes `indexSet` and `id`. (Reference: SED-ML Level 1 Version 5, Section 2.2.3.2.)

23804 ☑ A DataSource object may contain one and only one instance of the ListOfSlices element. (Reference: SED-ML Level 1 Version 5, Section 2.2.3.2.)

23805 ☑ The value of the attribute `indexSet` of a DataSource object must be the identifier of an existing object defined in the document. (Reference: SED-ML Level 1 Version 5, Section 2.2.3.2.)

23806 ☑ Apart from the general notes and annotations subobjects permitted on all SED-ML objects, a ListOfSlices container object may only contain Slice objects. (Reference: SED-ML Level 1 Version 5, Section 2.2.3.2.)

23807 ☑ A ListOfSlices object may have the optional SED-ML Level 1 attributes `id`, `name`, and `metaid`. (Reference: SED-ML Level 1 Version 5, Section 2.2.3.2.)

***Rules for Slice objects***

23901 ☑ A Slice object may have the optional SED-ML Level 1 attributes `id`, `name`, and `metaid`. (Reference: SED-ML Level 1 Version 5, Section 2.1.4.)

23902 ☑ A Slice object may have the optional SED-ML Level 1 subobjects for notes and annotations. (Reference: SED-ML Level 1 Version 5, Section 2.1.4.)

23903 ☑ A Slice object must have the required attribute `reference`, and may have the optional attributes `value`, `index`, `startIndex` and `endIndex`. (Reference: SED-ML Level 1 Version 5, Section 2.2.3.3.)

23904 ☑ The value of the attribute `reference` of a Slice object must be the identifier of an existing object defined in the document. (Reference: SED-ML Level 1 Version 5, Section 2.2.3.3.)

23905 ☑ The attribute `value` on a Slice must have a value of data type `string`. (Reference: SED-ML Level 1 Version 5, Section 2.2.3.3.)

23906 ☑ The value of the attribute `index` of a Slice object must be the identifier of an existing RepeatedTask object defined in the document. (Reference: SED-ML Level 1 Version 5, Section 2.2.3.3.)

23907 ☑ The attribute **startIndex** on a Slice must have a value of data type **integer**. (Reference: SED-ML Level 1 Version 5, Section 2.2.3.3.)

23908 ☑ The attribute **endIndex** on a Slice must have a value of data type **integer**. (Reference: SED-ML Level 1 Version 5, Section 2.2.3.3.)

23950 ☑ If a Slice defines both an **startIndex** and an **endIndex**, the value of the **endIndex** must be equal to or greater than that of the **startIndex**. (Reference: SED-ML Level 1 Version 5, Section 2.2.3.3)

#### *Rules for ParameterEstimationTask objects*

24001 ☑ A ParameterEstimationTask object may have the required SED-ML Level 1 attribute **id** and the optional attributes **name** and **metaid**. (Reference: SED-ML Level 1 Version 5, Section 2.1.4.)

24002 ☑ A ParameterEstimationTask object may have the optional SED-ML Level 1 subobjects for notes and annotations. (Reference: SED-ML Level 1 Version 5, Section 2.1.4.)

24003 ☑ A ParameterEstimationTask object must have the required attribute **modelReference**. (Reference: SED-ML Level 1 Version 5, Section 2.2.9.)

24004 ☑ A ParameterEstimationTask object must contain one and only one instance of each of the Algorithm, Objective, ListOfAdjustableParameters and ListOfFitExperiments elements. (Reference: SED-ML Level 1 Version 5, Section 2.2.9.)

24005 ☑ The value of the attribute **modelReference** of a ParameterEstimationTask object must be the identifier of an existing Model object defined in the document. (Reference: SED-ML Level 1 Version 5, Section 2.2.9.)

24006 ☑ Apart from the general notes and annotations subobjects permitted on all SED-ML objects, a ListOfAdjustableParameters container object may only contain AdjustableParameter objects. (Reference: SED-ML Level 1 Version 5, Section 2.2.9.)

24007 ☑ Apart from the general notes and annotations subobjects permitted on all SED-ML objects, a ListOfFitExperiments container object may only contain FitExperiment objects. (Reference: SED-ML Level 1 Version 5, Section 2.2.9.)

24008 ☑ A ListOfAdjustableParameters object may have the optional SED-ML Level 1 attributes **id**, **name**, and **metaid**. (Reference: SED-ML Level 1 Version 5, Section 2.2.9.)

24009 ☑ A ListOfFitExperiments object may have the optional SED-ML Level 1 attributes **id**, **name**, and **metaid**. (Reference: SED-ML Level 1 Version 5, Section 2.2.9.)

24050 ☑ Every ParameterEstimationTask must have exactly one ListOfAdjustableParameters child containing at least one child AdjustableParameter object. (Reference: SED-ML Level 1 Version 5, Section 2.2.9)

24051 ☑ Every ParameterEstimationTask must have exactly one ListOfFitExperiments child containing at least one child FitExperiment object. (Reference: SED-ML Level 1 Version 5, Section 2.2.9)

#### *Rules for Objective objects*

24101 ☑ An Objective object may have the optional SED-ML Level 1 attributes **id**, **name**, and **metaid**. (Reference: SED-ML Level 1 Version 5, Section 2.1.4.)

24102 ☑ An Objective object may have the optional SED-ML Level 1 subobjects for notes and annotations. (Reference: SED-ML Level 1 Version 5, Section 2.1.4.)

#### *Rules for LeastSquareObjectiveFunction objects*

24201 ☑ A LeastSquareObjectiveFunction object may have the optional SED-ML Level 1 attributes **id**, **name**, and **metaid**. (Reference: SED-ML Level 1 Version 5, Section 2.1.4.)

24202 ☑ A LeastSquareObjectiveFunction object may have the optional SED-ML Level 1 subobjects for notes and annotations. (Reference: SED-ML Level 1 Version 5, Section 2.1.4.)

#### *Rules for AdjustableParameter objects*

**24301** ☑ An AdjustableParameter object may have the optional SED-ML Level 1 attributes `id`, `name`, and `metaid`. (Reference: SED-ML Level 1 Version 5, Section 2.1.4.)

**24302** ☑ An AdjustableParameter object may have the optional SED-ML Level 1 subobjects for notes and annotations. (Reference: SED-ML Level 1 Version 5, Section 2.1.4.)

**24303** ☑ An AdjustableParameter object must have the required attribute `target`, and may have the optional attributes `initialValue` and `modelReference`. (Reference: SED-ML Level 1 Version 5, Section 2.2.9.3.)

**24304** ☑ An AdjustableParameter object must contain one and only one instance of the Bounds element, and may contain one and only one instance of the ListOfExperimentReferences element. (Reference: SED-ML Level 1 Version 5, Section 2.2.9.3.)

**24305** ☑ The attribute `target` on an AdjustableParameter must have a value of data type `TargetType` (Reference: SED-ML Level 1 Version 5, Section 2.2.9.3)

**24306** ☑ The attribute `initialValue` on an AdjustableParameter must have a value of data type `double`. (Reference: SED-ML Level 1 Version 5, Section 2.2.9.3.)

**24307** ☑ The value of the attribute `modelReference` of an AdjustableParameter object must be the identifier of an existing Model object defined in the document. (Reference: SED-ML Level 1 Version 5, Section 2.2.9.3.)

**24308** ☑ Apart from the general notes and annotations subobjects permitted on all SED-ML objects, a ListOfExperimentReferences container object may only contain ExperimentReference objects. (Reference: SED-ML Level 1 Version 5, Section 2.2.9.3.)

**24309** ☑ A ListOfExperimentReferences object may have the optional SED-ML Level 1 attributes `id`, `name`, and `metaid`. (Reference: SED-ML Level 1 Version 5, Section 2.2.9.3.)

#### *Rules for ExperimentReference objects*

**24401** ☑ An ExperimentReference object may have the optional SED-ML Level 1 attributes `id`, `name`, and `metaid`. (Reference: SED-ML Level 1 Version 5, Section 2.1.4.)

**24402** ☑ An ExperimentReference object may have the optional SED-ML Level 1 subobjects for notes and annotations. (Reference: SED-ML Level 1 Version 5, Section 2.1.4.)

**24403** ☑ An ExperimentReference object must have the required attribute `experimentId`. (Reference: SED-ML Level 1 Version 5, Section 2.2.9.5.)

**24404** ☑ The value of the `experiment` attribute of a ExperimentReference must be the identifier of an existing FitExperiment child of the parent ParameterEstimationTask. (Reference: SED-ML Level 1 Version 5, Section 2.1.8.3)

#### *Rules for FitExperiment objects*

**24501** ☑ A FitExperiment object may have the optional SED-ML Level 1 attributes `id`, `name`, and `metaid`. (Reference: SED-ML Level 1 Version 5, Section 2.1.4.)

**24502** ☑ A FitExperiment object may have the optional SED-ML Level 1 subobjects for notes and annotations. (Reference: SED-ML Level 1 Version 5, Section 2.1.4.)

**24503** ☑ A FitExperiment object must have the required attribute `type`. (Reference: SED-ML Level 1 Version 5, Section 2.2.9.6.)

**24504** ☑ A FitExperiment object must contain one and only one instance of each of the Algorithm and ListOfFitMappings elements. (Reference: SED-ML Level 1 Version 5, Section 2.2.9.6.)

**24505** ☑ The value of the attribute `type` of a FitExperiment object must conform to the syntax of SED-ML data type `ExperimentType` and may only take on the allowed values of `ExperimentType` defined in SED-ML; that is, the value must be one of the following: "`steadyState`" or "`timeCourse`". (Reference: SED-ML Level 1 Version 5, Section 2.2.9.6.)

**24506** ☑ Apart from the general notes and annotations subobjects permitted on all SED-ML objects, a ListOfFitMappings container object may only contain FitMapping objects. (Reference: SED-ML Level 1 Version 5, Section 2.2.9.6.)

**24507** ☑ A ListOfFitMappings object may have the optional SED-ML Level 1 attributes `id`, `name`, and `metaid`. (Reference: SED-ML Level 1 Version 5, Section 2.2.9.6.)

**24550** ☑ Every FitExperiment must have exactly one ListOfFitMappings child containing at least one child FitMapping object. (Reference: SED-ML Level 1 Version 5, Section 2.2.9.6)

#### *Rules for FitMapping objects*

**24601** ☑ A FitMapping object may have the optional SED-ML Level 1 attributes `id`, `name`, and `metaid`. (Reference: SED-ML Level 1 Version 5, Section 2.1.4.)

**24602** ☑ A FitMapping object may have the optional SED-ML Level 1 subobjects for notes and annotations. (Reference: SED-ML Level 1 Version 5, Section 2.1.4.)

**24603** ☑ A FitMapping object must have the required attributes `dataSource`, `target` and `type`, and may have the optional attributes `weight` and `pointWeight`. (Reference: SED-ML Level 1 Version 5, Section 2.2.9.7.)

**24604** ☑ The value of the attribute `dataSource` of a FitMapping object must be the identifier of an existing DataSource object defined in the document. (Reference: SED-ML Level 1 Version 5, Section 2.2.9.7.)

**24605** ☑ The value of the attribute `target` of a FitMapping object must be the identifier of an existing DataGenerator object defined in the document. (Reference: SED-ML Level 1 Version 5, Section 2.2.9.7.)

**24606** ☑ The value of the attribute `type` of a FitMapping object must conform to the syntax of SED-ML data type `MappingType` and may only take on the allowed values of `MappingType` defined in SED-ML; that is, the value must be one of the following: "`time`", "`experimentalCondition`" or "`observable`". (Reference: SED-ML Level 1 Version 5, Section 2.2.9.7.)

**24607** ☑ The attribute `weight` on a FitMapping must have a value of data type `double`. (Reference: SED-ML Level 1 Version 5, Section 2.2.9.7.)

**24608** ☑ The value of the `pointWeight` attribute of a FitMapping must be the identifier of an existing DataGenerator or DataSource object in the document. (Reference: SED-ML Level 1 Version 5, Section 2.2.9.7)

**24650** ☑ A FitMapping object may not define both its `weight` attribute and its `pointWeight` attribute, but may leave both undefined. (Reference: SED-ML Level 1 Version 5, Section 2.2.9.7)

**24651** ☑ The value of every element referenced by the `pointWeight` attribute of a FitMapping object must either be non-negative or defined as `notanumber` for missing data. No element may be set to "`infinity`". (Reference: SED-ML Level 1 Version 5, Section 2.2.9.7)

**24652** ☑ The value of the `weight` attribute of a FitMapping object must not be infinite or negative. (Reference: SED-ML Level 1 Version 5, Section 2.2.9.7)

**24653** ★ The value of every element referenced by the `pointWeight` attribute of a FitMapping should typically fall between 0 and 1, inclusive. (Reference: SED-ML Level 1 Version 5, Section 2.2.9.7)

**24654** ★ The `pointWeight` attribute of a FitMapping, if defined, must point to a DataGenerator or DataSource with the same dimensionality as the data referenced by the `dataSource` attribute. (Reference: SED-ML Level 1 Version 5, Section 2.2.9.7)

***Rules for Bounds objects***

**24701** ☑ A Bounds object may have the optional SED-ML Level 1 attributes `id`, `name`, and `metaid`. (Reference: SED-ML Level 1 Version 5, Section 2.1.4.)

**24702** ☑ A Bounds object may have the optional SED-ML Level 1 subobjects for notes and annotations. (Reference: SED-ML Level 1 Version 5, Section 2.1.4.)

**24703** ☑ A Bounds object must have the required attributes `lowerBound`, `upperBound` and `scale`. (Reference: SED-ML Level 1 Version 5, Section 2.2.9.4.)

**24704** ☑ The attribute `lowerBound` on a Bounds must have a value of data type `double`. (Reference: SED-ML Level 1 Version 5, Section 2.2.9.4.)

**24705** ☑ The attribute `upperBound` on a Bounds must have a value of data type `double`. (Reference: SED-ML Level 1 Version 5, Section 2.2.9.4.)

**24706** ☑ The value of the attribute `scale` of a Bounds object must conform to the syntax of SED-ML data type `ScaleType` and may only take on the allowed values of `ScaleType` defined in SED-ML; that is, the value must be one of the following: "`linear`", "`log`" or "`log10`". (Reference: SED-ML Level 1 Version 5, Section 2.2.9.4.)

**24750** ☑ The value of the `upperBound` attribute of a Bounds object must be greater than or equal to the value of the `lowerBound` attribute. (Reference: SED-ML Level 1 Version 5, Section 2.2.9.4)

***Rules for Figure objects***

**24801** ☑ A Figure object may have the optional SED-ML Level 1 attributes `id`, `name`, and `metaid`. (Reference: SED-ML Level 1 Version 5, Section 2.1.4.)

**24802** ☑ A Figure object may have the optional SED-ML Level 1 subobjects for notes and annotations. (Reference: SED-ML Level 1 Version 5, Section 2.1.4.)

**24803** ☑ A Figure object must have the required attributes `numRows` and `numCols`. (Reference: SED-ML Level 1 Version 5, Section 2.2.15.)

**24804** ☑ A Figure object may contain one and only one instance of the ListOfSubPlots element. (Reference: SED-ML Level 1 Version 5, Section 2.2.15.)

**24805** ☑ The attribute `numRows` on a Figure must have a value of data type `integer`. (Reference: SED-ML Level 1 Version 5, Section 2.2.15.)

**24806** ☑ The attribute `numCols` on a Figure must have a value of data type `integer`. (Reference: SED-ML Level 1 Version 5, Section 2.2.15.)

**24807** ☑ Apart from the general notes and annotations subobjects permitted on all SED-ML objects, a ListOfSubPlots container object may only contain SubPlot objects. (Reference: SED-ML Level 1 Version 5, Section 2.2.15.)

**24808** ☑ A ListOfSubPlots object may have the optional SED-ML Level 1 attributes `id`, `name`, and `metaid`. (Reference: SED-ML Level 1 Version 5, Section 2.2.15.)

***Rules for SubPlot objects***

**24901** ☑ A SubPlot object may have the optional SED-ML Level 1 attributes `id`, `name`, and `metaid`. (Reference: SED-ML Level 1 Version 5, Section 2.1.4.)

**24902** ☑ A SubPlot object may have the optional SED-ML Level 1 subobjects for notes and annotations. (Reference: SED-ML Level 1 Version 5, Section 2.1.4.)

**24903** ☑ A SubPlot object must have the required attributes `plot`, `row` and `col`, and may have the optional attributes `rowSpan` and `colSpan`. (Reference: SED-ML Level 1 Version 5, Section 2.2.15.1.)

**24904** ☑ The value of the attribute `plot` of a SubPlot object must be the identifier of an existing *Plot* object defined in the document. (Reference: SED-ML Level 1 Version 5, Section 2.2.15.1.)

**24905** ☑ The attribute `row` on a SubPlot must have a positive value of data type `integer`. (Reference: SED-ML Level 1 Version 5, Section 2.2.15.1.)

**24906** ☑ The attribute `col` on a SubPlot must have a positive value of data type `integer`. (Reference: SED-ML Level 1 Version 5, Section 2.2.15.1.)

**24907** ☑ The attribute `rowSpan` on a SubPlot must have a positive value of data type `integer`. (Reference: SED-ML Level 1 Version 5, Section 2.2.15.1.)

**24908** ☑ The attribute `colSpan` on a SubPlot must have a positive value of data type `integer`. (Reference: SED-ML Level 1 Version 5, Section 2.2.15.1.)

**24950** ☑ If defined, the `rowSpan` attribute of a SubPlot must have a value greater than or equal to one, and less than or equal to one plus the `numRows` attribute of the parent Figure minus the `row` attribute of the SubPlot. (Reference: SED-ML Level 1 Version 5, Section 2.2.15.1)

**24951** ☑ If defined, the `colSpan` attribute of a SubPlot must have a value greater than or equal to one, and less than or equal to one plus the `numCols` attribute of the parent Figure minus the `col` attribute of the SubPlot. (Reference: SED-ML Level 1 Version 5, Section 2.2.15.1)

#### *Rules for Axis objects*

**25001** ☑ An Axis object may have the optional SED-ML Level 1 attributes `id`, `name`, and `metaid`. (Reference: SED-ML Level 1 Version 5, Section 2.1.4.)

**25002** ☑ An Axis object may have the optional SED-ML Level 1 subobjects for notes and annotations. (Reference: SED-ML Level 1 Version 5, Section 2.1.4.)

**25003** ☑ An Axis object must have the required attribute `type`, and may have the optional attributes `min`, `max`, `grid`, `style` and `reverse`. (Reference: SED-ML Level 1 Version 5, Section 2.2.12.3.)

**25004** ☑ The value of the attribute `type` of an Axis object must conform to the syntax of SED-ML data type `AxisType` and may only take on the allowed values of `AxisType` defined in SED-ML; that is, the value must be one of the following: "`linear`" or "`log10`". (Reference: SED-ML Level 1 Version 5, Section 2.2.12.3.)

**25005** ☑ The attribute `min` on an Axis must have a value of data type `double`. (Reference: SED-ML Level 1 Version 5, Section 2.2.12.3.)

**25006** ☑ The attribute `max` on an Axis must have a value of data type `double`. (Reference: SED-ML Level 1 Version 5, Section 2.2.12.3.)

**25007** ☑ The attribute `grid` on an Axis must have a value of data type `boolean`. (Reference: SED-ML Level 1 Version 5, Section 2.2.12.3.)

**25008** ☑ The value of the attribute `style` of an Axis object must be the identifier of an existing Style object defined in the document. (Reference: SED-ML Level 1 Version 5, Section 2.2.12.3.)

**25009** ☑ The attribute `reverse` on an Axis must have a value of data type `boolean`. (Reference: SED-ML Level 1 Version 5, Section 2.2.12.3.)

**25050** ☑ If an Axis defines both its `max` attribute and its `min` attribute, the value of the `max` must be greater than or equal to the value of the `min` attribute. (Reference: SED-ML Level 1 Version 5, Section 2.2.12.3)

***Rules for Style objects***

**25101** ☑ A Style object may have the required SED-ML Level 1 attribute `id` and the optional attributes `name` and `metaid`. (Reference: SED-ML Level 1 Version 5, Section 2.1.4.)

**25102** ☑ A Style object may have the optional SED-ML Level 1 subobjects for notes and annotations. (Reference: SED-ML Level 1 Version 5, Section 2.1.4.)

**25103** ☑ A Style object must have the required attribute `id`, and may have the optional attribute `baseStyle`. (Reference: SED-ML Level 1 Version 5, Section 2.2.16.)

**25104** ☑ A Style object may contain one and only one instance of each of the Line, Marker and Fill elements. (Reference: SED-ML Level 1 Version 5, Section 2.2.16.)

**25105** ☑ The value of the attribute `baseStyle` of a Style object must be the identifier of an existing Style object defined in the document. (Reference: SED-ML Level 1 Version 5, Section 2.2.16.)

***Rules for Line objects***

**25201** ☑ A Line object may have the optional SED-ML Level 1 attributes `id`, `name`, and `metaid`. (Reference: SED-ML Level 1 Version 5, Section 2.1.4.)

**25202** ☑ A Line object may have the optional SED-ML Level 1 subobjects for notes and annotations. (Reference: SED-ML Level 1 Version 5, Section 2.1.4.)

**25203** ☑ A Line object may have the optional attributes `type`, `color` and `thickness`. (Reference: SED-ML Level 1 Version 5, Section 2.2.16.1.)

**25204** ☑ The value of the attribute `type` of a Line object must conform to the syntax of SED-ML data type `LineType` and may only take on the allowed values of `LineType` defined in SED-ML; that is, the value must be one of the following: "`none`", "`solid`", "`dash`", "`dot`", "`dashDot`" or "`dashDotDot`". (Reference: SED-ML Level 1 Version 5, Section 2.2.16.1.)

**25205** ☑ The attribute `color` on a Line must have a value of data type `SedColor` (Reference: SED-ML Level 1 Version 5, Section 2.2.16.1)

**25206** ☑ The attribute `thickness` on a Line must have a non-negative value of data type `double`. (Reference: SED-ML Level 1 Version 5, Section 2.2.16.1.)

***Rules for Marker objects***

**25301** ☑ A Marker object may have the optional SED-ML Level 1 attributes `id`, `name`, and `metaid`. (Reference: SED-ML Level 1 Version 5, Section 2.1.4.)

**25302** ☑ A Marker object may have the optional SED-ML Level 1 subobjects for notes and annotations. (Reference: SED-ML Level 1 Version 5, Section 2.1.4.)

**25303** ☑ A Marker object may have the optional attributes `size`, `type`, `fill`, `lineColor` and `lineThickness`. (Reference: SED-ML Level 1 Version 5, Section 2.2.16.2.)

**25304** ☑ The attribute `size` on a Marker must have a non-negative value of data type `double`. (Reference: SED-ML Level 1 Version 5, Section 2.2.16.2.)

**25305** ☑ The value of the attribute `type` of a Marker object must conform to the syntax of SED-ML data type `MarkerType` and may only take on the allowed values of `MarkerType` defined in SED-ML; that is, the value must be one of the following: "`none`", "`square`", "`circle`", "`diamond`", "`xCross`", "`plus`", "`star`", "`triangleUp`", "`triangleDown`", "`triangleLeft`", "`triangleRight`", "`hDash`" or "`vDash`". (Reference: SED-ML Level 1 Version 5, Section 2.2.16.2.)

**25306** ☑ The attribute `fill` on a Marker must have a value of data type `SedColor` (Reference: SED-ML Level 1 Version 5, Section 2.2.16.2)

**25307** ☑ The attribute `lineColor` on a Marker must have a value of data type `SedColor` (Reference: SED-ML Level 1 Version 5, Section 2.2.16.2)

**25308** ☑ The attribute `lineThickness` on a Marker must have a non-negative value of data type `double`. (Reference: SED-ML Level 1 Version 5, Section 2.2.16.2.)

#### *Rules for Fill objects*

**25401** ☑ A Fill object may have the optional SED-ML Level 1 attributes `id`, `name`, and `metaid`. (Reference: SED-ML Level 1 Version 5, Section 2.1.4.)

**25402** ☑ A Fill object may have the optional SED-ML Level 1 subobjects for notes and annotations. (Reference: SED-ML Level 1 Version 5, Section 2.1.4.)

**25403** ☑ A Fill object must have the required attribute `color`, and may have the optional attribute `secondColor`. (Reference: SED-ML Level 1 Version 5, Section 2.2.16.3.)

**25404** ☑ The attribute `color` on a Fill must have a value of data type `SedColor` (Reference: SED-ML Level 1 Version 5, Section 2.2.16.3)

#### *Rules for AppliedDimension objects*

**25501** ☑ An AppliedDimension object may have the optional SED-ML Level 1 attributes `id`, `name`, and `metaid`. (Reference: SED-ML Level 1 Version 5, Section 2.1.4.)

**25502** ☑ An AppliedDimension object may have the optional SED-ML Level 1 subobjects for notes and annotations. (Reference: SED-ML Level 1 Version 5, Section 2.1.4.)

**25503** ☑ An AppliedDimension object may have the optional attributes `target` and `dimensionTarget`. (Reference: SED-ML Level 1 Version 5, Section 2.1.8.4.)

**25504** ☑ The value of The value of the `target` attribute of an AppliedDimension must be the identifier of an existing RepeatedTask or SubTask object in the document, or the identifier of a Task referenced by a SubTask. (Reference: SED-ML Level 1 Version 5, Section 2.1.8.4)

**25505** ☑ The value of the `dimensionTarget` of an AppliedDimension must be the identifier of a dimension of the referenced data. (Reference: SED-ML Level 1 Version 5, Section 2.1.8.4)

**25550** ☑ Every AppliedDimension must have either its `target` attribute or its `dimensionTarget` attribute set, but not both. (Reference: SED-ML Level 1 Version 5, Section 2.1.8.4)

#### *Rules for DataRange objects*

**25601** ☑ A DataRange object may have the optional SED-ML Level 1 attributes `id`, `name`, and `metaid`. (Reference: SED-ML Level 1 Version 5, Section 2.1.4.)

**25602** ☑ A DataRange object may have the optional SED-ML Level 1 subobjects for notes and annotations. (Reference: SED-ML Level 1 Version 5, Section 2.1.4.)

**25603** ☑ A DataRange object must have the required attribute `sourceRef`. (Reference: SED-ML Level 1 Version 5, Section 2.2.8.3.5.)

**25604** ☑ The value of the attribute `sourceRef` of a DataRange object must be the identifier of an existing DataDescription object defined in the document. (Reference: SED-ML Level 1 Version 5, Section 2.2.8.3.5.)

#### *Rules for ShadedArea objects*

**25701** ☑ A ShadedArea object may have the optional SED-ML Level 1 attributes `id`, `name`, and `metaid`. (Reference: SED-ML Level 1 Version 5, Section 2.1.4.)

**25702** ☑ A ShadedArea object may have the optional SED-ML Level 1 subobjects for notes and annotations. (Reference: SED-ML Level 1 Version 5, Section 2.1.4.)

**25703** ☑ A ShadedArea object must have the required attributes `yDataReferenceFrom` and `yDataReferenceTo`. (Reference: SED-ML Level 1 Version 5, Section 2.2.12.6.)

**25704** ☑ The value of the attribute `yDataReferenceFrom` of a ShadedArea object must be the identifier of an existing DataGenerator object defined in the document. (Reference: SED-ML Level 1 Version 5, Section 2.2.12.6.)

25705 ☑ The value of the attribute `yDataReferenceTo` of a ShadedArea object must be the identifier of an existing DataGenerator object defined in the document. (Reference: SED-ML Level 1 Version 5, Section 2.2.12.6.)

25750 ☑ The `yDataReferenceFrom` and `yDataReferenceTo` attributes of a ShadedArea must reference data with the same dimensionality as each other. (Reference: SED-ML Level 1 Version 5, Section 2.2.12.6)

***Rules for ParameterEstimationResultPlot objects***

25801 ☑ A ParameterEstimationResultPlot object may have the optional SED-ML Level 1 attributes `id`, `name`, and `metaid`. (Reference: SED-ML Level 1 Version 5, Section 2.1.4.)

25802 ☑ A ParameterEstimationResultPlot object may have the optional SED-ML Level 1 subobjects for notes and annotations. (Reference: SED-ML Level 1 Version 5, Section 2.1.4.)

25803 ☑ A ParameterEstimationResultPlot object must have the required attribute `taskReference`. (Reference: SED-ML Level 1 Version 5, Section 2.2.12.8.)

25804 ☑ The value of the attribute `taskReference` of a ParameterEstimationResultPlot object must be the identifier of an existing ParameterEstimationTask object defined in the document. (Reference: SED-ML Level 1 Version 5, Section 2.2.12.8.)

***Rules for WaterfallPlot objects***

25901 ☑ A WaterfallPlot object may have the optional SED-ML Level 1 attributes `id`, `name`, and `metaid`. (Reference: SED-ML Level 1 Version 5, Section 2.1.4.)

25902 ☑ A WaterfallPlot object may have the optional SED-ML Level 1 subobjects for notes and annotations. (Reference: SED-ML Level 1 Version 5, Section 2.1.4.)

25903 ☑ A WaterfallPlot object must have the required attribute `taskReference`. (Reference: SED-ML Level 1 Version 5, Section 2.2.12.9.)

25904 ☑ The value of the attribute `taskReference` of a WaterfallPlot object must be the identifier of an existing ParameterEstimationTask object defined in the document. (Reference: SED-ML Level 1 Version 5, Section 2.2.12.9.)

***Rules for ParameterEstimationReport objects***

26001 ☑ A ParameterEstimationReport object may have the optional SED-ML Level 1 attributes `id`, `name`, and `metaid`. (Reference: SED-ML Level 1 Version 5, Section 2.1.4.)

26002 ☑ A ParameterEstimationReport object may have the optional SED-ML Level 1 subobjects for notes and annotations. (Reference: SED-ML Level 1 Version 5, Section 2.1.4.)

26003 ☑ A ParameterEstimationReport object must have the required attribute `taskReference`. (Reference: SED-ML Level 1 Version 5, Section 2.2.14.)

26004 ☑ The value of the attribute `taskReference` of a ParameterEstimationReport object must be the identifier of an existing ParameterEstimationTask object defined in the document. (Reference: SED-ML Level 1 Version 5, Section 2.2.14.)

***Rules for Analysis objects***

26101 ☑ An Analysis object may have the optional SED-ML Level 1 attributes `id`, `name`, and `metaid`. (Reference: SED-ML Level 1 Version 5, Section 2.1.4.)

26102 ☑ An Analysis object may have the optional SED-ML Level 1 subobjects for notes and annotations. (Reference: SED-ML Level 1 Version 5, Section 2.1.4.)
